# Influence of environmental conditions and seasonality on the metabolome and lipidome of *Psychotria viridis* leaves

**DOI:** 10.1111/tpj.70353

**Published:** 2025-07-22

**Authors:** Taynara Simão Matos, Camila Dias Lourenço Dos Santos, Luís Fernando Tófoli, Ílio Montanari Júnior, Márcia Cristina Breitkreitz, Alessandra Sussulini

**Affiliations:** ^1^ Laboratory of Bioanalytics and Integrated Omics (LaBIOmics), Institute of Chemistry Universidade Estadual de Campinas (UNICAMP) Campinas São Paulo Brazil; ^2^ Interdisciplinary Cooperation for Ayahuasca Research and Outreach (ICARO), School of Medical Sciences Universidade Estadual de Campinas (UNICAMP) Campinas São Paulo Brazil; ^3^ Faculty of Pharmaceutical Sciences Universidade Estadual de Campinas (UNICAMP) Campinas São Paulo Brazil; ^4^ Multidisciplinary Center for Chemical, Biological and Agricultural Research (CPQBA) Universidade Estadual de Campinas (UNICAMP) Campinas São Paulo Brazil; ^5^ National Institute of Science and Technology of Bioanalytics (INCTBio), Institute of Chemistry Universidade Estadual de Campinas (UNICAMP) Campinas São Paulo Brazil

**Keywords:** *Psychotria viridis*, seasonality, abiotic stress, LC–MS, GC–MS, metabolomics, lipidomics

## Abstract

*Psychotria viridis* Ruiz & Pav. has gained significant attention due to its role in the preparation of ayahuasca. This study aimed to improve the understanding of the specialized metabolite profile in *P. viridis* leaves and to evaluate how growing conditions and seasonality impact this composition. The specimens were grown either in the open field or in the shaded environment of rubber tree (*Hevea brasiliensis* L.) cultivation, forming a clonal population of the mother plant. Samples were collected in all four seasons of the year. After a three‐phase extraction of the samples, the aqueous and organic phases were analyzed using an ultra‐high‐performance liquid chromatography coupled with electrospray ionization and Orbitrap mass spectrometry (UHPLC‐ESI‐Orbitrap‐MS) system. The acquired data were processed using MS‐DIAL 4.9 and MetaboAnalyst 5.0 for multivariate statistics and pathway activity analysis. Chemical variations were investigated employing principal component analysis (PCA), hierarchical cluster analysis (HCA), and partial least squares discriminant analysis (PLS‐DA). The most important identified compounds for differentiation according to seasonality were flavonoids. The pathways presenting significant variation in response to seasonality were related to energy generation through biosynthesis and consumption of carbohydrates: ascorbate and aldarate metabolism, pentose and glucuronate interconversions, and citrate cycle. Meanwhile, the biosynthesis of flavonoids, flavones, and flavonols was associated with the influence of the cultivation location in full sunlight or shade in an intercrop, indicating a plant response to oxidative stress. In our comprehensive analysis, DMT concentrations did not exhibit any significant statistical variation across the studied conditions.

## Significance Statement

The metabolic pathways presenting significant variation in response to seasonality were associated with energy production through the biosynthesis and consumption of carbohydrates. In contrast, the biosynthesis of flavonoids, flavones, and flavonols was linked to the cultivation location, whether in full sunlight or shaded intercrop, indicating a plant response to oxidative stress.

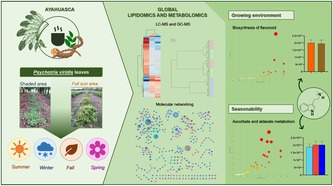

## INTRODUCTION

*Psychotria viridis*, popularly known as ‘chacruna’ among other names, has drawn significant attention due to its use in ayahuasca, a psychedelic indigenous brew recognized for its antidepressant, anxiolytic, and antiaddictive properties (Estrella‐Parra et al., 2019). *Psychotria viridis*, first described by Ruiz and Pavón in 1779, is generally found in humid forests and typically grows as a shrub. A morphological, anatomical, and histochemical study revealed variations in the leaf curl pattern, reduction in leaf area, and starch depletion in *P. viridis* leaves, indicating an effective strategy for tolerance to seasonal water stress (Miranda et al., 2020). Among the specialized metabolites of *P. viridis* leaves, triterpenes, steroids, hydrocarbons, and alkaloids have been isolated and characterized, with *N,N*‐dimethyltryptamine (DMT) being the most abundant (Callaway et al., 2005; Soares et al., 2017).

In the literature, it is often highlighted how conventional farming practices can have negative impacts on the environment and adversely affect the food and plants grown under these conditions (Oliveira et al., 2022; Rehberger et al., 2023). In contrast, regenerative agriculture practices emerge as a promising alternative to the conventional approach, with an emphasis on soil conservation (Rehberger et al., 2023). Ayahuasca groups, inspired by indigenous *ayahuasqueiro* traditions, have adopted regenerative practices to cultivate *Banisteriopsis caapi* (Spruce ex Griseb.) Morton and *P. viridis*, which are fundamental in the preparation of ayahuasca (Thevenin & Sambuichi, 2020). This approach not only aims to ensure environmental sustainability but also highlights a beneficial synergy between agricultural practices, the cultivation of important plants for specific uses, such as ayahuasca preparation, and the preservation of traditional knowledge.

**Figure 1:**
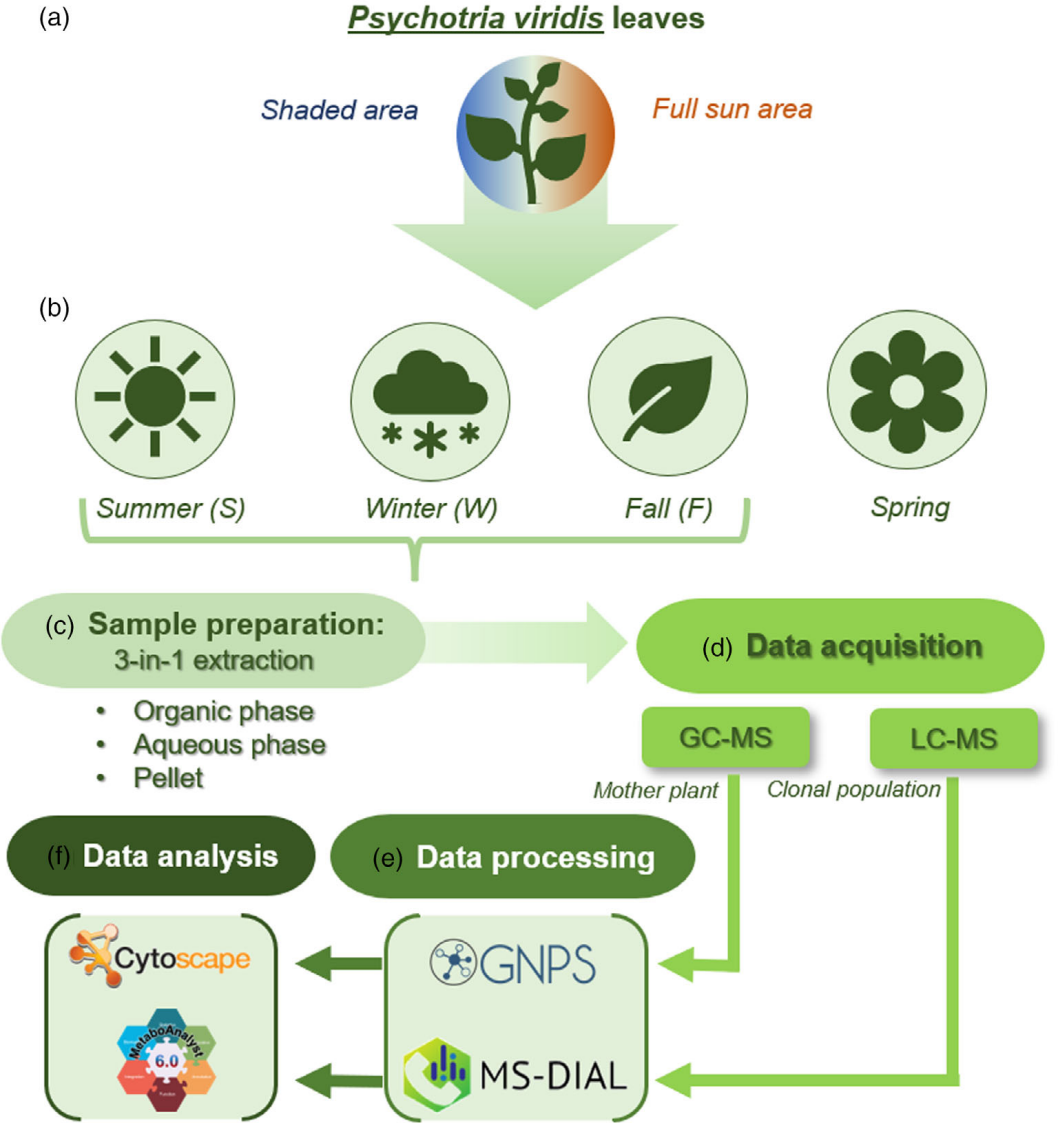
Schematic representation of experimental design and data analysis. (a) *Psychotria viridis* Ruiz & Pav. leaves cultivated in two different environmental conditions: a shaded area due to agroforestry with *Hevea brasiliensis* L., and a full sunlight area. (b) Leaves collection in different seasons. (c) Sample preparation was based on a three‐in‐one simultaneous extraction, where the organic phase was used for lipidomics analysis and the aqueous phase for metabolomics analysis. (d) Data acquisition was based on GC–MS (mother plant samples) and LC–MS (clonal population samples). (e) GC–MS data was processed in the GNPS platform while LC–MS data was processed with MS‐DIAL software. (f) GNPS archives were analyzed with Cytoscape while MetaboAnalyst was used to analyze MS‐DIAL archives.

Although *P. viridis* has been studied using different analytical platforms, most of them focus solely on DMT analysis. In this context, our study is pioneering by using an untargeted approach (Callaway et al., 2005; Cavalcante et al., 2018; Kowalczuk et al., 2015; Soares et al., 2017). Considering the importance of understanding the compounds present in this species, this study aimed to expand the knowledge of the specialized metabolites composition in the leaves of *P. viridis* and investigate how both the growing environment and seasonality influence this composition. In this study, a three‐phase extraction method was adopted, followed by analyses at different omics levels (lipidomics and metabolomics using LC–MS and GC–MS platforms) on leaves of the species, cultivated in both an agroforestry environment and under conditions of continuous sun exposure (Figure 1), in different seasons.

## RESULTS AND DISCUSSION

### Overview of the metabolome and lipidome of *P. viridis* leaves: flavonoids and very long carbon chain lipids increase due to temperature variation

**Figure 2:**
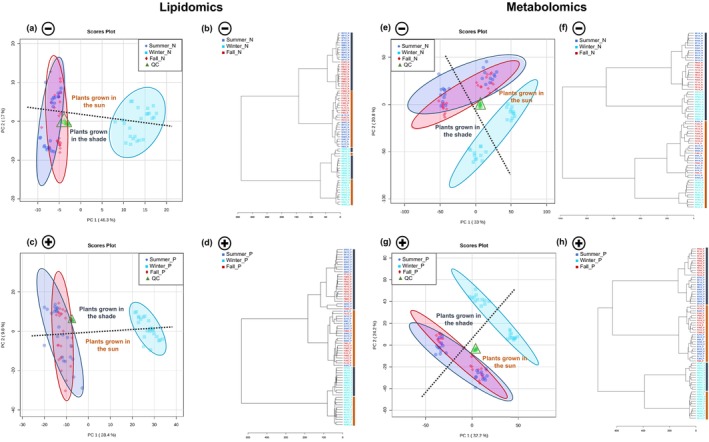
Exploratory analysis of lipidomics and metabolomics of samples analyzed by LC–MS. (a–d) Lipidomics data. (e–h) Metabolomics data. Ionization mode: Negative (N) or Positive (P). QC: Quality control. (a), (c), (e), and (g) PCA scores plot. (b), (d), (f), and (h) HCA dendrogram. Symbols indicate the ionization mode: a plus sign represents positive mode, and a minus sign represents negative mode. In the PCA score plots, the dashed lines highlight the separation of samples based on growth conditions, while in the HCA plot, the unbroken lines (colored to match the text) distinguish plants grown in the shade (dark blue) from those grown in the sun (dark orange).

Exploratory analysis of the LC–MS data can provide an overview of the samples according to seasonality and cultivation conditions. Clustering of the QC samples in the principal component analysis (PCA) score plots (Figure 2a,c,e,g) indicates instrumental stability throughout the analyses. Both lipidomics and metabolomics data (obtained from organic and aqueous phases of the extracted *P. viridis* leaf samples, respectively), in negative and positive ionization modes, present a clear separation of the winter samples, while fall and summer samples demonstrate higher similarity. Hierarchical cluster analysis (HCA) (Figure 2b,d,f,h) confirms what is observed by PCA. Lipidomics and metabolomics data also display a separation of the samples according to the cultivation area (sun exposure and shaded area). Overall, these results suggest that the compounds present in both organic and aqueous phases contribute to the seasonality effect. Additionally, an analysis of the aqueous phase offers further insights into compound variation according to environmental conditions.

Partial least squares discriminant analysis (PLS‐DA) was applied to identify which compounds are important for sample differentiation by season (Figure S1). Most of the statistically significant compounds (variable importance in projection [VIP] score > 1) of the aqueous phase were more abundant in fall samples and lower in winter samples (Figure S2).

Considering the LC–MS aqueous phase data and fragmentation pattern in both ionization modes (Tables S1 and S2), 35 compounds were selected using spectral similarity (cos > 0.8). Most of these compounds belong to the flavonoid chemical class (Table 1). Interestingly, these flavonoids were more abundant in the winter samples (Figure S2). A similar trend was observed in *Arbustus unedo* leaves (Sanna et al., 2023), where a drop in temperature triggered oxidative stress in the plant, leading to an increased production of these antioxidant compounds.

**Table 1:** Metabolites annotated as statistically significant according to the seasons (aqueous phase)

#	rt_*m/z*	Metabolite name	Molecular formula	Adduct	VIP	cos	Compound class
1	5.586_289.0718	**Epicatechin**	C_15_H_14_O_6_	[M − H]^−^	1.59	0.90	Flavonoid
2	4.967_305.0667	Epigallocatechin	C_15_H_14_O_7_	[M − H]^−^	1.54	0.87	Flavonoid
3	5.139_289.0718	**Catechin**	C_15_H_14_O_6_	[M − H]^−^	1.37	0.89	Flavonoid
4	6.974_144.0455	2‐Hydroxyquinoline	C_9_H_7_NO	[M − H]^−^	1.35	0.88	Quinoline
5	7.228_261.1345	9‐(2,3‐Dihydroxypropoxy)‐9‐oxononanoic acid	C_12_H_22_O_6_	[M − H]^−^	1.35	0.84	Fatty acid
6	6.307_175.0249	Ascorbic acid	C_6_H_8_O_6_	[M − H]^−^	1.35	0.84	Carbohydrate
7	6.337_463.0885	**Hyperoside**	C_21_H_20_O_12_	[M − H]^−^	1.24	0.94	Flavonoid
8	6.081_595.1312	**2‐(3,4‐Dihydroxyphenyl)‐5,7‐dihydroxy‐3‐[‐3,4,5‐trihydroxy‐6‐[[3,4,5‐trihydroxyoxan‐2‐yl]oxymethyl]oxan‐2‐yl]oxychromen‐4‐one**	C_26_H_28_O_16_	[M − H]^−^	1.21	0.86	Flavonoid
9	6.743_433.0787	Guajavarin	C_20_H_18_O_11_	[M − H]^−^	1.18	0.93	Flavonoid
10	7.678_187.0974	Azelaic acid	C_9_H_16_O_4_	[M − H]^−^	1.17	0.90	Fatty acid
11	6.680_549.0902	**Quercetin 3‐*O*‐malonylglucoside**	C_24_H_22_O_15_	[M − H]^−^	1.14	0.84	Flavonoid
12	8.450_263.1293	Abscisic acid	C_15_H_20_O_4_	[M − H]^−^	1.09	0.91	Carboxylic acid
13	6.167_206.0820	N‐acetylphenylalanine	C_11_H_13_NO_3_	[M − H]^−^	1.08	0.83	Amino acid
14	6.986_515.1197	3,5‐Di‐*O*‐caffeoyl quinic acid	C_25_H_24_O_12_	[M − H]^−^	1.08	0.84	Carboxylic acid
15	8.500_301.0356	Quercetin	C_15_H_10_O_7_	[M − H]^−^	1.06	0.87	Flavonoid
16	0.645_191.0562	Quinic acid	C_7_H_12_O_6_	[M − H]^−^	1.03	0.89	Carboxylic acid
17	2.579_153.0194	Patulin	C_7_H_6_O_4_	[M − H]^−^	1.01	0.88	Lactone
18	0.741_198.0760	Levodopa	C_9_H_11_NO_4_	[M + H]^+^	1.69	0.91	Amino acid
19	5.583_291.0859	**Epicatechin**	C_15_H_14_O_6_	[M + H]^+^	1.55	0.86	Flavonoid
20	0.667_360.1505	Gentiobiose	C_12_H_22_O_11_	[M + NH_4_]^+^	1.54	0.89	Carbohydrate
21	10.53_333.2036	Dihydroalbocycline	C_18_H_30_O_4_	[M + Na]^+^	1.54	0.80	Lactone
22	0.653_365.1046	Melibiose	C_12_H_22_O_11_	[M + Na]^+^	1.46	0.91	Carbohydrate
23	5.135_291.0858	**Catechin**	C_15_H_14_O_6_	[M + H]^+^	1.41	0.86	Flavonoid
24	6.34_465.1029	**Hyperoside**	C_21_H_20_O_12_	[M + H]^+^	1.28	0.88	Flavonoid
25	6.076_597.1442	**2‐(3,4‐Dihydroxyphenyl)‐5,7‐dihydroxy‐3‐[‐3,4,5‐trihydroxy‐6‐[[‐3,4,5‐trihydroxyoxan‐2‐yl]oxymethyl]oxan‐2‐yl]oxychromen‐4‐one**	C_26_H_28_O_16_	[M + H]^+^	1.24	0.85	Flavonoid
26	6.737_435.0922	Avicularin	C_20_H_18_O_11_	[M + H]^+^	1.19	0.88	Flavonoid
27	5.154_759.1968	3‐[‐4‐[‐4,5‐Dihydroxy‐6‐(hydroxymethyl)‐3‐[‐3,4,5‐trihydroxyoxan‐2‐yl]oxyoxan‐2‐yl]oxy‐3,5‐dihydroxy‐6‐(hydroxymethyl)oxan‐2‐yl]oxy‐2‐(3,4‐dihydroxyphenyl)‐5,7‐dihydroxychromen‐4‐one	C_32_H_38_O_21_	[M + H]^+^	1.19	0.80	Flavonoid
28	6.967_375.2371	2‐(Hydroxymethyl)‐6‐[4‐(4‐hydroxy‐2,6,6‐trimethylcyclohexen‐1‐yl)butan‐2‐yloxy]oxane‐3,4,5‐triol	C_19_H_34_O_7_	[M + H]^+^	1.19	0.87	Carbohydrate
29	0.643_104.0706	Gamma‐aminobutyric acid	C_4_H_9_NO_2_	[M + H]^+^	1.17	0.88	Amino acid
30	6.675_551.1016	**Quercetin 3‐*O*‐malonylglucoside**	C_24_H_22_O_15_	[M + H]^+^	1.16	0.91	Flavonoid
31	0.679_205.1177	1‐[4‐Hydroxy‐3‐(3‐methylbut‐2‐enyl)phenyl]ethanone	C_13_H_16_O_2_	[M + H]^+^	1.13	0.83	Ketone
32	0.742_289.0918	2‐Methyl‐3‐[‐3,4,5‐trihydroxy‐6‐(hydroxymethyl)oxan‐2‐yl]oxypyran‐4‐one	C_12_H_16_O_8_	[M + H]^+^	1.12	0.88	Carbohydrate
33	6.746_287.0543	Fisetin	C_15_H_10_O_6_	[M + H]^+^	1.04	0.91	Flavonoid
34	6.979_449.1075	4‐(3,4‐Dihydroxyphenyl)‐7‐hydroxy‐5‐[‐3,4,5‐trihydroxy‐6‐(hydroxymethyl)oxan‐2‐yl]oxychromen‐2‐one	C_21_H_20_O_11_	[M + H]^+^	1.04	0.87	Coumarin
35	12.481_357.3000	Monoolein	C_21_H_40_O_4_	[M + H]^+^	1.04	0.87	Glycerolipid

Compound names in bold were suggested as significant for differentiation in the two ionization modes.

Flavonoid is a vast chemical class with diverse functions in plants. They can attract pollinators, regulate cell growth, eliminate oxidative reactive species, and respond to biotic and abiotic stress, such as herbivory and temperature variations (Dias et al., 2021). In addition to flavonoids, primary metabolites such as amino acids, carboxylic acids, and carbohydrates have been suggested (Table 1). They have a crucial function in plant development and growth (Sweetlove et al., 2010).

**Figure 3:**
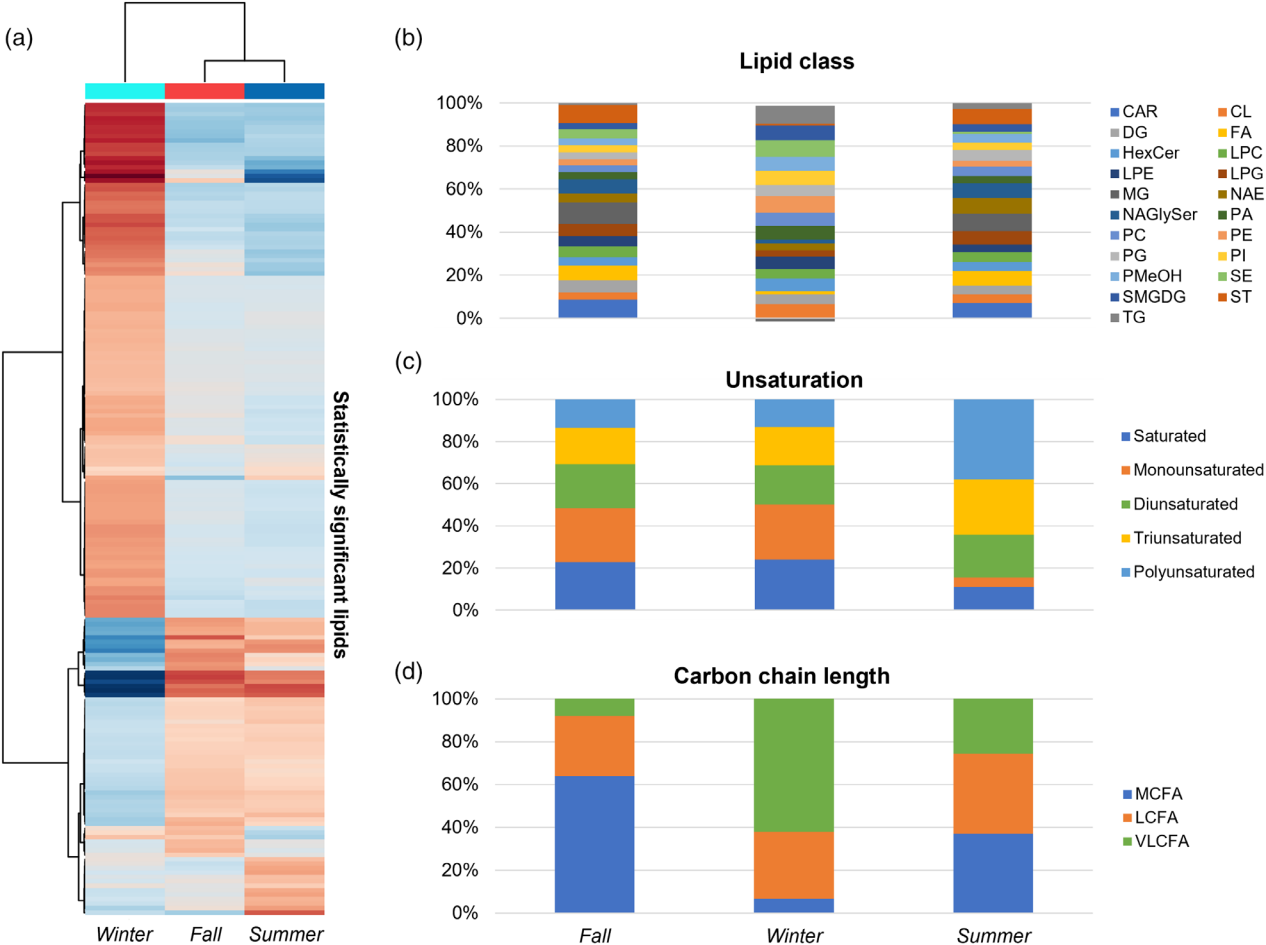
Overview of the lipid profile in leaves of *Psychotria viridis* according to seasonality by LC–MS data. (a) Heatmap with the average of the intensities of the significant lipids indicated by the two ionization modes. (b–d) Proportion of the different characteristics of lipids by seasonality. (d) Carbon chain length of the fatty acids as medium, long, and very long fatty acids (MCFA: >5 & ≤12; LCFA: >12 & ≤21; VLCFA: >21).

Annotation of the lipids present in the organic phase LC–MS data, based on the *in silico* MS‐DIAL internal library, identified compounds from data acquired in both ionization modes. These compounds fall into several chemical classes (Tables S3 and S4—acronym list on Supporting Information S1): carnitine (CAR), lysophosphatidylethanolamine (LPE), phosphatidylcholine (PC), seminolipid (SMGDG), cardiolipin (CL), lysophosphatidylglycerol (LPG), phosphatidylethanolamine (PE), sterol lipids (ST), diacylglycerol (DG), monoacylglycerol (MG), phosphatidylglycerol (PG), triacylglycerol (TG), free fatty acid (FA), N‐acyl glycyl serine (NAGlySer), phosphatidylmethanol (PMeOH), lysophosphatidylcholine (LPC), phosphatidic acid (PA), and sterol ester (SE). A total of 183 lipids selected by VIP score >1 (PLS‐DA) were considered statistically significant for seasonal differentiation. These lipids were used to generate a heatmap illustrating lipid fluctuations across seasons (Figure 3a), originated from several chemical classes (Figure 3b). Additionally, certain characteristics of the carbon chains, such as unsaturation (Figure 3c) and carbon chain length (Figure 3d) are noteworthy. An increase in lipids with polyunsaturated carbons was observed in the winter samples. However, the main difference across the seasons is observed in the carbon chain length, with an increase in summer and winter samples. An increase in both unsaturation and carbon chain length in the foliar membrane has been associated with increased foliar fluid, serving as a plant adaptation strategy to temperature variation (Chaves et al., 2024; He & Ding, 2020).

Positive ionization mode data from the organic phase was also processed on MZmine3, and some compounds were annotated with GNPS2 (Figure S3). For compound annotation, a cosine value higher than 0.8 was used (Table S5). As expected, more than half of the compounds annotated at level 3 confidence (Reisdorph et al., 2019) are from the fatty acid class, followed by terpenoids and alkaloids (Figure S3). It was suggested that some biochemical pathways were more abundant according to the seasons and growth area (Figures S4 and S5). Fall exhibits the highest diversity of biochemical classes, Summer shows a higher abundance of fatty acids, and Winter highlights terpenoids. When considering biochemical pathways and cultivation condition color patterns, two distinct regions emerge: Plants grown in full sunlight display greater biochemical class diversity, while those cultivated in shaded areas are enriched in fatty acids and terpenoids. DMT abundance was constant in all comparisons (Figure S6).

### Chemical classes in *P. viridis* leaves according to seasonality

**Figure 4:**
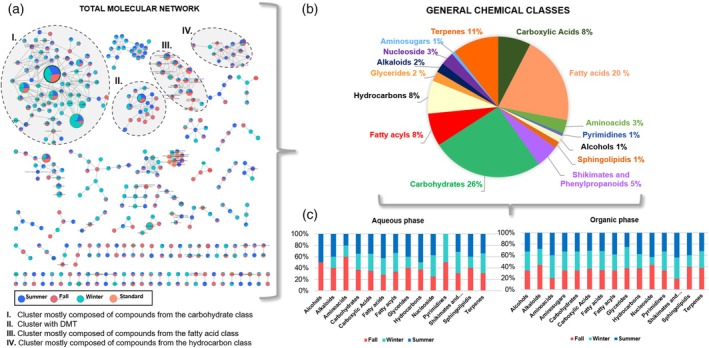
Overview of the metabolic profile in leaves of *Psychotria viridis* according to seasonality by GC–MS data. (a) Molecular networking was generated after analysis via GNPS. (b) Percentage of chemical classes of the annotated compounds. (c) Distribution of the chemical classes through seasons in the different extractions.

To gain more information about the metabolite diversity of *P. viridis* across different seasons, a molecular network was generated on GNPS using GC–MS data (Figure 4a). The molecular network consists of 609 edges connecting 393 nodes representing possible compounds. The inner colors of the nodes represent the compound intensity based on peak area in each season, and the node size is adjusted according to the intensity in the samples. As a result, it is possible to point out that the major compounds in the samples are quinic acid and 1‐kestose in Cluster I, DMT in Cluster II, palmitic acid and linoleic acid in Cluster III, and tetratriacontane in Cluster IV. Additionally, outside the highlighted clusters, compounds such as supraene and alpha‐tocopherol are present in relevant abundance in the samples.

By analyzing the Clusters (I, II, III, and IV) of the molecular network (Figure 4a), we can infer the prevalence of different chemical classes across seasons (Table S6). In Cluster I, most of the annotated compounds belong to the carbohydrate class and are more abundant in winter samples. Cluster II includes DMT and D‐galactosamine annotated, with DMT distributed equally across seasonal samples. Cluster III is mainly represented by fatty acid compounds in fall samples, while Cluster IV consists of hydrocarbon class compounds in summer samples.

The annotated compounds belong to different chemical classes, as presented in Figure 4b. Most of them are carbohydrates, fatty acids, and terpenes. It is also evident that the organic phase was more informative than the aqueous phase (Figure 4c). Figure 4c displays that alcohols and pyrimidines are present in all three analyzed seasons according to the organic phase, which differs from the aqueous phase. The exact number of compounds by class in each sample displayed in Figure 4c is presented in Table S7.

The presence of carbohydrates is inherent to plant growth, serving as the foundational components for structural polysaccharides such as cellulose and playing a vital role in energy production. According to Miranda *et al*.'s study, a reduction in the rate of starch (a polysaccharide synthesized through the polymerization of glucose during photosynthesis) accumulation was linked to seasonal water stress experienced by *P. viridis* plants (Miranda et al., 2020). During winter, plants rely on carbohydrate reserves to sustain themselves and protect against temperature‐induced damage (Guo et al., 2024). Additionally, carbohydrates mediate water stress, with their concentrations typically increasing during drought periods (Signori‐Müller et al., 2021). Consequently, the elevated levels of carbohydrates observed during this season align with the anticipated reduction in energy expenditure and the plant response to water stress.

**Figure 5:**
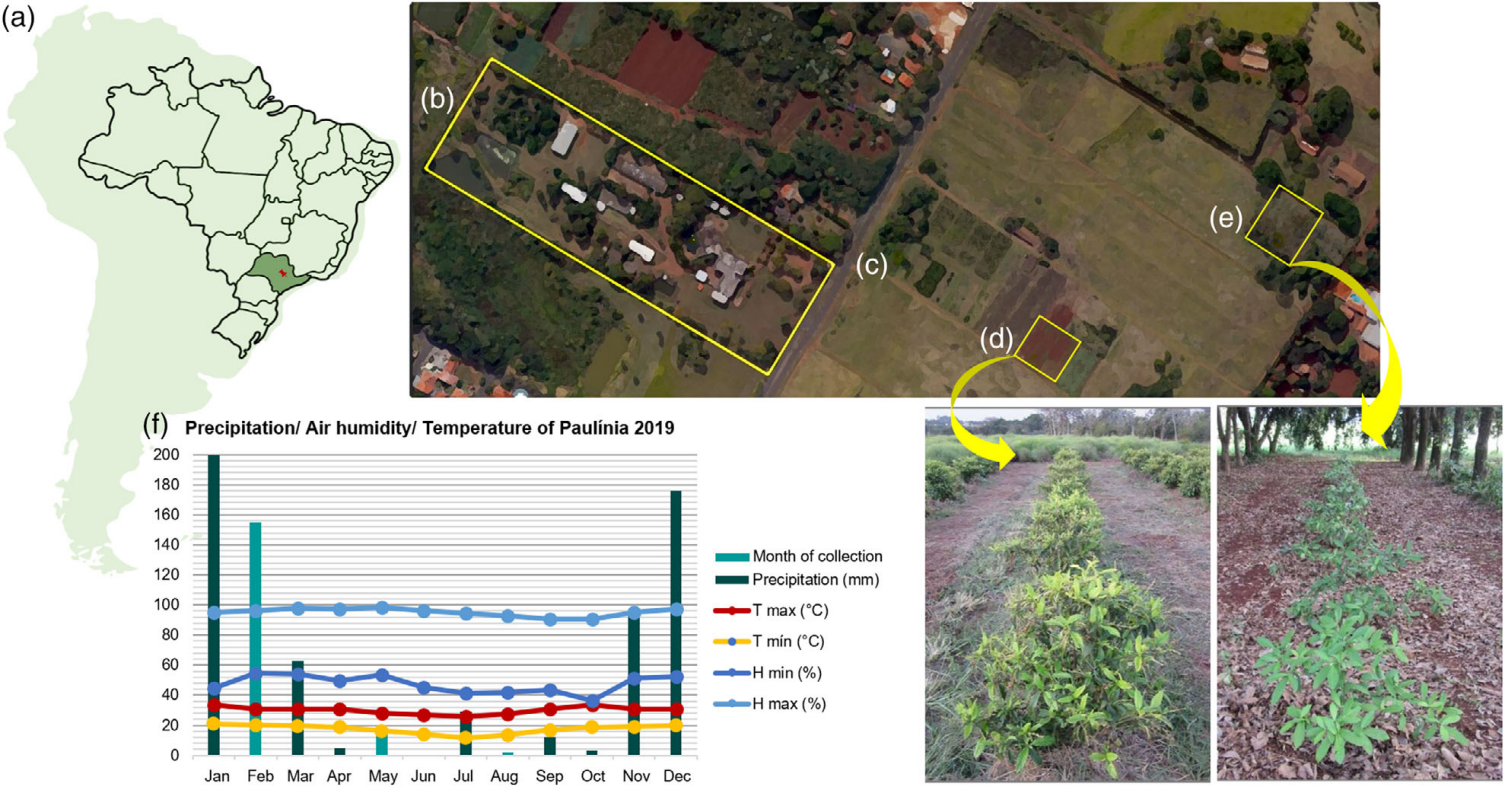
Overview of the sample collection location. (a) Geographical location of the cultivation. (b) Greenhouse of the Department of Agrotechnology, placed in the laboratory complex delimited by the rectangle. (c) Location of the mother plant of *Psychotria viridis*. (d) Full sunlight cultivation area. (e) Shaded with rubber tree cultivation area. (f) Environmental conditions across 2019 in Paulínia, SP, Brazil. Data provided by the Agrometeorological and Hydrological Portal of the State of São Paulo. There was a failure in collecting precipitation in the month of June.

Additionally, the combination of carbohydrates with organic acids, or specific proportions of different carbohydrates such as sucrose and malic acid (both identified in Cluster I), forms natural deep eutectic solvents (NADES). These liquids aid plant stabilization under extreme temperatures or drought conditions. NADES prevent freezing due to their low melting points, preserve cell viability, and are associated with the germination process and drought resistance (Choi et al., 2011; Dai et al., 2021). This is particularly significant when examining Figure 5f, which illustrates that August (winter) is the month with the lowest precipitation volume. Furthermore, NADES enhance the solubility of specialized metabolites of intermediate polarity, such as flavonoids and ascorbate (Dai et al., 2014, 2021). Consequently, these solvents play a crucial role in protecting membranes and cellular compartments from oxidative stress.

Within Cluster I (Figure 4a), quinic acid is present in substantial abundance across the samples and exhibits similar proportions among the different seasons, as indicated by the GC–MS analysis. However, it has been identified as a significant compound for differentiating seasons in the data obtained by LC–MS (Table 1). Quinic acid is both a precursor and product in the shikimate pathway, which is a biosynthetic route for aromatic amino acids, including phenylalanine, tyrosine, and tryptophan, derived from carbohydrates. This compound, along with its derivatives, is extensively documented and found in notable quantities in other plants of the Rubiaceae family, such as *Coffea* species, where it contributes to the plant defense mechanisms against pathogens, herbivores, and abiotic stresses, including salinity (Clifford et al., 2017).

Despite fall and summer samples displaying similarities in both organic and aqueous phases analyzed by LC–MS (Figures 2 and 3), GC–MS data suggest some trends. The leaf abscission phase concludes with senescence. This phase is further subdivided, during which nutrients, including nitrogen and carbohydrates, are mobilized to other parts of the plant, accompanied by a reduction in photosynthetic activity (Juvany et al., 2013). This decline in photosynthetic activity during senescence leads to oxidative damage in the leaf. Therefore, fatty acids, which constitute the majority in Cluster III (Figure 4a), play a significant role in the response of the plants to abiotic stresses, mainly as antioxidants against reactive oxygen species (He & Ding, 2020).

On the other hand, hydrocarbons annotated in Cluster IV (Figure 4a) were particularly prevalent during the summer. 2,4‐Dimethyl‐1‐heptene was identified exclusively in the summer samples. This compound has been suggested to be modified among the volatile compounds in sunflower seeds (*Helianthus annuus* L.) following treatment with fumigants phosphine, methyl bromide, or 1,2‐dichloroethane, indicating that it may be associated with the plant defense mechanisms (Austel et al., 2017). This season represents a time of heightened energetic activity for the plant, encompassing growth, reproduction, and photosynthesis, as well as a phase marked by increased production of reactive oxygen species due to elevated light levels and herbivore attacks (Lemoine et al., 2014; Skovmand et al., 2024). Additionally, the presence of reactive oxygen species leads to the degradation of fatty acids and aldehydes, resulting in the formation of hydrocarbons (Bortoluzzi et al., 2008; Grant et al., 2015; Monteiro et al., 2022). This process accounts for the increased abundance of these compounds in the samples collected during summer.

### Biochemical pathways in *P. viridis* leaves according to seasonality

**Figure 6:**
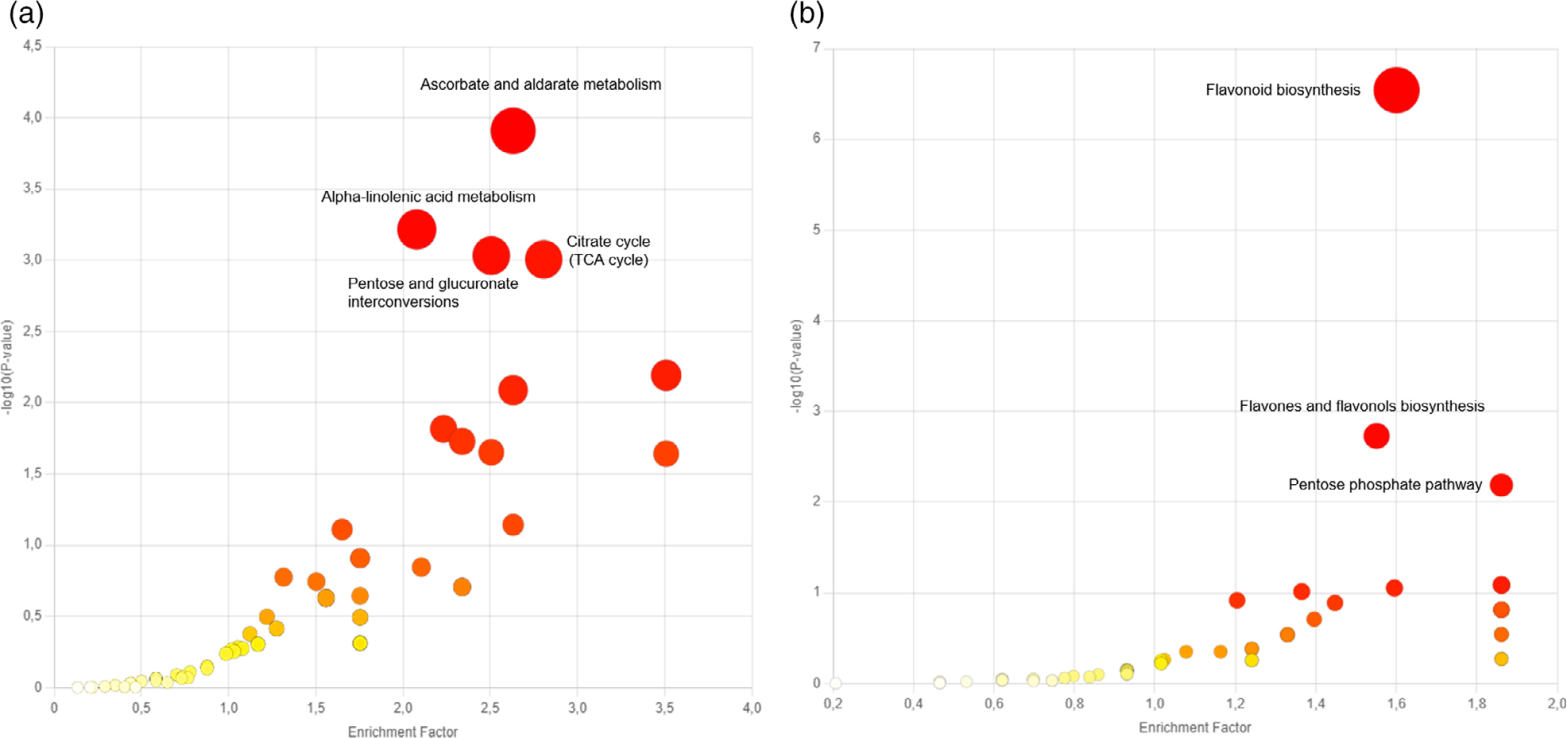
Altered biochemical pathways. (a) Influence of the seasons (autumn and summer versus winter). Data obtained by metabolomics and lipidomics analysis in both ionization modes. (b) Influence of the cultivation (in the shade versus in full sunlight) of data obtained by LC–MS metabolomics analysis in both ionization modes. The circles represent the altered pathways, colors and sizes corresponding to the *P*‐value and enrichment factors, respectively. The pathway enrichment factor is calculated based on the number of compounds annotated in the data and the expected number for that pathway.

After categorizing chemical classes by season, a pathway analysis was performed to obtain a holistic view on the biochemical pathways involved in the differentiation of fall and summer versus winter samples. For this purpose, aqueous and organic phases LC–MS data were used, suggesting 67 pathways for both ionization modes (Figure 6a; Table S8). Among them, the most impacted pathways by seasonality are ascorbate and aldarate metabolism, alpha‐linolenic acid metabolism, pentose and glucuronate interconversions, and citrate cycle (TCA cycle) (Figure 6a). With the exception of alpha‐linolenic acid metabolism, the other metabolic pathways are interconnected and are part of carbohydrate metabolism.

The metabolism of alpha‐linolenic acid, unlike other pathways, belongs to lipid metabolism. Alpha‐linolenic acid is the main unsaturated fatty acid found in cell membranes, playing a crucial role in preserving the integrity of these membranes in cold stress situations (He & Ding, 2020). Several studies have associated the metabolism of this acid with plant tolerance under cold stress, through the suppression of oxidative stress while maintaining a high degree of unsaturation of fatty acids in the cell membrane (Huan et al., 2022; Liu et al., 2021). An increase in fatty acids with a high degree of unsaturation was also observed in bromeliads as the altitude of the collection site increased, being associated with the cold tolerance of the species (Chaves et al., 2024). By analyzing the lipidomics results, it is possible to observe an increase in polyunsaturated lipids (Figure 3) in the winter samples. In this context, the change in the alpha‐linolenic acid pathway could be associated with the adaptation of the leaves to the drop in temperature that occurs naturally in the winter compared to the other seasons of the year.

The metabolism of ascorbate and aldarate generates intermediate metabolites for two other altered pathways: pentose and glucuronate interconversions and the citrate cycle. Ascorbate has a high antioxidant capacity, acting in the elimination of free radicals and in the detoxification of hydrogen peroxide generated by photorespiration and photosynthetic electron transport (Smirnoff, 2018). This pathway, as well as the flavonoid biosynthesis pathway, has been associated with delayed ripening of some fruits due to its antioxidant capacity (Bai et al., 2024). Both the metabolism of ascorbate and aldarate and alpha‐linolenic acid have also been associated with other stages of high‐energy consumption in plants, such as germination (Dong et al., 2024). In this context, this biochemical pathway may have been suggested in the differentiation of stages of higher biochemical activity versus that of lower activity (winter) as a plant response to oxidative stress and energy consumption.

The citrate cycle metabolism is the central pathway among the most significative pathways suggested as altered in Figure 6a. This pathway is widely recognized as a set of biochemical reactions responsible for the generation of energy (ATP synthesis) through the biosynthesis and breakdown of carbohydrates and fatty acids. In addition to playing this central role, this metabolic pathway is closely linked to several functions in the cellular metabolism of plants and can operate in non‐cyclic flux depending on the cell type and physiological context (Sweetlove et al., 2010). Considerable efforts have been dedicated to understanding the interaction of this pathway with processes associated with light fluctuations, such as photosynthesis and photorespiration (Fu & Walker, 2023; Noguchi & Yoshida, 2008; Nunes‐Nesi et al., 2007). In this context, the indication of changes in the TCA cycle when comparing the samples analyzed may be related to the variation in the incidence of sunlight seasonal changes. In addition, the citrate cycle generates a variety of intermediate compounds used in the production of organic acids and amino acids, playing a fundamental role in primary metabolism (Sweetlove et al., 2010), which is responsible for the growth and development of plants.

### Influence of sun or shaded areas of cultivation

Anatomical variations in the leaves of *P. viridis* have been reported as strategies for environmental adaptation (Miranda et al., 2020; Santos, 2021). These include differences in leaf area and stomatal density. Additionally, leaf curling during periods of high‐light intensity and the frequent presence of druses have been observed (Miranda et al., 2020). Druses, a form of calcium oxalate crystals commonly found in South American *Psychotria* species, not only offer protection against herbivory, but also help disperse sunlight in plants growing in shaded areas (Franceschi, 2001; Moraes et al., 2011; Quinteiro et al., 2006).

Figure 2 displays that the samples clearly separate according to the cultivation conditions. Subsequently, the fall and summer samples were used to evaluate solely the influence of cultivation conditions as season had a greater effect on the metabolic composition. The key compounds differentiating cultivation conditions were mostly from the flavonoid class, as can be observed in Table 2, with higher abundance in sun‐exposed than shaded samples. Flavonoids are a polyphenolic class of compounds that are well‐known for their antioxidant activity. They eliminate the oxygen reactive species generated by excessive light (Agati et al., 2013).

**Table 2:** Metabolites annotated as statistically significant according to the cultivation condition (aqueous phase)

#	rt_*m/z*	Metabolite name	Molecular formula	Adduct	VIP	cos	Compound class
1	6.743_433.0787	Guajavarin	C_20_H_18_O_11_	[M − H]^−^	1.60	0.93	Flavonoid
2	6.337_463.0885	Hyperoside	C_21_H_20_O_12_	[M − H]^−^	1.58	0.94	Flavonoid
3	7.066_419.0979	Kaempferol 3‐alpha‐L‐arabinopyranoside	C_20_H_18_O_10_	[M + H]^+^	1.81	0.91	Flavonoid
4	6.123_727.2063	5‐Hydroxy‐3‐[3‐hydroxy‐5‐(hydroxymethyl)‐4‐[3,4,5‐trihydroxy‐6‐(hydroxymethyl)oxan‐2‐yl]oxyoxolan‐2‐yl]oxy‐2‐(4‐hydroxyphenyl)‐7‐[3,4,5‐trihydroxy‐6‐methyloxan‐2‐yl]oxychromen‐4‐one	C_32_H_38_O_19_	[M + H]^+^	1.79	0.86	Flavonoid
5	6.46_449.1076	4‐(3,4‐Dihydroxyphenyl)‐7‐hydroxy‐5‐[3,4,5‐trihydroxy‐6‐(hydroxymethyl)oxan‐2‐yl]oxychromen‐2‐one	C_21_H_20_O_11_	[M + H]^+^	1.79	0.87	Coumarin
6	5.958_449.1077	Luteolin 4'‐*O*‐glucoside	C_21_H_20_O_11_	[M + H]^+^	1.76	0.84	Flavonoid
7	5.874_611.1615	Rutin	C_27_H_30_O_16_	[M + H]^+^	1.70	0.87	Flavonoid
8	6.495_303.0491	4‐(2‐Methoxy‐2‐oxoethyl)‐5‐[2‐[3‐phenylprop‐2‐enoyl]oxyethylidene]‐6‐[3,4,5‐trihydroxy‐6‐(hydroxymethyl)oxan‐2‐yl]oxy‐4H‐pyran‐3‐carboxylic acid	C_15_H_10_O_7_	[M + H]^+^	1.66	0.89	Isoprenoid
9	0.742_289.0918	2‐Methyl‐3‐[3,4,5‐trihydroxy‐6‐(hydroxymethyl)oxan‐2‐yl]oxypyran‐4‐one	C_12_H_16_O_8_	[M + H]^+^	1.54	0.88	Carbohydrate
10	6.978_517.1344	3,4‐Di‐*O*‐caffeoylquinic acid	C_25_H_24_O_12_	[M + H]^+^	1.50	0.83	Carboxylic acid

### Biochemical pathways in *P. viridis* leaves according to cultivation

As exploratory analysis of the aqueous phase samples (LC–MS metabolomics) demonstrated a clear differentiation of the samples due to the cultivation environment (Figure 2), only these data were used for this discussion. The flavonoid, flavone, and flavonol biosynthesis pathway, together with the pentose phosphate pathway, were identified as the most altered biochemical pathways (Figure 6b; Table S9).

Antioxidants, flavonoids, together with ascorbic acid, act to eliminate reactive oxygen species generated by excessive light stress (Agati et al., 2013). This class of compounds presents a photoprotective effect not only in response to light stress in leaves, but also post‐harvest in fruits (Dias et al., 2021; Zhang et al., 2017; Zhu et al., 2023). In this context, it is biologically plausible that the results indicate a greater change in the flavonoid biosynthesis pathway, given the comparison between cultivation in environments exposed to sunlight and shade.

### What about DMT?

**Figure 7:**
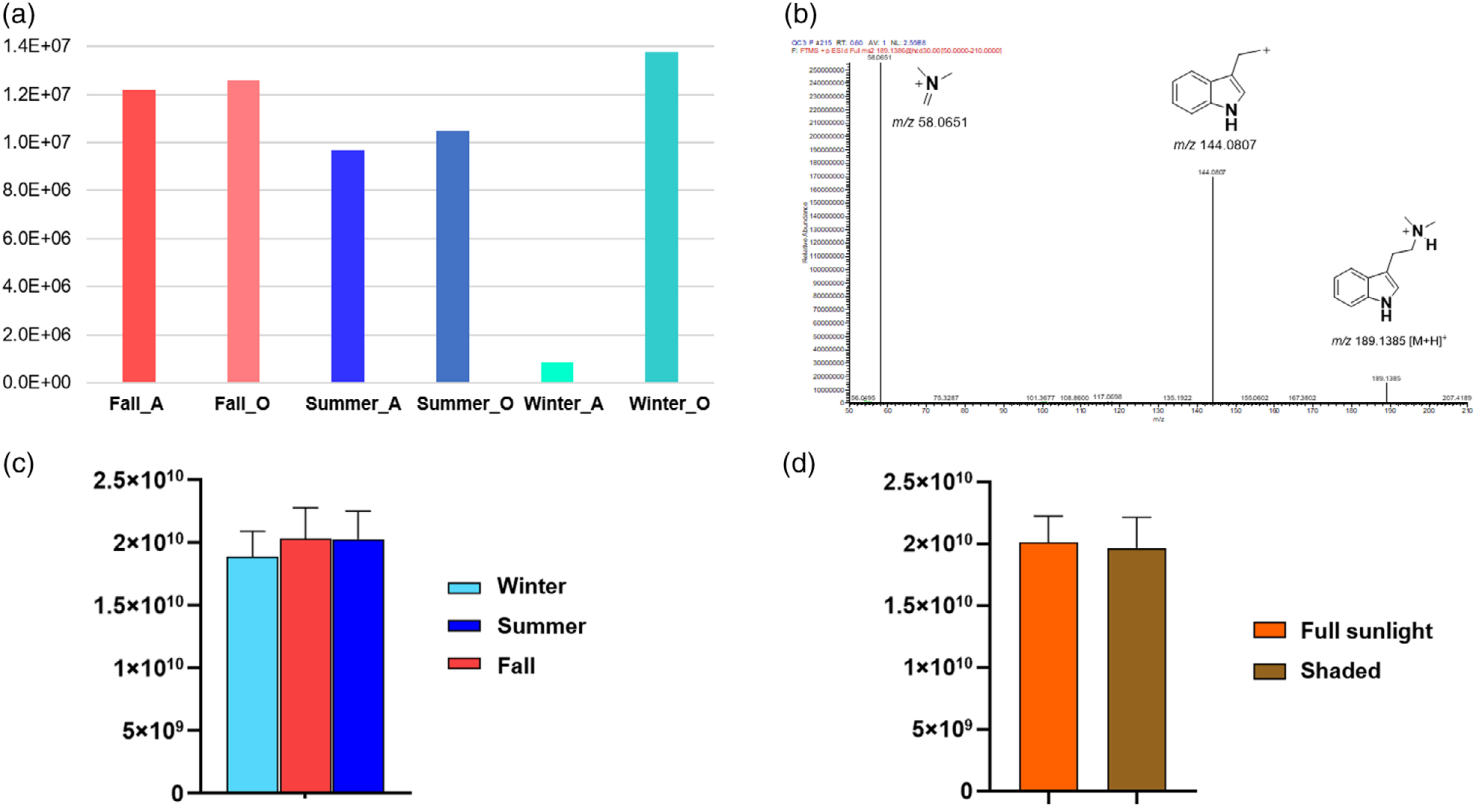
Analysis of DMT on the *Psychotria viridis* leaves. (a) Abundance of DMT through seasons on extracted phases (A: aqueous; O: organic) of GC–MS data. The aqueous phase samples had their values multiplied by 10 to facilitate visualization. (b) Fragmentation pattern of DMT on positive ionization mode. (c) Average ion intensities of DMT on positive ionization mode of organic phase of LC–MS data according to the season. (d) Average ion intensities of DMT through environmental conditions of cultivation (full sunlight and shaded area) on positive ionization mode of organic phase of LC–MS data. Data are represented as mean ± standard deviation, in (c) *n* = 24, in (d) *n* = 36.

DMT is the main compound identified in *P. viridis*, significantly contributing to the psychedelic effects of ayahuasca. According to Callaway, the concentration of DMT in the plant leaves exhibits diurnal variation, peaking at 6 p.m. This fluctuation is hypothesized to provide protection against solar radiation or preservation through radiation absorption (Callaway et al., 2005). In this study, the abundance of the DMT ion was utilized to assess variations in DMT levels under the evaluated environmental conditions (Figure 7).

Analysis of the GC–MS data (Figure 7a) reveals a higher abundance of DMT in the organic phase compared to the aqueous one. Additionally, there is a decreasing trend in DMT abundance in the aqueous phase across the season, following fall > summer > winter. However, GC–MS analyses were performed using a single sample (mother plant samples), which limits the reliability and robustness of the data. Cavalcante *et al*. reported seasonal variations in DMT concentration across different biomes, noting that in the Amazon region, DMT levels remained relatively stable throughout the year (Cavalcante et al., 2018). However, they observed a trend associated with rainfall, with DMT concentration increasing in accordance with the rainfall index during winter. Conversely, an inverse trend was noted in summer, likely due to the dilution of DMT within the plant tissue (Cavalcante et al., 2018).

In contrast to the GC–MS results, LC–MS data had statistical power, since they were based on the clone plants. DMT was identified based on the ion at *m/z* 189.1385 [M + H]^+^ corresponding to the protonated molecule and fragment ions at *m/z* 144.0808 and 58.0652 (Figure 7b), consistent with the molecular formula C_12_H_16_N_2_ (Chen et al., 2018). Figure 7c,d demonstrate that DMT abundance remained constant across the seasons, and cultivation conditions are different from what is reported in the literature. The specimens in this study were cultivated in Paulínia, a region predominantly characterized by the Mata Atlântica followed by Cerrado biome representation (Bargos & Matias, 2012; IBGE, 2024). Among Brazilian biomes, the Cerrado and Mata Atlântica exhibited the lowest average concentration of DMT in *P. viridis* dried leaves compared to other biomes (Cavalcante et al., 2018). DMT concentration varied seasonally across different biomes, where locals in the transition zone Mata Atlântica ↔ Cerrado presented similarly lower DMT abundance in winter compared to other seasons (Cavalcante et al., 2018), consistent with our findings. However, summer DMT levels did not closely align with fall levels. It is noteworthy that the extraction method employed in this study differed from those used by Callaway and Cavalcante, which may account for the discrepancies observed in DMT photoprotection and seasonal abundance (Callaway et al., 2005; Cavalcante et al., 2018). This study aimed for a more comprehensive metabolomic analysis; therefore, the chosen extraction method was not specifically optimized for DMT evaluation.

## EXPERIMENTAL PROCEDURES

### Plant material and cultivation

The leaves of *P. viridis* Ruiz & Pav. were collected at the Centro Pluridisciplinar de Pesquisas Químicas, Biológicas e Agrícolas (CPQBA), in the district of Betel, Paulínia, São Paulo (22°47′42.85″ S; 47°6′39.79″ O). This Center is part of the Universidade Estadual de Campinas (UNICAMP). Specimens were cultivated both in the open field and others in the shaded environment provided by rubber tree cultivation (*Hevea brasiliensis* L.). These specimens constitute a clonal population derived from the mother plant (CPMA item#1745), which is part of the CPQBA Collection of Aromatic and Medicinal Plants (CPMA). This study is registered in the National System for Management of Genetic Heritage and Associated Traditional Knowledge (SisGen) under registration number AB8AC9C.

The field setup consisted of two blocks (shaded area [A–F] and full sunlight area [G–L]) where each block consisted of six rows, with each row containing 10 *P. viridis* specimens. Additionally, edge rows were included but excluded from the experiment to prevent bias in the results (Santos, 2021). This experimental design resulted in 120 specimens in total: 60 individuals of the shaded area and 60 of the full sunlight area.

From each specimen, four leaves from different regions were harvested and stored in paper bags. Following collection, samples were dried in an oven at 40°C for 48 h, crushed using a mortar and pestle with liquid nitrogen. The crushed leaf samples of each row were grouped in 2, resulting in 12 samples from the shaded area and 12 from the full sunlight area. They were transferred to glass tubes and stored at −80°C until analysis.

Collection of the two blocks was performed across all four seasons of the year (summer, winter, spring, and autumn), with environmental conditions details presented in Figure 5 and Table S10. Notably, spring samples remained in paper bags longer than other samples; consequently, they were excluded from further analysis to maintain consistency. Additionally, samples from the matrix plant were collected in different seasons.

### Metabolome and lipidome extraction

The extraction process followed the three‐in‐one method developed by Hummel et al. (2011). In a 2‐ml tube, 50 mg of biological material macerated in liquid nitrogen was combined with 1 ml of the solvent mixture methanol (MeOH, LiChrosolv® Reag. Ph. Eur., Merck KGaA, Darmstadt, Germany) and methyl tert‐butyl ether (MTBE, 99.9% purity, Sigma‐Aldrich, St. Louis, MO, USA) in the MeOH:MTBE ratio (1:3 v/v) ice‐cold (−20°C). For extraction, the samples were incubated at 4°C for 10 min with shaking at 500 ***g*** in a microtube shaking incubator (AccuTherm, Labnet International Inc., Corning, NY, USA), followed by ultrasonic bath (5800, Branson Ultrasonics Corp., Danbury, CT, USA) in ice‐cold bath for 10 min. Subsequently, 500 μl of a MilliQ water: MeOH (3:1) mixture was added, and the samples were vortexed for 40 sec before being centrifuged for 10 min at 4°C and 10 000 ***g*** (Mikro 220R, Andreas Hettich GmbH & Corp., Tuttlingen, Germany). HPLC‐grade solvents were used, and the methanol was maintained at ice‐cold temperature. The resulting three phases (organic, aqueous, and protein pellet) were separated, dried in a vacuum concentrator (Concentrator Plus, Eppendorf AG, Hamburg, Germany) at room temperature, and stored at −80°C until chromatographic analysis. The organic (upper) phase was designated for lipidome analysis, while the aqueous (middle) phase was allocated for metabolome analysis.

### LC–MS acquisition

Chromatographic separation was performed using a Thermo Scientific UltiMate™ 3000 RSLC‐nano (Waltham, MA, USA) system equipped with a Titan C18 (100 × 2.1 mm, 1.9 μm) column (Sigma‐Aldrich). A gradient elution mode was employed with varying solvent proportions. Both organic and aqueous phases were resuspended in 1 ml solvent mixtures corresponding to the initial chromatographic run conditions: 60% mobile phase A and 40% mobile phase B, and subsequently filtered through a 0.22 μm PVDF filter.

For the organic phase samples, mobile phase A comprised 40% acetonitrile and 60% water, while mobile phase B consisted of 10% acetonitrile and 90% isopropanol, both containing 10 mmol L^−1^ ammonium acetate. Acetonitrile (Millipore, Billerica, MA, USA) was HPLC grade. Five microliters of each sample were injected into the column at a flow rate of 200 μl min^−1^ following the elution program: 0–2 min at 40% B, 2–3 min at 50% B, 3–6 min at 50% B, 6.1–8 min at 70% B, 8–9 min at 100% B, 9–11 min at 100% B, 11–12 min at 40% B, 12–14 min at 40% B.

For aqueous phase samples, mobile phase A was composed of water, and mobile phase B consisted of acetonitrile, both with 0.1% (v/v) formic acid. Five microliters of each sample were eluted at the same flow rate by using the following elution program: 0–2 min at 3% B; 3.7–5.6 min at 20% B; 7.4 min at 45% B; 9.3–14 min at 65% B; 16–20 min at 3% B.

The chromatography system was coupled to an Orbitrap QExactive mass spectrometer (Thermo Scientific, Waltham, MA, USA) featuring a heated‐electrospray ionization (HESI) source that operates in both ionization modes (positive and negative). The analysis was performed by full‐MS, followed by MS/MS analysis in data‐dependent acquisition (DDA) mode, targeting the five most intense peaks. Full‐MS data were acquired in the *m/z* range 100–1500 in profile mode, and at FT resolution 70 000 (at *m/z* 200). Automatic gain control was set at 1 × 10^6^, and the injection time was 100 ms. The sampler temperature was maintained at 10°C, with an over temperature setting of 40°C. The ESI voltage was set to +3.5 kV (positive mode) and −3.2 kV (negative mode). Ion optics settings included S‐Lens RF level of 50, S‐Lens of 25 V, skimmer at 15 V, and C‐Trap RF at 1010 V. The scaled normalized collision energy was set to 20–30–40 V.

Quality control (QC) samples, consisting of pooled samples, were included in LC–MS analysis aiming to evaluate the instrumental stability and data reliability. The chromatographic run began with injections of blank samples (mobile phase and extraction solvents), followed by the QC samples. QC samples were injected five times at the start of the batch and once after every 10 experimental samples. The injection order is detailed in Table S11 as recommended by Broadhurst et al. (2018).

LC–MS raw data are available on Metabolomics Workbench (Sud et al., 2016) under ID PR002007 (doi: 10.21228/M8624Z), with metabolomics data deposited under ID ST003219 and lipidomics data under ID ST003223.

### GC–MS acquisition

For GC–MS analyses, extractions from the mother plant were utilized. Microtubes containing samples from the separated and dried phases were thawed in a vacuum concentrator at 30°C for 30 min in aqueous‐vacuum mode to prevent water condensation on the inserts or microtubes.

The derivatization step followed the Fiehn method (Fiehn, 2016). Twenty milligrams of methoxyamine hydrochloride (MeOX) was weighed into a 2‐ml microtube, and 1 ml of anhydrous pyridine was added. The mixture was subjected to an ultrasound bath (40 kHz, Ecosonics Ultronique, model Q1.8/40, Indaiatuba, Brazil) for 2 min. Ten microliters of this solution was added into the insert containing the dry sample to initiate the methoximation reaction. Samples underwent three 2‐min cycles in the ultrasound bath, each followed by shaking (KASVI Multi Mixer, model K40‐10208, Pinhais, Brazil) at 2500 ***g***. The samples were then incubated for 1.5 h at 30°C in an oven (Tecnal, model TE‐394/1, Piracicaba, Brazil). For the silylation step, 91 μl of *N,O*‐bis(trimethylsilyl)trifluoroacetamide in 1% (v/v) trimethylchlorosilane was added, followed by shaking for 2 min at 2500 ***g*** and 2 min in an ultrasound bath. The reaction was completed with a 30‐min incubation at 30°C and the addition of 100 μl of heptane with stirring. Subsequently, samples were centrifuged (DLAB, model D1008, Beijing, China) for 30 sec, and the supernatant was transferred to new vials containing a 250 μl insert for GC–MS analysis.

One microliter of the derivatized sample was injected in the chromatography system using the Fiehn method (Kind et al., 2009). Derivatized samples from the organic phase were injected at an injector temperature of 250°C in pulsed split mode (initially at 60 psi for 0.75 min with a 10:1 ratio), while those from the aqueous phase were injected in the split mode with a 400:1 ratio. A gas chromatography instrument (7890A, Agilent Technologies, Santa Clara, CA, USA) equipped with a 7693 autosampler (Agilent) was used. Chromatographic separation was performed on a non‐polar capillary column with (5%‐phenyl)‐methylpolysiloxane stationary phase (30 m, 250 μm, 0.25 μm internal diameter, HP5‐MS, Agilent Technologies, Santa Clara, CA, USA) using helium as the carrier gas at a flow rate of 1 ml min^−1^. The temperature program was as follows: 60°C for 1 min, then the temperature was increased to 300°C at a heating rate of 10°C min^−1^ and maintained for 10 min. The total chromatographic run was 35 min.

The gas chromatography equipment was coupled to an Agilent 5975C inert XL Mass Selective Detector Triple‐Axis quadrupole mass spectrometer with an electron ionization source set to 70 eV. The ion source temperature was maintained at 280°C, and the detector temperature at 150°C. Spectra were acquired in full‐MS mode within the mass range of 50–600 Da at a rate of 2.66 scans sec^−1^. A solvent cut of 7 min was applied to exclude solvent peaks.

### Data processing and metabolite identification

LC–MS raw data were converted to .mzML format using MSConvert (version 3.0) from Proteowizard. The converted data were processed using the MS‐DIAL software version 4.9 (Tsugawa et al., 2015) for feature detection, spectral deconvolution, alignment, and compound annotation employing the previously optimized parameters, except that retention time end was 20 min (Matos et al., 2023). Identification was performed using the Fiehn/Vaniya Natural Product Library .MSP (#ID 20200109) comprising 28 347 compounds in positive mode and 11 590 compounds in negative mode, based on spectral similarity with a cosine value greater than 0.8 (https://systemsomicslab.github.io/compms/msdial/main.html#MSP). Compound classes were verified using *PubChem* (https://pubchem.ncbi.nlm.nih.gov/) and the Chemical Entities of Biological Interest ontology (Degtyarenko et al., 2007). MS‐DIAL lipidomics identification was based on the LipidBlast internal library (Kind et al., 2013). Potential adducts considered included [M + H]^+^, [M + Na]^+^, [M + K]^+^, [M + NH_4_]^+^, [M + CH_3_OH + H]^+^, [M + ACN + H]^+^, and [M + H − H_2_O]^+^ for positively charged adducts, and [M − H]^−^, [M + Cl]^−^, [M − H_2_O − H]^−^, [M + Na − 2H]^−^, [M + K − 2H]^−^, and [M + FA − H]^−^ for negatively charged adducts. The organic phase data from positive ionization mode was further analyzed and more details are in the Supporting Information S1.

GC–MS .CDF data were uploaded to the GNPS platform (Wang et al., 2016) using the WinSCP file manager with a .txt archive containing three columns: filename, ATTRIBUTE_Sampletype (Sample or Standard), and ATTRIBUTE_group (Fall, Summer, Winter, or Standard). On GNPS, data were auto‐deconvoluted using the machine learning approach MSHub (Aksenov et al., 2021) with parameters based on developer instructions (https://ccms‐ucsd.github.io/GNPSDocumentation/gc‐ms‐deconvolution/). Adjustments included a fragment ion mass tolerance of 0.5 Da, a minimum of six matched peaks, and a score threshold of 0.5. Advanced network options were set to a minimum pair cosine of 0.6 and a network topK of 10. Identification was performed using GNPS spectral libraries. Detailed processing and molecular network construction for GC–MS data are publicly accessible at https://gnps.ucsd.edu/ProteoSAFe/status.jsp?task=ce05be0628174734b20003b55d6403c2, and molecular networking was visualized using Cytoscape 3.10.1 (Shannon et al., 2003).

### Data visualization

LC–MS raw data matrices were uploaded to MetaboAnalyst (Chong et al., 2019) for data pretreatment and statistical analyses. The two extracted phases (organic and aqueous) analyzed in both two ionization modes (positive and negative) resulted in four raw data matrices. MetaboAnalyst developers encouraged exploration of various methods to determine if the results can be enhanced. Each matrix underwent an optimized pretreatment step: for positive ionization mode organic phase data, quantile normalization was first applied, followed by a 30% relative standard deviation (RSD) filter based on QC samples, log transformation, and Pareto scaling representing a reduction in the intensity table of 17 149 features to 5000 features. Negative ionization mode organic phase data were subjected to median normalization, a 30% RSD filter, square root transformation, and range scaling representing a reduction in the intensity table of 14 607 features to 3286 features. For positive ionization mode aqueous phase data, a 30% RSD filter, probabilistic quotient normalization, square root transformation, and autoscaling were applied, resulting in a reduction of 17 166 features to 2154 on the intensity table. The negative ionization mode aqueous phase data were treated similarly, except that the cube root transformation was used instead of square root transformation, resulting in a reduction of 22 025 features to 3414 on the intensity table.

Processed LC–MS data were evaluated using unsupervised exploratory analyses, PCA and HCA with Euclidean distance measure and Ward clustering algorithm. Supervised analysis was conducted using partial least squares discriminant analysis (PLS‐DA) with 5‐fold cross‐validation to assess model robustness and VIP scores higher than 1 (Tables S12 and S13). All figures of merit obtained for the PLS‐DA model were higher than 0.8 (Figure S2).

For biochemical pathway visualization, MetaboAnalyst Functional Analysis was performed using the Mummichog algorithm (Li et al., 2013). This tool identifies altered metabolic pathways by comparing two groups based on the MS^1^ spectra of the compounds. A .txt file containing four columns—m.z, mode (positive or negative), *P*.value, and r.t (retention time in minutes)—was submitted. The comparison for *P*‐value calculation was based on the result from the exploratory analysis: FS (Fall and Summer together) versus Winter, and Shade versus Full sunlight area. Compound annotation employed a mass precision of 5 p.p.m., retention time in minutes, generic data source format, mixed ionization mode, and no normalization step, as raw data tables were not utilized. Among the KEGG pathway libraries available on the platform, the *Arabidopsis thaliana* library was selected for being a model plant with one of the most comprehensive sets of biochemical information available in the literature. Moreover, a default *P*‐value cutoff and version 2.0 were applied.

## AUTHOR CONTRIBUTIONS

TSM performed metabolomics and lipidomics experiments, and CDLDS carried out the agronomic work. TSM performed data analysis and prepared the original draft. CDLDS, IMJ, MCB, LFT, and AS edited and revised the manuscript. IMJ and MCB supervised the agronomic work. AS and LFT conceptualized the study. AS designed the experiments, provided resources, acquired financial support, and supervised the research.

## CONFLICT OF INTEREST

The authors have not declared a conflict of interest.

## Supporting information


**Figure S1.** PLS‐DA score plots for distinguishing the groups. (a) Seasons: aqueous phase, negative ionization mode data. (b) Seasons: aqueous phase, positive ionization mode data. (c) Seasons: organic phase, negative ionization mode data. (d) Seasons: organic phase, positive ionization mode data. (e) Cultivation mode: aqueous phase, negative ionization mode data. (f) Cultivation mode: aqueous phase, positive ionization mode data.
**Figure S2.** Selection of significant variables by VIP scores and cross‐validation of discrimination models of the organic phase (Lipidomics) and aqueous phase (Metabolomics) samples in both ionization modes (Negative—N and Positive—P). The asterisk highlights the highest *Q* ^2^ value among the models.
**Figure S3.** Molecular network generated after analyzing positive ionization mode data from the organic phase using GNPS. The following parameters were set: mass tolerance of precursor and fragments ions defined as 0.02 Da, and a cosine score above 0.7. Colors represent the identified metabolite classes, as indicated in the caption frame. Gray nodes represent metabolites that were neither annotated nor had suggested biochemical pathways. For more details about the network on GNPS, visit: https://gnps2.org/status?task=dbe73b8583e046b687a37e815e8dd6df. Biochemical pathways were defined higher than 70% probability by SIRIUS/CANOPUS.
**Figure S4.** Molecular network generated after analysis via GNPS. Colors represent seasons or growth environmental conditions of cultivation (full sunlight and shaded area).
**Figure S5.** Cluster analysis presenting trends across seasons and cultivation conditions (full sunlight and shaded area). Three major regions are observed based on biochemical pathways and seasonal color patterns: Fall exhibits the highest diversity of biochemical classes, Summer shows a higher abundance of fatty acids, and Winter highlights terpenoids. When considering biochemical pathways and cultivation condition color patterns, two distinct regions emerge: plants grown in full sunlight display greater biochemical class diversity, while those cultivated in shaded areas are enriched in fatty acids and terpenoids.
**Figure S6.** Clusters with DMT considering seasons or cultivation conditions (full sunlight and shaded area). The alkaloids in these clusters exhibit similar abundance across the experimental groups.
**Table S1.** Annotated compounds based on MS/MS in aqueous phase negative ionization.
**Table S2.** Annotated compounds based on MS/MS in aqueous phase positive ionization mode data.
**Table S3.** Annotated compounds based on MS/MS in organic phase negative ionization mode data.
**Table S4.** Annotated compounds based on MS/MS in organic phase positive ionization mode data.
**Table S5.** Annotated compounds by GNPS on the samples analyzed by LC–MS.
**Table S6.** Annotated compounds by GNPS on the samples analyzed by GC–MS.
**Table S7.** Number of compounds by class in each sample analyzed by GC–MS.
**Table S8.** Mummichog pathway analysis result table for seasons differentiation.
**Table S9.** Mummichog pathway analysis result table for cultivation differentiation.
**Table S10.** Environmental conditions across 2019 in Paulínia, SP, Brazil.
**Table S11.** Injection order applied for the metabolomics and lipidomics assays in both ionization modes.
**Table S12.** Features of aqueous phase with their respective VIP (PLS‐DA). VIP > 1.60 based on positive ionization mode.
**Table S13.** Features of organic phase with their respective VIP (PLS‐DA) based on seasons. VIP > 1.60 based on negative ionization mode.

## Data Availability

The data that support the findings of this study are openly available in Metabolomics Workbench at https://www.metabolomicsworkbench.org/, reference number PR002007 (doi:10.21228/M8624Z).
