# Supplementary material for: Influence of environmental conditions and seasonality on the metabolome and lipidome of Psychotria viridis leaves
Source: Plant J. 2025 Jul 22;123(2):e70353. doi: 10.1111/tpj.70353 (PMC12283096; doi:10.1111/tpj.70353)
Supplement: Supplementary file 1 — Figure S1. PLS‐DA score plots for distinguishing the groups. (a) Seasons: aqueous phase, negative ionization mode data. (b) Seasons: aqueous phase, positive ionization mode data. (c) Seasons: organic phase, negative ionization mode data. (d) Seasons: organic phase, positive ionization mode data. (e) Cultivation mode: aqueous phase, negative ionization mode data. (f) Cultivation mode: aqueous phase, positive ionization mode data. Figure S2. Selection of significant variables by VIP scores and cross‐validation of discrimination models of the organic phase (Lipidomics) and aqueous phase (Metabolomics) samples in both ionization modes (Negative—N and Positive—P). The asterisk highlights the highest Q 2 value among the models. Figure S3. Molecular network generated after analyzing positive ionization mode data from the organic phase using GNPS. The following parameters were set: mass tolerance of precursor and fragments ions defined as 0.02 Da, and a cosine score above 0.7. Colors represent the identified metabolite classes, as indicated in the caption frame. Gray nodes represent metabolites that were neither annotated nor had suggested biochemical pathways. For more details about the network on GNPS, visit: https://gnps2.org/status?task=dbe73b8583e046b687a37e815e8dd6df. Biochemical pathways were defined higher than 70% probability by SIRIUS/CANOPUS. Figure S4. Molecular network generated after analysis via GNPS. Colors represent seasons or growth environmental conditions of cultivation (full sunlight and shaded area). Figure S5. Cluster analysis presenting trends across seasons and cultivation conditions (full sunlight and shaded area). Three major regions are observed based on biochemical pathways and seasonal color patterns: Fall exhibits the highest diversity of biochemical classes, Summer shows a higher abundance of fatty acids, and Winter highlights terpenoids. When considering biochemical pathways and cultivation condition color patterns, two distinct regions emer [file TPJ-123-0-s001.docx]

**SUPPORTING INFORMATION**

**Influence of environmental conditions and seasonality on the metabolome and lipidome of *Psychotria viridis* leaves**

Taynara Simão Matos^1^, Camila Dias Lourenço Dos Santos^2,3^, Luís Fernando Tófoli^2^, Ílio Montanari Júnior^4^, Márcia Cristina Breitkreitz^3,5^, Alessandra Sussulini^1,5^

*^1^ Laboratory of Bioanalytics and Integrated Omics (LaBIOmics), Institute of Chemistry, Universidade Estadual de Campinas (UNICAMP), Campinas, São Paulo, Brazil*

*^2^ Interdisciplinary Cooperation for Ayahuasca Research and Outreach (ICARO), School of Medical Sciences, Universidade Estadual de Campinas (UNICAMP), Campinas, São Paulo, Brazil*

*^3^ Faculty of Pharmaceutical Sciences, Universidade Estadual de Campinas (UNICAMP), Campinas, São Paulo, Brazil*

*^4^ Multidisciplinary Center for Chemical, Biological and Agricultural Research (CPQBA), Universidade Estadual de Campinas (UNICAMP), Campinas, São Paulo, Brazil*

*^5^ National Institute of Science and Technology of Bioanalytics (INCTBio), Institute of Chemistry, Universidade Estadual de Campinas (UNICAMP), Campinas, São Paulo, Brazil*

*For correspondence: sussulini@unicamp.br


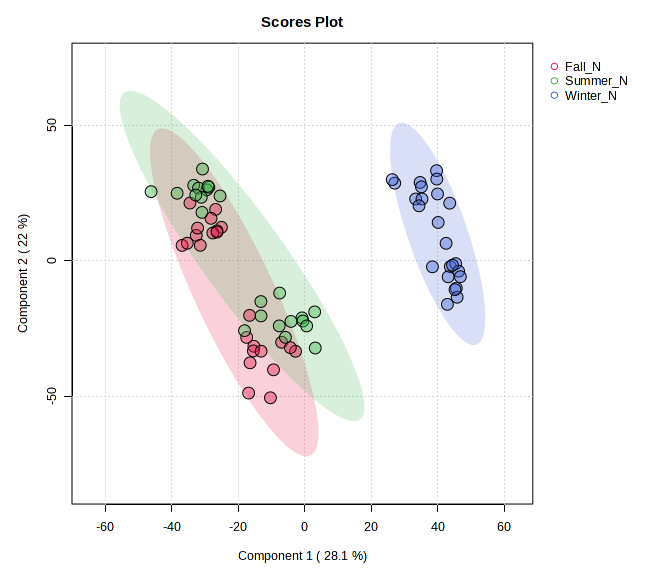

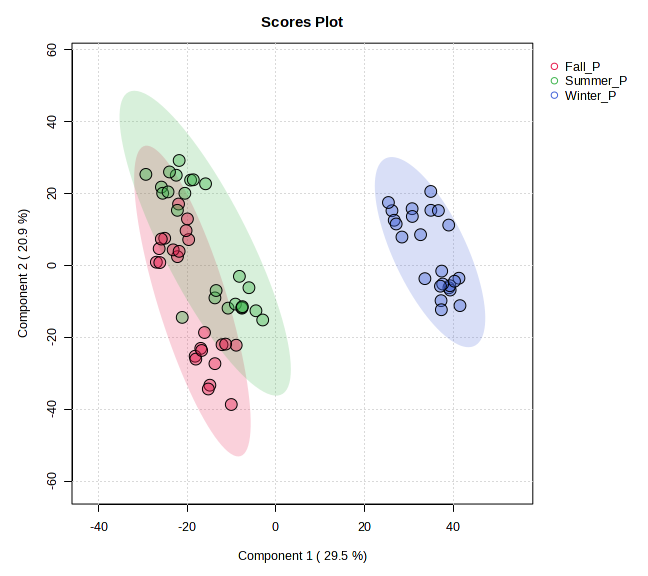


**A**


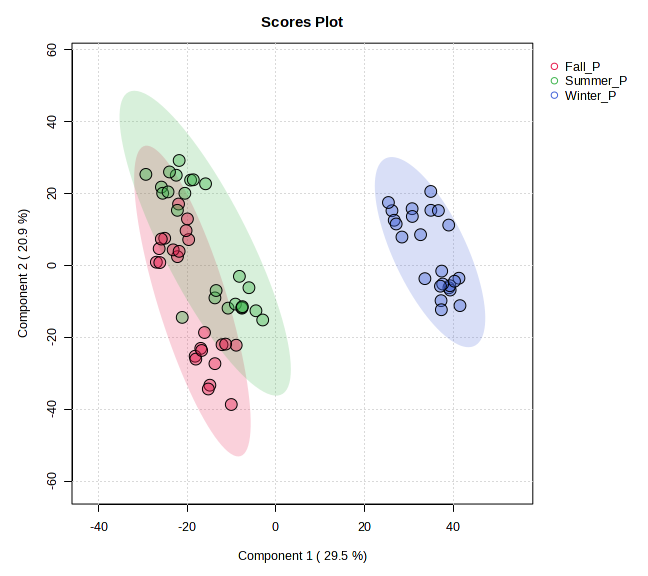

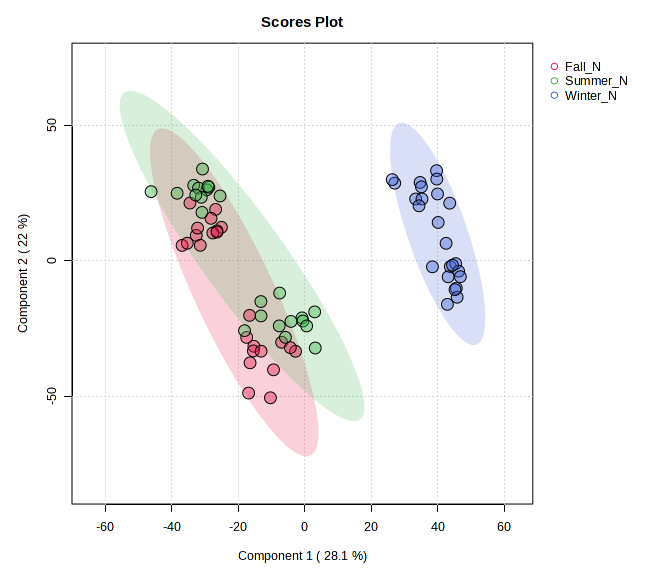


**B**


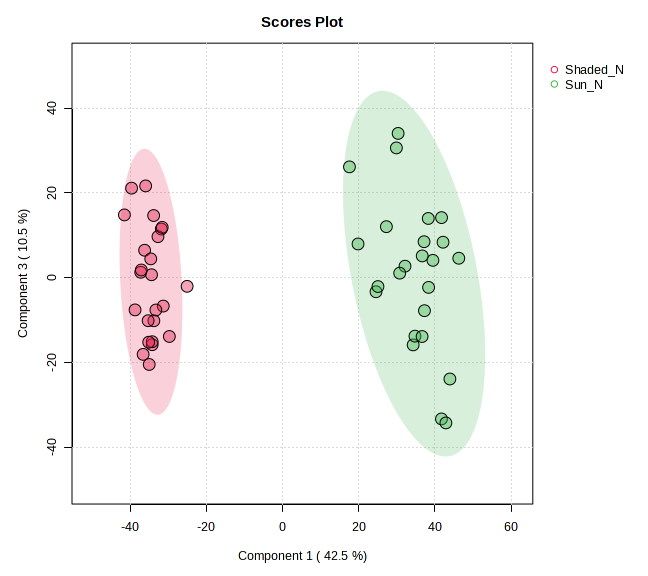

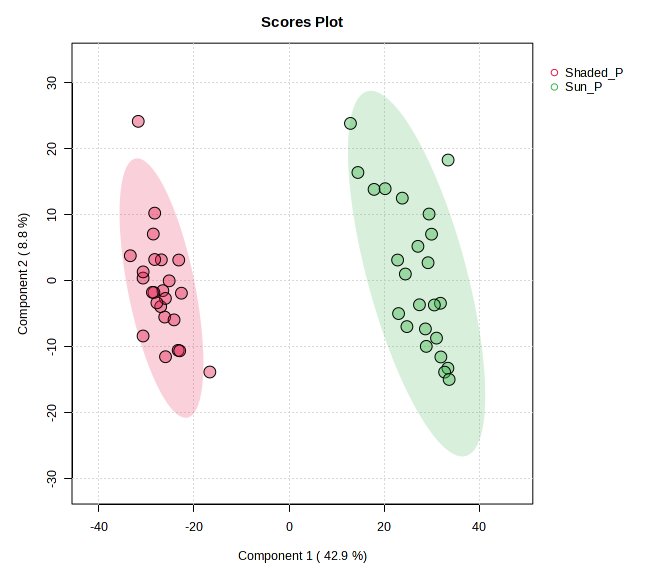


**E**

**F**


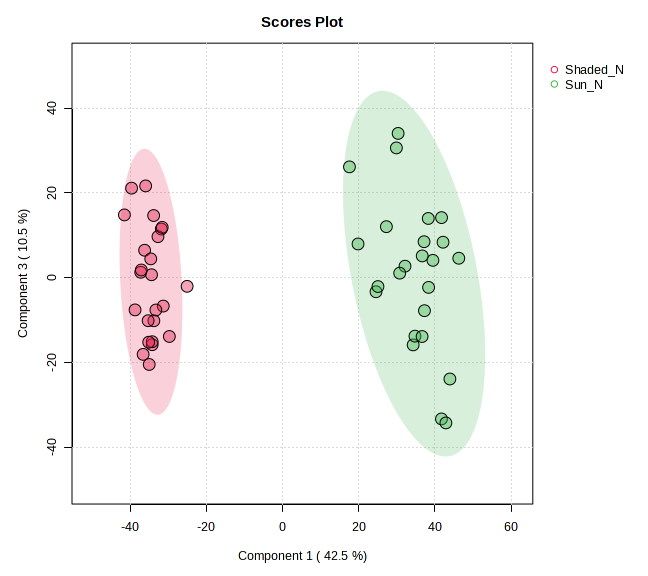

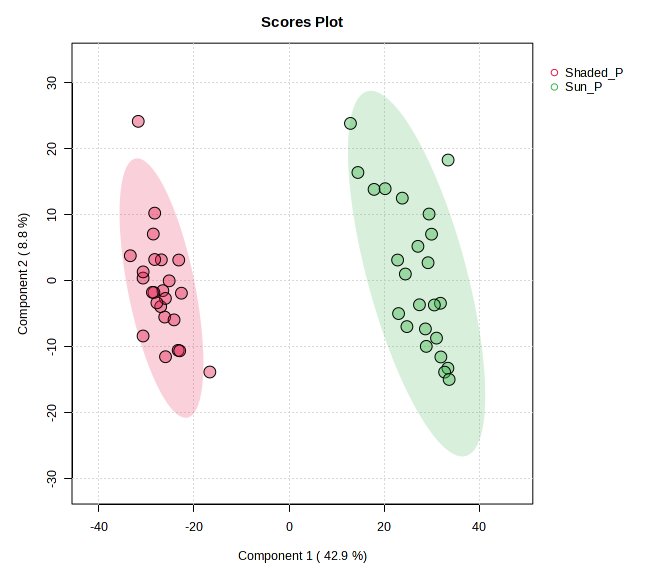

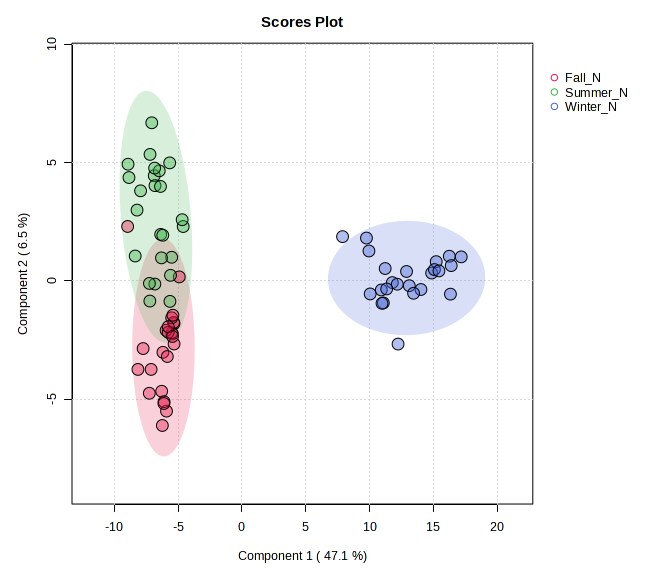

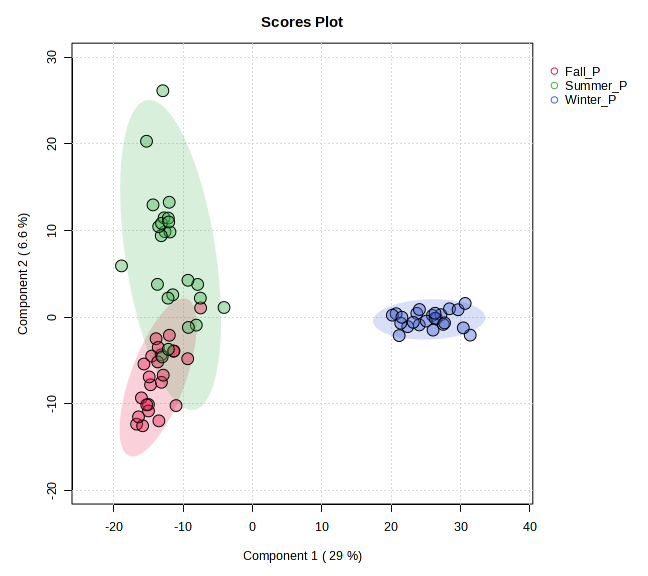


**C**

**D**


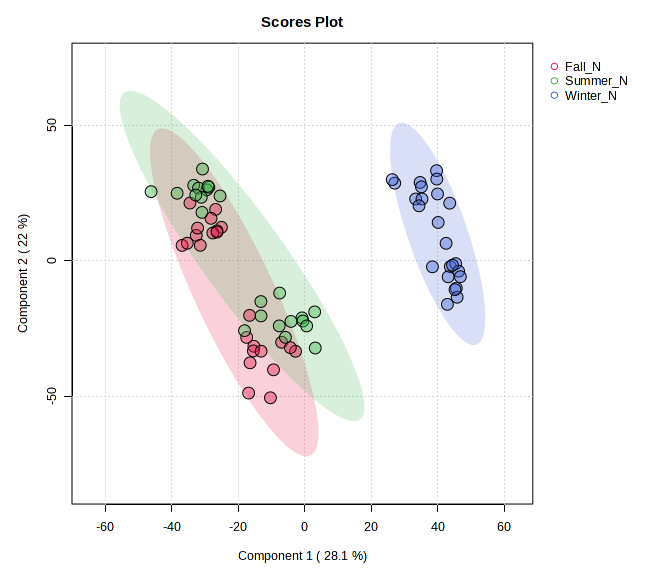

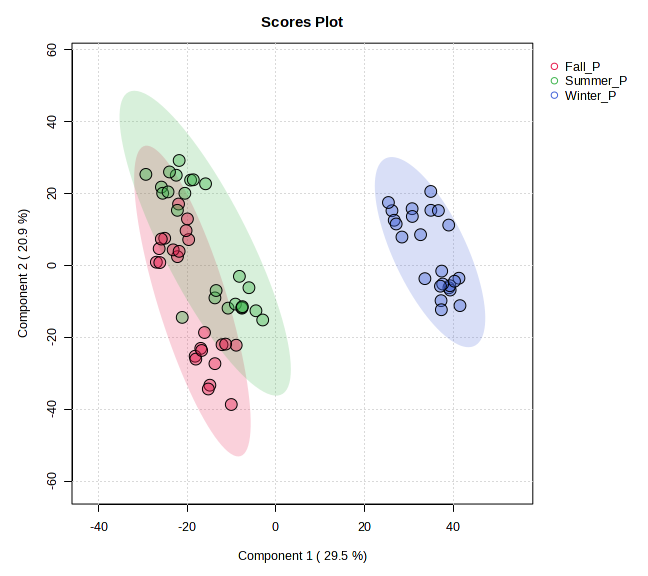


**Figure S1.** PLS-DA score plots for distinguishing the groups. (a) Seasons: aqueous phase, negative ionization mode data. (b) Seasons: aqueous phase, positive ionization mode data. (c) Seasons: organic phase, negative ionization mode data. (d) Seasons: organic phase, positive ionization mode data. (e) Cultivation mode: aqueous phase, negative ionization mode data. (f) Cultivation mode: aqueous phase, positive ionization mode data.

**Figure S2.** Selection of significant variables by VIP scores and cross-validation of discrimination models of the organic phase (Lipidomics) and aqueous phase (Metabolomics) samples in both ionization modes (Negative – N and Positive – P). The asterisk highlights the highest Q^2^ value among the models.


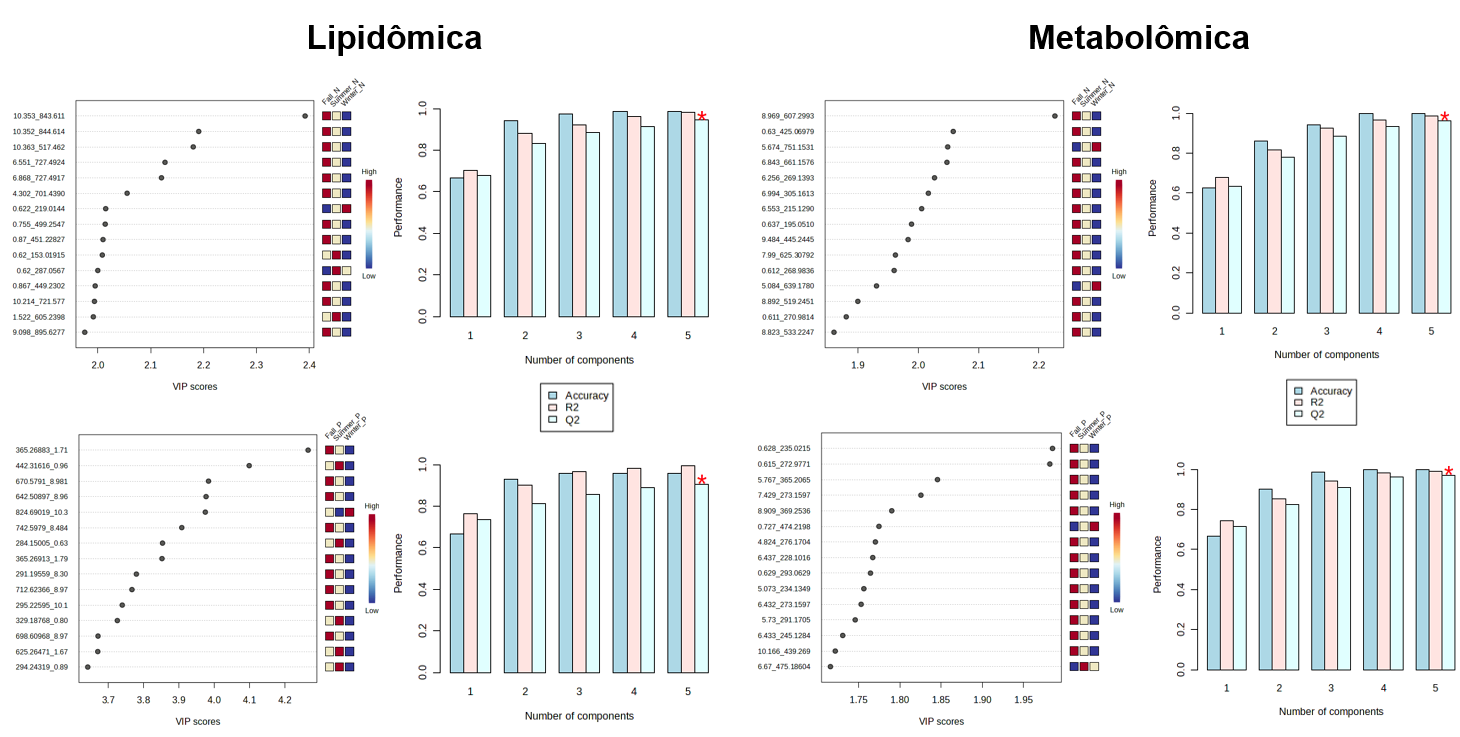


**Lipidomics**

**Metabolomics**

**Figure S3.** Molecular network generated after analyzing positive ionization mode data from the organic phase using GNPS. The following parameters were set: mass tolerance of precursor and fragments ions defined as 0.02 Da, and a cosine score above 0.7. Colors represent the identified metabolite classes, as indicated in the caption frame. Gray nodes represent metabolites that were neither annotated nor had suggested biochemical pathways. For more details about the network on GNPS, visit: https://gnps2.org/status?task=dbe73b8583e046b687a37e815e8dd6df


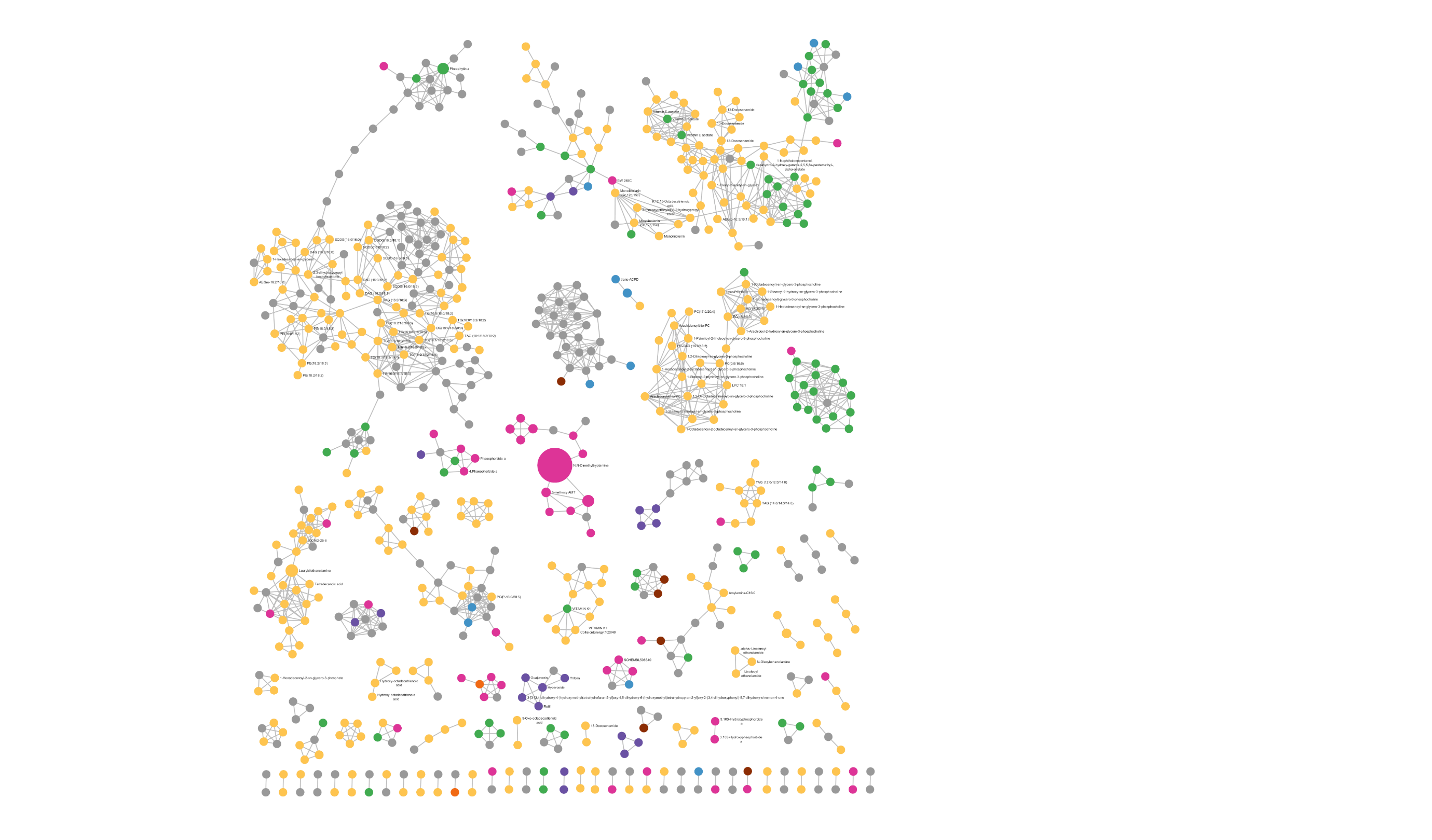

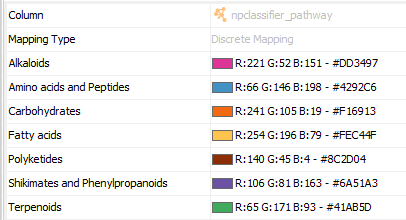

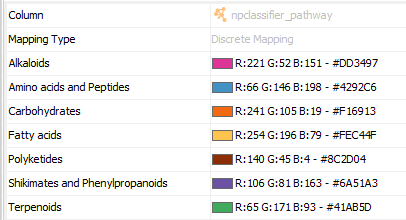

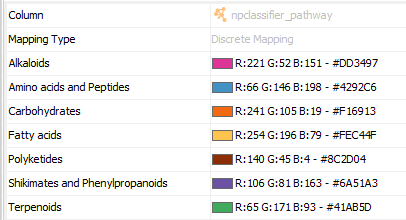


Biochemical pathways were defined higher than 70% probability by SIRIUS/CANOPUS^1,2^.

^1^Yannick *et al*. ClassyFire: automated chemical classification with a comprehensive, computable taxonomy. J Cheminf, 8, 2016. https://doi.org/10.1186/s13321-016-0174-y

^2^Kim *et al*. NPClassifier: A Deep Neural Network-Based Structural Classification Tool for Natural Products. J Nat Prod, 84, 2021. https://doi.org/10.1021/acs.jnatprod.1c00399

**Figure S4.** Molecular network generated after analysis via GNPS. Colors represent seasons or growth environmental conditions of cultivation (full sunlight and shaded area).


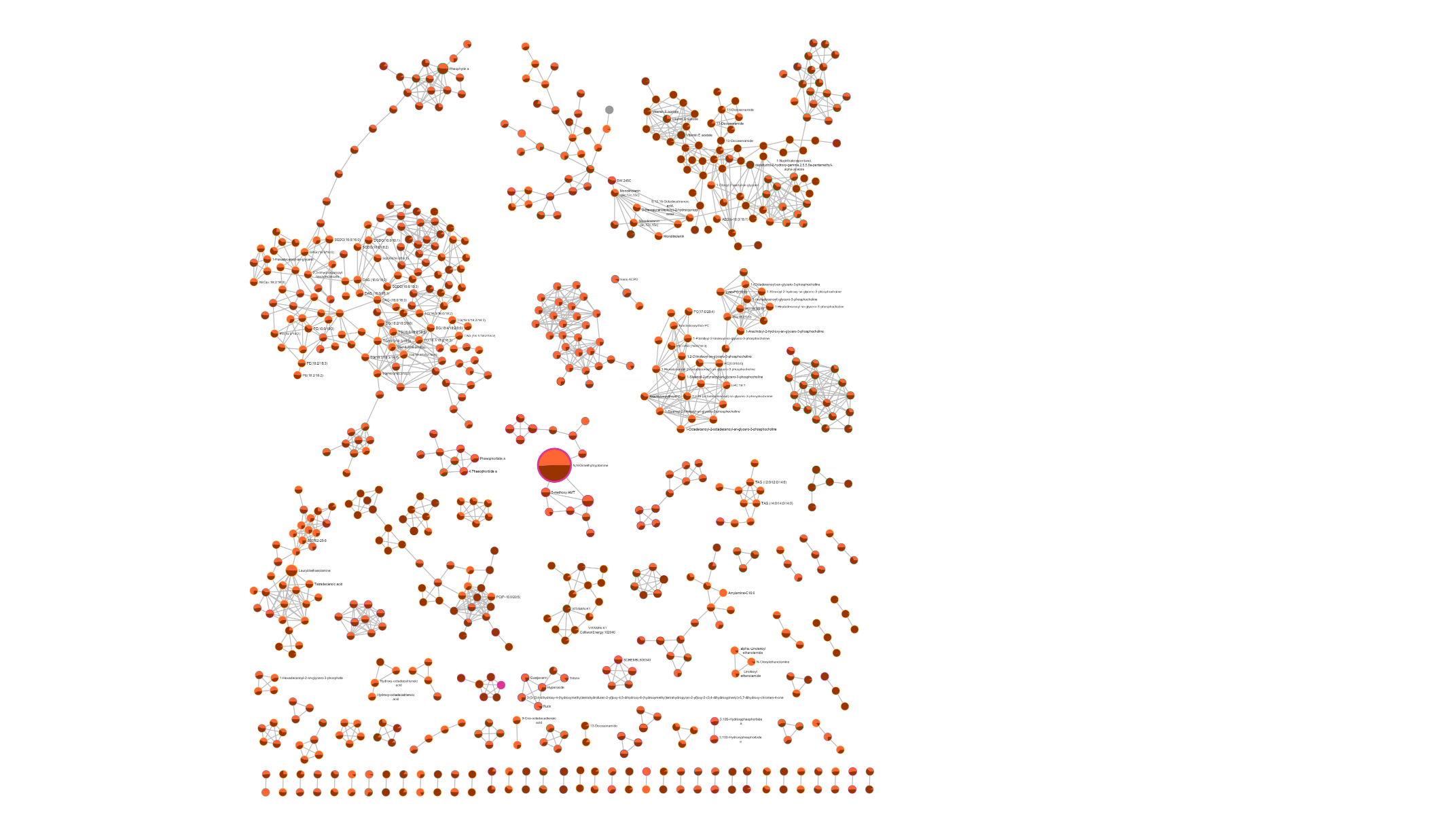

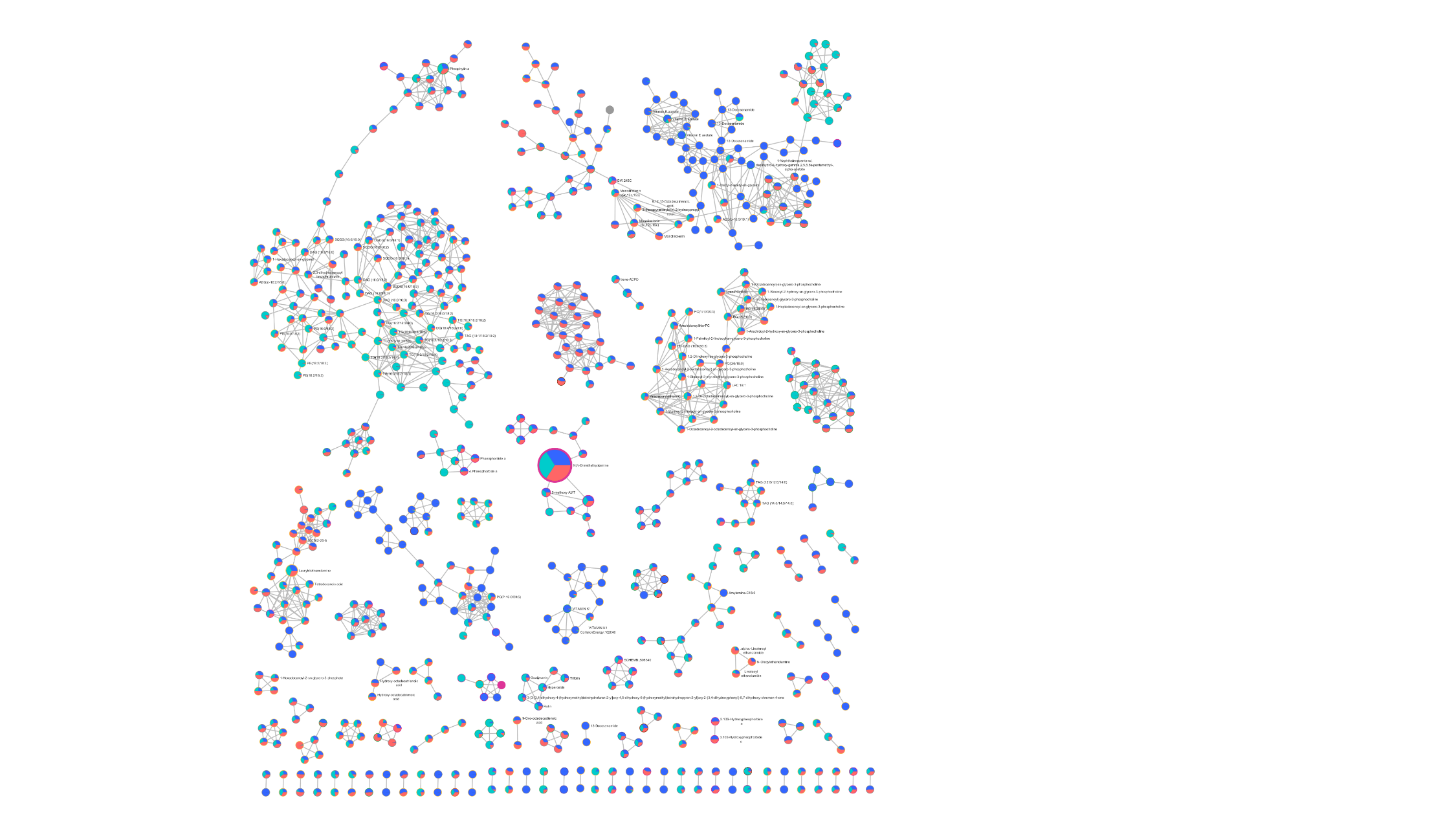

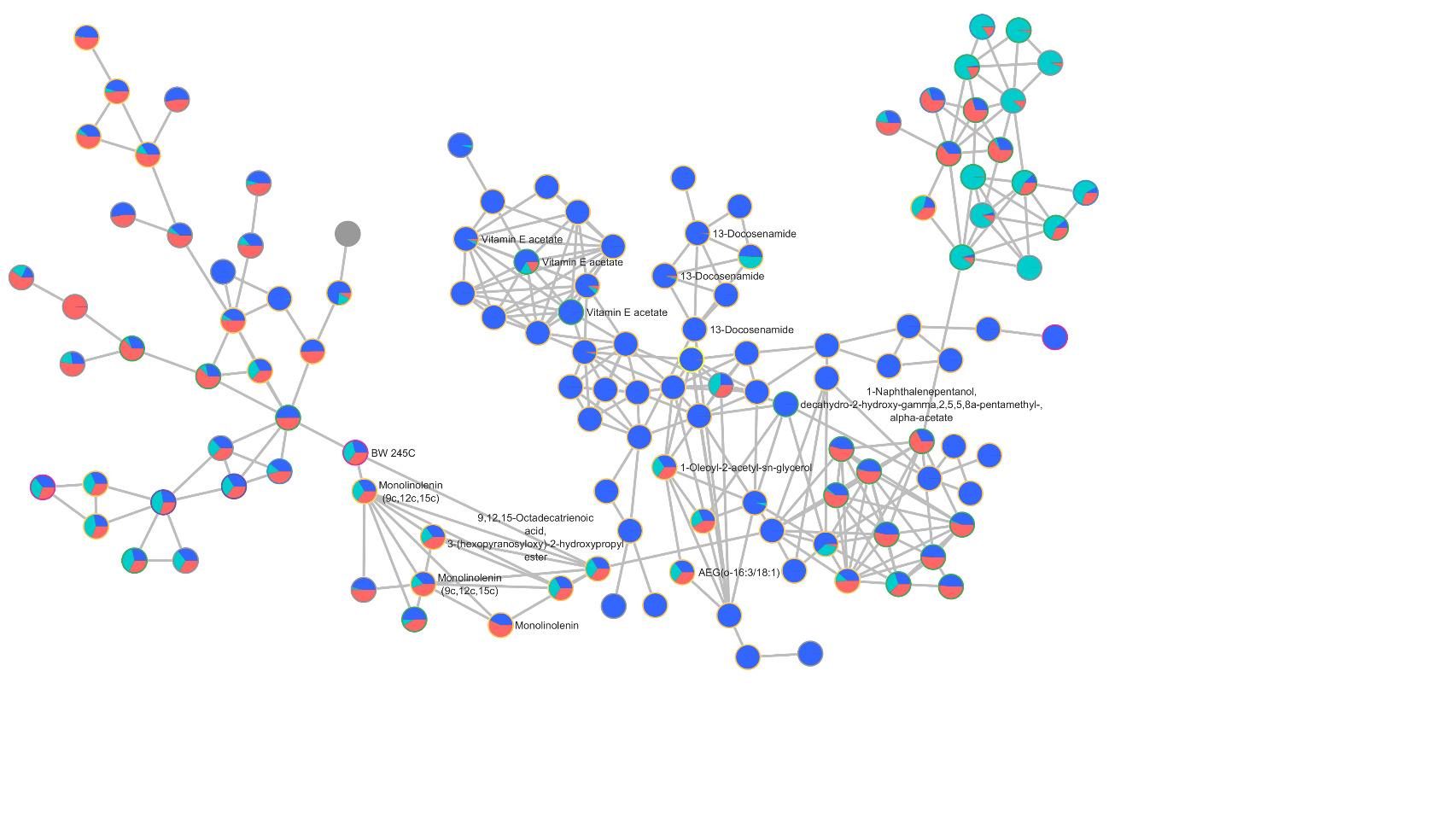

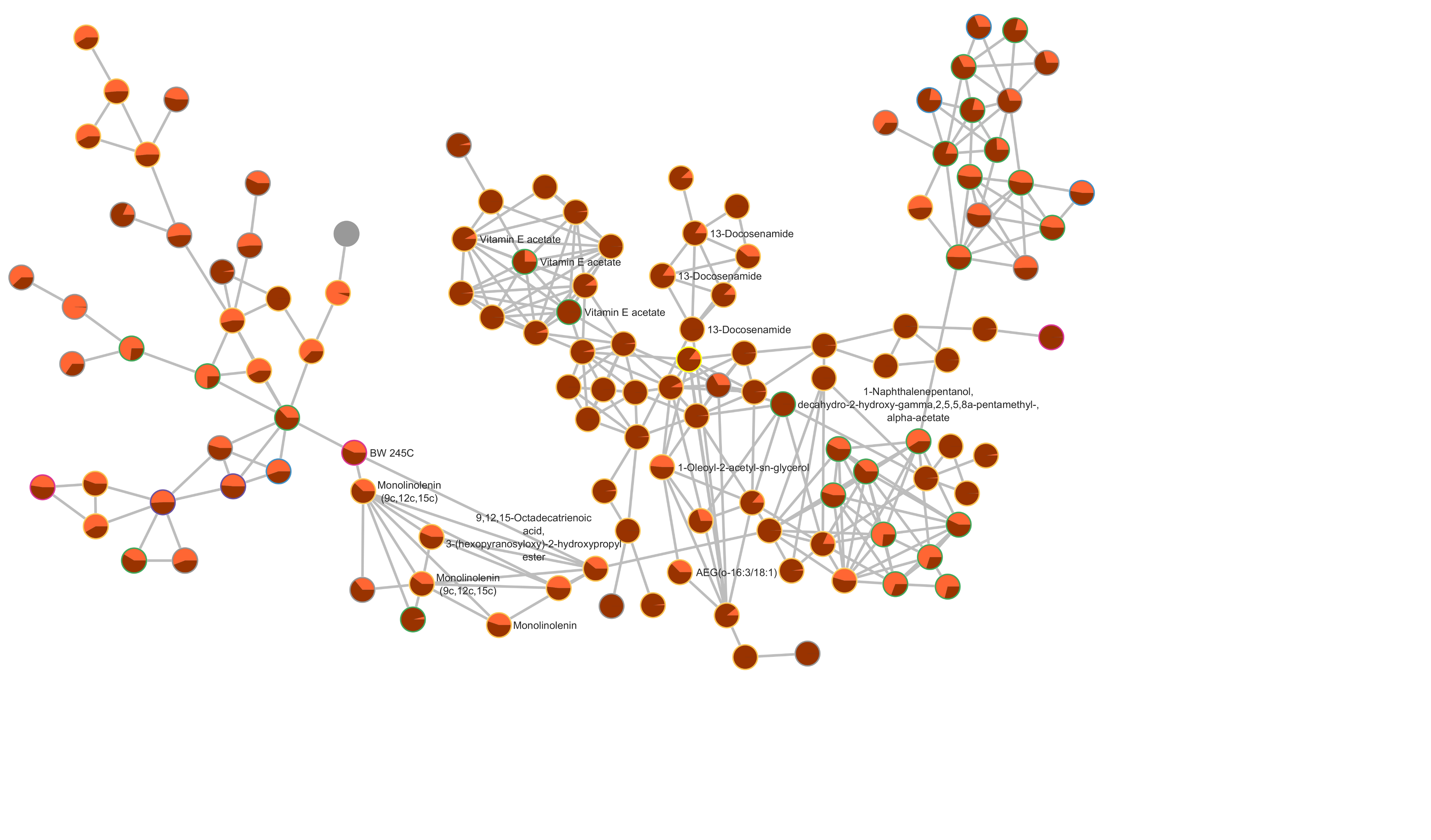

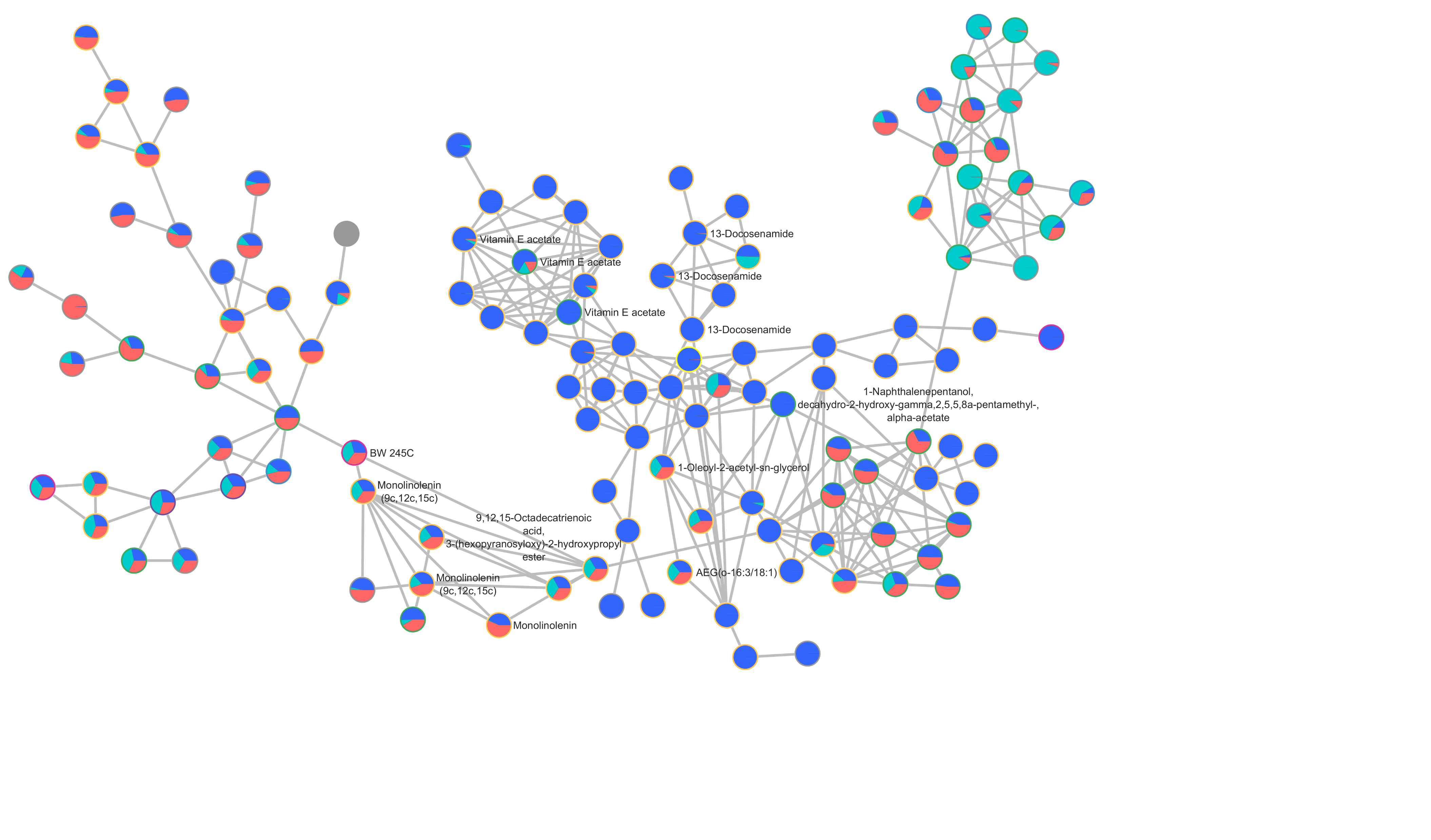

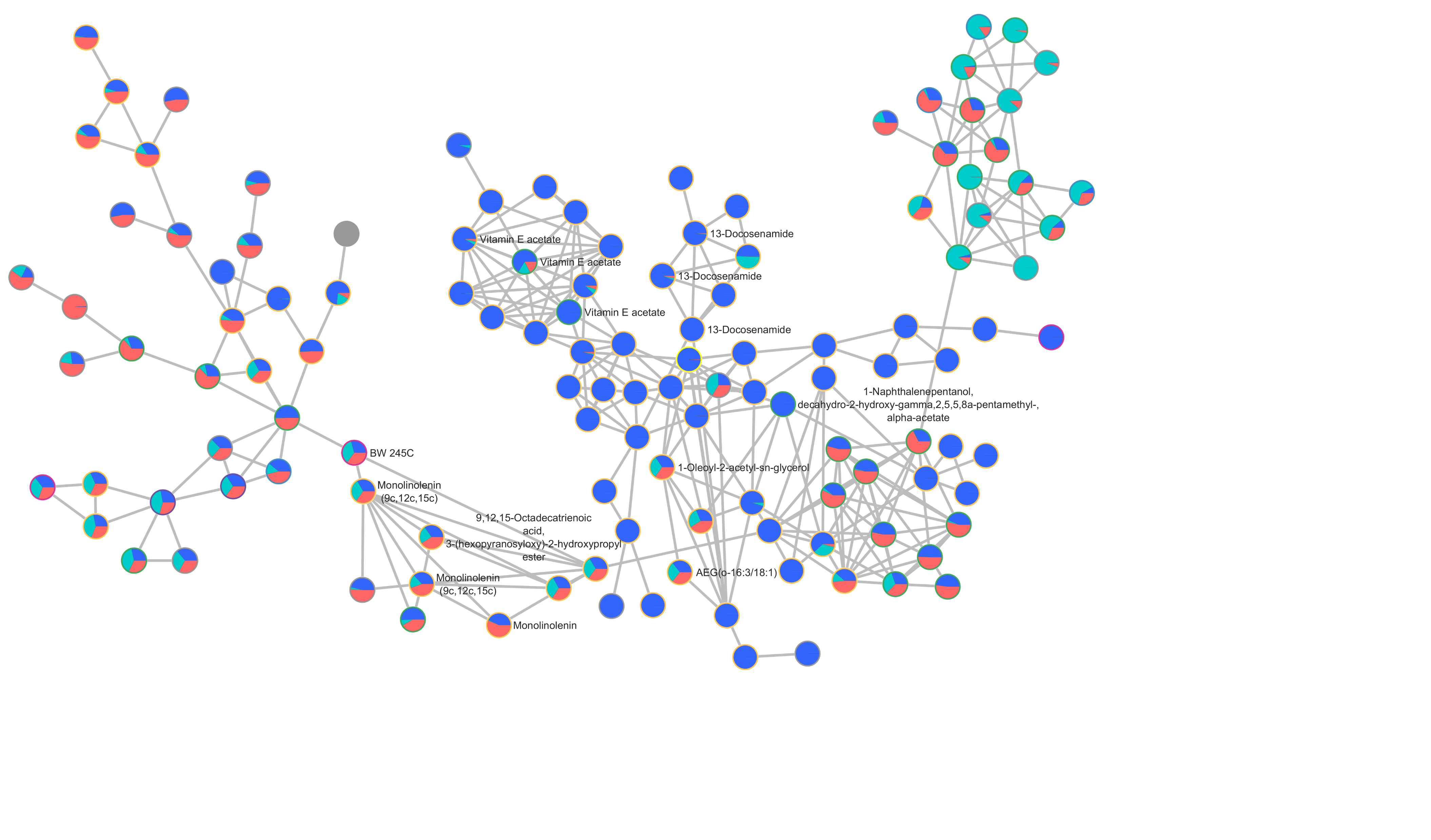

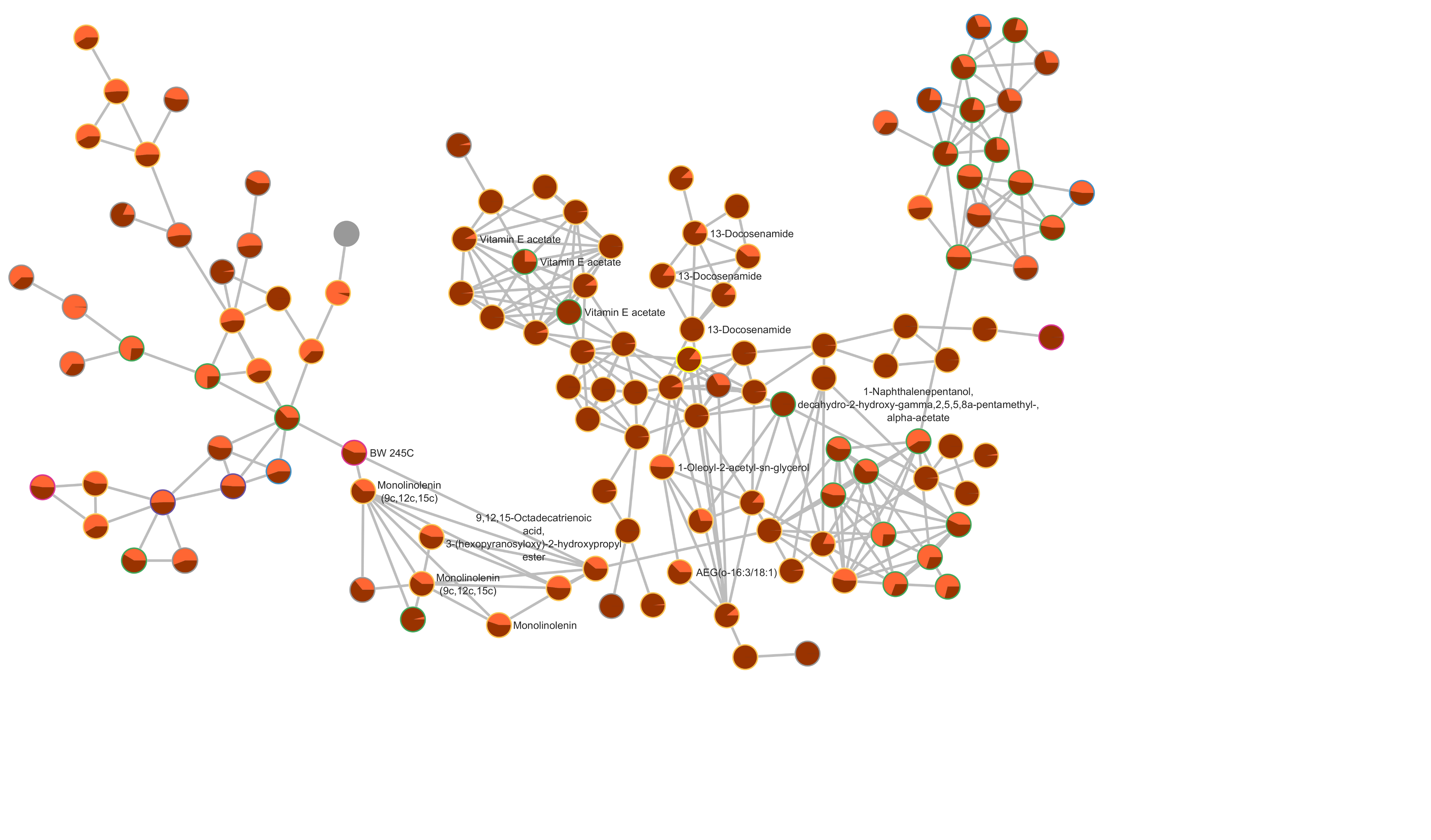


**Summer**

**Fall**

**Winter**

**Sunlight**

**Shaded**


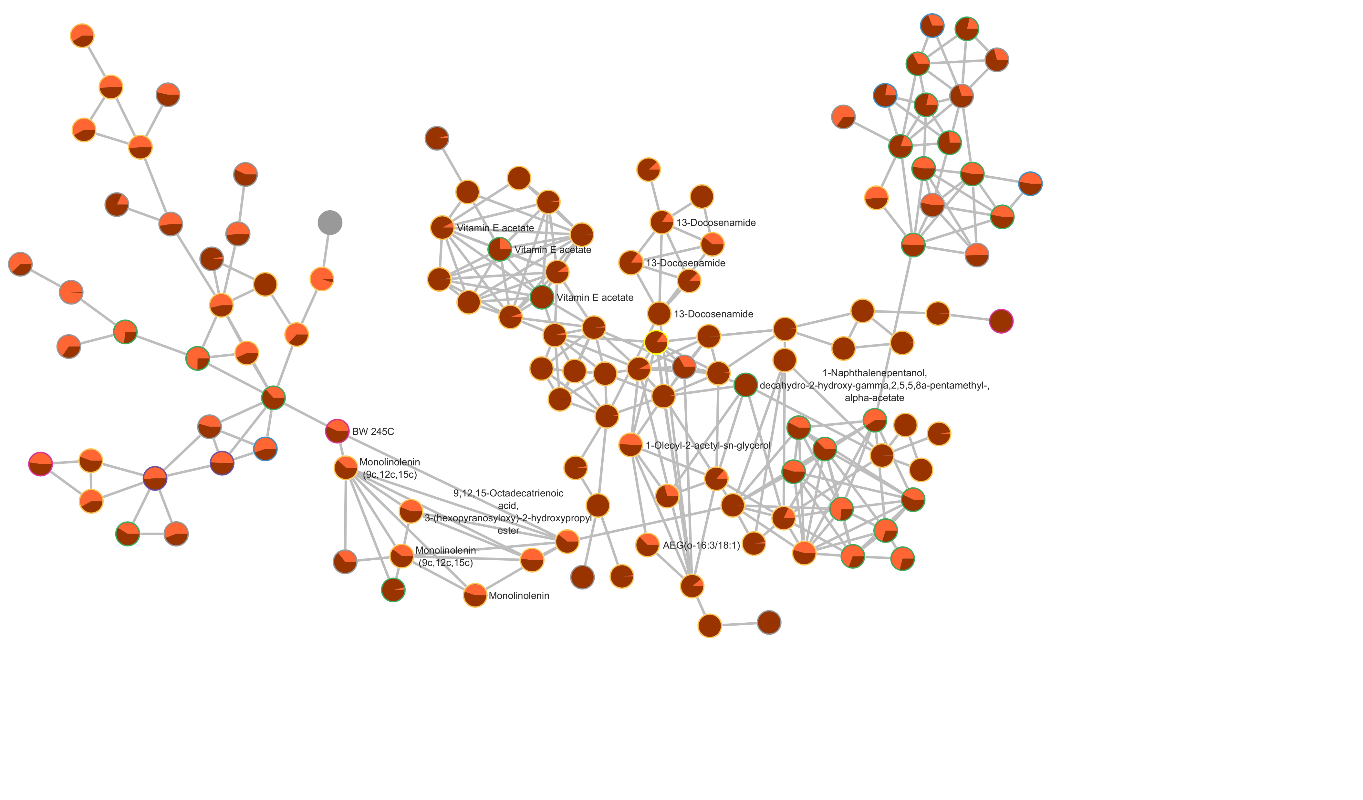

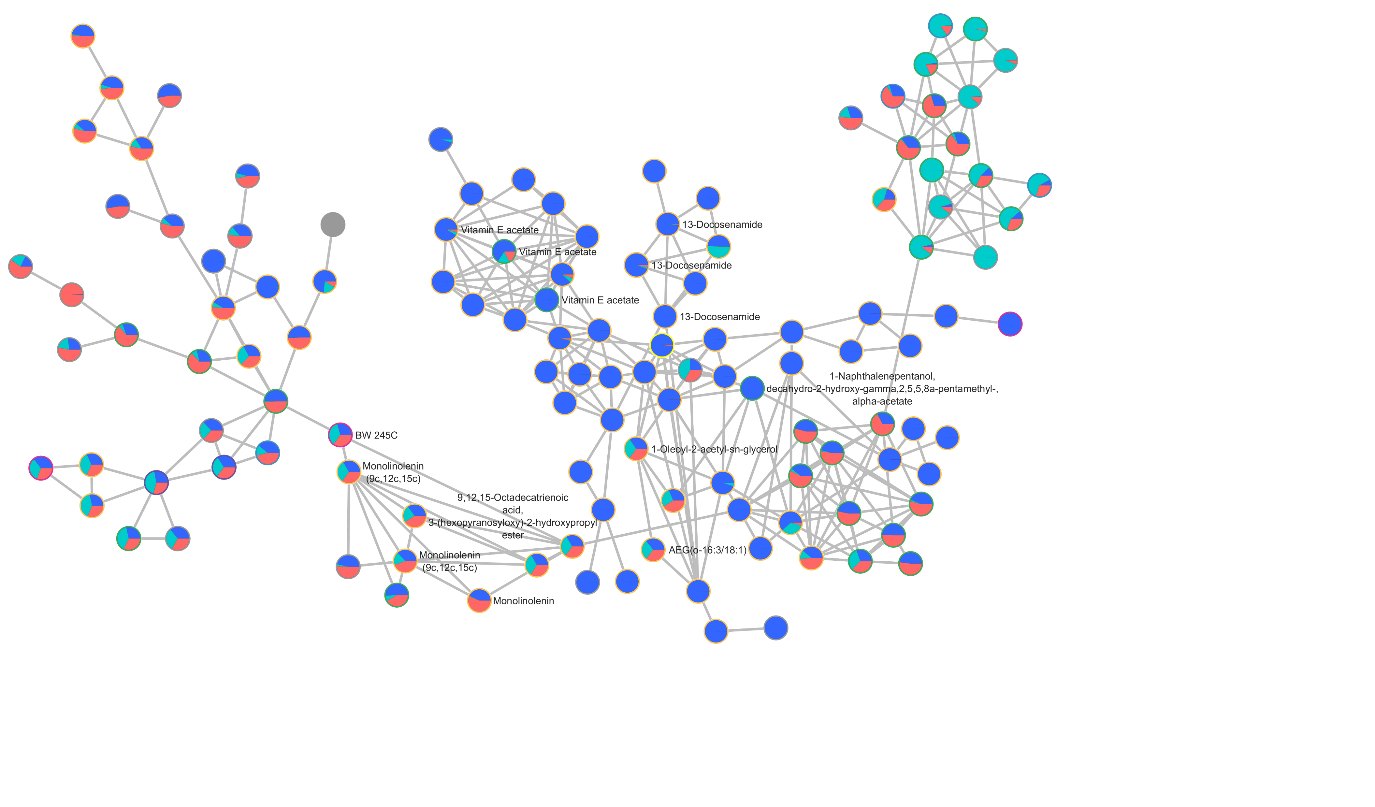

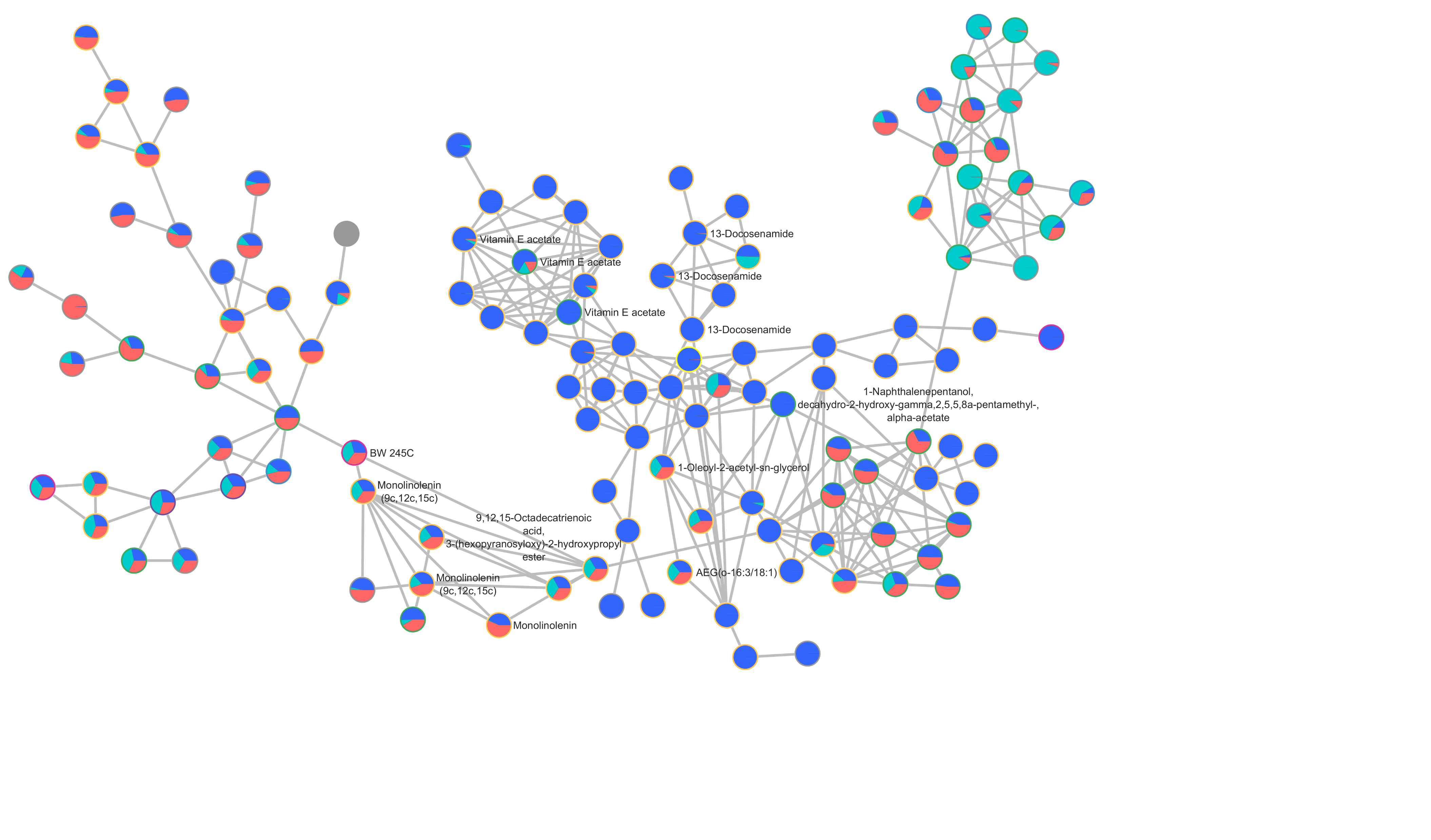

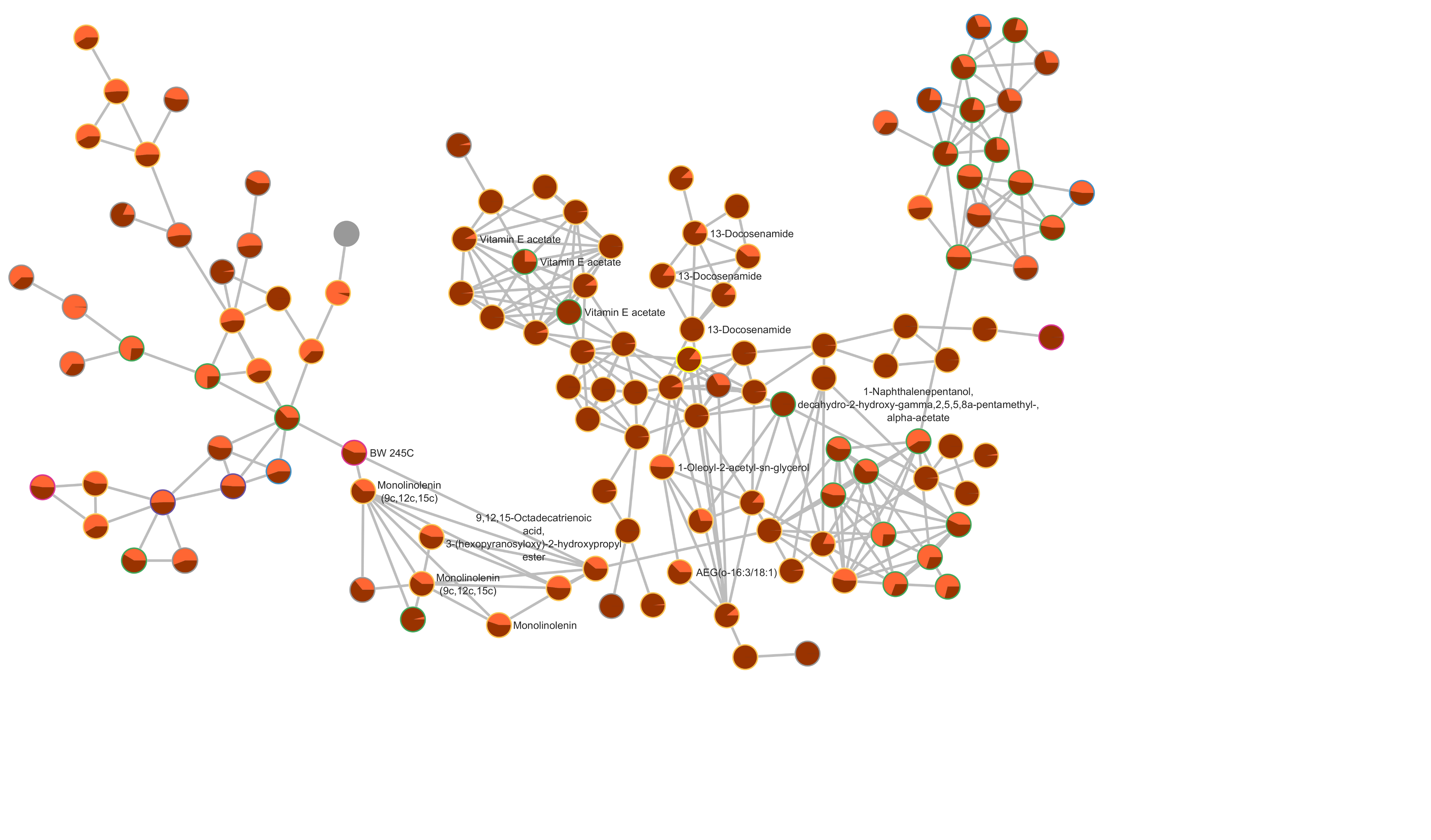

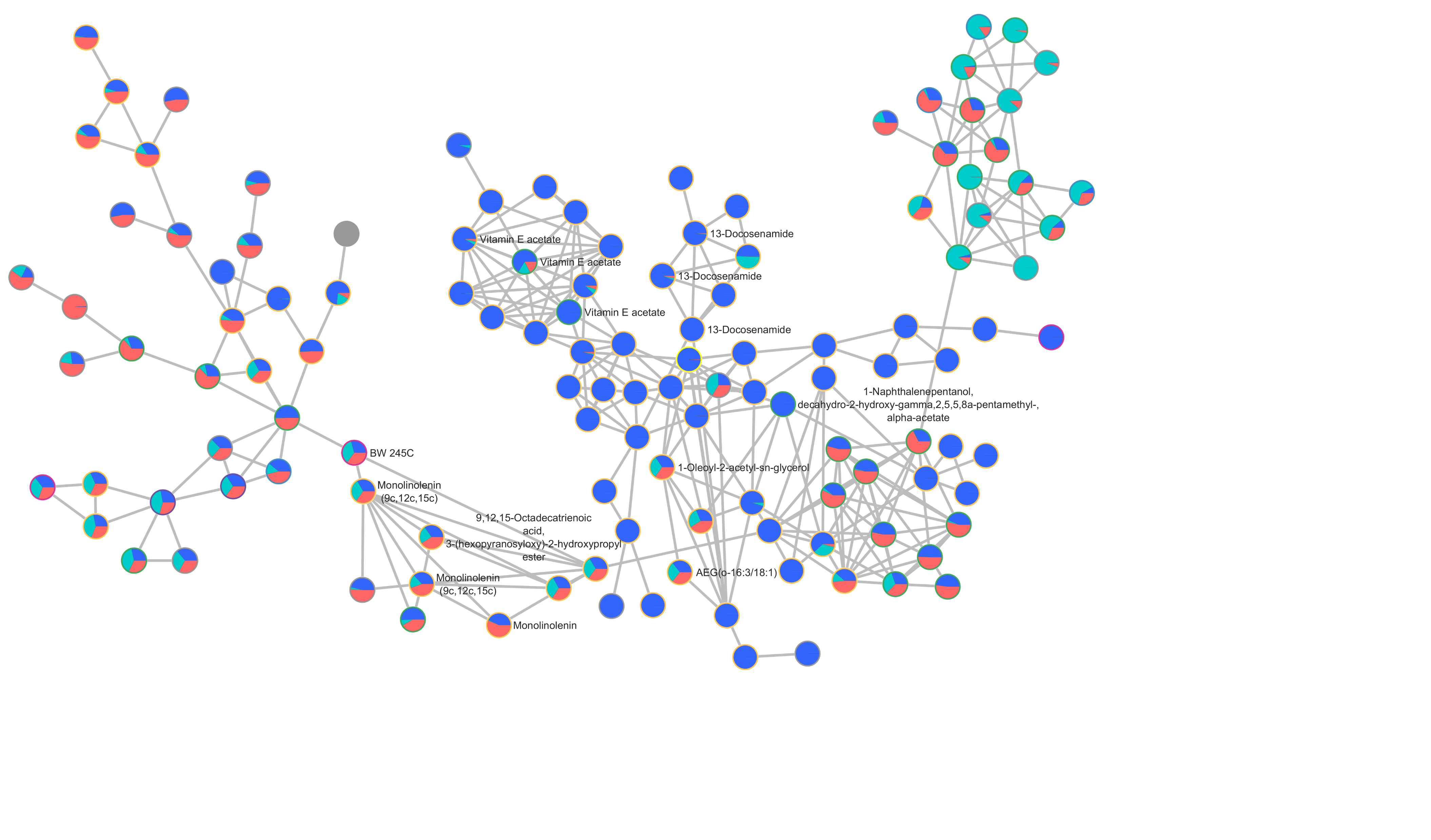

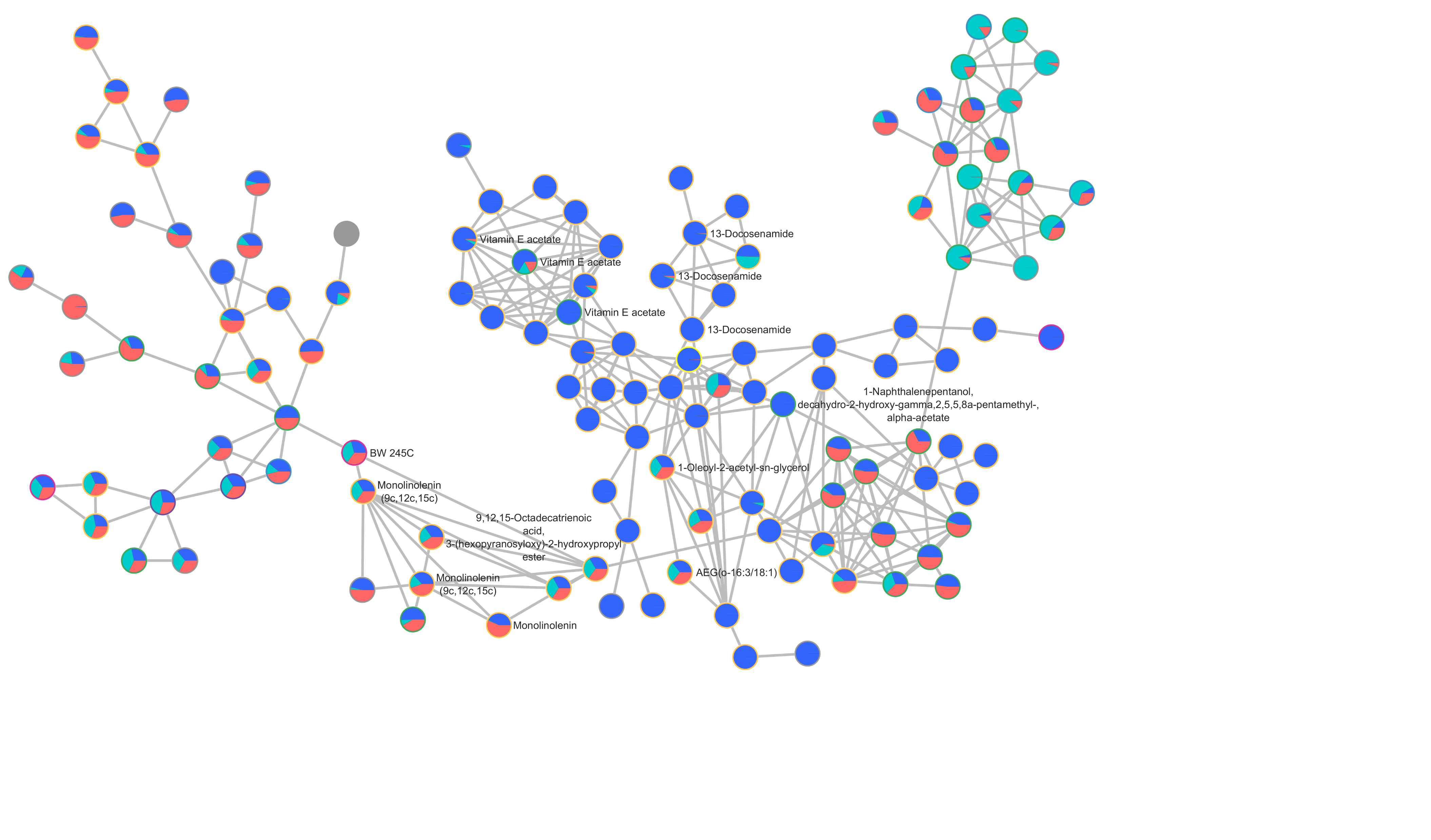

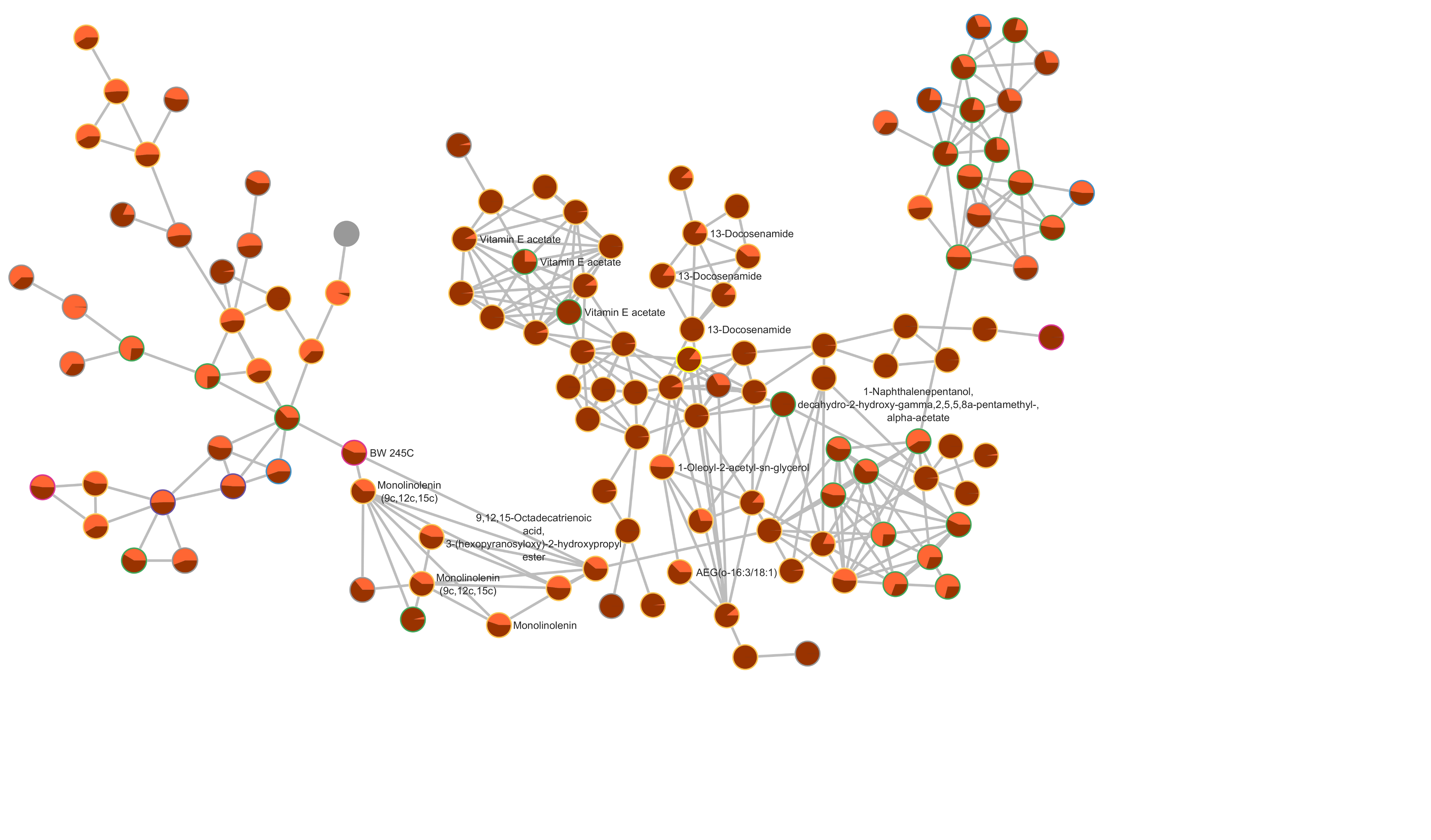


**Summer**

**Fall**

**Winter**

**Sunlight**

**Shaded**

**Figure S5.** Cluster analysis presenting trends across seasons and cultivation conditions (full sunlight and shaded area). Three major regions are observed based on biochemical pathways and seasonal color patterns: Fall exhibits the highest diversity of biochemical classes, Summer shows a higher abundance of fatty acids, and Winter highlights terpenoids. When considering biochemical pathways and cultivation condition color patterns, two distinct regions emerge: plants grown in full sunlight display greater biochemical class diversity, while those cultivated in shaded areas are enriched in fatty acids and terpenoids.

**Figure S6.** Clusters with DMT considering seasons or cultivation conditions (full sunlight and shaded area). The alkaloids in these clusters exhibit similar abundance across the experimental groups.


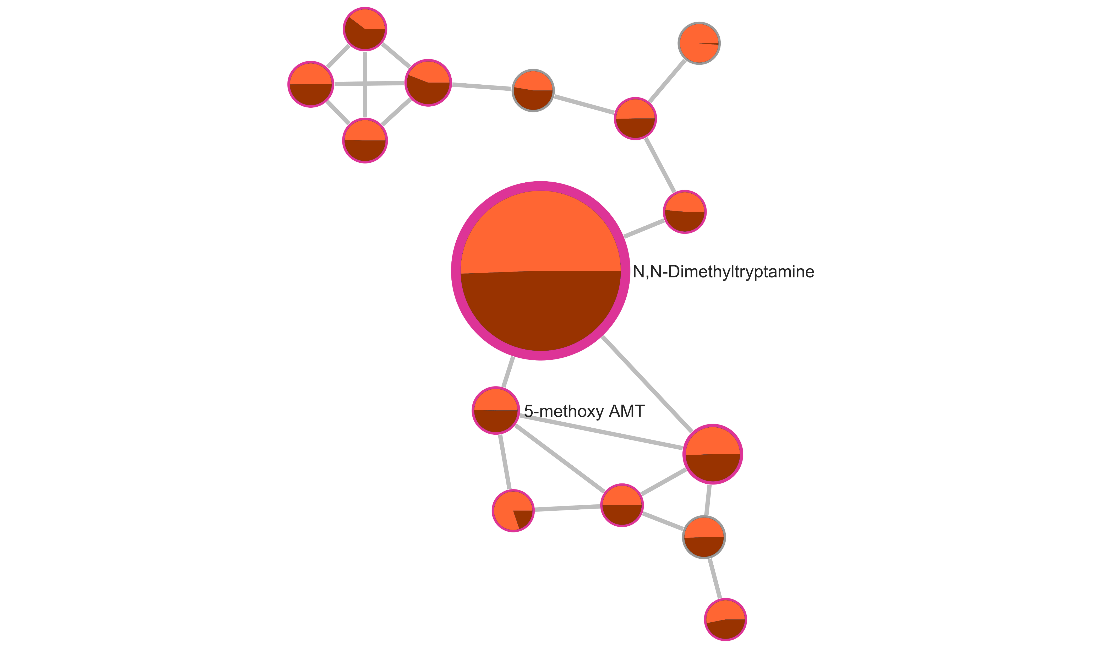

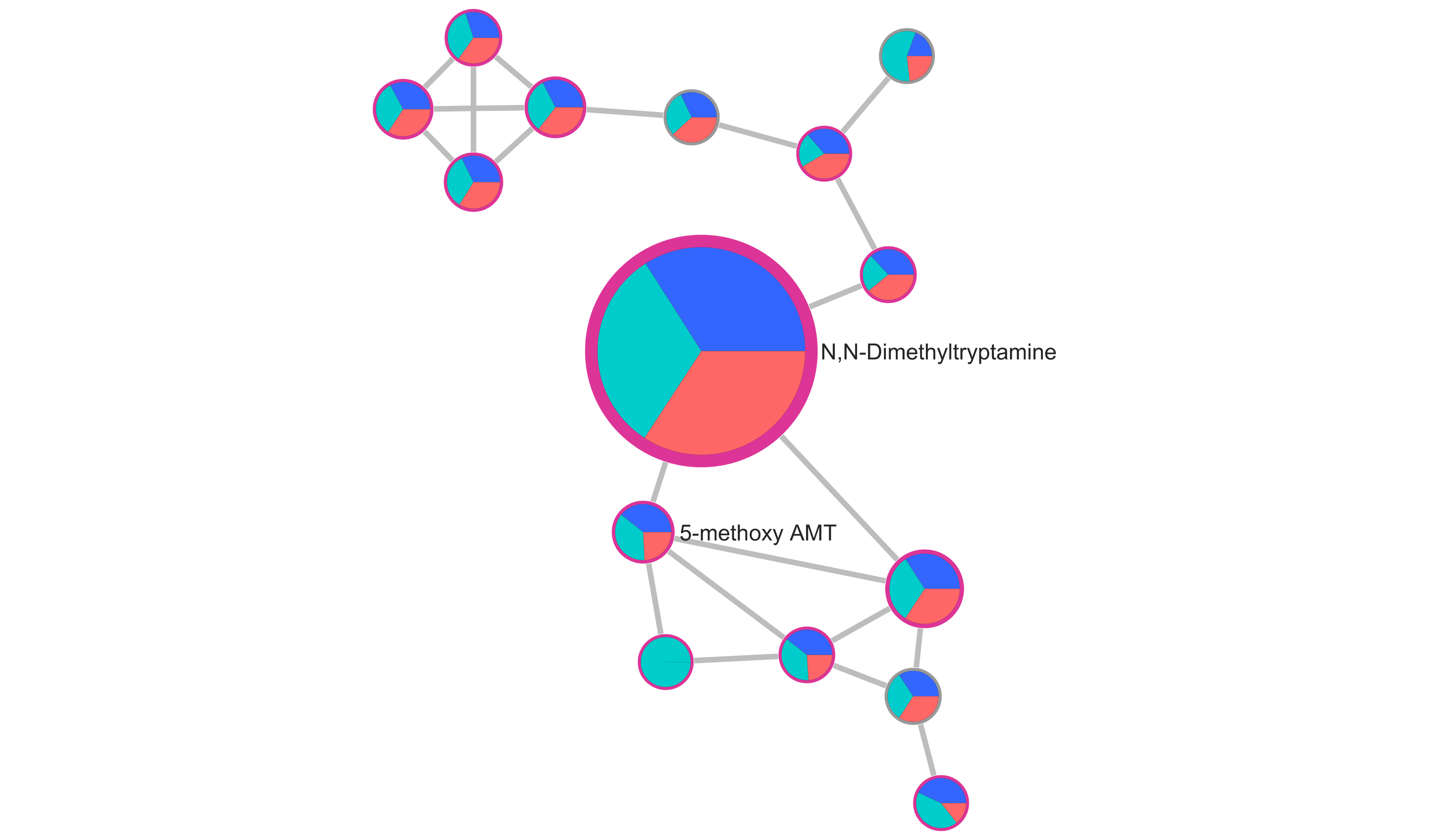

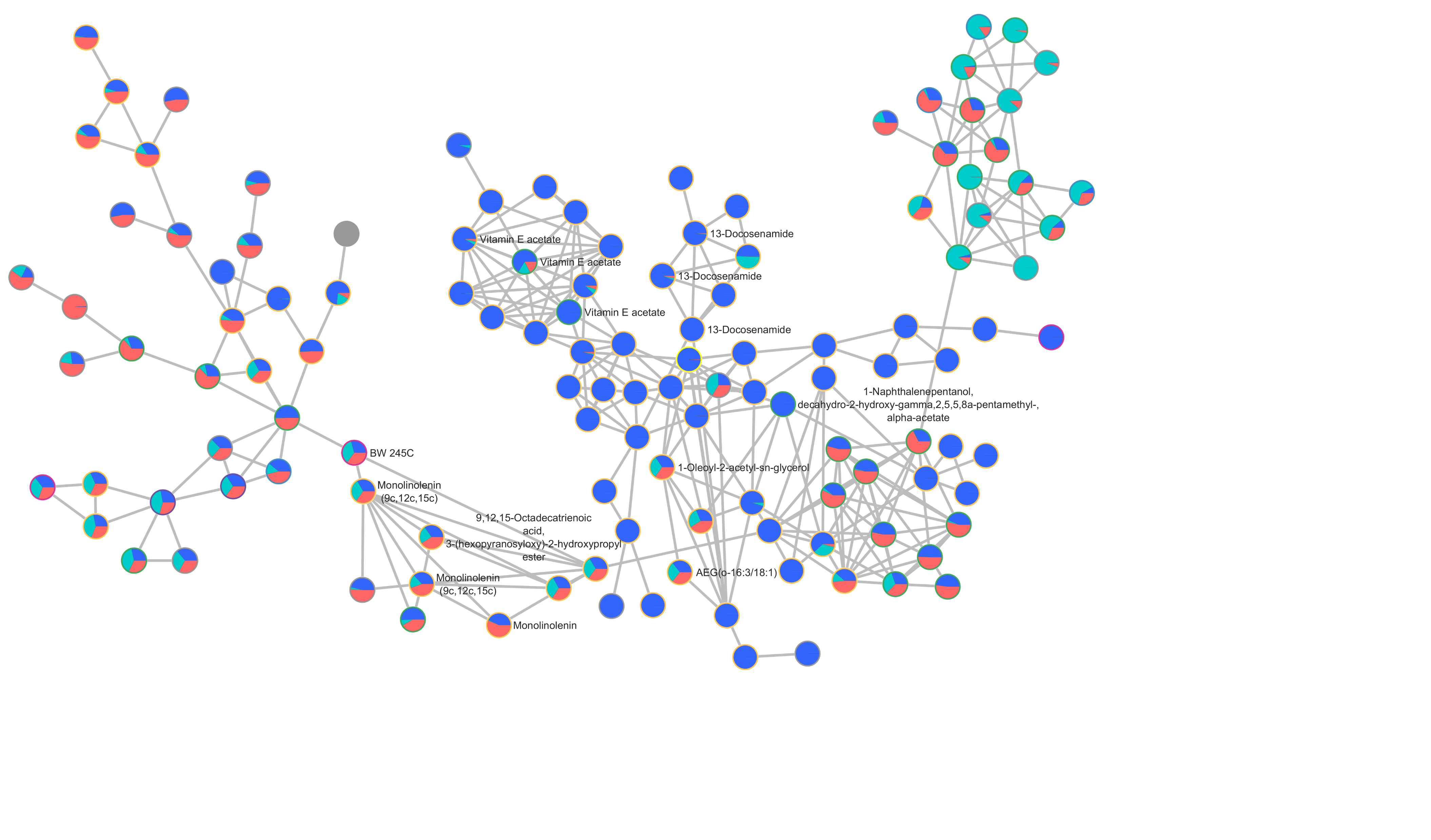

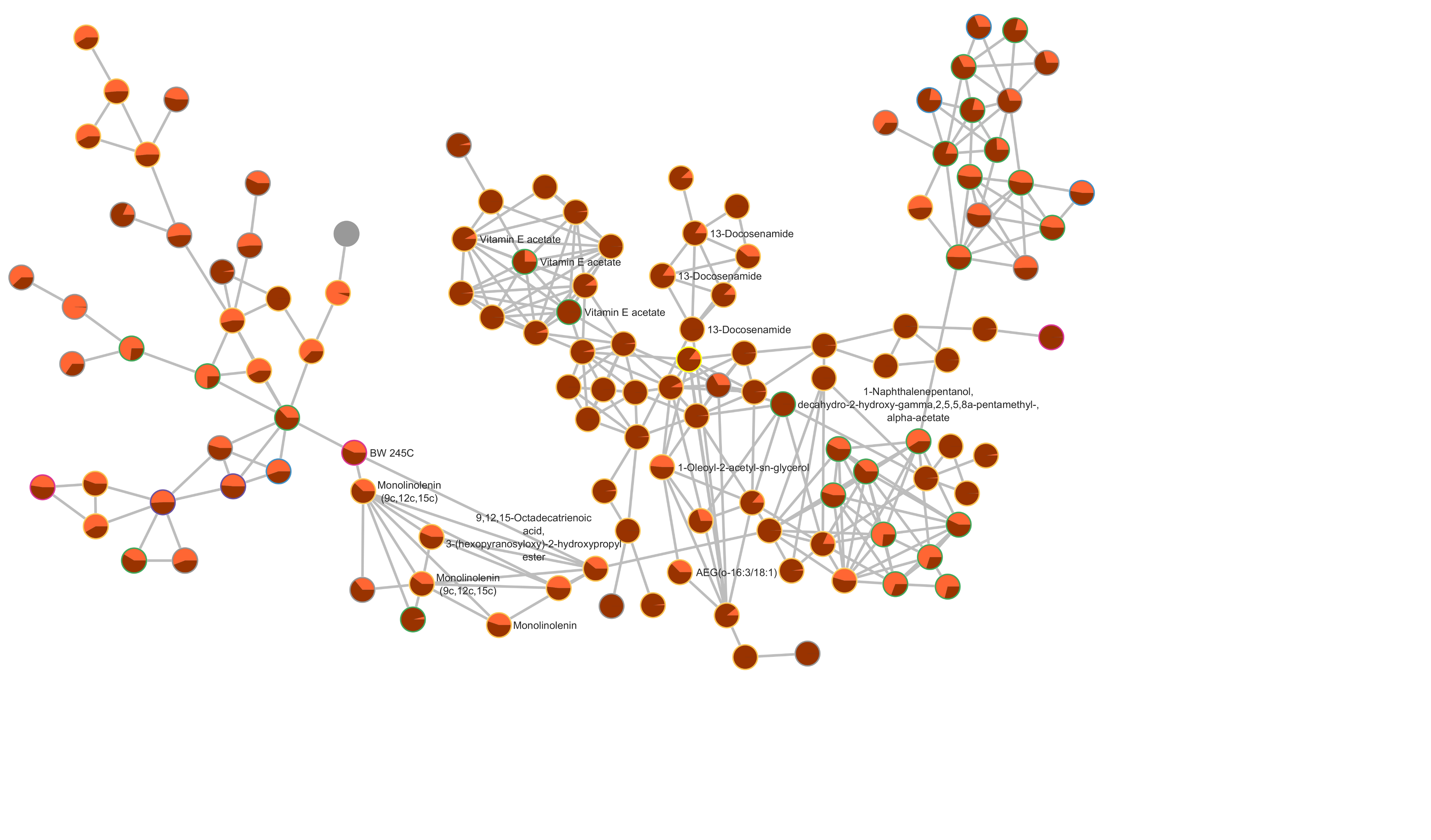

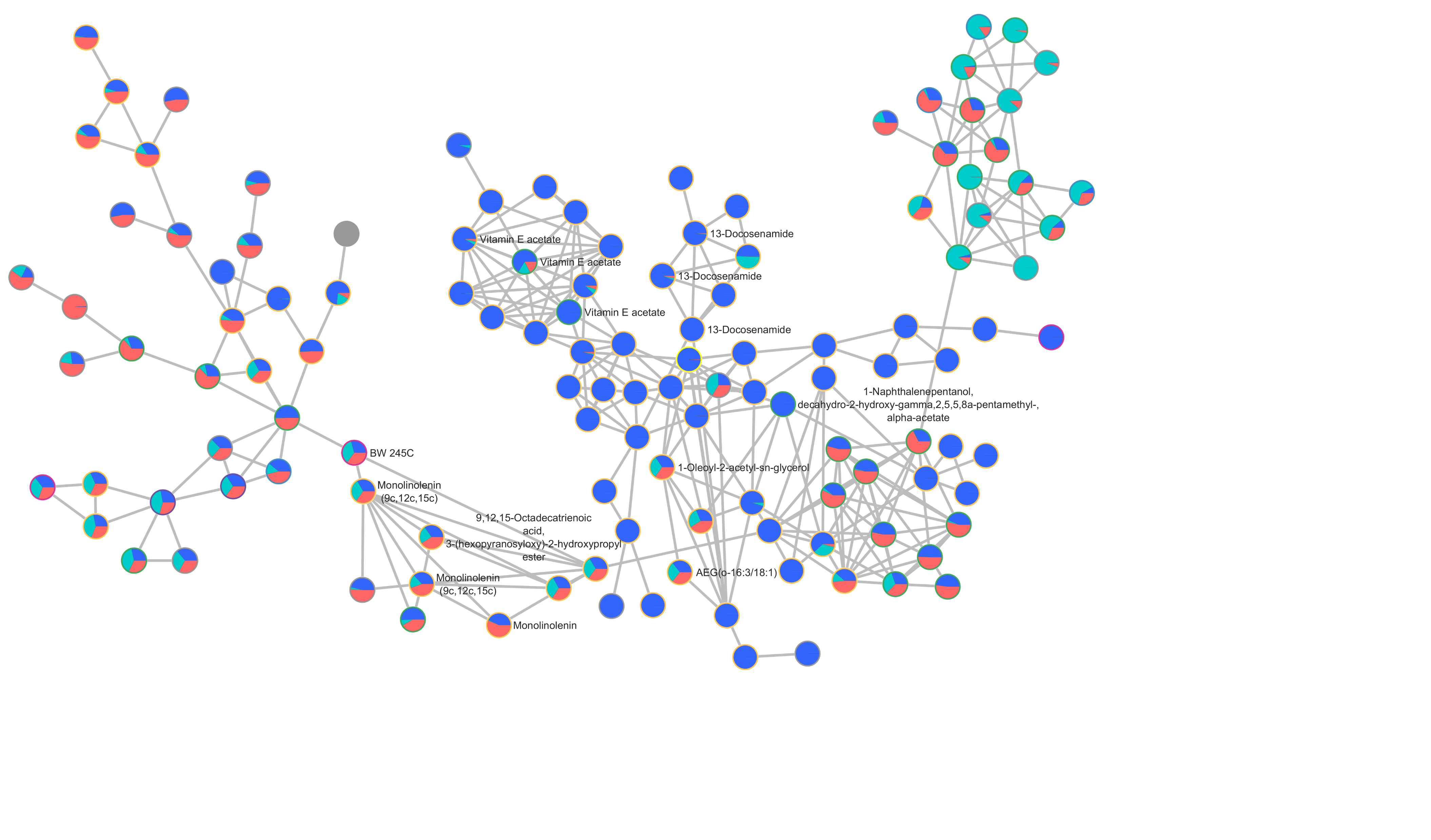

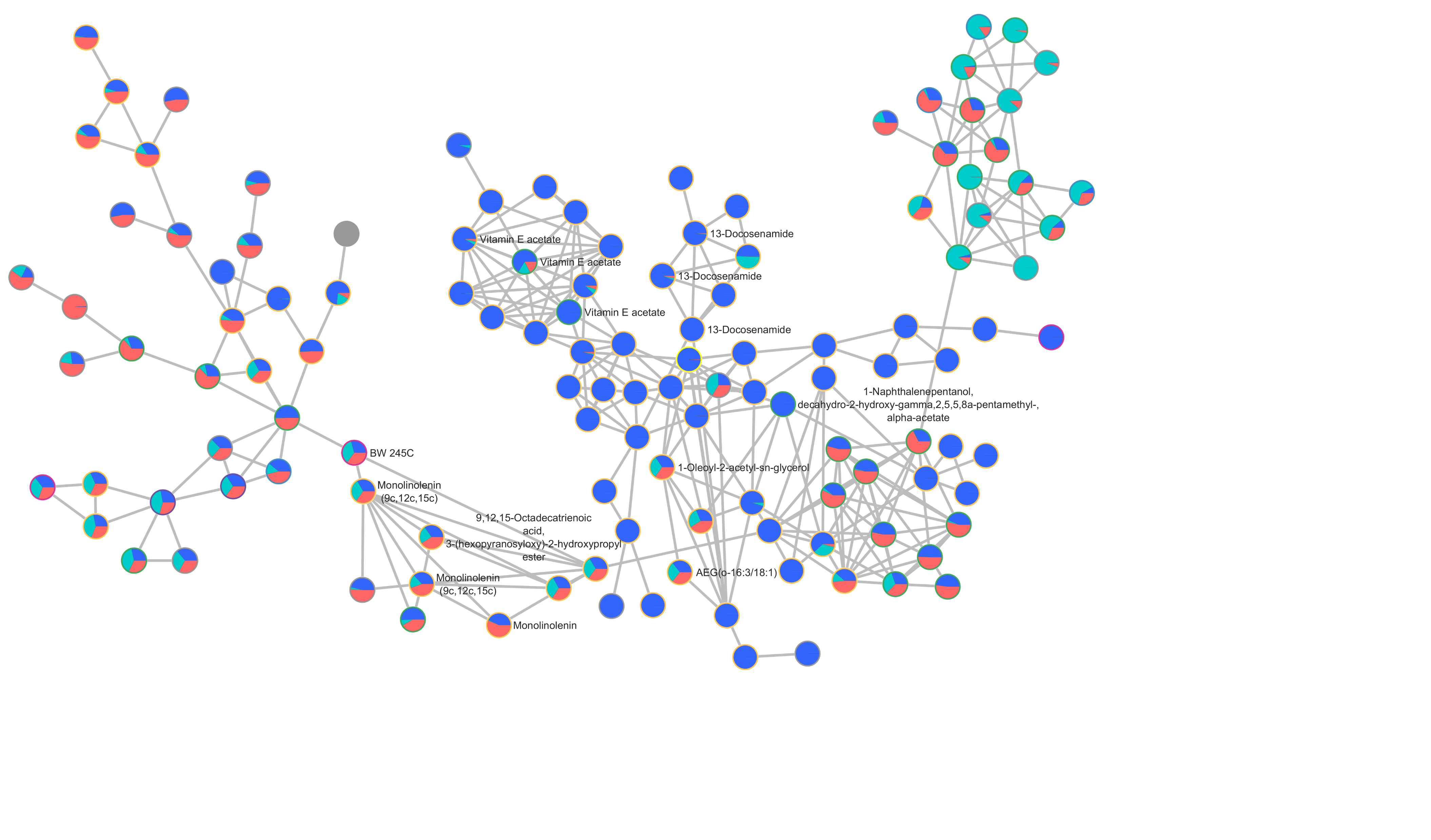

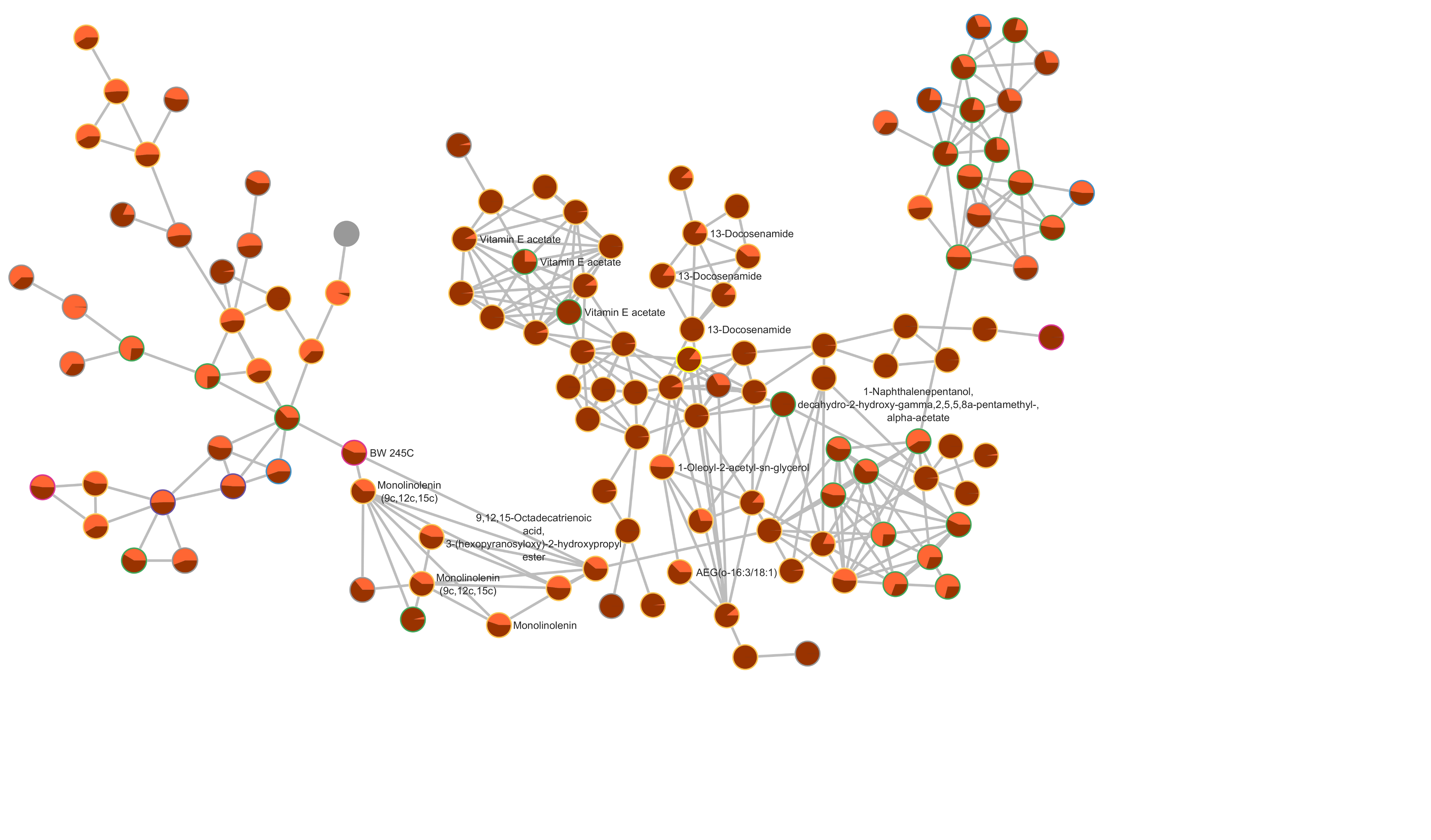


**Summer**

**Fall**

**Winter**

**Sunlight**

**Shaded**

**Table S1.** Annotated compounds based on MS/MS in aqueous phase negative ionization mode data. All annotations were as [M-H]^-^ adduct type.

| **ID** | **RT (min)** | ***m/z*** | **Metabolite name** |
| --- | --- | --- | --- |
| 769 | 8.29 | 121.0292 | Niacinamide |
| 1136 | 0.65 | 135.0300 | Hypoxanthine |
| 1343 | 4.78 | 144.0454 | 2-hydroxyquinoline |
| 1344 | 5.49 | 144.0454 | 2-hydroxyquinoline |
| 1345 | 5.03 | 144.0454 | 2-hydroxyquinoline |
| 1346 | 5.55 | 144.0454 | 2-hydroxyquinoline |
| 1347 | 5.43 | 144.0454 | 2-hydroxyquinoline |
| 1349 | 6.97 | 144.0455 | 2-hydroxyquinoline |
| 1620 | 4.31 | 153.0191 | Patulin |
| 1623 | 2.58 | 153.0194 | Patulin |
| 1624 | 5.82 | 153.0195 | Patulin |
| 1626 | 4.53 | 153.0195 | Patulin |
| 1627 | 4.39 | 153.0195 | Pyrocatechuic acid |
| 1631 | 2.03 | 153.0196 | Pyrocatechuic acid |
| 1841 | 5.22 | 161.0240 | Umbelliferone |
| 1847 | 8.46 | 161.0246 | Umbelliferone |
| 2023 | 5.28 | 165.0193 | Piperonylic acid |
| 2216 | 0.74 | 173.0092 | Aconitic acid |
| 2342 | 6.31 | 175.0249 | Ascorbic acid |
| 2390 | 6.87 | 177.0191 | Esculetin |
| 2392 | 5.32 | 177.0193 | Esculetin |
| 2547 | 8.73 | 181.0510 | 4-O-methylphloracetophenone |
| 2651 | 7.68 | 187.0974 | Azelaic acid |
| 2652 | 6.87 | 187.0974 | Azelaic acid |
| 2654 | 6.59 | 187.0974 | Azelaic acid |
| 2655 | 6.83 | 187.0974 | Azelaic acid |
| 2656 | 8.28 | 187.0974 | Azelaic acid |
| 2663 | 7.62 | 187.0980 | Azelaic acid |
| 2664 | 6.35 | 187.0980 | Azelaic acid |
| 2746 | 5.03 | 188.0351 | 4-Hydroxyquinoline-2-carboxylic acid |
| 2747 | 4.94 | 188.0352 | 4-Hydroxyquinoline-2-carboxylic acid |
| 2749 | 4.91 | 188.0357 | 4-Hydroxyquinoline-2-carboxylic acid |
| 2865 | 1.25 | 191.0560 | 1,3,4,5-tetrahydroxycyclohexane-1-carboxylic acid |
| 2867 | 1.69 | 191.0560 | 1,3,4,5-tetrahydroxycyclohexane-1-carboxylic acid |
| 2868 | 5.59 | 191.0561 | 1,3,4,5-tetrahydroxycyclohexane-1-carboxylic acid |
| 2869 | 4.83 | 191.0561 | 1,3,4,5-tetrahydroxycyclohexane-1-carboxylic acid |
| 2870 | 5.01 | 191.0561 | 1,3,4,5-tetrahydroxycyclohexane-1-carboxylic acid |
| 2871 | 5.22 | 191.0561 | 1,3,4,5-tetrahydroxycyclohexane-1-carboxylic acid |
| 2872 | 4.95 | 191.0561 | 1,3,4,5-tetrahydroxycyclohexane-1-carboxylic acid |
| 2873 | 1.84 | 191.0561 | 1,3,4,5-tetrahydroxycyclohexane-1-carboxylic acid |
| 2876 | 4.92 | 191.0562 | 1,3,4,5-tetrahydroxycyclohexane-1-carboxylic acid |
| 2877 | 1.20 | 191.0562 | 1,3,4,5-tetrahydroxycyclohexane-1-carboxylic acid |

**Table S1.** Annotated compounds based on MS/MS in aqueous phase negative ionization mode data. All annotations were as [M-H]^-^ adduct type. (cont.)

| **ID** | **RT (min)** | ***m/z*** | **Metabolite name** |
| --- | --- | --- | --- |
| 2878 | 1.77 | 191.0562 | 1,3,4,5-tetrahydroxycyclohexane-1-carboxylic acid |
| 2879 | 2.03 | 191.0562 | 1,3,4,5-tetrahydroxycyclohexane-1-carboxylic acid |
| 2880 | 1.60 | 191.0562 | 1,3,4,5-tetrahydroxycyclohexane-1-carboxylic acid |
| 2882 | 0.65 | 191.0562 | 1,3,4,5-tetrahydroxycyclohexane-1-carboxylic acid |
| 2883 | 4.69 | 191.0562 | 1,3,4,5-tetrahydroxycyclohexane-1-carboxylic acid |
| 2884 | 4.77 | 191.0563 | 1,3,4,5-tetrahydroxycyclohexane-1-carboxylic acid |
| 2952 | 6.41 | 193.0504 | Ferulic acid |
| 2953 | 6.74 | 193.0504 | Isoferulic acid |
| 3229 | 2.83 | 203.0824 | Tryptophan |
| 3230 | 2.89 | 203.0824 | Tryptophan |
| 3231 | 3.10 | 203.0824 | Tryptophan |
| 3233 | 3.47 | 203.0830 | Tryptophan |
| 3234 | 3.40 | 203.0830 | Tryptophan |
| 3235 | 4.63 | 203.0830 | Tryptophan |
| 3236 | 3.23 | 203.0831 | Tryptophan |
| 3237 | 2.95 | 203.0831 | Tryptophan |
| 3271 | 9.32 | 205.0873 | 4-hydroxy-3-(3-methylbut-2-enyl)benzoic acid |
| 3368 | 6.17 | 206.0820 | N-acetylphenylalanine |
| 3558 | 0.65 | 209.0304 | Mucic acid |
| 3621 | 9.76 | 211.1339 | Dihydrojasmonic acid |
| 4233 | 0.79 | 233.1543 | 4-(hydroxymethyl)-11,11-dimethyl-8-methylidenebicyclo[7.2.0]undec-4-en-3-one |
| 4234 | 0.73 | 233.1543 | 4-(hydroxymethyl)-11,11-dimethyl-8-methylidenebicyclo[7.2.0]undec-4-en-3-one |
| 4235 | 0.89 | 233.1544 | 4-(hydroxymethyl)-11,11-dimethyl-8-methylidenebicyclo[7.2.0]undec-4-en-3-one |
| 4236 | 11.88 | 233.1545 | 4-(hydroxymethyl)-11,11-dimethyl-8-methylidenebicyclo[7.2.0]undec-4-en-3-one |

**Table S1.** Annotated compounds based on MS/MS in aqueous phase negative ionization mode data. All annotations were as [M-H]^-^ adduct type. (cont.)

| **ID** | **RT (min)** | ***m/z*** | **Metabolite name** |
| --- | --- | --- | --- |
| 4237 | 2.22 | 233.1545 | 4-(hydroxymethyl)-11,11-dimethyl-8-methylidenebicyclo[7.2.0]undec-4-en-3-one |
| 4238 | 11.82 | 233.1551 | 4-(hydroxymethyl)-11,11-dimethyl-8-methylidenebicyclo[7.2.0]undec-4-en-3-one |
| 4239 | 0.49 | 233.1551 | 4-(hydroxymethyl)-11,11-dimethyl-8-methylidenebicyclo[7.2.0]undec-4-en-3-one |
| 4403 | 9.15 | 239.0716 | 2',4'-dihydroxychalcone |
| 4511 | 0.94 | 243.0619 | Arabinofuranosyluracil |
| 4554 | 6.56 | 245.0930 | N-acetyltryptophan |
| 4709 | 8.47 | 253.0508 | Rubiadin |
| 4745 | 8.33 | 255.0699 | Isoliquiritigenin |
| 4899 | 7.23 | 261.1345 | 9-(2,3-dihydroxypropoxy)-9-oxononanoic acid |
| 4957 | 8.45 | 263.1293 | Abscisic acid |
| 4999 | 11.16 | 265.1480 | Zinniol |
| 5076 | 9.22 | 269.0851 | Isoimperatorin |
| 5077 | 9.01 | 269.0861 | Isoimperatorin |
| 5117 | 7.51 | 271.0618 | 3',4',7-trihydroxyflavanone |
| 5131 | 10.88 | 271.0978 | 2',6'-dihydroxy-4'-methoxydihydrochalcone |
| 5372 | 5.75 | 281.1396 | 5,9-dihydroxy-7-(hydroxymethyl)-5,7-dimethyl-4,5a,6,8,8a,9-hexahydro-1H-azuleno[5,6-c]furan-3-one |
| 5392 | 10.18 | 283.0250 | Rhein |
| 5427 | 8.99 | 285.0406 | Fisetin |
| 5550 | 5.59 | 289.0718 | Epicatechin |
| 5551 | 5.14 | 289.0718 | Catechin |
| 5749 | 11.96 | 295.2275 | 9-hydroxy-10,12-octadecadienoic acid |
| 5788 | 11.38 | 297.1533 | Aurapten |
| 5791 | 10.41 | 297.1533 | Aurapten |
| 5797 | 11.51 | 297.1534 | Aurapten |

**Table S1.** Annotated compounds based on MS/MS in aqueous phase negative ionization mode data. All annotations were as [M-H]^-^ adduct type. (cont.)

| **ID** | **RT (min)** | ***m/z*** | **Metabolite name** |
| --- | --- | --- | --- |
| 5802 | 11.59 | 297.1534 | Aurapten |
| 5935 | 0.42 | 300.9996 | Ellagic acid |
| 5938 | 0.28 | 300.9997 | Ellagic acid |
| 5939 | 8.50 | 301.0356 | Quercetin |
| 6031 | 4.97 | 305.0667 | 2-(3,4,5-trihydroxyphenyl)-3,4-dihydro-2H-chromene-3,5,7-triol |
| 6033 | 2.49 | 305.0668 | 2-(3,4,5-trihydroxyphenyl)-3,4-dihydro-2H-chromene-3,5,7-triol |
| 6320 | 8.29 | 311.1686 | Triptophenolide |
| 6321 | 0.27 | 311.1686 | Triptophenolide |
| 6322 | 0.42 | 311.1686 | Triptophenolide |
| 6323 | 5.71 | 311.1686 | Triptophenolide |
| 6327 | 15.64 | 311.1687 | Triptophenolide |
| 6331 | 11.74 | 311.1687 | Triptophenolide |
| 6332 | 10.85 | 311.1687 | Triptophenolide |
| 6333 | 12.09 | 311.1687 | Triptophenolide |
| 6334 | 16.83 | 311.1687 | Triptophenolide |
| 6335 | 12.49 | 311.1687 | Triptophenolide |
| 6336 | 12.02 | 311.1688 | Triptophenolide |
| 6339 | 11.61 | 311.1688 | Triptophenolide |
| 6340 | 13.29 | 311.1688 | Triptophenolide |
| 6345 | 15.74 | 311.1689 | Triptophenolide |
| 6346 | 10.67 | 311.1689 | Triptophenolide |
| 6348 | 12.33 | 311.1689 | Triptophenolide |
| 6349 | 15.82 | 311.1689 | Triptophenolide |
| 6351 | 13.63 | 311.1689 | Triptophenolide |
| 6352 | 13.60 | 311.1689 | Triptophenolide |
| 6354 | 12.29 | 311.1690 | Triptophenolide |
| 6355 | 11.20 | 311.1690 | Triptophenolide |
| 6468 | 9.09 | 315.0510 | 3-methylquercetin |
| 6469 | 8.70 | 315.0512 | 3-methylquercetin |
| 6785 | 14.17 | 325.1844 | Hydroquinidine |
| 6786 | 12.91 | 325.1845 | Hydroquinidine |
| 6787 | 14.54 | 325.1845 | Hydroquinidine |
| 6788 | 8.29 | 325.1845 | Hydroquinidine |
| 6789 | 8.01 | 325.1845 | Hydroquinidine |
| 6790 | 0.27 | 325.1845 | Hydroquinidine |
| 6791 | 5.71 | 325.1845 | Hydroquinidine |
| 6793 | 14.31 | 325.1845 | Hydroquinidine |
| 6794 | 11.38 | 325.1845 | Hydroquinidine |
| 6795 | 0.42 | 325.1845 | Hydroquinidine |
| 6796 | 15.89 | 325.1845 | Hydroquinidine |
| 6797 | 10.69 | 325.1845 | Hydroquinidine |

**Table S1.** Annotated compounds based on MS/MS in aqueous phase negative ionization mode data. All annotations were as [M-H]^-^ adduct type. (cont.)

| **ID** | **RT (min)** | ***m/z*** | **Metabolite name** |
| --- | --- | --- | --- |
| 6798 | 13.80 | 325.1846 | Hydroquinidine |
| 6799 | 13.18 | 325.1846 | Hydroquinidine |
| 6800 | 11.16 | 325.1846 | Hydroquinidine |
| 6802 | 16.85 | 325.1846 | Hydroquinidine |
| 6803 | 13.06 | 325.1846 | Hydroquinidine |
| 6804 | 11.09 | 325.1846 | Hydroquinidine |
| 6806 | 13.55 | 325.1847 | Hydroquinidine |
| 6807 | 12.98 | 325.1847 | Hydroquinidine |
| 6808 | 14.47 | 325.1847 | Hydroquinidine |
| 6809 | 15.71 | 325.1847 | Hydroquinidine |
| 6810 | 13.42 | 325.1847 | Hydroquinidine |
| 6811 | 12.57 | 325.1847 | Hydroquinidine |
| 6812 | 15.66 | 325.1847 | Hydroquinidine |
| 6813 | 11.52 | 325.1847 | Hydroquinidine |
| 6814 | 15.97 | 325.1847 | Hydroquinidine |
| 6815 | 16.05 | 325.1847 | Hydroquinidine |
| 6816 | 13.47 | 325.1848 | Hydroquinidine |
| 6817 | 13.74 | 325.1848 | Hydroquinidine |
| 6819 | 12.81 | 325.1848 | Hydroquinidine |
| 6820 | 13.95 | 325.1848 | Hydroquinidine |
| 6821 | 15.80 | 325.1848 | Hydroquinidine |
| 6823 | 13.64 | 325.1848 | Hydroquinidine |
| 6825 | 12.74 | 325.1849 | Hydroquinidine |
| 6826 | 13.10 | 325.1849 | Hydroquinidine |
| 6828 | 11.02 | 325.2024 | Hydroquinidine |
| 6839 | 11.52 | 325.2388 | Avocadyne acetate |
| 6840 | 11.75 | 325.2388 | Avocadyne acetate |
| 7252 | 4.80 | 339.0725 | Aesculin |
| 7351 | 1.33 | 341.1086 | Trehalose |
| 7352 | 0.67 | 341.1087 | Trehalose |
| 7354 | 1.29 | 341.1087 | Trehalose |
| 7358 | 1.23 | 341.1088 | Trehalose |
| 7399 | 9.67 | 343.0833 | Santin |
| 7759 | 5.59 | 353.0876 | Chlorogenic acid |
| 7760 | 5.22 | 353.0876 | Chlorogenic acid |
| 7762 | 1.41 | 353.0877 | 4-{[(2E)-3-(3,4-Dihydroxyphenyl)-2-propenoyl]oxy}-1,3,5-trihydroxycyclohexanecarboxylic acid |
| 7764 | 1.46 | 353.0877 | 4-{[(2E)-3-(3,4-Dihydroxyphenyl)-2-propenoyl]oxy}-1,3,5-trihydroxycyclohexanecarboxylic acid |
| 7767 | 4.99 | 353.0878 | Chlorogenic acid |
| 7768 | 2.26 | 353.0878 | 4-{[(2E)-3-(3,4-Dihydroxyphenyl)-2-propenoyl]oxy}-1,3,5-trihydroxycyclohexanecarboxylic acid |

**Table S1.** Annotated compounds based on MS/MS in aqueous phase negative ionization mode data. All annotations were as [M-H]^-^ adduct type. (cont.)

| **ID** | **RT (min)** | ***m/z*** | **Metabolite name** |
| --- | --- | --- | --- |
| 7769 | 4.92 | 353.0878 | Chlorogenic acid |
| 7770 | 4.28 | 353.0878 | 4-{[(2E)-3-(3,4-Dihydroxyphenyl)-2-propenoyl]oxy}-1,3,5-trihydroxycyclohexanecarboxylic acid |
| 7771 | 1.52 | 353.0878 | 4-{[(2E)-3-(3,4-Dihydroxyphenyl)-2-propenoyl]oxy}-1,3,5-trihydroxycyclohexanecarboxylic acid |
| 7772 | 2.15 | 353.0878 | 4-{[(2E)-3-(3,4-Dihydroxyphenyl)-2-propenoyl]oxy}-1,3,5-trihydroxycyclohexanecarboxylic acid |
| 7773 | 6.52 | 353.0878 | Undulatoside A |
| 7776 | 2.06 | 353.0878 | 4-{[(2E)-3-(3,4-Dihydroxyphenyl)-2-propenoyl]oxy}-1,3,5-trihydroxycyclohexanecarboxylic acid |
| 7777 | 1.70 | 353.0878 | 4-{[(2E)-3-(3,4-Dihydroxyphenyl)-2-propenoyl]oxy}-1,3,5-trihydroxycyclohexanecarboxylic acid |
| 7778 | 3.98 | 353.0878 | 4-{[(2E)-3-(3,4-Dihydroxyphenyl)-2-propenoyl]oxy}-1,3,5-trihydroxycyclohexanecarboxylic acid |
| 7780 | 2.87 | 353.0879 | 4-{[(2E)-3-(3,4-Dihydroxyphenyl)-2-propenoyl]oxy}-1,3,5-trihydroxycyclohexanecarboxylic acid |
| 7781 | 6.03 | 353.0879 | Undulatoside A |
| 7782 | 1.64 | 353.0879 | 4-{[(2E)-3-(3,4-Dihydroxyphenyl)-2-propenoyl]oxy}-1,3,5-trihydroxycyclohexanecarboxylic acid |
| 7783 | 1.06 | 353.0879 | 4-{[(2E)-3-(3,4-Dihydroxyphenyl)-2-propenoyl]oxy}-1,3,5-trihydroxycyclohexanecarboxylic acid |
| 7784 | 1.77 | 353.0879 | 4-{[(2E)-3-(3,4-Dihydroxyphenyl)-2-propenoyl]oxy}-1,3,5-trihydroxycyclohexanecarboxylic acid |
| 7785 | 4.66 | 353.0879 | Chlorogenic acid |
| 7786 | 1.11 | 353.0879 | 4-{[(2E)-3-(3,4-Dihydroxyphenyl)-2-propenoyl]oxy}-1,3,5-trihydroxycyclohexanecarboxylic acid |
| 7788 | 4.83 | 353.0880 | Chlorogenic acid |
| 7791 | 4.78 | 353.0880 | Chlorogenic acid |
| 7792 | 0.76 | 353.0880 | Undulatoside A |
| 7793 | 1.83 | 353.0880 | 4-{[(2E)-3-(3,4-Dihydroxyphenyl)-2-propenoyl]oxy}-1,3,5-trihydroxycyclohexanecarboxylic acid |
| 7798 | 1.58 | 353.0880 | 4-{[(2E)-3-(3,4-Dihydroxyphenyl)-2-propenoyl]oxy}-1,3,5-trihydroxycyclohexanecarboxylic acid |
| 8245 | 5.87 | 367.1030 | 3-O-Feruloylquinic acid |
| 8336 | 5.99 | 371.0985 | 6-(3-benzoyloxy-2-hydroxypropoxy)-3,4,5-trihydroxyoxane-2-carboxylic acid |
| 9564 | 5.48 | 413.1107 | Asperuloside |
| 9706 | 7.30 | 417.0833 | Kaempferol 3-alpha-arabinopyranoside |

**Table S1.** Annotated compounds based on MS/MS in aqueous phase negative ionization mode data. All annotations were as [M-H]^-^ adduct type. (cont.)

| **ID** | **RT (min)** | ***m/z*** | **Metabolite name** |
| --- | --- | --- | --- |
| 9707 | 6.18 | 417.0837 | Kaempferol 3-alpha-L-arabinopyranoside |
| 10133 | 4.66 | 431.1190 | 7-(acetyloxymethyl)-5-hydroxy-1-[(2S,3R,4S,5S,6R)-3,4,5-trihydroxy-6-(hydroxymethyl)oxan-2-yl]oxy-1,4a,5,7a-tetrahydrocyclopenta[c]pyran-4-carboxylic acid |
| 10134 | 5.18 | 431.1190 | 7-(acetyloxymethyl)-5-hydroxy-1-[(2S,3R,4S,5S,6R)-3,4,5-trihydroxy-6-(hydroxymethyl)oxan-2-yl]oxy-1,4a,5,7a-tetrahydrocyclopenta[c]pyran-4-carboxylic acid |
| 10135 | 4.80 | 431.1191 | 7-(acetyloxymethyl)-5-hydroxy-1-[(2S,3R,4S,5S,6R)-3,4,5-trihydroxy-6-(hydroxymethyl)oxan-2-yl]oxy-1,4a,5,7a-tetrahydrocyclopenta[c]pyran-4-carboxylic acid |
| 10137 | 4.74 | 431.1192 | 7-(acetyloxymethyl)-5-hydroxy-1-[(2S,3R,4S,5S,6R)-3,4,5-trihydroxy-6-(hydroxymethyl)oxan-2-yl]oxy-1,4a,5,7a-tetrahydrocyclopenta[c]pyran-4-carboxylic acid |
| 10138 | 4.86 | 431.1192 | 7-(acetyloxymethyl)-5-hydroxy-1-[(2S,3R,4S,5S,6R)-3,4,5-trihydroxy-6-(hydroxymethyl)oxan-2-yl]oxy-1,4a,5,7a-tetrahydrocyclopenta[c]pyran-4-carboxylic acid |
| 10139 | 3.34 | 431.1192 | 7-(acetyloxymethyl)-5-hydroxy-1-[(2S,3R,4S,5S,6R)-3,4,5-trihydroxy-6-(hydroxymethyl)oxan-2-yl]oxy-1,4a,5,7a-tetrahydrocyclopenta[c]pyran-4-carboxylic acid |
| 10140 | 4.43 | 431.1192 | 7-(acetyloxymethyl)-5-hydroxy-1-[(2S,3R,4S,5S,6R)-3,4,5-trihydroxy-6-(hydroxymethyl)oxan-2-yl]oxy-1,4a,5,7a-tetrahydrocyclopenta[c]pyran-4-carboxylic acid |
| 10143 | 4.47 | 431.1193 | 7-(acetyloxymethyl)-5-hydroxy-1-[(2S,3R,4S,5S,6R)-3,4,5-trihydroxy-6-(hydroxymethyl)oxan-2-yl]oxy-1,4a,5,7a-tetrahydrocyclopenta[c]pyran-4-carboxylic acid |
| 10214 | 6.74 | 433.0787 | Guajavarin |
| 10674 | 7.66 | 447.0925 | Quercitrin |
| 10675 | 7.88 | 447.0926 | Quercitrin |
| 10676 | 7.37 | 447.0926 | Quercitrin |
| 10677 | 6.99 | 447.0928 | Kaempferol 7-O-glucoside |
| 10678 | 5.97 | 447.0928 | Kaempferol 7-O-glucoside |
| 10679 | 6.75 | 447.0928 | Kaempferol 7-O-glucoside |
| 11226 | 6.39 | 463.0880 | Hyperoside |
| 11227 | 7.82 | 463.0881 | Hyperoside |
| 11229 | 7.15 | 463.0882 | Hyperoside |

**Table S1.** Annotated compounds based on MS/MS in aqueous phase negative ionization mode data. All annotations were as [M-H]^-^ adduct type. (cont.)

| **ID** | **RT (min)** | ***m/z*** | **Metabolite name** |
| --- | --- | --- | --- |
| 11230 | 7.37 | 463.0882 | Hyperoside |
| 11231 | 5.59 | 463.0882 | Hyperoside |
| 11232 | 5.41 | 463.0883 | Hyperoside |
| 11234 | 6.34 | 463.0885 | Hyperoside |
| 11677 | 7.05 | 477.1051 | Isorhamnetin 3-galactoside |
| 11745 | 5.90 | 479.0834 | 5,7-dihydroxy-3-[3,4,5-trihydroxy-6-(hydroxymethyl)oxan-2-yl]oxy-2-(3,4,5-trihydroxyphenyl)chromen-4-one |
| 12030 | 10.51 | 487.3427 | 1,8,10-trihydroxy-1,2,6a,6b,9,9,12a-heptamethyl-2,3,4,5,6,6a,7,8,8a,10,11,12,13,14b-tetradecahydropicene-4a-carboxylic acid |
| 12032 | 10.85 | 487.3430 | 1,8,10-trihydroxy-1,2,6a,6b,9,9,12a-heptamethyl-2,3,4,5,6,6a,7,8,8a,10,11,12,13,14b-tetradecahydropicene-4a-carboxylic acid |
| 12034 | 10.13 | 487.3430 | 1,8,10-trihydroxy-1,2,6a,6b,9,9,12a-heptamethyl-2,3,4,5,6,6a,7,8,8a,10,11,12,13,14b-tetradecahydropicene-4a-carboxylic acid |
| 12035 | 10.34 | 487.3431 | 1,8,10-trihydroxy-1,2,6a,6b,9,9,12a-heptamethyl-2,3,4,5,6,6a,7,8,8a,10,11,12,13,14b-tetradecahydropicene-4a-carboxylic acid |
| 12036 | 11.04 | 487.3432 | Asiatic Acid |
| 12509 | 9.88 | 503.3380 | 1,10,11-trihydroxy-9-(hydroxymethyl)-2,2,6a,6b,9,12a-hexamethyl-1,3,4,5,6,6a,7,8,8a,10,11,12,13,14b-tetradecahydropicene-4a-carboxylic acid |
| 12511 | 9.92 | 503.3382 | 1,10,11-trihydroxy-9-(hydroxymethyl)-2,2,6a,6b,9,12a-hexamethyl-1,3,4,5,6,6a,7,8,8a,10,11,12,13,14b-tetradecahydropicene-4a-carboxylic acid |
| 12819 | 6.99 | 515.1197 | 3,5-bis({[(2E)-3-(3,4-dihydroxyphenyl)prop-2-enoyl]oxy})-1,4-dihydroxycyclohexane-1-carboxylic acid |
| 12820 | 6.81 | 515.1197 | 3,4-di-O-caffeoylquinic acid |
| 12822 | 7.52 | 515.1198 | 3,5-bis({[(2E)-3-(3,4-dihydroxyphenyl)prop-2-enoyl]oxy})-1,4-dihydroxycyclohexane-1-carboxylic acid |
| 12823 | 7.20 | 515.1198 | 3,5-bis({[(2E)-3-(3,4-dihydroxyphenyl)prop-2-enoyl]oxy})-1,4-dihydroxycyclohexane-1-carboxylic acid |
| 13858 | 6.68 | 549.0902 | Quercetin 3-O-malonylglucoside |
| 14896 | 5.75 | 579.1342 | 5,7-dihydroxy-2-(4-hydroxyphenyl)-3-[3,4,5-trihydroxy-6-[[3,4,5-trihydroxyoxan-2-yl]oxymethyl]oxan-2-yl]oxychromen-4-one |

**Table S1.** Annotated compounds based on MS/MS in aqueous phase negative ionization mode data. All annotations were as [M-H]^-^ adduct type. (cont.)

| **ID** | **RT (min)** | ***m/z*** | **Metabolite name** |
| --- | --- | --- | --- |
| 14900 | 6.48 | 579.1345 | 5,7-dihydroxy-2-(4-hydroxyphenyl)-3-[3,4,5-trihydroxy-6-[[3,4,5-trihydroxyoxan-2-yl]oxymethyl]oxan-2-yl]oxychromen-4-one |
| 14901 | 6.42 | 579.1347 | 5,7-dihydroxy-2-(4-hydroxyphenyl)-3-[3,4,5-trihydroxy-6-[[3,4,5-trihydroxyoxan-2-yl]oxymethyl]oxan-2-yl]oxychromen-4-one |
| 15294 | 6.48 | 593.1497 | Kaempferol 7-neohesperidoside |
| 15295 | 5.82 | 593.1497 | Kaempferol 7-neohesperidoside |
| 15297 | 6.70 | 593.1530 | Kaempferol-3-O-rutinoside |
| 15337 | 6.08 | 595.1312 | 2-(3,4-dihydroxyphenyl)-5,7-dihydroxy-3-[3,4,5-trihydroxy-6-[[3,4,5-trihydroxyoxan-2-yl]oxymethyl]oxan-2-yl]oxychromen-4-one |
| 15664 | 6.58 | 609.1452 | 3-[4,5-dihydroxy-6-(hydroxymethyl)-3-[-3,4,5-trihydroxyoxan-2-yl]oxyoxan-2-yl]oxy-2-(3,4-dihydroxyphenyl)-5-hydroxy-7-methoxychromen-4-one |
| 15667 | 6.53 | 609.1457 | Rutin |
| 15670 | 6.17 | 609.1458 | Rutin |
| 15988 | 6.77 | 623.1628 | 5,7-dihydroxy-2-(4-hydroxy-3-methoxyphenyl)-3-[3,4,5-trihydroxy-6-[[3,4,5-trihydroxy-6-methyloxan-2-yl]oxymethyl]oxan-2-yl]oxychromen-4-one |
| 19137 | 5.81 | 755.2066 | 3-[6-[[3,5-dihydroxy-6-methyl-4-[3,4,5-trihydroxy-6-methyloxan-2-yl]oxyoxan-2-yl]oxymethyl]-3,4,5-trihydroxyoxan-2-yl]oxy-2-(3,4-dihydroxyphenyl)-5,7-dihydroxychromen-4-one |
| 19166 | 5.66 | 757.1842 | 3-[4-[4,5-dihydroxy-6-(hydroxymethyl)-3-[3,4,5-trihydroxyoxan-2-yl]oxyoxan-2-yl]oxy-3,5-dihydroxy-6-(hydroxymethyl)oxan-2-yl]oxy-2-(3,4-dihydroxyphenyl)-5,7-dihydroxychromen-4-one |

**Table S2.** Annotated compounds based on MS/MS in aqueous phase positive ionization mode data.

| **ID** | **RT (min)** | ***m/z*** | **Type** | **Metabolite name** |
| --- | --- | --- | --- | --- |
| 156 | 0.64 | 104.0707 | [M+H]^+^ | Gamma-Aminobutyric Acid |
| 736 | 3.66 | 118.0862 | [M+NH_4_]^+^ | Gyromitrin |
| 741 | 3.73 | 118.0863 | [M+NH_4_]^+^ | Gyromitrin |
| 743 | 3.41 | 118.0863 | [M+NH_4_]^+^ | Gyromitrin |
| 744 | 0.64 | 118.0863 | [M+NH_4_]^+^ | Gyromitrin |
| 794 | 0.81 | 120.0808 | [M+NH_4_]^+^ | Cycloserine |
| 795 | 1.00 | 120.0809 | [M+NH_4_]^+^ | Cycloserine |
| 796 | 0.93 | 120.0809 | [M+NH_4_]^+^ | Cycloserine |
| 797 | 2.18 | 120.0810 | [M+NH_4_]^+^ | Cycloserine |
| 1416 | 0.71 | 136.0619 | [M+H]^+^ | Adenine |
| 1463 | 0.65 | 138.0549 | [M+H]^+^ | Trigonelline |
| 1804 | 4.73 | 146.0601 | [M+H]^+^ | 2-hydroxyquinoline |
| 1806 | 5.46 | 146.0602 | [M+H]^+^ | 2-hydroxyquinoline |
| 2381 | 5.28 | 161.1072 | [M+H]^+^ | Tryptamine |
| 2382 | 5.20 | 161.1072 | [M+H]^+^ | Tryptamine |
| 2384 | 5.24 | 161.1076 | [M+H]^+^ | Tryptamine |
| 2408 | 0.75 | 163.0389 | [M+H]^+^ | Umbelliferone |
| 2464 | 7.26 | 164.1071 | [M+H]^+^ | N-acetyl-2-phenylethylamine |
| 2544 | 5.08 | 167.0705 | [M+H]^+^ | Isopeonol |
| 3234 | 6.12 | 183.0655 | [M+H]^+^ | 4-O-methylphloracetophenone |
| 3743 | 0.74 | 198.0760 | [M+H]^+^ | Levodopa |
| 3974 | 4.59 | 205.0972 | [M+H]^+^ | Tryptophan |
| 3976 | 0.68 | 205.1178 | [M+H]^+^ | 1-[4-hydroxy-3-(3-methylbut-2-enyl)phenyl]ethenone |
| 3979 | 5.42 | 205.1333 | [M+H]^+^ | 1-[4-hydroxy-3-(3-methylbut-2-enyl)phenyl]ethenone |
| 3980 | 5.47 | 205.1333 | [M+H]^+^ | 1-[4-hydroxy-3-(3-methylbut-2-enyl)phenyl]ethenone |
| 3986 | 5.36 | 205.1335 | [M+H]^+^ | 1-[4-hydroxy-3-(3-methylbut-2-enyl)phenyl]ethenone |
| 4149 | 1.05 | 209.0919 | [M+H]^+^ | Kynurenine |
| 4150 | 2.04 | 209.0919 | [M+H]^+^ | Kynurenine |
| 4151 | 0.96 | 209.0919 | [M+H]^+^ | Kynurenine |
| 4152 | 0.89 | 209.0923 | [M+H]^+^ | Kynurenine |
| 6026 | 6.41 | 271.0599 | [M+H]^+^ | 7,3',4'-trihydroxyflavone |
| 6465 | 6.75 | 287.0543 | [M+H]^+^ | Fisetin |
| 6466 | 7.29 | 287.0544 | [M+H]^+^ | 3',4',5,7-tetrahydroxyflavone |
| 6467 | 5.99 | 287.0544 | [M+H]^+^ | Fisetin |
| 6468 | 6.46 | 287.0544 | [M+H]^+^ | Luteolin |
| 6469 | 6.12 | 287.0544 | [M+H]^+^ | Fisetin |
| 6470 | 7.06 | 287.0547 | [M+H]^+^ | 3',4',5,7-tetrahydroxyflavone |
| 6471 | 8.99 | 287.0554 | [M+H]^+^ | 3',4',5,7-tetrahydroxyflavone |
| 6548 | 0.74 | 289.0918 | [M+H]^+^ | 2-methyl-3-[3,4,5-trihydroxy-6-(hydroxymethyl)oxan-2-yl]oxypyran-4-one |
| 6599 | 5.13 | 291.0858 | [M+H]^+^ | Epicatechin |
| 6600 | 5.93 | 291.0859 | [M+H]^+^ | Epicatechin |
| 6601 | 5.58 | 291.0859 | [M+H]^+^ | Epicatechin |
| 6602 | 5.77 | 291.0863 | [M+H]^+^ | Epicatechin |
| 6697 | 9.07 | 293.2113 | [M+H]^+^ | 8-[3-oxo-2-[(E)-pent-2-enyl]cyclopenten-1-yl]octanoic acid |
| 6698 | 11.69 | 293.2114 | [M+H]^+^ | 8-[3-oxo-2-[(E)-pent-2-enyl]cyclopenten-1-yl]octanoic acid |

**Table S2.** Annotated compounds based on MS/MS in aqueous phase positive ionization mode data. (cont.)

| **ID** | **RT (min)** | ***m/z*** | **Type** | **Metabolite name** |
| --- | --- | --- | --- | --- |
| 7043 | 6.49 | 303.0491 | [M+H]^+^ | 4-(2-methoxy-2-oxoethyl)-5-[2-[(E)-3-phenylprop-2-enoyl]oxyethylidene]-6-[3,4,5-trihydroxy-6-(hydroxymethyl)oxan-2-yl]oxy-4H-pyran-3-carboxylic acid |
| 7046 | 5.74 | 303.0493 | [M+H]^+^ | 4-(2-methoxy-2-oxoethyl)-5-[2-[(E)-3-phenylprop-2-enoyl]oxyethylidene]-6-[3,4,5-trihydroxy-6-(hydroxymethyl)oxan-2-yl]oxy-4H-pyran-3-carboxylic acid |
| 7047 | 8.49 | 303.0499 | [M+H]^+^ | 4-(2-methoxy-2-oxoethyl)-5-[2-[(E)-3-phenylprop-2-enoyl]oxyethylidene]-6-[3,4,5-trihydroxy-6-(hydroxymethyl)oxan-2-yl]oxy-4H-pyran-3-carboxylic acid |
| 7048 | 6.74 | 303.0500 | [M+H]^+^ | 4-(2-methoxy-2-oxoethyl)-5-[2-[(E)-3-phenylprop-2-enoyl]oxyethylidene]-6-[3,4,5-trihydroxy-6-(hydroxymethyl)oxan-2-yl]oxy-4H-pyran-3-carboxylic acid |
| 7049 | 7.45 | 303.0500 | [M+H]^+^ | 4-(2-methoxy-2-oxoethyl)-5-[2-[(E)-3-phenylprop-2-enoyl]oxyethylidene]-6-[3,4,5-trihydroxy-6-(hydroxymethyl)oxan-2-yl]oxy-4H-pyran-3-carboxylic acid |
| 7050 | 6.33 | 303.0500 | [M+H]^+^ | 4-(2-methoxy-2-oxoethyl)-5-[2-[(E)-3-phenylprop-2-enoyl]oxyethylidene]-6-[3,4,5-trihydroxy-6-(hydroxymethyl)oxan-2-yl]oxy-4H-pyran-3-carboxylic acid |
| 7051 | 5.87 | 303.0500 | [M+H]^+^ | 4-(2-methoxy-2-oxoethyl)-5-[2-[(E)-3-phenylprop-2-enoyl]oxyethylidene]-6-[3,4,5-trihydroxy-6-(hydroxymethyl)oxan-2-yl]oxy-4H-pyran-3-carboxylic acid |
| 7052 | 6.08 | 303.0500 | [M+H]^+^ | 4-(2-methoxy-2-oxoethyl)-5-[2-[(E)-3-phenylprop-2-enoyl]oxyethylidene]-6-[3,4,5-trihydroxy-6-(hydroxymethyl)oxan-2-yl]oxy-4H-pyran-3-carboxylic acid |
| 7236 | 10.06 | 309.1310 | [M+Na]^+^ | methyl 2-ethyl-4-[5-hydroxy-4,5-dimethyl-2-oxooxolan-3-yl]-2-methyl-3-oxobutanoate |
| 7476 | 8.69 | 317.0650 | [M+H]^+^ | 3-methylquercetin |
| 8000 | 10.53 | 333.2036 | [M+Na]^+^ | Dihydroalbocycline |
| 8314 | 9.67 | 345.0965 | [M+H]^+^ | Santin |
| 8598 | 1.39 | 355.1022 | [M+H]^+^ | 4-{[(2E)-3-(3,4-Dihydroxyphenyl)-2-propenoyl]oxy}-1,3,5-trihydroxycyclohexanecarboxylic acid |
| 8599 | 0.75 | 355.1023 | [M+H]^+^ | Chlorogenic acid |
| 8600 | 4.81 | 355.1023 | [M+H]^+^ | 4-{[(2E)-3-(3,4-Dihydroxyphenyl)-2-propenoyl]oxy}-1,3,5-trihydroxycyclohexanecarboxylic acid |
| 8601 | 4.26 | 355.1023 | [M+H]^+^ | 4-{[(2E)-3-(3,4-Dihydroxyphenyl)-2-propenoyl]oxy}-1,3,5-trihydroxycyclohexanecarboxylic acid |
| 8602 | 1.77 | 355.1023 | [M+H]^+^ | 4-{[(2E)-3-(3,4-Dihydroxyphenyl)-2-propenoyl]oxy}-1,3,5-trihydroxycyclohexanecarboxylic acid |
| 8603 | 4.96 | 355.1024 | [M+H]^+^ | 4-{[(2E)-3-(3,4-Dihydroxyphenyl)-2-propenoyl]oxy}-1,3,5-trihydroxycyclohexanecarboxylic acid |
| 8604 | 1.33 | 355.1024 | [M+H]^+^ | 4-{[(2E)-3-(3,4-Dihydroxyphenyl)-2-propenoyl]oxy}-1,3,5-trihydroxycyclohexanecarboxylic acid |
| 8606 | 4.90 | 355.1024 | [M+H]^+^ | 4-{[(2E)-3-(3,4-Dihydroxyphenyl)-2-propenoyl]oxy}-1,3,5-trihydroxycyclohexanecarboxylic acid |
| 8607 | 5.22 | 355.1025 | [M+H]^+^ | 4-{[(2E)-3-(3,4-Dihydroxyphenyl)-2-propenoyl]oxy}-1,3,5-trihydroxycyclohexanecarboxylic acid |
| 8608 | 1.19 | 355.1026 | [M+H]^+^ | 4-{[(2E)-3-(3,4-Dihydroxyphenyl)-2-propenoyl]oxy}-1,3,5-trihydroxycyclohexanecarboxylic acid |
| 8609 | 2.03 | 355.1028 | [M+H]^+^ | 4-{[(2E)-3-(3,4-Dihydroxyphenyl)-2-propenoyl]oxy}-1,3,5-trihydroxycyclohexanecarboxylic acid |

**Table S2.** Annotated compounds based on MS/MS in aqueous phase positive ionization mode data. (cont.)

| **ID** | **RT (min)** | ***m/z*** | **Type** | **Metabolite name** |
| --- | --- | --- | --- | --- |
| 8610 | 5.59 | 355.1029 | [M+H]^+^ | 4-{[(2E)-3-(3,4-Dihydroxyphenyl)-2-propenoyl]oxy}-1,3,5-trihydroxycyclohexanecarboxylic acid |
| 8611 | 1.53 | 355.1031 | [M+H]^+^ | Chlorogenic acid |
| 8680 | 12.48 | 357.3000 | [M+H]^+^ | Monoolein |
| 8769 | 0.83 | 360.1496 | [M+NH_4_]^+^ | Gentiobiose |
| 8770 | 0.67 | 360.1505 | [M+NH_4_]^+^ | Gentiobiose |
| 8800 | 12.70 | 361.2352 | [M+Na]^+^ | 2-(2-hydroxybut-3-en-2-yl)-3a,6,6,9a-tetramethyl-2,4,5,5a,7,8,9,9b-octahydro-1H-benzo[e][1]benzofuran-4,5-diol |
| 8879 | 0.65 | 365.1046 | [M+Na]^+^ | Melibiose |
| 9099 | 5.21 | 372.1280 | [M+NH_4_]^+^ | Chlorogenic acid |
| 9100 | 5.59 | 372.1284 | [M+NH_4_]^+^ | Chlorogenic acid |
| 9183 | 7.52 | 375.2370 | [M+H]^+^ | 2-(hydroxymethyl)-6-[4-(4-hydroxy-2,6,6-trimethylcyclohexen-1-yl)butan-2-yloxy]oxane-3,4,5-triol |
| 9185 | 6.97 | 375.2371 | [M+H]^+^ | 2-(hydroxymethyl)-6-[4-(4-hydroxy-2,6,6-trimethylcyclohexen-1-yl)butan-2-yloxy]oxane-3,4,5-triol |
| 9187 | 5.82 | 375.2373 | [M+H]^+^ | 2-(hydroxymethyl)-6-[4-(4-hydroxy-2,6,6-trimethylcyclohexen-1-yl)butan-2-yloxy]oxane-3,4,5-triol |
| 9188 | 6.67 | 375.2377 | [M+H]^+^ | 2-(hydroxymethyl)-6-[4-(4-hydroxy-2,6,6-trimethylcyclohexen-1-yl)butan-2-yloxy]oxane-3,4,5-triol |
| 9223 | 4.73 | 377.0831 | [M+Na]^+^ | Chlorogenic acid |
| 9225 | 5.22 | 377.0844 | [M+Na]^+^ | Chlorogenic acid |
| 9226 | 4.80 | 377.0844 | [M+Na]^+^ | Chlorogenic acid |
| 9227 | 4.95 | 377.0845 | [M+Na]^+^ | Chlorogenic acid |
| 9228 | 4.91 | 377.0845 | [M+Na]^+^ | Chlorogenic acid |
| 9235 | 5.64 | 377.1456 | [M+H]^+^ | Riboflavin |
| 10312 | 6.17 | 419.0969 | [M+H]^+^ | Kaempferol 3-alpha-L-arabinopyranoside |
| 10313 | 7.29 | 419.0978 | [M+H]^+^ | Kaempferol 3-alpha-L-arabinopyranoside |
| 10314 | 7.06 | 419.0979 | [M+H]^+^ | Kaempferol 3-alpha-L-arabinopyranoside |
| 10633 | 7.89 | 433.1123 | [M+H]^+^ | Afzelin |
| 10637 | 5.34 | 433.1343 | [M+H]^+^ | 3-hydroxy-3-methyl-5-oxo-5-[[3,4,5-trihydroxy-6-(2-methyl-4-oxopyran-3-yl)oxyoxan-2-yl]methoxy]pentanoic acid |
| 10687 | 6.49 | 435.0922 | [M+H]^+^ | Avicularin |
| 10688 | 6.74 | 435.0923 | [M+H]^+^ | Avicularin |
| 11105 | 6.98 | 449.1076 | [M+H]^+^ | 4-(3,4-dihydroxyphenyl)-7-hydroxy-5-[3,4,5-trihydroxy-6-(hydroxymethyl)oxan-2-yl]oxychromen-2-one |
| 11106 | 6.46 | 449.1076 | [M+H]^+^ | 4-(3,4-dihydroxyphenyl)-7-hydroxy-5-[3,4,5-trihydroxy-6-(hydroxymethyl)oxan-2-yl]oxychromen-2-one |

**Table S2.** Annotated compounds based on MS/MS in aqueous phase positive ionization mode data. (cont.)

| **ID** | **RT (min)** | ***m/z*** | **Type** | **Metabolite name** |
| --- | --- | --- | --- | --- |
| 11107 | 5.96 | 449.1077 | [M+H]^+^ | Luteolin 4'-O-glucoside |
| 11109 | 6.75 | 449.1086 | [M+H]^+^ | 4-(3,4-dihydroxyphenyl)-7-hydroxy-5-[3,4,5-trihydroxy-6-(hydroxymethyl)oxan-2-yl]oxychromen-2-one |
| 11132 | 4.77 | 450.1598 | [M+NH_4_]^+^ | 7-(acetyloxymethyl)-5-hydroxy-1-[3,4,5-trihydroxy-6-(hydroxymethyl)oxan-2-yl]oxy-1,4a,5,7a-tetrahydrocyclopenta[c]pyran-4-carboxylic acid |
| 11133 | 5.17 | 450.1599 | [M+NH_4_]^+^ | 7-(acetyloxymethyl)-5-hydroxy-1-[3,4,5-trihydroxy-6-(hydroxymethyl)oxan-2-yl]oxy-1,4a,5,7a-tetrahydrocyclopenta[c]pyran-4-carboxylic acid |
| 11134 | 4.70 | 450.1599 | [M+NH_4_]^+^ | 7-(acetyloxymethyl)-5-hydroxy-1-[3,4,5-trihydroxy-6-(hydroxymethyl)oxan-2-yl]oxy-1,4a,5,7a-tetrahydrocyclopenta[c]pyran-4-carboxylic acid |
| 11510 | 5.87 | 465.1027 | [M+H]^+^ | Hyperoside |
| 11511 | 6.08 | 465.1029 | [M+H]^+^ | Hyperoside |
| 11512 | 7.16 | 465.1029 | [M+H]^+^ | Hyperoside |
| 11513 | 6.34 | 465.1030 | [M+H]^+^ | Hyperoside |
| 11514 | 5.75 | 465.1035 | [M+H]^+^ | Hyperoside |
| 11515 | 5.59 | 465.1036 | [M+H]^+^ | Hyperoside |
| 11875 | 7.04 | 479.1176 | [M+H]^+^ | Isorhamnetin 3-galactoside |
| 12690 | 6.98 | 517.1344 | [M+H]^+^ | 3,4-di-O-caffeoylquinic acid |
| 12691 | 6.80 | 517.1345 | [M+H]^+^ | 3,4-di-O-caffeoylquinic acid |
| 12693 | 7.51 | 517.1345 | [M+H]^+^ | 3,4-di-O-caffeoylquinic acid |
| 13392 | 6.67 | 551.1016 | [M+H]^+^ | Quercetin 3-O-malonylglucoside |
| 13687 | 7.89 | 565.1551 | [M+H]^+^ | 5-hydroxy-2-(4-hydroxyphenyl)-7-(3,4,5-trihydroxy-6-methyloxan-2-yl)oxy-3-(3,4,5-trihydroxyoxan-2-yl)oxychromen-4-one |
| 13963 | 6.43 | 581.1497 | [M+H]^+^ | 3-[4,5-dihydroxy-6-(hydroxymethyl)-3-[3,4,5-trihydroxyoxan-2-yl]oxyoxan-2-yl]oxy-5,7-dihydroxy-2-(4-hydroxyphenyl)chromen-4-one |
| 13967 | 5.75 | 581.1512 | [M+H]^+^ | 3-[4,5-dihydroxy-6-(hydroxymethyl)-3-[3,4,5-trihydroxyoxan-2-yl]oxyoxan-2-yl]oxy-5,7-dihydroxy-2-(4-hydroxyphenyl)chromen-4-one |
| 14275 | 6.69 | 595.1658 | [M+H]^+^ | Nicotiflorin |
| 14276 | 6.47 | 595.1660 | [M+H]^+^ | 3-[3,5-dihydroxy-6-(hydroxymethyl)-4-[3,4,5-trihydroxy-6-methyloxan-2-yl]oxyoxan-2-yl]oxy-5,7-dihydroxy-2-(4-hydroxyphenyl)chromen-4-one |
| 14277 | 6.12 | 595.1666 | [M+H]^+^ | 3-[3,5-dihydroxy-6-(hydroxymethyl)-4-[3,4,5-trihydroxy-6-methyloxan-2-yl]oxyoxan-2-yl]oxy-5,7-dihydroxy-2-(4-hydroxyphenyl)chromen-4-one |

**Table S2.** Annotated compounds based on MS/MS in aqueous phase positive ionization mode data. (cont.)

| **ID** | **RT (min)** | ***m/z*** | **Type** | **Metabolite name** |
| --- | --- | --- | --- | --- |
| 14278 | 5.82 | 595.1669 | [M+H]^+^ | Nicotiflorin |
| 14317 | 6.08 | 597.1442 | [M+H]^+^ | 2-(3,4-dihydroxyphenyl)-5,7-dihydroxy-3-[3,4,5-trihydroxy-6-[[3,4,5-trihydroxyoxan-2-yl]oxymethyl]oxan-2-yl]oxychromen-4-one |
| 14637 | 5.75 | 611.1595 | [M+H]^+^ | Rutin |
| 14638 | 6.16 | 611.1599 | [M+H]^+^ | Rutin |
| 14639 | 5.87 | 611.1615 | [M+H]^+^ | Rutin |
| 14921 | 6.81 | 625.1761 | [M+H]^+^ | 5,7-dihydroxy-2-(4-hydroxy-3-methoxyphenyl)-3-[3,4,5-trihydroxy-6-[[3,4,5-trihydroxy-6-methyloxan-2-yl]oxymethyl]oxan-2-yl]oxychromen-4-one |
| 16978 | 6.12 | 727.2063 | [M+H]^+^ | 5-hydroxy-3-[3-hydroxy-5-(hydroxymethyl)-4-[3,4,5-trihydroxy-6-(hydroxymethyl)oxan-2-yl]oxyoxolan-2-yl]oxy-2-(4-hydroxyphenyl)-7-[3,4,5-trihydroxy-6-methyloxan-2-yl]oxychromen-4-one |
| 16979 | 5.99 | 727.2064 | [M+H]^+^ | 5-hydroxy-3-[3-hydroxy-5-(hydroxymethyl)-4-[3,4,5-trihydroxy-6-(hydroxymethyl)oxan-2-yl]oxyoxolan-2-yl]oxy-2-(4-hydroxyphenyl)-7-[3,4,5-trihydroxy-6-methyloxan-2-yl]oxychromen-4-one |
| 17632 | 5.15 | 759.1968 | [M+H]^+^ | 3-[4-[4,5-dihydroxy-6-(hydroxymethyl)-3-[3,4,5-trihydroxyoxan-2-yl]oxyoxan-2-yl]oxy-3,5-dihydroxy-6-(hydroxymethyl)oxan-2-yl]oxy-2-(3,4-dihydroxyphenyl)-5,7-dihydroxychromen-4-one |

**Table S3.** Annotated compounds based on MS/MS in organic phase negative ionization mode data.

| **ID** | **RT (min)** | ***m/z*** | **Type** | **Metabolite name** |
| --- | --- | --- | --- | --- |
| 1911 | 0.61 | 179.0564 | [M-H]^-^ | Beta-D-Allose |
| 2741 | 8.51 | 233.1544 | [M-H]^-^ | FA 15:4 |
| 2742 | 11.88 | 233.1545 | [M-H]^-^ | FA 15:4 |
| 2743 | 8.81 | 233.1545 | [M-H]^-^ | FA 15:4 |
| 3149 | 3.87 | 255.2325 | [M-H]^-^ | FA 16:0 |
| 3150 | 2.27 | 255.2325 | [M-H]^-^ | FA 16:0 |
| 3152 | 2.07 | 255.2334 | [M-H]^-^ | FA 16:0 |
| 3191 | 0.77 | 257.1757 | [M-H]^-^ | FA 14:1 |
| 3396 | 2.09 | 271.2281 | [M-H]^-^ | FA 16:0(2OH) |
| 3436 | 0.93 | 275.2021 | [M-H]^-^ | FA 18:4 |
| 3438 | 11.31 | 275.2023 | [M-H]^-^ | FA 18:4 |
| 3468 | 1.32 | 277.2171 | [M-H]^-^ | FA 18:3 |
| 3469 | 2.04 | 277.2172 | [M-H]^-^ | FA 18:3 |
| 3507 | 0.84 | 279.1968 | [M-H]^-^ | FA 17:3 |
| 3512 | 2.77 | 279.2333 | [M-H]^-^ | FA 18:2 |
| 3536 | 4.02 | 281.2482 | [M-H]^-^ | FA 18:1 |
| 3560 | 5.48 | 283.2644 | [M-H]^-^ | FA 18:0 |
| 3655 | 0.88 | 289.1812 | [M-H]^-^ | FA 18:5 |
| 3684 | 11.70 | 291.1967 | [M-H]^-^ | FA 18:4 |
| 3685 | 0.94 | 291.1968 | [M-H]^-^ | FA 18:4 |
| 3686 | 1.30 | 291.2330 | [M-H]^-^ | FA 19:3 |
| 3762 | 0.92 | 293.2125 | [M-H] ^-^ | FA 18:3 |
| 3763 | 1.27 | 293.2125 | [M-H]^-^ | FA 18:3 |
| 3764 | 11.30 | 293.2127 | [M-H]^-^ | FA 18:3 |
| 3765 | 10.18 | 293.2127 | [M-H]^-^ | FA 18:3 |
| 3767 | 1.09 | 293.2128 | [M-H]^-^ | FA 18:3 |
| 3768 | 0.77 | 293.2129 | [M-H]^-^ | FA 18:3 |
| 3810 | 1.27 | 295.2279 | [M-H]^-^ | FA 18:2 |
| 3811 | 11.96 | 295.2279 | [M-H]^-^ | FA 18:2 |
| 3812 | 1.07 | 295.2280 | [M-H]^-^ | FA 18:2 |
| 3849 | 1.13 | 297.2432 | [M-H] ^-^ | FA 18:1 |
| 3855 | 1.28 | 297.2435 | [M-H]^-^ | FA 18:1 |
| 3925 | 1.84 | 301.2168 | [M-H]^-^ | FA 20:5 |
| 3926 | 2.37 | 301.2178 | [M-H]^-^ | FA 20:5 |
| 3928 | 1.98 | 301.2179 | [M-H]^-^ | FA 20:5 |
| 4069 | 9.70 | 309.2075 | [M-H]^-^ | FA 18:3 |
| 4070 | 9.54 | 309.2075 | [M-H]^-^ | FA 18:3 |
| 4071 | 10.56 | 309.2075 | [M-H]^-^ | FA 18:3 |
| 4072 | 0.78 | 309.2075 | [M-H]^-^ | FA 18:3 |
| 4073 | 10.43 | 309.2075 | [M-H]^-^ | FA 18:3 |
| 4076 | 10.25 | 309.2076 | [M-H]^-^ | FA 18:3 |
| 4077 | 1.20 | 309.2437 | [M-H]^-^ | FA 19:2 |
| 4167 | 0.84 | 311.2223 | [M-H]^-^ | FA 18:2 |
| 4168 | 1.04 | 311.2224 | [M-H]^-^ | FA 18:2 |
| 4169 | 11.38 | 311.2224 | [M-H]^-^ | FA 18:2 |
| 4171 | 10.97 | 311.2236 | [M-H]^-^ | FA 18:2 |
| 4199 | 0.77 | 313.2383 | [M-H] ^-^ | FA 18:1 |
| 4235 | 1.02 | 315.2541 | [M-H]^-^ | FA 18:0 |
| 4236 | 11.17 | 315.2542 | [M-H]^-^ | FA 18:0 |
| 4238 | 11.37 | 315.2543 | [M-H]^-^ | FA 18:0 |
| 4353 | 1.13 | 321.2438 | [M-H]^-^ | FA 20:3 |
| 4396 | 1.33 | 323.2592 | [M-H]^-^ | FA 20:2 |

**Table S3.** Annotated compound based on MS/MS in organic phase negative ionization mode data. (cont.)

| **ID** | **RT (min)** | ***m/z*** | **Type** | **Metabolite name** |
| --- | --- | --- | --- | --- |
| 4462 | 0.67 | 325.2015 | [M-H]^-^ | FA 18:3 |
| 4464 | 9.35 | 325.2029 | [M-H]^-^ | FA 18:3 |
| 4494 | 0.67 | 327.2176 | [M-H]^-^ | FA 18:2 |
| 4496 | 9.06 | 327.2177 | [M-H]^-^ | FA 18:2 |
| 4497 | 9.65 | 327.2177 | [M-H]^-^ | FA 18:2 |
| 4526 | 0.69 | 329.2330 | [M-H]^-^ | FA 18:1 |
| 4527 | 9.31 | 329.2342 | [M-H]^-^ | FA 18:1 |
| 4820 | 0.64 | 343.2130 | [M-H]^-^ | FA 18:2 |
| 4969 | 1.81 | 351.2903 | [M-H]^-^ | FA 22:2 |
| 5525 | 1.02 | 381.2642 | [M-H]^-^ | FA 22:3 |
| 5556 | 8.26 | 383.3525 | [M-H]^-^ | FA 24:0(2OH) |
| 6883 | 0.86 | 446.3116 | [M+HCOO]^-^ | Cer 22:1 |
| 7043 | 1.81 | 452.2784 | [M-H]^-^ | LPE 16:0 |
| 7045 | 1.67 | 452.2784 | [M-H]^-^ | LPE 16:0 |
| 7046 | 12.26 | 452.2786 | [M-H]^-^ | LPE 16:0 |
| 7359 | 3.92 | 465.2989 | [M-H]^-^ | ST 27:1 |
| 7482 | 3.41 | 469.3161 | [M-H]^-^ | NAGlySer 20:1 |
| 7594 | 1.12 | 474.2638 | [M-H]^-^ | LPE 18:3 |
| 7637 | 1.41 | 476.2787 | [M-H]^-^ | LPE 18:2 |
| 7638 | 11.67 | 476.2787 | [M-H]^-^ | LPE 18:2 |
| 7723 | 2.85 | 480.3094 | [M-H]^-^ | LPE 18:0 |
| 7749 | 1.30 | 481.2570 | [M-H]^-^ | LPG 16:1 |
| 7750 | 11.70 | 481.2574 | [M-H]^-^ | LPG 16:1 |
| 7751 | 1.23 | 481.2574 | [M-H]^-^ | LPG 16:1 |
| 7788 | 1.36 | 483.2737 | [M-H]^-^ | LPG 16:0 |
| 7789 | 1.47 | 483.2739 | [M-H]^-^ | LPG 16:0 |
| 7790 | 12.12 | 483.2740 | [M-H]^-^ | LPG 16:0 |
| 7793 | 4.24 | 483.3317 | [M-H]^-^ | NAGlySer 21:1 |
| 7977 | 2.50 | 492.3102 | [M-H]^-^ | LPE 19:1 |
| 8143 | 2.95 | 499.3633 | [M-H]^-^ | NAGlySer 22:0 |
| 8295 | 1.17 | 507.2728 | [M-H]^-^ | LPG 18:2 |
| 8296 | 11.49 | 507.2733 | [M-H]^-^ | LPG 18:2 |
| 8315 | 4.48 | 508.3399 | [M-H]^-^ | LPE 20:0 |
| 8326 | 1.53 | 509.2891 | [M-H]^-^ | LPG 18:1 |
| 8327 | 12.51 | 509.2895 | [M-H]^-^ | LPG 18:1 |
| 8637 | 1.98 | 523.3409 | [M-H]^-^ | LPG O-20:1 |
| 8982 | 1.56 | 540.3298 | [M+HCOO]^-^ | LPC 16:0 |
| 8983 | 1.70 | 540.3300 | [M+HCOO]^-^ | LPC 16:0 |
| 8984 | 12.01 | 540.3301 | [M+HCOO]^-^ | LPC 16:0 |
| 8985 | 12.33 | 540.3303 | [M+HCOO]^-^ | LPC 16:0 |
| 9402 | 1.07 | 562.3140 | [M+HCOO]^-^ | LPC 18:3 |
| 9403 | 10.97 | 562.3140 | [M+HCOO]^-^ | LPC 18:3 |
| 9404 | 11.12 | 562.3143 | [M+HCOO]^-^ | LPC 18:3 |
| 9433 | 1.33 | 564.3303 | [M+HCOO]^-^ | LPC 18:2 |
| 9434 | 1.25 | 564.3303 | [M+HCOO]^-^ | LPC 18:2 |
| 9436 | 11.72 | 564.3308 | [M+HCOO]^-^ | LPC 18:2 |
| 9473 | 1.65 | 566.3448 | [M+HCOO]^-^ | LPC 18:1 |
| 9474 | 1.78 | 566.3448 | [M+HCOO]^-^ | LPC 18:1 |
| 9521 | 2.65 | 568.3610 | [M+HCOO]^-^ | LPC 18:0 |
| 9573 | 1.37 | 571.2901 | [M-H]^-^ | LPI 16:0 |
| 9574 | 11.37 | 571.2902 | [M-H]^-^ | LPI 16:0 |
| 9744 | 2.34 | 580.3629 | [M+HCOO]^-^ | LPC 19:1 |

**Table S3.** Annotated compounds based on MS/MS in organic phase negative ionization mode data. (cont.)

| **ID** | **RT (min)** | ***m/z*** | **Type** | **Metabolite name** |
| --- | --- | --- | --- | --- |
| 9747 | 14.13 | 580.3633 | [M+HCOO]^-^ | LPC 19:1 |
| 9995 | 10.53 | 593.2731 | [M-H]^-^ | LPI 18:3 |
| 9996 | 10.39 | 593.2733 | [M-H]^-^ | LPI 18:3 |
| 9997 | 0.92 | 593.2735 | [M-H]^-^ | LPI 18:3 |
| 10026 | 10.97 | 595.2898 | [M-H]^-^ | LPI 18:2 |
| 10028 | 1.11 | 595.2901 | [M-H]^-^ | LPI 18:2 |
| 10047 | 4.24 | 596.3939 | [M+HCOO]^-^ | LPC 20:0 |
| 10819 | 9.06 | 645.4498 | [M-H]^-^ | PA 32:1\|PA 16:0_16:1 |
| 10852 | 9.32 | 647.4651 | [M-H]^-^ | PA 32:0\|PA 16:0_16:0 |
| 10961 | 4.58 | 655.3905 | [M-H]^-^ | PI O-22:0 |
| 11097 | 4.90 | 663.4495 | [M+HCOO]^-^ | ASG 28:1 |
| 11176 | 8.13 | 667.4345 | [M-H]^-^ | PA 34:4\|PA 16:1_18:3 |
| 11194 | 8.36 | 669.4511 | [M-H]^-^ | PA 34:3\|PA 16:0_18:3 |
| 11195 | 8.56 | 669.4511 | [M-H]^-^ | PA 34:3\|PA 16:0_18:3 |
| 11232 | 8.81 | 671.4644 | [M-H]^-^ | PA 34:2\|PA 16:0_18:2 |
| 11233 | 9.07 | 671.4645 | [M-H]^-^ | PA 34:2\|PA 16:1_18:1 |
| 11264 | 9.34 | 673.4825 | [M-H]^-^ | PA 34:1\|PA 16:0_18:1 |
| 11428 | 8.66 | 683.4647 | [M-H]^-^ | PA 35:3\|PA 17:0_18:3 |
| 11429 | 8.37 | 683.4650 | [M-H]^-^ | PMeOH 34:3\|PMeOH 16:0_18:3 |
| 11483 | 9.80 | 687.4993 | [M-H]^-^ | PA 35:1\|PA 16:0_19:1 |
| 11552 | 6.44 | 691.4368 | [M-H]^-^ | PA 36:6\|PA 18:3_18:3 |
| 11579 | 7.77 | 693.4498 | [M-H]^-^ | PA 36:5\|PA 18:2_18:3 |
| 11610 | 8.36 | 695.4639 | [M-H]^-^ | PA 36:4\|PA 18:2_18:2 |
| 11647 | 8.99 | 697.4814 | [M-H]^-^ | PA 36:3\|PA 18:0_18:3 |
| 11648 | 8.83 | 697.4818 | [M-H]^-^ | PA 36:3\|PA 18:1_18:2 |
| 11687 | 9.46 | 699.4953 | [M-H]^-^ | PA 36:2\|PA 18:0_18:2 |
| 11688 | 9.35 | 699.4962 | [M-H]^-^ | PA 36:2\|PA 18:1_18:1 |
| 11713 | 10.00 | 701.5138 | [M-H]^-^ | PA 36:1\|PA 18:0_18:1 |
| 11817 | 8.80 | 709.4813 | [M-H]^-^ | PA 37:4\|PA 19:1_18:3 |
| 11842 | 9.26 | 711.4977 | [M-H]^-^ | PA 37:3\|PA 19:1_18:2 |
| 11853 | 8.66 | 712.4933 | [M-H]^-^ | PE 34:3\|PE 16:0_18:3 |
| 11854 | 8.42 | 712.5311 | [M-H]^-^ | PE O-35:3\|PE O-17:0_18:3 |
| 11882 | 9.82 | 713.5138 | [M-H]^-^ | PA 37:2\|PA 18:1_19:1 |
| 11891 | 9.12 | 714.5093 | [M-H]^-^ | PE 34:2\|PE 16:0_18:2 |
| 11957 | 8.81 | 719.4855 | [M-H]^-^ | PG 32:1\|PG 16:0_16:1 |
| 11994 | 9.03 | 721.5035 | [M-H]^-^ | PG 32:0\|PG 16:0_16:0 |
| 12064 | 9.66 | 725.5141 | [M-H]^-^ | PA 38:3\|PA 20:0_18:3 |
| 12069 | 10.59 | 726.6273 | [M+HCOO]^-^ | Cer 42:1 |
| 12102 | 10.08 | 727.5291 | [M-H]^-^ | PA 38:2\|PA 20:0_18:2 |
| 12116 | 10.70 | 728.6428 | [M+HCOO]^-^ | Cer 42:0 |
| 12136 | 10.38 | 729.5436 | [M-H]^-^ | PA 38:1\|PA 20:0_18:1 |
| 12223 | 8.15 | 736.4907 | [M-H]^-^ | PE 36:5\|PE 18:2_18:3 |
| 12241 | 8.37 | 737.4395 | [M-H]^-^ | SMGDG 28:1 |
| 12257 | 8.62 | 738.5092 | [M-H]^-^ | PE 36:4\|PE 18:2_18:2 |
| 12258 | 7.05 | 738.5094 | [M-2H]^2-^ | CL 74:7\|CL 18:1_18:1_18:2_20:3 |
| 12329 | 9.34 | 741.4695 | [M-2H]^2-^ | CL 75:11\|CL 18:1_18:2_18:3_21:5 |
| 12331 | 7.84 | 741.4733 | [M-H]^-^ | PG 34:4\|PG 16:1_18:3 |
| 12356 | 8.35 | 743.4891 | [M-H]^-^ | PG 34:3\|PG 16:1_18:2 |
| 12357 | 8.13 | 743.4892 | [M-H]^-^ | PG 34:3\|PG 16:0_18:3 |
| 12358 | 9.21 | 743.5317 | [M-2H]^2-^ | CL 74:2\|CL 16:0_16:0_22:0_20:2 |
| 12386 | 8.56 | 745.5022 | [M-H]^-^ | PG 34:2\|PG 16:0_18:2 |
| 12387 | 8.83 | 745.5027 | [M-H]^-^ | PG 34:2\|PG 16:1_18:1 |

**Table S3.** Annotated compounds based on MS/MS in organic phase negative ionization mode data. (cont.)

| **ID** | **RT (min)** | ***m/z*** | **Type** | **Metabolite name** |
| --- | --- | --- | --- | --- |
| 12406 | 9.04 | 74.5189 | [M-H]^-^ | PG 34:1\|PG 16:0_18:1 |
| 12407 | 9.44 | 747.5192 | [M-H]^-^ | PG 34:1\|PG 18:0_16:1 |
| 12431 | 9.03 | 749.5144 | [M-H]^-^ | PA 40:5\|PA 22:2_18:3 |
| 12457 | 9.51 | 751.5286 | [M-H]^-^ | PA 40:4\|PA 18:2_22:2 |
| 12572 | 8.42 | 758.5451 | [M+HCOO]^-^ | HexCer 34:2 |
| 12589 | 9.24 | 759.5167 | [M-H]^-^ | PG 35:2\|PG 16:1_19:1 |
| 12625 | 9.48 | 761.5360 | [M-H]^-^ | PG 35:1\|PG 16:0_19:1 |
| 12665 | 8.18 | 765.4841 | [M-H]^-^ | PG 36:6\|PG 18:3_18:3 |
| 12666 | 8.31 | 765.5138 | [M-2H]^2-^ | CL 78:8\|CL 16:0_24:0_18:3_20:5 |
| 12702 | 8.73 | 767.5313 | [M-2H] ^2-^ | CL 78:6\|CL 24:0_18:2_18:2_18:2 |
| 12716 | 9.97 | 768.5571 | [M-H]^-^ | PE 38:3\|PE 20:0_18:3 |
| 12861 | 9.49 | 778.5611 | [M+HCOO]^-^ | PC 32:0\|PC 16:0_16:0 |
| 12971 | 9.10 | 786.5721 | [M+HCOO]^-^ | HexCer 36:2 |
| 13027 | 8.58 | 791.4965 | [M-2H] ^2-^ | CL 83:17\|CL 17:2_22:5_22:5_22:5 |
| 13115 | 9.85 | 796.6283 | [M-H]^-^ | SL 47:2 |
| 13120 | 9.86 | 796.7081 | [M+CH_3_COO]^-^ | Cer 46:1 |
| 13127 | 8.86 | 797.5407 | [M-H]^-^ | PG 38:4\|PG 20:1_18:3 |
| 13169 | 8.51 | 800.5454 | [M+HCOO]^-^ | PC 34:3\|PC 16:0_18:3 |
| 13197 | 8.96 | 802.5582 | [M+HCOO]^-^ | PC 34:2\|PC 16:0_18:2 |
| 13216 | 9.51 | 804.5784 | [M+HCOO]^-^ | PC O-34:2 |
| 13284 | 8.92 | 809.5207 | [M-H]^-^ | PI 32:0\|PI 16:0_16:0 |
| 13286 | 8.68 | 809.5419 | [M-H]^-^ | SMGDG O-33:0\|SMGDG O-17:0_16:0 |
| 13366 | 9.81 | 814.6078 | [M+HCOO]^-^ | HexCer 38:2 |
| 13468 | 6.79 | 822.5275 | [M+HCOO]^-^ | PC 36:6\|PC 18:3_18:3 |
| 13497 | 7.97 | 824.5448 | [M+HCOO]^-^ | PC 36:5\|PC 18:2_18:3 |
| 13524 | 8.41 | 826.5309 | [M+HCOO]^-^ | PC 35:5 |
| 13525 | 8.49 | 826.5587 | [M+HCOO]^-^ | PC 36:4\|PC 18:2_18:2 |
| 13559 | 8.97 | 828.5750 | [M+HCOO]^-^ | PC 36:3\|PC 18:1_18:2 |
| 13560 | 9.14 | 828.5753 | [M+HCOO]^-^ | PC 36:3\|PC 18:0_18:3 |
| 13596 | 8.00 | 831.4211 | [M-H]^-^ | SMGDG 36:10 |
| 13610 | 8.00 | 831.5005 | [M-H]^-^ | PI 34:3\|PI 16:0_18:3 |
| 13626 | 10.11 | 832.6057 | [M+HCOO]^-^ | PC 36:1\|PC 18:0_18:1 |
| 13652 | 8.47 | 833.5169 | [M-H]^-^ | PI 34:2\|PI 16:0_18:2 |
| 13678 | 8.95 | 835.5352 | [M-H]^-^ | PI 34:1\|PI 16:0_18:1 |
| 13679 | 9.08 | 835.5627 | [M-H]^-^ | SMGDG O-35:1\|SMGDG O-17:0_18:1 |
| 13729 | 8.95 | 840.5752 | [M+HCOO]^-^ | PC 37:4\|PC 19:1_18:3 |
| 13753 | 9.42 | 842.5901 | [M+HCOO]^-^ | PC 37:3\|PC 19:1_18:2 |
| 13757 | 9.85 | 842.6371 | [M-H]^-^ | SL 51:7 |
| 13758 | 10.45 | 842.6703 | [M-H]^-^ | HexCer 42:1 |
| 13779 | 9.96 | 844.6068 | [M+HCOO]^-^ | PC 37:2\|PC 18:1_19:1 |
| 13785 | 9.91 | 844.6528 | [M+HCOO]^-^ | PC O-38:2\|PC O-20:1_18:1 |
| 13913 | 5.66 | 853.4886 | [M-H]^-^ | PI 36:6\|PI 18:3_18:3 |
| 13965 | 8.65 | 859.5343 | [M-H]^-^ | PI 36:3\|PI 18:0_18:3 |
| 13977 | 10.14 | 860.6469 | [M+HCOO]^-^ | HexCer 40:1 |
| 14087 | 10.54 | 870.6674 | [M+HCOO]^-^ | HexCer 42:2 |
| 14130 | 10.32 | 874.6654 | [M+HCOO]^-^ | HexCer 41:1 |
| 14290 | 10.45 | 888.6777 | [M+HCOO]^-^ | HexCer 42:1 |
| 14453 | 10.56 | 902.6935 | [M+HCOO]^-^ | HexCer 43:1 |
| 14608 | 10.65 | 916.7083 | [M+HCOO]^-^ | HexCer 44:1 |
| 15066 | 8.01 | 967.4775 | [M-H]^-^ | PI 40:9 |

| **Acronym** | **Lipid subclass** |
| --- | --- |
| **CAR** | Carnitine |
| **LPE** | Lysophosphatidylethanolamine |
| **PC** | Phosphatidylcholine |
| **SMGDG** | Semino lipid |
| **CL** | Cardiolipin |
| **LPG** | Lysophosphatidylglycerol |
| **PE** | Phosphatidylethanolamine |
| **ST** | Sterol Lipids |
| **DG** | Diacylglycerol |
| **MG** | Monoacylglycerol |
| **PG** | Phosphatidylglycerol |
| **TG** | Triacylglycerol |
| **FA** | Free fatty acid |
| **NAE** | N-acyl ethanolamine |
| **PI** | Phosphatidylinositol |
| **HexCer** | Hexosylceramide |
| **NAGlySer** | N-acyl glycyl serine |
| **PMeOH** | Phosphatidylmethanol |
| **LPC** | Lysophosphatidylcholine |
| **PA** | Phosphatidic acid |
| **SE** | Sterol ester |

**Table S4.** Annotated compounds based on MS/MS in organic phase positive ionization mode data.

| **ID** | **RT (min)** | ***m/z*** | **Type** | **Metabolite name** |
| --- | --- | --- | --- | --- |
| 2874 | 0.61 | 202.1803 | [M+H]^+^ | NAE 9:0 |
| 3319 | 0.67 | 216.1961 | [M+H]^+^ | NAE 10:0 |
| 4638 | 0.81 | 256.2267 | [M+H]^+^ | NAE 13:1 |
| 4639 | 2.60 | 256.2267 | [M+H]^+^ | NAE 13:1 |
| 4640 | 2.88 | 256.2267 | [M+H]^+^ | NAE 13:1 |
| 4641 | 4.13 | 256.2267 | [M+H]^+^ | NAE 13:1 |
| 4643 | 4.51 | 256.2268 | [M+H]^+^ | NAE 13:1 |
| 4700 | 1.30 | 258.2424 | [M+H]^+^ | NAE 13:0 |
| 5047 | 2.73 | 270.2426 | [M+H]^+^ | NAE 14:1 |
| 5101 | 1.00 | 272.2579 | [M+H]^+^ | NAE 14:0 |
| 5570 | 0.67 | 288.2162 | [M+H]^+^ | CAR 8:0 |
| 5797 | 0.85 | 296.2560 | [M+H]^+^ | NAE 16:2 |
| 6611 | 1.37 | 322.2733 | [M+H]^+^ | NAE 18:3 |
| 6728 | 1.76 | 324.2888 | [M+H]^+^ | NAE 18:2 |
| 6729 | 1.24 | 324.2900 | [M+H]^+^ | NAE 18:2 |
| 6811 | 2.45 | 326.3047 | [M+H]^+^ | NAE 18:1 |
| 6812 | 1.49 | 326.3047 | [M+H]^+^ | NAE 18:1 |
| 6852 | 0.78 | 328.2478 | [M+NH_4_]^+^ | MG 15:3 |
| 6894 | 0.84 | 330.2638 | [M+H]^+^ | CAR 11:0 |
| 7017 | 0.85 | 334.2744 | [M+H]^+^ | NAE 19:4 |
| 7190 | 1.71 | 340.3203 | [M+H]^+^ | NAE 19:1 |
| 7366 | 1.76 | 346.2719 | [M+H]^+^ | NAE 20:5 |
| 7422 | 1.18 | 348.2900 | [M+H]^+^ | NAE 20:4 |
| 7423 | 0.97 | 348.2900 | [M+H]^+^ | NAE 20:4 |
| 7424 | 2.82 | 348.3104 | [M+NH_4_]^+^ | MG 16:0 |
| 7489 | 0.94 | 350.3057 | [M+H]^+^ | NAE 20:3 |
| 7490 | 1.36 | 350.3057 | [M+H]^+^ | NAE 20:3 |
| 7564 | 2.02 | 352.3214 | [M+H]^+^ | NAE 20:2 |
| 7565 | 1.69 | 352.3215 | [M+H]^+^ | NAE 20:2 |
| 7567 | 0.91 | 352.3216 | [M+H]^+^ | NAE 20:2 |
| 7568 | 1.16 | 352.3216 | [M+H]^+^ | NAE 20:2 |
| 7621 | 2.31 | 354.3359 | [M+H]^+^ | NAE 20:1 |
| 7622 | 2.04 | 354.3359 | [M+H]^+^ | NAE 20:1 |
| 7679 | 0.79 | 356.2788 | [M+H]^+^ | CAR 13:1 |
| 7909 | 1.10 | 364.3205 | [M+H]^+^ | NAE 21:3 |
| 7996 | 0.83 | 367.2473 | [M+Na]^+^ | DG 16:0 |
| 8025 | 1.40 | 368.2790 | [M+NH_4_]^+^ | MG 18:4 |
| 8026 | 0.78 | 368.2791 | [M+NH_4_]^+^ | MG 18:4 |
| 8098 | 1.58 | 370.2951 | [M+NH_4_]^+^ | MG 18:3 |
| 8149 | 1.37 | 372.2866 | [M+H]^+^ | NAE 22:6 |
| 8214 | 1.69 | 374.3029 | [M+H]^+^ | NAE 22:5 |
| 8215 | 1.33 | 374.3034 | [M+H]^+^ | NAE 22:5 |
| 8272 | 2.29 | 376.3185 | [M+H]^+^ | NAE 22:4 |
| 8273 | 2.08 | 376.3187 | [M+H]^+^ | NAE 22:4 |
| 8274 | 1.63 | 376.3187 | [M+H]^+^ | NAE 22:4 |
| 8276 | 4.48 | 376.3414 | [M+NH_4_]^+^ | MG 18:0 |
| 8399 | 2.37 | 380.3516 | [M+H]^+^ | NAE 22:2 |
| 8456 | 3.47 | 382.3685 | [M+H]^+^ | NAE 22:1 |
| 8513 | 0.94 | 384.3102 | [M+NH_4_]^+^ | MG 19:3 |
| 8582 | 0.95 | 386.3263 | [M+H]^+^ | CAR 15:0 |
| 8903 | 2.30 | 395.3627 | [M+H-H_2_O]^+^ | ST 29:2;O |
| 8904 | 1.37 | 395.3629 | [M+H-H_2_O]^+^ | ST 29:2;O |

**Table S4.** Annotated compounds based on MS/MS in organic phase positive ionization mode data. (cont.)

| **ID** | **RT (min)** | ***m/z*** | **Type** | **Metabolite name** |
| --- | --- | --- | --- | --- |
| 8906 | 10.81 | 395.3666 | [M+H-H_2_O]^+^ | ST 29:2;O |
| 8907 | 10.85 | 395.3667 | [M+H-H_2_O]^+^ | ST 29:2;O |
| 8977 | 1.33 | 397.3782 | [M+H-H_2_O]^+^ | ST 29:1;O |
| 8982 | 10.90 | 397.3822 | [M+H-H_2_O]^+^ | ST 29:1;O |
| 9460 | 8.53 | 413.3778 | [M+H]^+^ | ST 29:2;O |
| 10246 | 0.90 | 437.3744 | [M+Na]^+^ | ST 29:1;O |
| 10733 | 1.82 | 454.2922 | [M+H]^+^ | LPE 16:0 |
| 11095 | 0.80 | 465.3484 | [M+Na]^+^ | DG 23:0 |
| 11453 | 1.13 | 476.2763 | [M+H]^+^ | LPE 18:3 |
| 11492 | 1.41 | 478.2936 | [M+H]^+^ | LPE 18:2 |
| 11529 | 4.65 | 479.3723 | [M+Na]^+^ | DG 24:0 |
| 11602 | 2.86 | 482.3228 | [M+H]^+^ | LPE 18:0 |
| 11770 | 5.80 | 488.3955 | [M+NH_4_]^+^ | TG 24:0\|TG 8:0_8:0_8:0 |
| 11972 | 2.52 | 494.3231 | [M+H]^+^ | LPE 19:1 |
| 12016 | 1.71 | 496.3385 | [M+H]^+^ | LPC 16:0/0:0 |
| 12467 | 4.51 | 510.3536 | [M+H]^+^ | LPE 20:0 |
| 12468 | 2.12 | 510.3560 | [M+H]^+^ | LPC 17:0/0:0 |
| 12469 | 2.06 | 510.3644 | [M+H]^+^ | LPC 17:0/0:0 |
| 12613 | 1.00 | 516.3082 | [M+H]^+^ | LPC 18:4/0:0 |
| 12676 | 1.71 | 518.3222 | [M+Na]^+^ | LPC 16:0 |
| 12677 | 1.08 | 518.3253 | [M+H]^+^ | LPC 18:3/0:0 |
| 12733 | 1.34 | 520.3403 | [M+H]^+^ | LPC 18:2/0:0 |
| 12786 | 1.65 | 522.3555 | [M+H]^+^ | LPC 18:1 |
| 12787 | 1.79 | 522.3558 | [M+H]^+^ | LPC 18:1/0:0 |
| 12841 | 2.67 | 524.3695 | [M+H]^+^ | LPC 18:0/0:0 |
| 13143 | 2.35 | 536.3710 | [M+H]^+^ | LPC 19:1/0:0 |
| 13144 | 2.14 | 536.3711 | [M+H]^+^ | LPC 19:1 |
| 13552 | 4.27 | 552.4028 | [M+H]^+^ | LPC 20:0/0:0 |
| 14496 | 10.35 | 586.5402 | [M+NH_4_]^+^ | DG 32:0\|DG 16:0_16:0 |
| 14569 | 5.04 | 589.4816 | [M+Na]^+^ | DG 32:1 |
| 14633 | 10.33 | 591.4961 | [M+Na]^+^ | DG 32:0 |
| 15002 | 6.57 | 603.4900 | [M+Na]^+^ | DG 33:1 |
| 15146 | 9.53 | 608.5237 | [M+NH_4_]^+^ | DG 34:3\|DG 16:0_18:3 |
| 15188 | 10.01 | 610.5401 | [M+NH_4_]^+^ | DG 34:2\|DG 16:0_18:2 |
| 15211 | 3.81 | 611.4673 | [M+Na]^+^ | DG 34:4 |
| 15213 | 3.88 | 611.4675 | [M+Na]^+^ | DG 34:4 |
| 15256 | 10.36 | 612.5552 | [M+NH_4_]^+^ | DG 34:1\|DG 16:0_18:1 |
| 15274 | 9.53 | 613.4805 | [M+Na]^+^ | DG 34:3 |
| 15275 | 7.86 | 613.4836 | [M+Na]^+^ | DG 34:3 |
| 15301 | 10.60 | 614.5700 | [M+NH_4_]^+^ | DG 34:0\|DG 16:0_18:0 |
| 15312 | 10.01 | 615.4960 | [M+Na]^+^ | DG 34:2 |
| 15383 | 6.13 | 617.5033 | [M+Na]^+^ | DG 34:1 |
| 15384 | 6.09 | 617.5034 | [M+Na]^+^ | DG 34:1 |
| 15385 | 5.98 | 617.5035 | [M+Na]^+^ | DG 34:1 |
| 15386 | 5.11 | 617.5040 | [M+Na]^+^ | DG 34:1 |
| 15447 | 10.59 | 619.5276 | [M+Na]^+^ | DG 34:0 |
| 15659 | 8.29 | 628.4951 | [M+NH_4_]^+^ | DG 36:7\|DG 18:3_18:4 |
| 15661 | 10.38 | 628.5513 | [M+NH_4_]^+^ | TG 34:0\|TG 8:0_12:0_14:0 |
| 15725 | 8.52 | 630.5099 | [M+NH_4_]^+^ | DG 36:6\|DG 18:3_18:3 |
| 15772 | 8.97 | 632.5237 | [M+NH_4_]^+^ | DG 36:5\|DG 18:2_18:3 |
| 15795 | 8.29 | 633.4487 | [M+Na]^+^ | DG 36:7 |
| 15830 | 9.50 | 634.5391 | [M+NH_4_]^+^ | DG 36:4\|DG 18:2_18:2 |

**Table S4.** Annotated compounds based on MS/MS in organic phase positive ionization mode data. (cont.)

| **ID** | **RT (min)** | ***m/z*** | **Type** | **Metabolite name** |
| --- | --- | --- | --- | --- |
| 15845 | 8.53 | 635.4645 | [M+Na]^+^ | DG 36:6 |
| 15895 | 8.98 | 637.4798 | [M+Na]^+^ | DG 36:5 |
| 15932 | 10.37 | 638.5695 | [M+NH_4_]^+^ | DG 36:2\|DG 18:0_18:2 |
| 16061 | 10.78 | 642.6020 | [M+NH_4_]^+^ | DG 36:0\|DG 18:0_18:0 |
| 16174 | 10.78 | 647.5592 | [M+Na]^+^ | DG 36:0 |
| 16226 | 8.07 | 649.4789 | [M+Na]^+^ | DG 37:6 |
| 16428 | 10.61 | 656.5812 | [M+NH_4_]^+^ | TG 36:0\|TG 10:0_12:0_14:0 |
| 17123 | 10.45 | 682.6350 | [M+H]^+^ | Cer 42:1;O4\|Cer 18:1;O3/24:0(2OH) |
| 17170 | 10.79 | 684.6141 | [M+NH_4_]^+^ | TG 38:0\|TG 10:0_12:0_16:0 |
| 17284 | 10.79 | 689.5674 | [M+Na]^+^ | TG 38:0\|TG 10:0_12:0_16:0 |
| 17345 | 11.34 | 692.6325 | [M+NH_4_]^+^ | SE 29:1/18:3 |
| 17840 | 10.93 | 712.6460 | [M+NH_4_]^+^ | TG 40:0\|TG 10:0_14:0_16:0 |
| 17883 | 8.67 | 714.5038 | [M+H]^+^ | PE 34:3\|PE 16:0_18:3 |
| 17885 | 8.43 | 714.5492 | [M+H]^+^ | HexCer 34:2;O3\|HexCer 18:2;O2/16:0;O |
| 17936 | 9.13 | 716.5233 | [M+H]^+^ | PE 34:2\|PE 16:0_18:2 |
| 17976 | 10.93 | 717.5989 | [M+Na]^+^ | TG 40:0\|TG 12:0_12:0_16:0 |
| 18404 | 9.50 | 734.5689 | [M+H]^+^ | PC 32:0\|PC 16:0_16:0 |
| 18496 | 8.17 | 738.5087 | [M+H]^+^ | PE 36:5 |
| 18497 | 8.81 | 738.5269 | [M+NH_4_]^+^ | PG 32:1\|PG 16:0_16:1 |
| 18543 | 8.64 | 740.5205 | [M+H]^+^ | PE 36:4\|PE 18:2_18:2 |
| 18545 | 9.03 | 740.5448 | [M+NH_4_]^+^ | PG 32:0\|PG 16:0_16:0 |
| 18547 | 11.05 | 740.6734 | [M+NH_4_]^+^ | TG 42:0\|TG 12:0_14:0_16:0 |
| 18585 | 9.11 | 742.5846 | [M+H]^+^ | HexCer 36:2;O3\|HexCer 18:2;O2/18:0;O |
| 18668 | 8.66 | 745.5922 | [M+H]^+^ | SM 36:2;O2 |
| 18721 | 10.34 | 747.6050 | [M+Na]^+^ | DG 44:6 |
| 18778 | 9.96 | 749.6204 | [M+Na]^+^ | DG 44:5 |
| 18971 | 8.52 | 756.5552 | [M+H]^+^ | PC 34:3\|PC 16:0_18:3 |
| 19012 | 8.96 | 758.5678 | [M+H]^+^ | PC 34:2\|PC 16:0_18:2 |
| 19058 | 7.85 | 760.5130 | [M+NH_4_]^+^ | PG 34:4\|PG 16:1_18:3 |
| 19060 | 9.52 | 760.5842 | [M+H]^+^ | PC 34:1 |
| 19109 | 8.36 | 762.5275 | [M+NH_4_]^+^ | PG 34:3\|PG 16:1_18:2 |
| 19134 | 10.05 | 763.6364 | [M+Na]^+^ | DG 45:5 |
| 19162 | 8.83 | 764.5434 | [M+NH_4_]^+^ | PG 34:2\|PG 16:1_18:1 |
| 19220 | 9.05 | 766.5583 | [M+NH_4_]^+^ | PG 34:1\|PG 16:0_18:1 |
| 19226 | 11.03 | 766.6484 | [M+NH_4_]^+^ | TG 43:2;O\|TG 12:0_14:0_17:2;O |
| 19229 | 11.06 | 766.6882 | [M+NH_4_]^+^ | TG 44:1\|TG 12:0_14:0_18:1 |
| 19334 | 8.88 | 770.5802 | [M+H]^+^ | PC 35:3 |
| 19335 | 9.83 | 770.6154 | [M+H]^+^ | HexCer 38:2;O3\|HexCer 18:2;O2/20:0;O |
| 19369 | 11.04 | 771.6047 | [M+Na]^+^ | DG 46:8 |
| 19451 | 9.95 | 774.6026 | [M+H]^+^ | PC 35:1 |
| 19467 | 8.87 | 775.5344 | [M+Na]^+^ | DG 47:13 |
| 19507 | 9.35 | 777.5511 | [M+Na]^+^ | DG 47:12 |
| 19527 | 6.82 | 778.5383 | [M+H]^+^ | PC 36:6\|PC 18:3_18:3 |
| 19582 | 8.96 | 780.5511 | [M+Na]^+^ | PC 34:2 |
| 19583 | 7.99 | 780.5516 | [M+H]^+^ | PC 36:5 |
| 19650 | 8.51 | 782.5671 | [M+H]^+^ | PC 36:4\|PC 18:2_18:2 |
| 19651 | 9.52 | 782.5681 | [M+Na]^+^ | PC 34:1 |
| 19701 | 9.15 | 784.5856 | [M+H]^+^ | PC 36:3 |
| 19702 | 8.97 | 784.5858 | [M+H]^+^ | PC 36:3 |
| 19709 | 10.24 | 784.6649 | [M+H]^+^ | Cer 50:6;O4\|Cer 18:0;O3/32:6(2OH) |
| 19758 | 9.53 | 786.6011 | [M+H]^+^ | PC 36:2 |
| 19759 | 9.65 | 786.6013 | [M+H]^+^ | PC 36:2 |

**Table S4.** Annotated compounds based on MS/MS in organic phase positive ionization mode data. (cont.)

| **ID** | **RT (min)** | ***m/z*** | **Type** | **Metabolite name** |
| --- | --- | --- | --- | --- |
| 19819 | 10.12 | 788.6146 | [M+H]^+^ | PC 36:1 |
| 19976 | 11.16 | 794.7208 | [M+NH_4_]^+^ | TG 46:1\|TG 14:0_16:0_16:1 |
| 20013 | 8.95 | 796.5824 | [M+H]^+^ | PC 37:4 |
| 20020 | 11.26 | 796.7386 | [M+NH_4_]^+^ | TG 46:0\|TG 14:0_16:0_16:0 |
| 20051 | 9.43 | 798.6019 | [M+H]^+^ | PC 37:3\|PC 19:1_18:2 |
| 20056 | 9.86 | 798.6450 | [M+H]^+^ | PC O-38:3 |
| 20100 | 9.97 | 800.6144 | [M+H]^+^ | PC 37:2\|PC 17:1_20:1 |
| 20210 | 9.95 | 805.5887 | [M+Na]^+^ | DG 49:12 |
| 20461 | 9.33 | 815.5660 | [M+Na]^+^ | DG 50:14 |
| 20488 | 10.45 | 816.6484 | [M+H]^+^ | PC 38:1 |
| 20490 | 10.15 | 816.6550 | [M+H]^+^ | HexCer 40:1;O4\|HexCer 18:1;O3/22:0(2OH) |
| 20538 | 10.88 | 818.6843 | [M+NH_4_]^+^ | ASG 28:1;O;Hex;FA 16:0 |
| 20563 | 10.05 | 819.6052 | [M+Na]^+^ | DG 50:12 |
| 20586 | 11.17 | 820.7380 | [M+NH_4_]^+^ | TG 48:2\|TG 16:0_16:1_16:1 |
| 20766 | 8.93 | 828.5609 | [M+NH_4_]^+^ | PI 32:0 |
| 20798 | 5.34 | 829.5844 | [M+Na]^+^ | DG 51:14 |
| 20819 | 10.34 | 830.6696 | [M+H]^+^ | HexCer 41:1;O4\|HexCer 18:1;O3/23:0(2OH) |
| 20820 | 10.87 | 830.6848 | [M+NH_4_]^+^ | ASG 29:2;O;Hex;FA 16:0 |
| 20868 | 10.91 | 832.7050 | [M+NH_4_]^+^ | ASG 29:1;O;Hex;FA 16:0 |
| 21158 | 10.45 | 844.6847 | [M+H]^+^ | HexCer 42:1;O4\|HexCer 18:1;O3/24:0(2OH) |
| 21256 | 11.27 | 848.7686 | [M+NH_4_]^+^ | TG 50:2\|TG 16:0_16:1_18:1 |
| 21301 | 8.01 | 850.5416 | [M+NH_4_]^+^ | PI 34:3 |
| 21339 | 8.48 | 852.5576 | [M+NH_4_]^+^ | PI 34:2 |
| 21345 | 10.57 | 852.6702 | [M+NH_4_]^+^ | ASG 29:2;O;Hex;FA 18:3 |
| 21373 | 8.96 | 854.5738 | [M+NH_4_]^+^ | PI 34:1 |
| 21376 | 10.63 | 854.6865 | [M+NH_4_]^+^ | ASG 29:1;O;Hex;FA 18:3 |
| 21390 | 8.02 | 855.4987 | [M+Na]^+^ | PI 34:3 |
| 21415 | 10.77 | 856.7042 | [M+NH_4_]^+^ | ASG 29:1;O;Hex;FA 18:2 |
| 21458 | 10.56 | 858.7006 | [M+H]^+^ | HexCer 43:1;O4\|HexCer 18:1;O3/25:0(2OH) |
| 21459 | 10.90 | 858.7180 | [M+NH_4_]^+^ | ASG 29:1;O;Hex;FA 18:1 |
| 21663 | 10.99 | 868.7411 | [M+NH_4_]^+^ | TG 52:6\|TG 16:0_18:3_18:3 |
| 21704 | 10.92 | 870.7181 | [M+NH_4_]^+^ | TG 51:6;O\|TG 16:0_18:2_17:4;O |
| 21706 | 11.08 | 870.7546 | [M+NH_4_]^+^ | TG 52:5\|TG 16:0_18:2_18:3 |
| 21739 | 5.68 | 872.5309 | [M+NH_4_]^+^ | PI 36:6 |
| 21742 | 10.65 | 872.7198 | [M+H]^+^ | HexCer 44:1;O4\|HexCer 18:1;O3/26:0(2OH) |
| 21743 | 10.99 | 872.7327 | [M+NH_4_]^+^ | ASG 29:1;O;Hex;FA 19:1 |
| 21744 | 11.17 | 872.7691 | [M+NH_4_]^+^ | TG 52:4\|TG 16:0_18:2_18:2 |
| 21787 | 11.26 | 874.7845 | [M+NH_4_]^+^ | TG 52:3\|TG 16:0_18:1_18:2 |
| 21805 | 11.08 | 875.7119 | [M+Na]^+^ | TG 52:5\|TG 16:0_18:2_18:3 |
| 21833 | 11.36 | 876.8013 | [M+NH_4_]^+^ | TG 52:2\|TG 16:0_18:1_18:1 |
| 22008 | 10.45 | 884.7330 | [M+NH_4_]^+^ | TG 52:6;O\|TG 16:0_18:3_18:3;O |
| 22085 | 10.70 | 888.7096 | [M+NH_4_]^+^ | TG 54:10\|TG 18:3_18:3_18:4 |
| 22131 | 10.77 | 890.7244 | [M+NH_4_]^+^ | TG 54:9\|TG 18:2_18:3_18:4 |
| 22167 | 10.88 | 892.7386 | [M+NH_4_]^+^ | TG 54:8\|TG 18:2_18:3_18:3 |
| 22188 | 10.70 | 893.6631 | [M+Na]^+^ | TG 54:10\|TG 18:3_18:3_18:4 |
| 22207 | 10.97 | 894.7534 | [M+NH_4_]^+^ | TG 54:7\|TG 18:2_18:2_18:3 |
| 22225 | 10.77 | 895.6801 | [M+Na]^+^ | TG 54:9\|TG 18:3_18:3_18:3 |
| 22251 | 11.07 | 896.7687 | [M+NH_4_]^+^ | TG 54:6\|TG 18:1_18:2_18:3 |
| 22266 | 10.87 | 897.6931 | [M+Na]^+^ | TG 54:8\|TG 18:2_18:3_18:3 |
| 22284 | 11.16 | 898.7853 | [M+NH_4_]^+^ | TG 54:5\|TG 18:1_18:2_18:2 |
| 22297 | 10.98 | 899.7060 | [M+Na]^+^ | TG 54:7\|TG 18:2_18:2_18:3 |
| 22323 | 11.26 | 900.8018 | [M+NH_4_]^+^ | TG 54:4\|TG 18:1_18:1_18:2 |

**Table S4.** Annotated compounds based on MS/MS in organic phase positive ionization mode data. (cont.)

| **ID** | **RT (min)** | ***m/z*** | **Type** | **Metabolite name** |
| --- | --- | --- | --- | --- |
| 22379 | 11.35 | 902.8188 | [M+NH_4_]^+^ | TG 54:3\|TG 18:0_18:1_18:2 |
| 22468 | 9.97 | 906.7152 | [M+NH_4_]^+^ | TG 54:9;O\|TG 18:3_18:3_18:3;O |
| 22469 | 10.29 | 906.7170 | [M+NH_4_]^+^ | TG 54:9;O\|TG 18:3_18:3_18:3;O |
| 22504 | 10.24 | 908.7321 | [M+NH_4_]^+^ | TG 54:8;O\|TG 18:2_18:3_18:3;O |
| 22506 | 11.06 | 908.7702 | [M+NH_4_]^+^ | TG 55:7\|TG 19:1_18:3_18:3 |
| 22727 | 10.77 | 918.7534 | [M+NH_4_]^+^ | TG 56:9\|TG 18:3_18:3_20:3 |

**Table S5.** Annotated compounds by GNPS on the samples analyzed by LC-MS.

| **#** | **Scan** | **Compound_name** | **RT_(min)** | **SpecMZ** | **Adduct** | **Molecular_formula** | **NPclassifier_pathway** |
| --- | --- | --- | --- | --- | --- | --- | --- |
| **1** | 824 | BW 245C | 1.58 | 391.2243 | [M+Na]^+^ | C19H32N2O5 | Alkaloids |
| **2** | 344 | SCHEMBL506340 | 0.78 | 415.2113 | [M+NH_4_]^+^ | C19H28ClN3O4 | Alkaloids |
| **3** | 193 | N,N-Dimethyltryptamine | 0.65 | 189.1387 | [M+H]^+^ | C12H16N2 | Alkaloids |
| **4** | 42 | Chlorogenic acid | 0.59 | 355.1021 | [M+H]^+^ | C16H18O9 | Shikimates and Phenylpropanoids |
| **5** | 78 | Palatinose | 0.59 | 360.1498 | [M+NH_4_]^+^ | C12H22O11 | Carbohydrates |
| **6** | 914 | 1-Hexadecanoyl-sn-glycero-3-phosphocholine | 1.72 | 518.3216 | [M+Na]^+^ | C24H50NO7P | Fatty acids |
| **7** | 117 | Hyperoside | 0.60 | 465.1026 | [M+H]^+^ | C21H20O12 | Shikimates and Phenylpropanoids |
| **8** | 1903 | 1-Stearoyl-2-linoleoyl-sn-glycero-3-phosphocholine | 9.53 | 786.6004 | [M+H]^+^ | C44H84NO8P | Fatty acids |
| **9** | 98 | Rutin | 0.60 | 611.1604 | [M+H]^+^ | C27H30O16 | Shikimates and Phenylpropanoids |
| **10** | 125 | Guaijaverin | 0.61 | 435.092 | [M+H]^+^ | C20H18O11 | Shikimates and Phenylpropanoids |
| **11** | 1783 | 1-Palmitoyl-2-linoleoyl-sn-glycero-3-phosphocholine | 8.96 | 758.5691 | [M+H]^+^ | C42H80NO8P | Fatty acids |
| **12** | 1828 | Arachidonoylthio-PC | 9.16 | 784.5847 | [M+H]^+^ | C44H82NO6P | Fatty acids |
| **13** | 1100 | 1-Stearoyl-2-hydroxy-sn-glycero-3-phosphocholine | 2.67 | 524.371 | [M+H]^+^ | C26H54NO7P | Fatty acids |
| **14** | 934 | 1-(Octadecenoyl)-sn-glycero-3-phosphocholine | 1.79 | 522.3553 | [M+H]^+^ | C26H52NO7P | Fatty acids |
| **15** | 912 | Lyso-PC(16:0) | 1.71 | 496.3396 | [M+H]^+^ | C24H50NO7P | Fatty acids |
| **16** | 742 | alpha.-Linolenoyl ethanolamide | 1.37 | 322.2738 | [M+H_2_O+H] ^+^ | C20H33NO | Fatty acids |
| **17** | 1793 | Arachidonoylthio-PC | 8.97 | 784.5844 | [M+H]^+^ | C44H82NO8P | Fatty acids |
| **18** | 1022 | 1-Heptadecanoyl-sn-glycero-3-phosphocholine | 2.13 | 510.3551 | [M+H]^+^ | C25H52NO7P | Fatty acids |
| **19** | 333 | Unknown | 0.78 | 460.2691 | [M+Na]^+^ | C24H39NO6 | Alkaloids |
| **20** | 322 | Unknown | 0.77 | 432.238 | [M+Na]^+^ | C23H31N5O2 | Alkaloids |
| **21** | 1842 | PC(P-16:0/20:5) | 9.26 | 764.5732 | [M+H]^+^ | C44H78NO7P | Fatty acids |
| **22** | 1900 | 1-Hexadecanoyl-2-(octadecenoyl)-sn-glycero-3-phosphocholine | 9.52 | 760.5847 | [M+H]^+^ | C42H82NO8P | Fatty acids |
| **23** | 1467 | 1,2-Di-(octadecatrienoyl)-sn-glycero-3-phosphocholine | 6.83 | 778.5378 | [M+H]^+^ | C44H76NO8P | Fatty acids |
| **24** | 471 | Hydroxy-octadecatrienoic acid | 0.93 | 277.2162 | [M-H_2_O+H]^+^ | C18H30O3 | Fatty acids |
| **25** | 1897 | 1-Stearoyl-2-myristoyl-sn-glycero-3-phosphocholine | 9.50 | 734.5691 | [M+H]^+^ | C40H80NO8P | Fatty acids |

**Table S5.** Annotated compounds by GNPS on the samples analyzed by LC-MS. (cont.)

| **#** | **Scan** | **Compound_name** | **RT_(min)** | **SpecMZ** | **Adduct** | **Molecular_formula** | **NPclassifier_pathway** |
| --- | --- | --- | --- | --- | --- | --- | --- |
| **26** | 1655 | PC-DAG (16:0/18:3) | 8.52 | 756.5534 | [M+H]+ | C42H78NO8P | Fatty acids |
| **27** | 1277 | 1-Arachidoyl-2-hydroxy-sn-glycero-3-phosphocholine | 4.27 | 552.4023 | [M+H]+ | C28H58NO7P | Fatty acids |
| **28** | 641 | Unknown | 1.19 | 403.2325 | [M+H]+ | C20H34O8 | Fatty acids |
| **29** | 1650 | 1,2-Dilinoleoyl-sn-glycero-3-phosphocholine | 8.51 | 782.5691 | [M+H]+ | C44H80NO8P | Fatty acids |
| **30** | 1149 | 1-Hexadecanoyl-sn-glycerol | 2.83 | 331.284 | [M+H]+ | C19H38O4 | Fatty acids |
| **31** | 611 | Unknown | 1.14 | 400.3056 | [M-H2O+H]+ | C22H43NO6 | Fatty acids |
| **32** | 82 | 3-[3-[3,4-dihydroxy-4-(hydroxymethyl)tetrahydrofuran-2-yl]oxy-4,5-dihydroxy-6-(hydroxymethyl)tetrahydropyran-2-yl]oxy-2-(3,4-dihydroxyphenyl)-5,7-dihydroxy-chromen-4-one | 0.59 | 597.1449 | [M+H]+ | C26H28O16 | Shikimates and Phenylpropanoids |
| **33** | 822 | PC(0:0/16:0) | 1.57 | 496.3396 | [M+H]+ | C24H50NO7P | Fatty acids |
| **34** | 2069 | DAG (16:0/18:1) | 10.36 | 612.5562 | [M+NH4]+ | C37H70O5 | Fatty acids |
| **35** | 404 | Lauryldiethanolamine | 0.85 | 274.2741 | [M+H]+ | C16H35NO2 | Fatty acids |
| **36** | 790 | Phytosphingosine | 1.47 | 318.3001 | [M+H]+ | C18H39NO3 | Fatty acids |
| **37** | 16 | trans-ACPD | 0.58 | 174.0761 | [M+H]+ | C7H11NO4 | Amino acids and Peptides |
| **38** | 1779 | PC(17:0/20:4) | 8.96 | 796.5852 | [M+H]+ | C45H82NO8P | Fatty acids |
| **39** | 948 | 300762-25-8 | 1.85 | 292.2844 | [M+NH4]+ | C16H34O3 | Fatty acids |
| **40** | 1133 | VITAMIN K1 | 2.75 | 226.1801 | M+2H] | C31H46O2 | Terpenoids |
| **41** | 100 | Quercetin | 0.60 | 303.0496 | [M+H]+ | C15H10O7 | Shikimates and Phenylpropanoids |
| **42** | 1082 | N-Oleoylethanolamine | 2.45 | 326.3052 | [M+H2O+H]+ | C20H37NO | Fatty acids |
| **43** | 2007 | 1-Octadecanoyl-2-octadecenoyl-sn-glycero-3-phosphocholine | 10.12 | 788.6163 | [M+H]+ | C44H86NO8P | Fatty acids |
| **44** | 217 | Unknown | 0.66 | 212.1182 | [M-H2O+H]+ | C13H15N3O | Amino acids and Peptides |

**Table S5.** Annotated compounds by GNPS on the samples analyzed by LC-MS. (cont.)

| **#** | **Scan** | **Compound_name** | **RT_(min)** | **SpecMZ** | **Adduct** | **Molecular_formula** | **NPclassifier_pathway** |
| --- | --- | --- | --- | --- | --- | --- | --- |
| **45** | 1095 | Unknown | 2.62 | 282.2789 | [M-H2O+H]+ | C18H37NO2 | Fatty acids |
| **46** | 1904 | DAG (16:0/18:3) | 9.53 | 608.5247 | [M+NH4]+ |  | Fatty acids |
| **47** | 651 | Vitamin E acetate | 1.20 | 228.1957 | [M-H2O+2H]+2 | C31H52O3 | Terpenoids |
| **48** | 264 | Vitamin E acetate | 0.73 | 228.1958 | [M-H2O+2H]+2 | C31H52O3 | Terpenoids |
| **49** | 2184 | Pyropheophytin a | 10.73 | 813.5674 | [M+H]+ | C53H72N4O3 | Terpenoids |
| **50** | 1121 | Vitamin E acetate | 2.73 | 228.1957 | [M-H2O+H]+ | C13H27NO3 | Fatty acids |
| **51** | 1186 | VITAMIN | 3.05 | 226.18 | [M-H2O+H]+ | C13H25NO3 | Fatty acids |
| **52** | 592 | Hydroxy-octadecatrienoic acid | 1.10 | 277.2162 | [M-H2O+H]+ | C18H30O3 | Fatty acids |
| **53** | 2068 | DAG (16:0/16:0) | 10.35 | 586.5404 | [M+H]+ | C43H68O5 | Fatty acids |
| **54** | 1755 | AEG(o-16:3/18:1) | 8.83 | 575.5033 | [M+H]+ | N/A | Fatty acids |
| **55** | 727 | PC(18:2/0:0) | 1.34 | 520.3398 | [M+H]+ | C26H50NO7P | Fatty acids |
| **56** | 578 | PC(18:3/0:0) | 1.08 | 518.3241 | [M+H]+ | C26H48NO7P | Fatty acids |
| **57** | 1180 | Unknown | 3.05 | 228.1957 | [M+H2O+H]+ | C13H23NO | Fatty acids |
| **58** | 1050 | Unknown | 2.31 | 228.1956 | [M-H2O+H]+ | C13H27NO3 | Fatty acids |
| **59** | 1841 | Unknown | 9.26 | 663.4533 | [M+H]+ | C44H58N2O3 | Shikimates and Phenylpropanoids |
| **60** | 864 | Unknown | 1.65 | 228.1957 | [M-H2O+H]+ | C13H27NO3 | Fatty acids |
| **61** | 2273 | Unknown | 10.93 | 712.6446 | [M+NH4]+ | C43H82O6 | Fatty acids |
| **62** | 1753 | SQDG(16:0/18:1) | 8.82 | 838.5705 | [M+NH4]+ | C43H80O12S | Fatty acids |
| **63** | 632 | Unknown | 1.17 | 316.3209 | [M+H2O+H]+ | C19H38O2 | Fatty acids |
| **64** | 1891 | Unknown | 9.49 | 893.5422 | [M+H]+ | C52H76O12 | Terpenoids |
| **65** | 828 | Monolinolenin (9c,12c,15c) | 1.58 | 353.2684 | [M+H]+ | C21H36O4 | Fatty acids |
| **66** | 2077 | Pheophytin a | 10.4 | 871.5728 | [M+H]+ | C55H74N4O5 | Terpenoids |
| **67** | 700 | Unknown | 1.29 | 316.3209 | [M+H]+ | C19H41NO2 | Fatty acids |
| **68** | 1413 | 13-Docosenamide | 5.87 | 338.3415 | [M+H]+ | C22H43NO | Fatty acids |
| **69** | 1786 | AEG(o-16:2/16:0) | 8.96 | 551.5032 | [M+H]+ | C35H64O3 | Fatty acids |
| **70** | 2146 | Unknown | 10.61 | 656.5821 | [M+NH4]+ | C39H74O6 | Fatty acids |
| **71** | 1386 | 13-Docosenamide | 5.59 | 338.3415 | [M+H]+ | C22H43NO | Fatty acids |

**Table S5.** Annotated compounds by GNPS on the samples analyzed by LC-MS. (cont.)

| **#** | **Scan** | **Compound_name** | **RT_(min)** | **SpecMZ** | **Adduct** | **Molecular_formula** | **NPclassifier_pathway** |
| --- | --- | --- | --- | --- | --- | --- | --- |
| **72** | 837 | 1-Hexadecanoyl-2-sn-glycero-3-phosphate | 1.60 | 393.2398 | [M-H2O+H]+ | C19H39O7P | Fatty acids |
| **73** | 731 | Hydroxy-octadecatrienoic acid | 1.36 | 277.2161 | [M+H]+-H2O | C18H30O3 | Fatty acids |
| **74** | 1320 | Amylamine-C16:0 | 4.6 | 326.3416 | [M+H]+ | C21H43NO | Fatty acids |
| **75** | 991 | 13-Docosenamide | 2.04 | 338.3415 | [M+H]+ | C22H43NO | Fatty acids |
| **76** | 666 | Monolinolenin (9c,12c,15c) | 1.23 | 353.2684 | [M+H]+ | C21H36O4 | Fatty acids |
| **77** | 925 | Linoleoyl ethanolamide | 1.76 | 324.2893 | [M+H]+ | C20H37NO2 | Fatty acids |
| **78** | 102 | Trifolin | 0.60 | 449.1076 | [M+H]+ | C21H20O11 | Shikimates and Phenylpropanoids |
| **79** | 1731 | SQDG(16:0/16:0) | 8.79 | 812.5549 | [M+NH4]+ | C41H78O12S | Fatty acids |
| **80** | 1894 | Unknown | 9.5 | 634.5401 | [M+Na]+ | C37H73NO5 | Fatty acids |
| **81** | 1537 | SQDG(16:0/18:3) | 7.88 | 834.5394 | [M+NH4]+ | C43H76O2S | Fatty acids |
| **82** | 2336 | Unknown | 11.36 | 876.801 | [M+NH4]+ | C55H102O6 | Fatty acids |
| **83** | 1629 | Unknown | 8.48 | 575.5032 | [M-H2O+H]+ | C37H68O5 | Fatty acids |
| **84** | 2330 | Unknown | 11.27 | 874.7852 | [M+NH4]+ | C55H100O6 | Fatty acids |
| **85** | 1600 | SQDG(16:0/18:2) | 8.36 | 836.5551 | [M+NH4]+ | C43H78O12S | Fatty acids |
| **86** | 878 | LPC 18:1 | 1.66 | 522.3553 | [M+H]+ | C26H52NO7P | Fatty acids |
| **87** | 2310 | TG(18:1/18:2/18:3) | 11.07 | 896.7689 | [M+NH4]+ | C57H98O6 | Fatty acids |
| **88** | 303 | Unknown | 0.74 | 181.1223 | [M+H2O+H]+ | C11H14O | Terpenoids |
| **89** | 1661 | DG(18:4/18:2/0:0) | 8.53 | 630.5089 | [M+NH4]+ | C39H64O5 | Fatty acids |
| **90** | 966 | Phaeophorbide a | 1.94 | 593.2756 | [M+H]+ | C35H36N4O5 | Alkaloids |
| **91** | 1054 | 1-(nonadecenoyl)-glycero-3-phosphocholine | 2.36 | 536.371 | [M+H]+ | C27H54NO7P | Fatty acids |
| **92** | 907 | 3.10S-Hydroxypheophorbide a | 1.7 | 609.2705 | [M+H]+ | C35H36N4O6 | Alkaloids |
| **93** | 2312 | TG(16:0/18:2/18:3) | 11.08 | 870.7538 | [M+NH4]+ | C55H96O6 | Fatty acids |
| **94** | 189 | 5-methoxy AMT | 0.64 | 205.1335 | [M+H]+ | C12H16N2O | Alkaloids |
| **95** | 1709 | PE(16:0/18:3) | 8.67 | 714.5065 | [M+H]+ | C39H72NO8P | Fatty acids |
| **96** | 37 | Unknown | 0.58 | 163.039 | [M-H2O+H]+ | C9H8O4 | Shikimates and Phenylpropanoids |

**Table S5.** Annotated compounds by GNPS on the samples analyzed by LC-MS. (cont.)

| **#** | **Scan** | **Compound_name** | **RT_(min)** | **SpecMZ** | **Adduct** | **Molecular_formula** | **NPclassifier_pathway** |
| --- | --- | --- | --- | --- | --- | --- | --- |
| **97** | 1017 | 4.Phaeophorbide a | 2.11 | 593.2756 | [M+H]+ | C35H36N4O5 | Alkaloids |
| **98** | 831 | 3.10S-Hydroxypheophorbide a | 1.58 | 609.2705 | [M+H]+ | C35H36N4O6 | Alkaloids |
| **99** | 997 | Unknown | 2.05 | 391.1829 | [M+Na]+ | C14H24N8O4 | Amino acids and Peptides |
| **100** | 2303 | TAG (14:0/14:0/14:0) | 11.05 | 740.6758 | [M+NH4]+ | C45H86O6 | Fatty acids |
| **101** | 472 | Monolinolenin | 0.93 | 353.2684 | [M+H]+ | C21H36O4 | Fatty acids |
| **102** | 1384 | 13-Docosenamide | 5.59 | 675.6759 | 2[M+H]+ | C22H43NO | Fatty acids |
| **103** | 394 | Unknown | 0.84 | 432.251 | [M-H2O+H]+ | C23H35N3O6 | Amino acids and Peptides |
| **104** | 1533 | Unknown | 7.87 | 613.4823 | [M+Na]+ | C37H66O5 | Fatty acids |
| **105** | 2322 | TAG (18:1/18:2/18:2) | 11.17 | 898.7848 | [M+NH4]+ | C57H100O6 | Fatty acids |
| **106** | 2248 | TG(18:2/18:2/18:4) | 10.88 | 892.7378 | [M+NH4]+ | C57H94O6 | Fatty acids |
| **107** | 2192 | TG(18:3/18:3/18:3) | 10.77 | 890.7228 | [M+NH4]+ | C57H92O6 | Fatty acids |
| **108** | 126 | Unknown | 0.61 | 389.2168 | [M-H2O+H]+ | C19H34O9 | Terpenoids |
| **109** | 661 | 9,12,15-Octadecatrienoic acid, 3-(hexopyranosyloxy)-2-hydroxypropyl ester | 1.23 | 532.3481 | [M+NH4]+ | C27H46O9 | Fatty acids |
| **110** | 224 | Loliolide | 0.66 | 197.1173 | [M+H]+ | C11H16O3 | Terpenoids |
| **111** | 1056 | 1-Oleoyl-2-acetyl-sn-glycerol | 2.37 | 381.2994 | [M-H2O+H]+ | C23H42O5 | Fatty acids |
| **112** | 1787 | DG(18:2/18:3/0:0) | 8.97 | 632.5247 | [M+NH4]+ | C39H66O5 | Fatty acids |
| **113** | 2284 | TG(18:2/18:2/18:3) | 10.98 | 894.7532 | [M+NH4]+ | C57H96O6 | Fatty acids |
| **114** | 1886 | DGDG(16:0/18:1) | 9.49 | 936.6616 | [M+NH4]+ | C49H90O15 | Fatty acids |
| **115** | 942 | 1-Palmitoyl-2-hydroxy-sn-glycero-3-phosphoethanolamine | 1.82 | 454.2926 | [M+H]+ | C21H44NO7P | Fatty acids |
| **116** | 541 | Unknown | 1.04 | 391.3105 | [M-H2O+H]+ | C27H40N2O | Alkaloids |
| **117** | 587 | Unknown | 1.10 | 302.3052 | [M+H]+ | C18H39NO2 | Fatty acids |
| **118** | 1821 | PE(16:0/18:2) | 9.13 | 716.5221 | [M+H]+ | C39H74NO8P | Fatty acids |
| **119** | 274 | Unknown | 0.73 | 212.0197 | [M+H]+ | C9H9NOS2 | Alkaloids |
| **120** | 949 | Unknown | 1.85 | 275.258 | [M+H]+ | C16H34O3 | Fatty acids |
| **121** | 1578 | PE(18:2/18:3) | 8.17 | 738.5064 | [M+H]+ | C41H72NO8P | Fatty acids |
| **122** | 2324 | TG(16:0/18:2/18:2) | 11.17 | 872.7696 | [M+NH4]+ | C55H98O6 | Fatty acids |

**Table S5.** Annotated compounds by GNPS on the samples analyzed by LC-MS. (cont.)

| **#** | **Scan** | **Compound_name** | **RT_(min)** | **SpecMZ** | **Adduct** | **Molecular_formula** | **NPclassifier_pathway** |
| --- | --- | --- | --- | --- | --- | --- | --- |
| **123** | 2331 | TG(16:0/16:0/18:2) | 11.27 | 848.7698 | [M+NH4]+ | C53H98O6 | Fatty acids |
| **124** | 1702 | PE(18:2/18:2) | 8.64 | 740.5222 | [M+H]+ | C41H74NO8P | Fatty acids |
| **125** | 1742 | Unknown | 8.81 | 549.4877 | [M-H2O+H]+ | C35H66O5 | Fatty acids |
| **126** | 1554 | Unknown | 8.02 | 573.4876 | [M+H2O+H]+ | C37H62O3 | Fatty acids |
| **127** | 883 | 1-Naphthalenepentanol, decahydro-2-hydroxy-gamma,2,5,5,8a-pentamethyl-, alpha-acetate | 1.68 | 335.2942 | [M+H2O+H]+ | C22H40O3 | Terpenoids |
| **128** | 2309 | TG(18:3/18:3/19:1) | 11.06 | 908.77 | [M+NH4]+ | C58H98O6 | Fatty acids |
| **129** | 596 | 9-Oxo-octadecadienoic acid | 1.11 | 295.2267 | [M+H]+ | C18H30O3 | Fatty acids |
| **130** | 1822 | Unknown | 9.13 | 612.182 | [M+Na]+ | C24H35N3O12S | Amino acids and Peptides |
| **131** | 291 | Tetradecanoic acid | 0.74 | 246.2426 | [M+NH4]+ | C14H28O2 | Fatty acids |
| **132** | 2394 | Unknown | 17.73 | 224.128 | [M-H2O+H]+ | C12H19NO4 | Alkaloids |
| **133** | 2392 | Unknown | 17.55 | 189.0524 | [M+K]+ | C6H14O4 | Fatty acids |
| **134** | 2390 | Unknown | 17.44 | 240.9669 | [M+K]+ | C7H7O5P | Shikimates and Phenylpropanoids |
| **135** | 2389 | Unknown | 17.43 | 226.9513 | [M+H]+ | C6H4Cl2O5 | Shikimates and Phenylpropanoids |
| **136** | 2388 | Unknown | 17.42 | 157.0835 | [M+Na]+ | C6H14O3 | Fatty acids |
| **137** | 2386 | Unknown | 17.43 | 189.0524 | [M+K]+ | C6H14O4 | Fatty acids |
| **138** | 2382 | Unknown | 17.33 | 157.0835 | [M+Na]+ | C6H14O3 | Fatty acids |
| **139** | 2381 | Unknown | 17.33 | 189.0524 | [M+K]+ | C6H14O4 | Fatty acids |
| **140** | 2380 | Unknown | 17.29 | 226.9513 | [M+H]+ | C6H4Cl2O5 | Shikimates and Phenylpropanoids |
| **141** | 2379 | Unknown | 17.23 | 189.0524 | [M+K]+ | C6H14O4 | Fatty acids |
| **142** | 2378 | Unknown | 17.07 | 189.0524 | [M+K]+ | C6H14O4 | Fatty acids |
| **143** | 2339 | Unknown | 11.56 | 264.0847 | [M+K]+ | C8H19NO6 | Carbohydrates |
| **144** | 2338 | Unknown | 11.56 | 263.0815 | [M+Na]+ | C11H16N2O2S | Alkaloids |
| **145** | 2333 | Unknown | 11.34 | 692.6336 | [M-H2O+H]+ | C47H83NO3 | Fatty acids |
| **146** | 2328 | Unknown | 11.20 | 780.6651 | [M+H]+ | C54H85NO2 | Terpenoids |
| **147** | 2301 | Unknown | 11.03 | 766.6494 | [M+NH4]+ | C53H80O2 | Terpenoids |
| **148** | 2271 | Unknown | 10.92 | 717.6000 | [M+Na]+ | C43H82O6 | Fatty acids |
| **149** | 2269 | Unknown | 10.92 | 754.6919 | [M-H2O+H]+ | C46H93NO7 | Fatty acids |
| **150** | 2265 | Unknown | 10.91 | 832.7022 | [M+NH4]+ | C51H90O7 | Terpenoids |
| **151** | 2260 | Unknown | 10.90 | 628.3741 | [M+H2O+H]+ | C38H47N3O4 | Alkaloids |

**Table S5.** Annotated compounds by GNPS on the samples analyzed by LC-MS. (cont.)

| **#** | **Scan** | **Compound_name** | **RT_(min)** | **SpecMZ** | **Adduct** | **Molecular_formula** | **NPclassifier_pathway** |
| --- | --- | --- | --- | --- | --- | --- | --- |
| **153** | 2253 | Unknown | 10.88 | 782.6807 | [M+H]+ | C54H87NO2 | Terpenoids |
| **154** | 2250 | Unknown | 10.88 | 818.6866 | [M-H2O+H]+ | C50H93NO8 | Fatty acids |
| **155** | 2235 | Unknown | 10.84 | 395.3671 | [M-H2O+H]+ | C29H48O | Terpenoids |
| **156** | 2232 | Unknown | 10.83 | 574.5556 | [M+H2O+H]+ | C38H69NO | Fatty acids |
| **157** | 2228 | Unknown | 10.80 | 812.6549 | [M-H2O+H]+ | C47H92NO8P | Fatty acids |
| **158** | 2213 | Unknown | 10.79 | 713.584 | [M+Na]+ | C47H78O3 | Fatty acids |
| **159** | 2212 | Unknown | 10.79 | 689.5674 | [M+Na]+ | C41H78O6 | Fatty acids |
| **160** | 2211 | Unknown | 10.78 | 673.5917 | [M-H2O+H]+ | C47H78O3 | Terpenoids |
| **161** | 2210 | Unknown | 10.79 | 685.6167 | [M+H]+ | C35H76N10O3 | Fatty acids |
| **162** | 2202 | Unknown | 10.78 | 642.6029 | [M+NH4]+ | C39H76O5 | Fatty acids |
| **163** | 2161 | Unknown | 10.65 | 680.5819 | [M+H]+ | C41H77NO6 | Fatty acids |
| **164** | 2139 | Unknown | 10.60 | 824.6909 | [M-H2O+H]+ | C15H14I4N2O7 | Amino acids and Peptides |
| **165** | 2138 | Unknown | 10.60 | 796.66 | [M+H]+ | C54H85NO3 | Terpenoids |
| **166** | 2136 | Unknown | 10.60 | 614.5716 | [M+NH4]+ | C37H72O5 | Fatty acids |
| **167** | 2132 | Unknown | 10.59 | 579.534 | [M-H2O+H]+ | C37H72O5 | Fatty acids |
| **168** | 2128 | Unknown | 10.59 | 761.6227 | [M-H2O+H]+ | C54H82O3 | Terpenoids |
| **169** | 2112 | Unknown | 10.53 | 620.5762 | [M-H2O+H]+ | C43H75NO2 | Fatty acids |
| **170** | 2106 | Unknown | 10.48 | 628.3741 | [M+K]+ | C33H55N3O6 | Fatty acids |
| **171** | 2098 | Unknown | 10.45 | 844.6869 | [M+H]+ | C48H93NO10 | Fatty acids |
| **172** | 2089 | Unknown | 10.44 | 833.2416 | [M+K]+ | C42H42N4O12 | Polyketides |
| **173** | 2085 | Unknown | 10.42 | 652.5508 | [M+NH4]+ | C39H70O6 | Fatty acids |
| **174** | 2083 | Unknown | 10.39 | 638.57 | [M+NH4]+ | C39H72O5 | Fatty acids |
| **175** | 2071 | Unknown | 10.38 | 628.551 | [M+NH4]+ | C37H70O6 | Fatty acids |
| **176** | 2070 | Unknown | 10.38 | 397.3826 | [M+H]+ | C29H48 | Terpenoids |
| **177** | 2066 | Unknown | 10.34 | 830.6714 | [M-H2O+H]+ | C47H93NO11 | Fatty acids |
| **178** | 2065 | Unknown | 10.35 | 747.6073 | [M+Na]+ | C51H80O2 | Terpenoids |
| **179** | 2063 | Unknown | 10.33 | 591.4956 | [M-H2O+H]+ | C37H68O6 | Fatty acids |
| **180** | 2050 | Unknown | 10.30 | 568.5087 | [M-H2O+H]+ | C34H63N7O | Terpenoids |
| **181** | 2048 | Unknown | 10.30 | 798.6753 | [M-H2O+H]+ | C13H12I4N2O7 | Amino acids and Peptides |

**Table S5.** Annotated compounds by GNPS on the samples analyzed by LC-MS. (cont.)

| **#** | **Scan** | **Compound_name** | **RT_(min)** | **SpecMZ** | **Adduct** | **Molecular_formula** | **NPclassifier_pathway** |
| --- | --- | --- | --- | --- | --- | --- | --- |
| **182** | 2030 | Unknown | 10.26 | 565.5665 | [M-H2O+H]+ | C36H74N2O3 | Fatty acids |
| **183** | 2024 | Unknown | 10.23 | 784.6654 | [M-H2O+H]+ | C46H91NO9 | Fatty acids |
| **184** | 2009 | Unknown | 10.15 | 816.6557 | [M+H]+ | C46H89NO10 | Fatty acids |
| **185** | 2005 | Unknown | 10.11 | 295.2266 | [M-H2O+H]+ | C18H32O4 | Fatty acids |
| **186** | 1994 | Unknown | 10.06 | 763.638 | [M-H2O+H]+ | C54H84O3 | Terpenoids |
| **187** | 1988 | Unknown | 10.01 | 611.5436 | [M+H]+ | C41H70O3 | Fatty acids |
| **188** | 1982 | Unknown | 9.97 | 800.6162 | [M+H]+ | C45H86NO8P | Fatty acids |
| **189** | 1975 | Unknown | 9.96 | 749.6229 | [M-H2O+H]+ | C53H82O3 | Terpenoids |
| **190** | 1974 | Unknown | 9.96 | 784.66 | [M+NH4]+ | C53H82O3 | Terpenoids |
| **191** | 1973 | Unknown | 9.96 | 552.5138 | [M-H2O+H]+ | C38H67NO2 | Terpenoids |
| **192** | 1971 | Unknown | 9.96 | 812.6909 | [M-H2O+H]+ | C14H14I4N2O7 | Amino acids and Peptides |
| **193** | 1969 | Unknown | 9.95 | 774.6009 | [M+H]+ | C43H84NO8P | Fatty acids |
| **194** | 1962 | Unknown | 9.87 | 550.6283 | [M-H2O+H]+ | C38H81NO | Fatty acids |
| **195** | 1960 | Unknown | 9.87 | 798.6446 | [M+H]+ | C46H87NO9 | Fatty acids |
| **196** | 1958 | Unknown | 9.87 | 820.6274 | [M+Na]+ | C46H87NO9 | Fatty acids |
| **197** | 1957 | Unknown | 9.86 | 780.6342 | [M+Na]+ | C44H87NO8 | Fatty acids |
| **198** | 1955 | Unknown | 9.84 | 812.6168 | [M+H]+ | C46H86NO8P | Fatty acids |
| **199** | 1943 | Unknown | 9.74 | 764.5738 | [M+H]+ | C38H77N5O10 | Amino acids and Peptides |
| **200** | 1942 | Unknown | 9.73 | 618.5818 | [M+Na]+ | C38H77NO3 | Fatty acids |
| **201** | 1940 | Unknown | 9.73 | 555.5134 | [M-H2O+H]+ | C38H68O3 | Terpenoids |
| **202** | 1939 | Unknown | 9.73 | 595.5059 | [M+Na]+ | C38H68O3 | Fatty acids |
| **203** | 1934 | Unknown | 9.7 | 538.5386 | [M+H]+ | C32H67N5O | Fatty acids |
| **204** | 1933 | Unknown | 9.7 | 537.5353 | [M+H2O+H]+ | C34H66N2O | Fatty acids |
| **205** | 1929 | Unknown | 9.7 | 638.6555 | [M+H2O+H]+ | C40H81N3O | Alkaloids |
| **206** | 1922 | Unknown | 9.69 | 744.5535 | [M+H]+ | C41H78NO8P | Fatty acids |
| **207** | 1918 | Unknown | 9.65 | 786.6004 | [M+H]+ | C44H84NO8P | Fatty acids |
| **208** | 1906 | Unknown | 9.54 | 613.48 | [M+Na]+ | C37H66O5 | Fatty acids |
| **209** | 1902 | Unknown | 9.53 | 636.5553 | [M+Na]+ | C37H75NO5 | Fatty acids |
| **210** | 1901 | Unknown | 9.53 | 782.5667 | [M+Na]+ | C42H82NO8P | Fatty acids |
| **211** | 1875 | Unknown | 9.43 | 798.6008 | [M+H]+ | C45H84NO8P | Fatty acids |
| **212** | 1864 | Unknown | 9.36 | 692.5222 | [M+H]+ | C37H74NO8P | Fatty acids |

**Table S5.** Annotated compounds by GNPS on the samples analyzed by LC-MS. (cont.)

| **#** | **Scan** | **Compound_name** | **RT_(min)** | **SpecMZ** | **Adduct** | **Molecular_formula** | **NPclassifier_pathway** |
| --- | --- | --- | --- | --- | --- | --- | --- |
| **213** | 1863 | Unknown | 9.35 | 777.5482 | [M+Na]+ | C43H78O10 | Fatty acids |
| **214** | 1859 | Unknown | 9.34 | 810.6086 | [M+NH4]+ | C46H80O10 | Fatty acids |
| **215** | 1857 | Unknown | 9.34 | 815.5638 | [M+Na]+ | C46H80O10 | Fatty acids |
| **216** | 1852 | Unknown | 9.28 | 730.5379 | [M+H2O+H]+ | C40H74NO7P | Fatty acids |
| **217** | 1845 | Unknown | 9.26 | 708.5113 | [M+H]+ | C46H65N3O3 | Alkaloids |
| **218** | 1835 | Unknown | 9.22 | 762.5721 | [M-H2O+H]+ | C41H81NO12 | Fatty acids |
| **219** | 1834 | Unknown | 9.22 | 522.5971 | [M-H2O+H]+ | C36H77NO | Fatty acids |
| **220** | 1829 | Unknown | 9.16 | 636.5712 | [M+Na]+ | C41H75NO2 | Terpenoids |
| **221** | 1811 | Unknown | 9.05 | 766.5595 | [M+K]+ | C42H81NO8 | Fatty acids |
| **222** | 1810 | Unknown | 9.03 | 740.5435 | [M+K]+ | C40H79NO8 | Fatty acids |
| **223** | 1805 | Unknown | 9 | 633.4853 | [M+H2O+H]+ | C42H62O3 | Terpenoids |
| **224** | 1800 | Unknown | 8.98 | 716.5221 | [M+H]+ | C39H74NO8P | Fatty acids |
| **225** | 1799 | Unknown | 8.98 | 637.4801 | [M+Na]+ | C39H66O5 | Fatty acids |
| **226** | 1795 | Unknown | 8.98 | 712.6235 | [M+H]+ | C46H81NO4 | Fatty acids |
| **227** | 1794 | Unknown | 8.98 | 698.6079 | [M+H]+ | C45H79NO4 | Fatty acids |
| **228** | 1791 | Unknown | 8.97 | 611.5027 | [M+H]+ | C40H66O4 | Terpenoids |
| **229** | 1788 | Unknown | 8.97 | 628.5297 | [M+NH4]+ | C40H66O4 | Terpenoids |
| **230** | 1784 | Unknown | 8.97 | 780.5508 | [M+Na]+ | C42H80NO8P | Fatty acids |
| **231** | 1769 | Unknown | 8.93 | 828.5595 | [M+H]+ | C41H82NO13P | Fatty acids |
| **232** | 1767 | Unknown | 8.89 | 770.5767 | [M+NH4]+ | C43H76O10 | Fatty acids |
| **233** | 1765 | Unknown | 8.87 | 775.5326 | [M+Na]+ | C43H76O10 | Fatty acids |
| **234** | 1763 | Unknown | 8.86 | 801.5483 | [M+Na]+ | C45H78O10 | Fatty acids |
| **235** | 1761 | Unknown | 8.85 | 798.6081 | [M+Na]+ | C43H85NO10 | Fatty acids |
| **236** | 1760 | Unknown | 8.85 | 716.5222 | [M+H2O+H]+ | C39H72NO7P | Fatty acids |
| **237** | 1759 | Unknown | 8.84 | 769.4987 | [M+Na]+ | C40H75O10P | Fatty acids |
| **238** | 1757 | Unknown | 8.83 | 764.5434 | [M+NH4]+ | C40H75O10P | Fatty acids |
| **239** | 1754 | Unknown | 8.82 | 484.4512 | [M+H]+ | C33H57NO | Terpenoids |
| **240** | 1750 | Unknown | 8.82 | 690.5066 | [M+H]+ | C37H72NO8P | Fatty acids |
| **241** | 1745 | Unknown | 8.81 | 728.5222 | [M+H2O+H]+ | C40H72NO7P | Fatty acids |
| **242** | 1740 | Unknown | 8.81 | 738.5276 | [M+NH4]+ | C38H73O10P | Fatty acids |

**Table S5.** Annotated compounds by GNPS on the samples analyzed by LC-MS. (cont.)

| **#** | **Scan** | **Compound_name** | **RT_(min)** | **SpecMZ** | **Adduct** | **Molecular_formula** | **NPclassifier_pathway** |
| --- | --- | --- | --- | --- | --- | --- | --- |
| **243** | 1739 | Unknown | 8.81 | 664.6026 | [M-H2O+H]+ | C45H79NO3 | Terpenoids |
| **244** | 1737 | Unknown | 8.81 | 636.5712 | [M+Na]+ | C41H75NO2 | Terpenoids |
| **245** | 1728 | Unknown | 8.79 | 817.5103 | [M+Na]+ | C41H78O12S | Fatty acids |
| **246** | 1727 | Unknown | 8.79 | 826.6705 | [M+H2O+H]+ | C48H90NO6P | Fatty acids |
| **247** | 1719 | Unknown | 8.73 | 786.5723 | [M-H2O+H]+ | C43H81NO12 | Fatty acids |
| **248** | 1706 | Unknown | 8.66 | 768.5618 | [M+NH4]+ | C43H74O10 | Fatty acids |
| **249** | 1705 | Unknown | 8.65 | 773.5169 | [M+Na]+ | C43H74O10 | Fatty acids |
| **250** | 1704 | Unknown | 8.69 | 745.5914 | [M+K]+ | C48H82O3 | Terpenoids |
| **251** | 1696 | Unknown | 8.63 | 429.3726 | [M-H2O+H]+ | C29H50O3 | Terpenoids |
| **252** | 1695 | Unknown | 8.63 | 430.3803 | [M-H2O+H]+ | C27H49N3O2 | Alkaloids |
| **253** | 1694 | Unknown | 8.63 | 445.3674 | [M+H]+ | C29H48O3 | Terpenoids |
| **254** | 1687 | Unknown | 8.6 | 368.3886 | [M+H]+ | C24H49NO | Fatty acids |
| **255** | 1673 | Unknown | 8.53 | 409.3827 | [M-H2O+H]+ | C30H50O | Terpenoids |
| **256** | 1672 | Unknown | 8.54 | 413.3776 | [M+H]+ | C29H48O | Terpenoids |
| **257** | 1668 | Unknown | 8.53 | 427.3933 | [M-H2O+H]+ | C30H52O2 | Terpenoids |
| **258** | 1663 | Unknown | 8.53 | 635.4642 | [M+Na]+ | C39H64O5 | Fatty acids |
| **259** | 1658 | Unknown | 8.53 | 478.389 | [M+Na]+ | C27H53NO4 | Fatty acids |
| **260** | 1652 | Unknown | 8.51 | 444.4198 | [M+H2O+H]+ | C30H51N | Terpenoids |
| **261** | 1633 | Unknown | 8.52 | 492.4046 | [M+K]+ | C26H55N5O | Alkaloids |
| **262** | 1617 | Unknown | 8.39 | 799.5327 | [M+Na]+ | C45H76O10 | Fatty acids |
| **263** | 1614 | Unknown | 8.39 | 693.4463 | [M+Na]+ | C37H67O8P | Fatty acids |
| **264** | 1612 | Unknown | 8.38 | 688.4909 | [M+NH4]+ | C37H67O8P | Fatty acids |
| **265** | 1610 | Unknown | 8.39 | 478.389 | [M+Na]+ | C27H53NO4 | Fatty acids |
| **266** | 1608 | Unknown | 8.39 | 464.3733 | [M-H2O+H]+ | C28H51NO5 | Terpenoids |
| **267** | 1607 | Unknown | 8.37 | 714.5066 | [M+H2O+H]+ | C39H70NO7P | Fatty acids |
| **268** | 1602 | Unknown | 8.36 | 762.5277 | [M+NH4]+ | C40H73O10P | Fatty acids |
| **269** | 1591 | Unknown | 8.32 | 784.5566 | [M+Na]+ | C47H75N3O5 | Fatty acids |
| **270** | 1587 | Unknown | 8.29 | 633.4485 | [M+Na]+ | C39H62O5 | Fatty acids |
| **271** | 1586 | Unknown | 8.29 | 628.4935 | [M+NH4]+ | C39H62O5 | Fatty acids |

**Table S5.** Annotated compounds by GNPS on the samples analyzed by LC-MS. (cont.)

| **#** | **Scan** | **Compound_name** | **RT_(min)** | **SpecMZ** | **Adduct** | **Molecular_formula** | **NPclassifier_pathway** |
| --- | --- | --- | --- | --- | --- | --- | --- |
| **272** | 1567 | Unknown | 8.07 | 609.4875 | [M-H2O+H]+ | C40H66O5 | Terpenoids |
| **273** | 1565 | Unknown | 8.07 | 672.5559 | [M+H]+ | C42H73NO5 | Terpenoids |
| **274** | 1562 | Unknown | 8.05 | 409.3827 | [M-H2O+H]+ | C30H50O | Terpenoids |
| **275** | 1552 | Unknown | 8.01 | 833.5173 | [M+H]+ | C43H77O13P | Fatty acids |
| **276** | 1549 | Unknown | 7.99 | 780.5537 | [M+H]+ | C44H78NO8P | Fatty acids |
| **277** | 1535 | Unknown | 7.87 | 820.593 | [M+H]+ | C47H81NO10 | Fatty acids |
| **278** | 1534 | Unknown | 7.87 | 792.5617 | [M+NH4]+ | C45H74O10 | Fatty acids |
| **279** | 1532 | Unknown | 7.87 | 797.5169 | [M+Na]+ | C45H74O10 | Fatty acids |
| **280** | 1529 | Unknown | 7.87 | 795.5712 | [M-H2O+H]+ | C40H76N8O9 | Amino acids and Peptides |
| **281** | 1524 | Unknown | 7.85 | 760.512 | [M+NH4]+ | C40H71O10P | Fatty acids |
| **282** | 1514 | Unknown | 7.60 | 340.357 | [M-H2O+H]+ | C22H47NO2 | Fatty acids |
| **283** | 1513 | Unknown | 7.61 | 679.7075 | [M-H2O+H]+ | C44H92N2O3 | Fatty acids |
| **284** | 1512 | Unknown | 7.57 | 705.7232 | [M-H2O+H]+ | C46H94N2O3 | Fatty acids |
| **285** | 1511 | Unknown | 7.52 | 366.3729 | [M-H2O+H]+ | C24H49NO2 | Fatty acids |
| **286** | 1508 | Unknown | 7.32 | 790.5461 | [M+NH4]+ | C46H68N4O6 | Fatty acids |
| **287** | 1505 | Unknown | 7.24 | 445.3675 | [M-H2O+H]+ | C29H50O4 | Terpenoids |
| **288** | 1496 | Unknown | 7.09 | 667.4154 | [M+Na]+ | C38H60O8 | Terpenoids |
| **289** | 1490 | Unknown | 7.03 | 445.3674 | [M+H]+ | C29H48O3 | Terpenoids |
| **290** | 1484 | Unknown | 6.94 | 429.3726 | [M-H2O+H]+ | C29H50O3 | Terpenoids |
| **291** | 1482 | Unknown | 6.94 | 492.4409 | [M-H2O+H]+ | C25H60N5O3P | Fatty acids |
| **292** | 1479 | Unknown | 6.87 | 451.3569 | [M+Na]+ | C29H48O2 | Terpenoids |
| **293** | 1456 | Unknown | 6.59 | 603.4881 | [M-H2O+H]+ | C40H64N2O3 | Fatty acids |
| **294** | 1450 | Unknown | 6.48 | 710.4752 | [M+H]+ | C41H63N3O7 | Fatty acids |
| **295** | 1440 | Unknown | 6.27 | 776.522 | [M+H]+ | C44H74NO8P | Fatty acids |
| **296** | 1439 | Unknown | 6.26 | 587.5122 | [M-H2O+H]+ | C33H64N8O2 | Alkaloids |
| **297** | 1438 | Unknown | 6.25 | 462.4303 | [M+NH4]+ | C30H52O2 | Terpenoids |
| **298** | 1437 | Unknown | 6.25 | 547.5197 | [M+K]+ | C35H72O | Fatty acids |
| **299** | 1429 | Unknown | 6.15 | 508.4362 | [M-H2O+H]+ | C31H59NO5 | Fatty acids |
| **300** | 1426 | Unknown | 6.12 | 617.5039 | [M+H]+ | C41H64N2O2 | Terpenoids |
| **301** | 1423 | Unknown | 5.97 | 617.5039 | [M+H2O+H]+ | C41H62N2O | Alkaloids |

**Table S5.** Annotated compounds by GNPS on the samples analyzed by LC-MS. (cont.)

| **#** | **Scan** | **Compound_name** | **RT_(min)** | **SpecMZ** | **Adduct** | **Molecular_formula** | **NPclassifier_pathway** |
| --- | --- | --- | --- | --- | --- | --- | --- |
| **302** | 1414 | Unknown | 5.9 | 340.3572 | [M-H2O+H]+ | C22H47NO2 | Fatty acids |
| **303** | 1409 | Unknown | 5.8 | 460.3631 | [M+NH4]+ | C25H46O6 | Fatty acids |
| **304** | 1406 | Unknown | 5.8 | 465.3186 | [M+Na]+ | C25H46O6 | Fatty acids |
| **305** | 1394 | Unknown | 5.6 | 649.6608 | [M+H]+ | C42H84N2O2 | Fatty acids |
| **306** | 1391 | Unknown | 5.6 | 321.3149 | [M+H]+ | C22H40O | Fatty acids |
| **307** | 1390 | Unknown | 5.6 | 312.326 | [M+H]+ | C20H41NO | Fatty acids |
| **308** | 1387 | Unknown | 5.6 | 341.3511 | [M+H]+ | C21H44N2O | Fatty acids |
| **309** | 1385 | Unknown | 5.6 | 355.368 | [M+NH4]+ | C22H43NO | Fatty acids |
| **310** | 1379 | Unknown | 5.44 | 568.4273 | [M+H2O+H]+ | C36H56NOP | Terpenoids |
| **311** | 1376 | Unknown | 5.45 | 389.134 | [M+H]+ | C19H20N2O7 | Alkaloids |
| **312** | 1367 | Unknown | 5.33 | 568.4274 | [M-H2O+H]+ | C36H60NO3P | Terpenoids |
| **313** | 1362 | Unknown | 5.26 | 312.3258 | [M-H2O+H]+ | C20H43NO2 | Fatty acids |
| **314** | 1360 | Unknown | 5.24 | 385.4149 | [M+H]+ | C24H52N2O | Fatty acids |
| **315** | 1359 | Unknown | 5.26 | 357.3837 | [M-H2O+H]+ | C22H50N2O2 | Fatty acids |
| **316** | 1352 | Unknown | 5.18 | 377.2837 | [M+H]+ | C27H36O | Terpenoids |
| **317** | 1351 | Unknown | 5.16 | 405.129 | [M-H2O+H]+ | C19H22N2O9 | Alkaloids |
| **318** | 1349 | Unknown | 5.16 | 772.5485 | [M+H2O+H]+ | C36H77N5O7P2 | Fatty acids |
| **319** | 1344 | Unknown | 5.07 | 324.326 | [M-H2O+H]+ | C21H43NO2 | Fatty acids |
| **320** | 1340 | Unknown | 5.05 | 589.4824 | [M+Na]+ | C35H66O5 | Fatty acids |
| **321** | 1335 | Unknown | 5.01 | 377.2837 | [M-H2O+H]+ | C27H38O2 | Terpenoids |
| **322** | 1330 | Unknown | 4.89 | 336.326 | [M-H2O+H]+ | C22H43NO2 | Fatty acids |
| **323** | 1329 | Unknown | 4.82 | 351.268 | [M-H2O+H]+ | C25H36O2 | Terpenoids |
| **324** | 1323 | Unknown | 4.65 | 351.268 | [M-H2O+H]+ | C25H36O2 | Terpenoids |
| **325** | 1322 | Unknown | 4.61 | 740.4939 | [M+NH4]+ | C40H66O11 | Terpenoids |
| **326** | 1319 | Unknown | 4.6 | 336.3259 | [M-H2O+H]+ | C22H43NO2 | Fatty acids |
| **327** | 1317 | Unknown | 4.52 | 338.3415 | [M-H2O+H]+ | C22H45NO2 | Fatty acids |
| **328** | 1316 | Unknown | 4.52 | 256.227 | [M+H]+ | C15H29NO2 | Fatty acids |
| **329** | 1314 | Unknown | 4.51 | 510.3554 | [M+H]+ | C25H52NO7P | Fatty acids |
| **330** | 1310 | Unknown | 4.5 | 298.3103 | [M+H]+ | C19H39NO | Fatty acids |
| **331** | 1309 | Unknown | 4.47 | 504.4409 | [M-H2O+H]+ | C32H59NO4 | Terpenoids |

**Table S5.** Annotated compounds by GNPS on the samples analyzed by LC-MS. (cont.)

| **#** | **Scan** | **Compound_name** | **RT_(min)** | **SpecMZ** | **Adduct** | **Molecular_formula** | **NPclassifier_pathway** |
| --- | --- | --- | --- | --- | --- | --- | --- |
| **332** | 1307 | Unknown | 4.48 | 436.3419 | [M+Na]+ | C24H47NO4 | Fatty acids |
| **333** | 1288 | Unknown | 4.37 | 336.3259 | [M-H2O+H]+ | C22H43NO2 | Fatty acids |
| **334** | 1287 | Unknown | 4.33 | 312.3258 | [M-H2O+H]+ | C20H43NO2 | Fatty acids |
| **335** | 1286 | Unknown | 4.33 | 520.436 | [M+Na]+ | C30H59NO4 | Fatty acids |
| **336** | 1282 | Unknown | 4.32 | 432.3318 | [M-H2O+H]+ | C23H47NO7 | Fatty acids |
| **337** | 1279 | Unknown | 4.26 | 310.3102 | [M-H2O+H]+ | C20H41NO2 | Fatty acids |
| **338** | 1272 | Unknown | 4.2 | 413.266 | [M+Na]+ | C24H38O4 | Shikimates and Phenylpropanoids |
| **339** | 1268 | Unknown | 4.13 | 256.227 | [M+H2O+H]+ | C15H27NO | Fatty acids |
| **340** | 1267 | Unknown | 4.12 | 459.4155 | [M+H2O+H]+ | C26H52N2O3 | Fatty acids |
| **341** | 1265 | Unknown | 4.08 | 310.3102 | [M+H]+ | C20H39NO | Fatty acids |
| **342** | 1255 | Unknown | 3.89 | 813.5119 | [M+Na]+ | C45H74O11 | Fatty acids |
| **343** | 1249 | Unknown | 3.87 | 436.342 | [M-H2O+H]+ | C26H47NO5 | Terpenoids |
| **344** | 1247 | Unknown | 3.85 | 611.4668 | [M+Na]+ | C37H64O5 | Fatty acids |
| **345** | 1245 | Unknown | 3.81 | 808.5567 | [M+Na]+ | C49H75N3O5 | Fatty acids |
| **346** | 1244 | Unknown | 3.80 | 346.3103 | [M+H]+ | C23H39NO | Fatty acids |
| **347** | 1241 | Unknown | 3.80 | 355.368 | [M+H]+ | C22H46N2O | Fatty acids |
| **348** | 1240 | Unknown | 3.79 | 310.3102 | [M-H2O+H]+ | C20H41NO2 | Fatty acids |
| **349** | 1238 | Unknown | 3.79 | 383.3993 | [M+H]+ | C24H50N2O | Fatty acids |
| **350** | 1236 | Unknown | 3.65 | 324.3258 | [M-H2O+H]+ | C21H43NO2 | Fatty acids |
| **351** | 1231 | Unknown | 3.61 | 284.2947 | [M+H2O+H]+ | C18H35N | Fatty acids |
| **352** | 1230 | Unknown | 3.61 | 329.3525 | [M-H2O+H]+ | C20H46N2O2 | Fatty acids |
| **353** | 1228 | Unknown | 3.62 | 587.4757 | [M-H2O+H]+ | C32H60N8O3 | Alkaloids |
| **354** | 1227 | Unknown | 3.61 | 565.4938 | [M+Na]+ | C32H66N2O4 | Fatty acids |
| **355** | 1223 | Unknown | 3.47 | 568.4273 | [M+H2O+H]+ | C36H56NOP | Terpenoids |
| **356** | 1222 | Unknown | 3.47 | 569.4325 | [M+H]+ | C40H56O2 | Terpenoids |
| **357** | 1221 | Unknown | 3.47 | 551.4247 | [M-H2O+H]+ | C40H56O2 | Terpenoids |
| **358** | 1219 | Unknown | 3.46 | 382.3678 | [M+H2O+H]+ | C24H45NO | Fatty acids |
| **359** | 1216 | Unknown | 3.42 | 806.541 | [M-H2O+H]+ | C45H77NO12 | Fatty acids |
| **360** | 1214 | Unknown | 3.38 | 349.2735 | [M-H2O+H]+ | C22H38O4 | Fatty acids |
| **361** | 1212 | Unknown | 3.35 | 334.3102 | [M-H2O+H]+ | C22H41NO2 | Fatty acids |

**Table S5.** Annotated compounds by GNPS on the samples analyzed by LC-MS. (cont.)

| **#** | **Scan** | **Compound_name** | **RT_(min)** | **SpecMZ** | **Adduct** | **Molecular_formula** | **NPclassifier_pathway** |
| --- | --- | --- | --- | --- | --- | --- | --- |
| **362** | 1210 | Unknown | 3.33 | 516.3895 | [M+H2O+H]+ | C28H51NO6 | Fatty acids |
| **363** | 1207 | Unknown | 3.23 | 568.4273 | [M+H2O+H]+ | C36H56NOP | Alkaloids |
| **364** | 1206 | Unknown | 3.23 | 569.4325 | [M+H2O+H]+ | C35H54N2O3 | Terpenoids |
| **365** | 1204 | Unknown | 3.13 | 334.3102 | [M-H2O+H]+ | C22H41NO2 | Fatty acids |
| **366** | 1202 | Unknown | 3.13 | 585.43 | [M+H2O+H]+ | C40H54O2 | Terpenoids |
| **367** | 1195 | Unknown | 3.05 | 431.3842 | [M+Na]+ | C27H52O2 | Fatty acids |
| **368** | 1191 | Unknown | 3.05 | 459.4155 | [M-H2O+H]+ | C26H56N2O5 | Fatty acids |
| **369** | 1190 | Unknown | 3.05 | 445.3997 | [M-H2O+H]+ | C25H54N2O5 | Fatty acids |
| **370** | 1188 | Unknown | 3.05 | 250.1774 | [M+Na]+ | C13H25NO2 | Alkaloids |
| **371** | 1185 | Unknown | 3.04 | 211.1692 | [M+H]+ | C13H22O2 | Fatty acids |
| **372** | 1184 | Unknown | 3.05 | 209.1535 | [M-H2O+H]+ | C13H22O3 | Fatty acids |
| **373** | 1179 | Unknown | 3.05 | 408.3083 | [M+Na]+ | C22H43NO4 | Fatty acids |
| **374** | 1178 | Unknown | 3.05 | 424.2821 | [M+K]+ | C22H43NO4 | Fatty acids |
| **375** | 1177 | Unknown | 3.05 | 244.1906 | [M-H2O+H]+ | C13H27NO4 | Fatty acids |
| **376** | 1176 | Unknown | 3.04 | 311.2368 | [M-H2O+H]+ | C22H32O2 | Terpenoids |
| **377** | 1171 | Unknown | 3.03 | 193.1588 | [M+H]+ | C13H20O | Terpenoids |
| **378** | 1169 | Unknown | 3.01 | 284.2948 | [M-H2O+H]+ | C18H39NO2 | Fatty acids |
| **379** | 1168 | Unknown | 2.99 | 308.2946 | [M+H]+ | C20H37NO | Fatty acids |
| **380** | 1160 | Unknown | 2.92 | 303.2892 | [M+H]+ | C18H38O3 | Fatty acids |
| **381** | 1159 | Unknown | 2.89 | 311.2368 | [M-H2O+H]+ | C22H32O2 | Terpenoids |
| **382** | 1156 | Unknown | 2.88 | 256.227 | [M+H]+ | C15H29NO2 | Fatty acids |
| **383** | 1155 | Unknown | 2.86 | 482.324 | [M+H]+ | C23H48NO7P | Fatty acids |
| **384** | 1147 | Unknown | 2.83 | 601.4247 | [M-H2O+H]+ | C40H58O5 | Terpenoids |
| **385** | 1146 | Unknown | 2.82 | 364.3419 | [M+H2O+H]+ | C20H43NO3 | Fatty acids |
| **386** | 1144 | Unknown | 2.82 | 270.2791 | [M+H]+ | C17H35NO | Fatty acids |
| **387** | 1143 | Unknown | 2.81 | 308.2947 | [M+H]+ | C20H37NO | Fatty acids |
| **388** | 1138 | Unknown | 2.75 | 374.3627 | [M-H2O+H]+ | C22H49NO4 | Fatty acids |
| **389** | 1137 | Unknown | 2.75 | 607.2912 | [M+H]+ | C36H38N4O5 | Alkaloids |
| **390** | 1135 | Unknown | 2.73 | 408.3083 | [M+Na]+ | C22H43NO4 | Fatty acids |
| **391** | 1134 | Unknown | 2.74 | 431.3842 | [M+Na]+ | C27H52O2 | Fatty acids |

**Table S5.** Annotated compounds by GNPS on the samples analyzed by LC-MS. (cont.)

| **#** | **Scan** | **Compound_name** | **RT_(min)** | **SpecMZ** | **Adduct** | **Molecular_formula** | **NPclassifier_pathway** |
| --- | --- | --- | --- | --- | --- | --- | --- |
| **392** | 1130 | Unknown | 2.73 | 459.4154 | [M-H2O+H]+ | C26H56N2O5 | Fatty acids |
| **393** | 1127 | Unknown | 2.73 | 270.2426 | [M+H]+ | C16H31NO2 | Fatty acids |
| **394** | 1126 | Unknown | 2.72 | 250.1774 | [M+Na]+ | C13H25NO2 | Alkaloids |
| **395** | 1123 | Unknown | 2.74 | 193.1588 | [M+H]+ | C13H20O | Terpenoids |
| **396** | 1122 | Unknown | 2.73 | 175.1482 | [M-H2O+H]+ | C13H20O | Terpenoids |
| **397** | 1117 | Unknown | 2.72 | 793.6274 | [M+H]+ | C46H84N2O8 | Polyketides |
| **398** | 1116 | Unknown | 2.72 | 771.6452 | [M+Na]+ | C47H88O6 | Fatty acids |
| **399** | 1115 | Unknown | 2.73 | 445.3996 | [M-H2O+H]+ | C25H54N2O5 | Fatty acids |
| **400** | 1113 | Unknown | 2.73 | 244.1906 | [M+H]+ | C13H25NO3 | Fatty acids |
| **401** | 1107 | Unknown | 2.72 | 211.1692 | [M+H]+ | C13H22O2 | Fatty acids |
| **402** | 1101 | Unknown | 2.69 | 400.3782 | [M+H2O+H]+ | C24H47NO2 | Fatty acids |
| **403** | 1099 | Unknown | 2.68 | 270.2791 | [M-H2O+H]+ | C17H37NO2 | Fatty acids |
| **404** | 1098 | Unknown | 2.65 | 585.43 | [M+H2O+H]+ | C40H54O2 | Terpenoids |
| **405** | 1093 | Unknown | 2.62 | 308.2946 | [M-H2O+H]+ | C20H39NO2 | Fatty acids |
| **406** | 1092 | Unknown | 2.62 | 353.3522 | [M+H]+ | C22H44N2O | Fatty acids |
| **407** | 1091 | Unknown | 2.61 | 256.227 | [M+H]+ | C15H29NO2 | Fatty acids |
| **408** | 1088 | Unknown | 2.58 | 369.3837 | [M-H2O+H]+ | C23H50N2O2 | Fatty acids |
| **409** | 1086 | Unknown | 2.56 | 607.2912 | [M+H]+ | C35H42O9 | Terpenoids |
| **410** | 1083 | Unknown | 2.52 | 494.324 | [M+H]+ | C24H48NO7P | Fatty acids |
| **411** | 1081 | Unknown | 2.39 | 358.3677 | [M-H2O+H]+ | C22H49NO3 | Fatty acids |
| **412** | 1079 | Unknown | 2.38 | 380.3521 | [M-H2O+H]+ | C24H47NO3 | Fatty acids |
| **413** | 1076 | Unknown | 2.37 | 159.0649 | [M-H2O+H]+ | C7H12O5 | Carbohydrates |
| **414** | 1071 | Unknown | 2.37 | 602.4624 | [M-H2O+H]+ | C33H65NO9 | Fatty acids |
| **415** | 1065 | Unknown | 2.37 | 518.3688 | [M+NH4]+ | C27H48O8 | Fatty acids |
| **416** | 1063 | Unknown | 2.37 | 501.3421 | [M+H]+ | C27H48O8 | Fatty acids |
| **417** | 1058 | Unknown | 2.37 | 523.324 | [M+Na]+ | C27H48O8 | Fatty acids |
| **418** | 1057 | Unknown | 2.36 | 441.321 | [M+H2O+H]+ | C25H42O5 | Fatty acids |
| **419** | 1055 | Unknown | 2.36 | 282.2789 | [M-H2O+H]+ | C18H37NO2 | Fatty acids |
| **420** | 1053 | Unknown | 2.36 | 537.3745 | [M+Na]+ | C29H54O7 | Terpenoids |
| **421** | 1049 | Unknown | 2.32 | 212.2008 | [M-H2O+H]+ | C13H27NO2 | Fatty acids |

**Table S5.** Annotated compounds by GNPS on the samples analyzed by LC-MS. (cont.)

| **#** | **Scan** | **Compound_name** | **RT_(min)** | **SpecMZ** | **Adduct** | **Molecular_formula** | **NPclassifier_pathway** |
| --- | --- | --- | --- | --- | --- | --- | --- |
| **422** | 1046 | Unknown | 2.32 | 336.3259 | [M-H2O+H]+ | C22H43NO2 | Fatty acids |
| **423** | 1043 | Unknown | 2.3 | 427.4257 | [M-H2O+H]+ | C26H56N2O3 | Fatty acids |
| **424** | 1042 | Unknown | 2.3 | 413.4099 | [M+H]+ | C25H52N2O2 | Fatty acids |
| **425** | 1041 | Unknown | 2.31 | 354.3364 | [M+H]+ | C22H43NO2 | Fatty acids |
| **426** | 1039 | Unknown | 2.31 | 399.3944 | [M+H]+ | C24H50N2O2 | Fatty acids |
| **427** | 1029 | Unknown | 2.25 | 601.4249 | [M+H2O+H]+ | C40H54O3 | Terpenoids |
| **428** | 1028 | Unknown | 2.23 | 256.2633 | [M+H]+ | C16H33NO | Fatty acids |
| **429** | 1015 | Unknown | 2.11 | 559.2691 | [M+H]+ | C34H38O7 | Shikimates and Phenylpropanoids |
| **430** | 1014 | Unknown | 2.09 | 604.3264 | [M+H2O+H]+ | C36H43NO6 | Shikimates and Phenylpropanoids |
| **431** | 1012 | Unknown | 2.09 | 581.2506 | [M+Na]+ | C34H38O7 | Shikimates and Phenylpropanoids |
| **432** | 1011 | Unknown | 2.09 | 368.3158 | [M+H2O+H]+ | C22H39NO2 | Fatty acids |
| **433** | 1007 | Unknown | 2.06 | 427.4258 | [M-H2O+H]+ | C26H56N2O3 | Fatty acids |
| **434** | 993 | Unknown | 2.04 | 399.3945 | [M-H2O+H]+ | C24H52N2O3 | Fatty acids |
| **435** | 990 | Unknown | 2.03 | 354.3364 | [M-H2O+H]+ | C22H45NO3 | Fatty acids |
| **436** | 989 | Unknown | 2.04 | 455.457 | [M+H2O+H]+ | C28H56N2O | Alkaloids |
| **437** | 987 | Unknown | 2.04 | 356.3522 | [M+H2O+H]+ | C22H43NO | Fatty acids |
| **438** | 986 | Unknown | 2.03 | 352.3208 | [M+H]+ | C22H41NO2 | Fatty acids |
| **439** | 963 | Unknown | 1.94 | 339.2317 | [M+H]+ | C23H30O2 | Shikimates and Phenylpropanoids |
| **440** | 959 | Unknown | 1.9 | 598.3795 | [M+NH4]+ | C28H52O12 | Fatty acids |
| **441** | 957 | Unknown | 1.9 | 481.3134 | [M+Na]+ | C25H46O7 | Terpenoids |
| **442** | 955 | Unknown | 1.9 | 504.3894 | [M-H2O+H]+ | C23H51N7O6 | Amino acids and Peptides |
| **443** | 953 | Unknown | 1.89 | 476.3581 | [M+NH4]+ | C25H46O7 | Fatty acids |
| **444** | 950 | Unknown | 1.87 | 368.3158 | [M-H2O+H]+ | C22H43NO4 | Fatty acids |
| **445** | 940 | Unknown | 1.83 | 642.578 | [M+Na]+ | C40H77NO3 | Fatty acids |
| **446** | 938 | Unknown | 1.81 | 597.5201 | [M+Na]+ | C38H70O3 | Fatty acids |
| **447** | 937 | Unknown | 1.81 | 579.5095 | [M+Na]+ | C38H68O2 | Fatty acids |
| **448** | 933 | Unknown | 1.79 | 319.2841 | [M+H]+ | C18H38O4 | Fatty acids |
| **449** | 932 | Unknown | 1.79 | 336.3106 | [M+NH4]+ | C18H38O4 | Fatty acids |
| **450** | 929 | Unknown | 1.79 | 400.3783 | [M-H2O+H]+ | C24H51NO4 | Fatty acids |
| **451** | 928 | Unknown | 1.78 | 259.2055 | [M+H]+ | C18H26O | Terpenoids |
| **452** | 917 | Unknown | 1.73 | 380.3369 | [M+H2O+H]+ | C20H43NO4 | Fatty acids |

**Table S5.** Annotated compounds by GNPS on the samples analyzed by LC-MS. (cont.)

| **#** | **Scan** | **Compound_name** | **RT_(min)** | **SpecMZ** | **Adduct** | **Molecular_formula** | **NPclassifier_pathway** |
| --- | --- | --- | --- | --- | --- | --- | --- |
| **453** | 916 | Unknown | 1.72 | 372.347 | [M+H2O+H]+ | C22H43NO2 | Fatty acids |
| **454** | 913 | Unknown | 1.71 | 365.2684 | [M-H2O+H]+ | C22H38O5 | Fatty acids |
| **455** | 902 | Unknown | 1.69 | 672.4163 | [M+NH4]+ | C31H58O14 | Fatty acids |
| **456** | 900 | Unknown | 1.69 | 424.2633 | [M+NH4]+ | C30H30O | Shikimates and Phenylpropanoids |
| **457** | 897 | Unknown | 1.69 | 411.3943 | [M-H2O+H]+ | C25H52N2O3 | Fatty acids |
| **458** | 895 | Unknown | 1.69 | 352.3208 | [M+H]+ | C22H41NO2 | Fatty acids |
| **459** | 893 | Unknown | 1.68 | 425.4099 | [M+H]+ | C26H52N2O2 | Fatty acids |
| **460** | 892 | Unknown | 1.69 | 398.3822 | [M+Na]+ | C18H47N8 | Alkaloids |
| **461** | 891 | Unknown | 1.69 | 397.3787 | [M-H2O+H]+ | C24H50N2O3 | Fatty acids |
| **462** | 871 | Unknown | 1.65 | 654.3317 | [M+NH4]+ | C30H44N4O11 | Polyketides |
| **463** | 866 | Unknown | 1.65 | 696.3794 | [M-H2O+H]+ | C40H51N5O7 | Amino acids and Peptides |
| **464** | 865 | Unknown | 1.65 | 738.4255 | [M+H]+ | C43H55N5O6 | Amino acids and Peptides |
| **465** | 860 | Unknown | 1.64 | 336.3259 | [M-H2O+H]+ | C22H43NO2 | Fatty acids |
| **466** | 857 | Unknown | 1.64 | 415.3892 | [M+H]+ | C24H50N2O3 | Fatty acids |
| **467** | 855 | Unknown | 1.64 | 399.3945 | [M-H2O+H]+ | C24H52N2O3 | Fatty acids |
| **468** | 841 | Unknown | 1.62 | 392.3133 | [M+Na]+ | C22H43NO3 | Fatty acids |
| **469** | 836 | Unknown | 1.60 | 428.2771 | [M+NH4]+ | C19H39O7P | Fatty acids |
| **470** | 832 | Unknown | 1.59 | 524.3429 | [M+H2O+H]+ | C26H43N5O5 | Fatty acids |
| **471** | 829 | Unknown | 1.58 | 370.295 | [M+NH4]+ | C21H36O4 | Fatty acids |
| **472** | 825 | Unknown | 1.57 | 214.1801 | [M+H]+ | C12H23NO2 | Fatty acids |
| **473** | 817 | Unknown | 1.56 | 672.4163 | [M+K]+ | C39H59N3O4 | Fatty acids |
| **474** | 814 | Unknown | 1.55 | 330.3364 | [M+H]+ | C20H43NO2 | Fatty acids |
| **475** | 813 | Unknown | 1.54 | 601.4249 | [M-H2O+H]+ | C40H58O5 | Terpenoids |
| **476** | 812 | Unknown | 1.55 | 242.2113 | [M+H2O+H]+ | C14H25NO | Fatty acids |
| **477** | 807 | Unknown | 1.52 | 502.3737 | [M-H2O+H]+ | C27H53NO8 | Polyketides |
| **478** | 806 | Unknown | 1.52 | 474.3425 | [M+NH4]+ | C25H44O7 | Fatty acids |
| **479** | 803 | Unknown | 1.52 | 326.3052 | [M+H2O+H]+ | C20H37NO | Fatty acids |
| **480** | 799 | Unknown | 1.51 | 530.3476 | [M+H]+ | C31H47NO6 | Terpenoids |
| **481** | 798 | Unknown | 1.51 | 502.3162 | [M+NH4]+ | C29H40O6 | Terpenoids |

**Table S5.** Annotated compounds by GNPS on the samples analyzed by LC-MS. (cont.)

| **#** | **Scan** | **Compound_name** | **RT_(min)** | **SpecMZ** | **Adduct** | **Molecular_formula** | **NPclassifier_pathway** |
| --- | --- | --- | --- | --- | --- | --- | --- |
| **482** | 794 | Unknown | 1.49 | 583.4145 | [M-H2O+H]+ | C40H56O4 | Terpenoids |
| **483** | 791 | Unknown | 1.47 | 228.2321 | [M-H2O+H]+ | C14H31NO2 | Fatty acids |
| **484** | 771 | Unknown | 1.42 | 478.2926 | [M+H]+ | C23H44NO7P | Fatty acids |
| **485** | 769 | Unknown | 1.41 | 351.2528 | [M+H]+ | C21H34O4 | Terpenoids |
| **486** | 763 | Unknown | 1.38 | 530.3699 | [M-H2O+H]+ | C25H45N11O3 | Alkaloids |
| **487** | 758 | Unknown | 1.38 | 295.1725 | [M-H2O+H]+ | C17H28O3S | Polyketides |
| **488** | 757 | Unknown | 1.38 | 573.3034 | [M-H2O+H]+ | C25H51O13P | Fatty acids |
| **489** | 753 | Unknown | 1.38 | 502.3381 | [M+Na]+ | C27H47N2O5 | Terpenoids |
| **490** | 751 | Unknown | 1.38 | 583.4145 | [M-H2O+H]+ | C40H56O4 | Terpenoids |
| **491** | 750 | Unknown | 1.38 | 479.2622 | [M+Na]+ | C24H40O8 | Terpenoids |
| **492** | 747 | Unknown | 1.38 | 544.3851 | [M-H2O+H]+ | C29H55NO9 | Polyketides |
| **493** | 740 | Unknown | 1.37 | 395.3631 | [M-H2O+H]+ | C24H48N2O3 | Fatty acids |
| **494** | 738 | Unknown | 1.36 | 375.268 | [M-H2O+H]+ | C27H36O2 | Terpenoids |
| **495** | 736 | Unknown | 1.36 | 350.305 | [M+H]+ | C22H39NO2 | Fatty acids |
| **496** | 730 | Unknown | 1.35 | 629.2605 | [M-H2O+H]+ | C33H42O13 | Terpenoids |
| **497** | 720 | Unknown | 1.32 | 397.3788 | [M-H2O+H]+ | C24H50N2O3 | Fatty acids |
| **498** | 718 | Unknown | 1.33 | 334.3102 | [M-H2O+H]+ | C22H41NO2 | Fatty acids |
| **499** | 716 | Unknown | 1.33 | 368.3158 | [M+H]+ | C22H41NO3 | Fatty acids |
| **500** | 713 | Unknown | 1.32 | 441.405 | [M-H2O+H]+ | C26H54N2O4 | Fatty acids |
| **501** | 711 | Unknown | 1.32 | 413.3735 | [M-H2O+H]+ | C24H50N2O4 | Fatty acids |
| **502** | 705 | Unknown | 1.31 | 458.3111 | [M+NH4]+ | C24H40O7 | Fatty acids |
| **503** | 703 | Unknown | 1.29 | 639.4321 | [M+H]+ | C33H58N4O8 | Polyketides |
| **504** | 702 | Unknown | 1.3 | 258.2426 | [M-H2O+H]+ | C15H33NO3 | Fatty acids |
| **505** | 701 | Unknown | 1.29 | 297.2423 | [M+H2O+H]+ | C18H30O2 | Fatty acids |
| **506** | 693 | Unknown | 1.26 | 244.1906 | [M+H]+ | C13H25NO3 | Fatty acids |
| **507** | 690 | Unknown | 1.26 | 431.3845 | [M+Na]+ | C27H52O2 | Fatty acids |
| **508** | 689 | Unknown | 1.26 | 408.3083 | [M+Na]+ | C22H43NO4 | Fatty acids |
| **509** | 686 | Unknown | 1.26 | 390.1884 | [M+H]+ | C17H23N7O4 | Alkaloids |

**Table S5.** Annotated compounds by GNPS on the samples analyzed by LC-MS. (cont.)

| **#** | **Scan** | **Compound_name** | **RT_(min)** | **SpecMZ** | **Adduct** | **Molecular_formula** | **NPclassifier_pathway** |
| --- | --- | --- | --- | --- | --- | --- | --- |
| **510** | 681 | Unknown | 1.24 | 272.2946 | [M-H2O+H]+ | C17H39NO2 | Fatty acids |
| **511** | 671 | Unknown | 1.22 | 417.405 | [M+Na]+ | C27H54O | Fatty acids |
| **512** | 669 | Unknown | 1.22 | 395.3326 | [M-H2O+H]+ | C28H44O2 | Terpenoids |
| **513** | 662 | Unknown | 1.23 | 418.4084 | [M+H]+ | C18H47N11 | Alkaloids |
| **514** | 657 | Unknown | 1.22 | 372.3471 | [M+H]+ | C22H45NO3 | Fatty acids |
| **515** | 653 | Unknown | 1.22 | 324.2895 | [M-H2O+H]+ | C20H39NO3 | Fatty acids |
| **516** | 647 | Unknown | 1.19 | 429.3687 | [M+H]+ | C24H48N2O4 | Fatty acids |
| **517** | 644 | Unknown | 1.19 | 348.289 | [M+H]+ | C22H37NO2 | Fatty acids |
| **518** | 640 | Unknown | 1.19 | 425.2144 | [M+Na]+ | C20H34O8 | Fatty acids |
| **519** | 639 | Unknown | 1.18 | 393.2785 | [M-H2O+H]+ | C27H38O3 | Terpenoids |
| **520** | 635 | Unknown | 1.15 | 352.3208 | [M+H]+ | C22H41NO2 | Fatty acids |
| **521** | 633 | Unknown | 1.17 | 291.2316 | [M-H2O+H]+ | C19H32O3 | Fatty acids |
| **522** | 631 | Unknown | 1.17 | 352.3055 | [M+Na]+ | C17H39N5O | Alkaloids |
| **523** | 629 | Unknown | 1.17 | 287.0912 | [M+H]+ | C16H14O5 | Shikimates and Phenylpropanoids |
| **524** | 626 | Unknown | 1.16 | 356.2793 | [M-H2O+H]+ | C20H39NO5 | Fatty acids |
| **525** | 622 | Unknown | 1.16 | 471.452 | [M-H2O+H]+ | C28H60N2O4 | Fatty acids |
| **526** | 621 | Unknown | 1.16 | 457.4364 | [M-H2O+H]+ | C27H58N2O4 | Fatty acids |
| **527** | 620 | Unknown | 1.15 | 370.3314 | [M+H2O+H]+ | C22H41NO2 | Fatty acids |
| **528** | 619 | Unknown | 1.15 | 415.3893 | [M-H2O+H]+ | C24H52N2O4 | Fatty acids |
| **529** | 612 | Unknown | 1.12 | 392.3135 | [M+Na]+ | C22H43NO3 | Fatty acids |
| **530** | 610 | Unknown | 1.15 | 396.3321 | [M+H2O+H]+ | C20H43NO5 | Fatty acids |
| **531** | 609 | Unknown | 1.13 | 476.2771 | [M+H]+ | C23H42NO7P | Fatty acids |
| **532** | 604 | Unknown | 1.12 | 440.3579 | [M-H2O+H]+ | C23H47N5O4 | Fatty acids |
| **533** | 603 | Unknown | 1.12 | 367.2629 | [M-H2O+H]+ | C25H36O3 | Terpenoids |
| **534** | 595 | Unknown | 1.11 | 278.2194 | [M+H]+ | C13H29N2O4 | Fatty acids |
| **535** | 593 | Unknown | 1.11 | 340.2844 | [M+H]+ | C20H37NO3 | Fatty acids |
| **536** | 589 | Unknown | 1.1 | 364.3208 | [M-H2O+H]+ | C23H43NO3 | Fatty acids |
| **537** | 588 | Unknown | 1.1 | 607.2549 | [M+H2O+H]+ | C34H36O9 | Shikimates and Phenylpropanoids |
| **538** | 584 | Unknown | 1.09 | 555.3138 | [M+Na]+ | C27H48O10 | Polyketides |
| **539** | 582 | Unknown | 1.09 | 502.3163 | [M-H2O+H]+ | C29H45NO7 | Polyketides |

**Table S5.** Annotated compounds by GNPS on the samples analyzed by LC-MS. (cont.)

| **#** | **Scan** | **Compound_name** | **RT_(min)** | **SpecMZ** | **Adduct** | **Molecular_formula** | **NPclassifier_pathway** |
| --- | --- | --- | --- | --- | --- | --- | --- |
| **540** | 573 | Unknown | 1.07 | 472.3395 | [M+K]+ | C25H47N5O | Alkaloids |
| **541** | 571 | Unknown | 1.07 | 500.3709 | [M+H]+ | C26H49N3O6 | Amino acids and Peptides |
| **542** | 563 | Unknown | 1.05 | 366.3001 | [M-H2O+H]+ | C22H41NO4 | Fatty acids |
| **543** | 562 | Unknown | 1.07 | 279.2316 | [M+H]+ | C18H30O2 | Fatty acids |
| **544** | 560 | Unknown | 1.06 | 694.4006 | [M+NH4]+ | C33H56O14 | Fatty acids |
| **545** | 554 | Unknown | 1.06 | 388.282 | [M+Na]+ | C22H39NO3 | Fatty acids |
| **546** | 552 | Unknown | 1.07 | 421.2324 | [M+Na]+ | C21H30N6O2 | Alkaloids |
| **547** | 546 | Unknown | 1.05 | 324.2168 | [M+H]+ | C18H29NO4 | Shikimates and Phenylpropanoids |
| **548** | 542 | Unknown | 1.05 | 409.2735 | [M-H2O+H]+ | C27H38O4 | Terpenoids |
| **549** | 540 | Unknown | 1.04 | 304.2844 | [M-H2O+H]+ | C17H39NO4 | Fatty acids |
| **550** | 536 | Unknown | 1.02 | 178.1591 | [M+H]+ | C12H19N | Alkaloids |
| **551** | 531 | Unknown | 1.01 | 324.2353 | [M+H]+ | C19H33NOS | Terpenoids |
| **552** | 530 | Unknown | 1.01 | 422.1605 | [M+Na]+ | C20H25N5O2S | Alkaloids |
| **553** | 528 | Unknown | 1 | 516.3085 | [M+H]+ | C26H46NO7P | Fatty acids |
| **554** | 524 | Unknown | 0.96 | 181.1223 | [M+H]+ | C11H16O2 | Terpenoids |
| **555** | 522 | Unknown | 0.97 | 400.3057 | [M+NH4]+ | C22H38O5 | Fatty acids |
| **556** | 519 | Unknown | 0.97 | 414.2848 | [M+H]+ | C23H35N5O2 | Alkaloids |
| **557** | 517 | Unknown | 0.96 | 390.2975 | [M+Na]+ | C22H41NO3 | Fatty acids |
| **558** | 516 | Unknown | 0.97 | 348.2896 | [M-H2O+H]+ | C22H39NO3 | Fatty acids |
| **559** | 507 | Unknown | 0.97 | 413.3736 | [M+H2O+H]+ | C24H46N2O2 | Fatty acids |
| **560** | 506 | Unknown | 0.96 | 368.3159 | [M-H2O+H]+ | C22H43NO4 | Fatty acids |
| **561** | 495 | Unknown | 0.95 | 288.2896 | [M+H2O+H]+ | C17H35NO | Fatty acids |
| **562** | 489 | Unknown | 0.96 | 429.3688 | [M-H2O+H]+ | C24H50N2O5 | Fatty acids |
| **563** | 488 | Unknown | 0.95 | 384.3105 | [M+H]+ | C22H41NO4 | Fatty acids |
| **564** | 485 | Unknown | 0.94 | 186.2217 | [M+H]+ | C12H27N | Fatty acids |
| **565** | 480 | Unknown | 0.93 | 339.1905 | [M+Na]+ | C15H28N2O5 | Amino acids and Peptides |
| **566** | 477 | Unknown | 0.93 | 244.2634 | [M-H2O+H]+ | C15H35NO2 | Fatty acids |
| **567** | 470 | Unknown | 0.95 | 406.2927 | [M+Na]+ | C22H41NO4 | Fatty acids |
| **568** | 467 | Unknown | 0.93 | 431.3844 | [M+Na]+ | C27H52O2 | Fatty acids |
| **569** | 466 | Unknown | 0.93 | 386.3263 | [M+H]+ | C22H43NO4 | Fatty acids |

**Table S5.** Annotated compounds by GNPS on the samples analyzed by LC-MS. (cont.)

| **#** | **Scan** | **Compound_name** | **RT_(min)** | **SpecMZ** | **Adduct** | **Molecular_formula** | **NPclassifier_pathway** |
| --- | --- | --- | --- | --- | --- | --- | --- |
| **570** | 461 | Unknown | 0.94 | 289.293 | [M+H]+ | C13H34N7 | Alkaloids |
| **571** | 453 | Unknown | 0.93 | 408.3083 | [M+Na]+ | C22H43NO4 | Fatty acids |
| **572** | 452 | Unknown | 0.92 | 290.2689 | [M+H2O+H]+ | C16H33NO2 | Fatty acids |
| **573** | 445 | Unknown | 0.91 | 352.3208 | [M+H]+ | C22H41NO2 | Fatty acids |
| **574** | 433 | Unknown | 0.89 | 291.1951 | [M+H2O+H]+ | C18H24O2 | Terpenoids |
| **575** | 428 | Unknown | 0.89 | 643.2398 | [M+Na]+ | C37H36N2O7 | Alkaloids |
| **576** | 425 | Unknown | 0.89 | 388.3421 | [M+H]+ | C22H45NO4 | Fatty acids |
| **577** | 419 | Unknown | 0.88 | 350.3053 | [M+H]+ | C22H39NO2 | Fatty acids |
| **578** | 414 | Unknown | 0.87 | 302.2687 | [M+H]+ | C17H35NO3 | Fatty acids |
| **579** | 410 | Unknown | 0.86 | 296.2558 | [M+Na]+ | C16H35NO2 | Fatty acids |
| **580** | 403 | Unknown | 0.85 | 438.1554 | [M-H2O+H]+ | C24H25NO8 | Amino acids and Peptides |
| **581** | 402 | Unknown | 0.85 | 360.232 | [M+H]+ | C25H29NO | Alkaloids |
| **582** | 399 | Unknown | 0.85 | 330.2636 | [M+NH4]+ | C18H32O4 | Fatty acids |
| **583** | 397 | Unknown | 0.84 | 295.2268 | [M-H2O+H]+ | C18H32O4 | Fatty acids |
| **584** | 396 | Unknown | 0.84 | 367.2478 | [M-H2O+H]+ | C21H36O6 | Fatty acids |
| **585** | 393 | Unknown | 0.84 | 318.3001 | [M+H]+ | C18H39NO3 | Fatty acids |
| **586** | 390 | Unknown | 0.84 | 387.1929 | [M+H]+ | C20H31ClO5 | Polyketides |
| **587** | 386 | Unknown | 0.83 | 391.2452 | [M+Na]+ | C21H36O5 | Terpenoids |
| **588** | 384 | Unknown | 0.82 | 414.3212 | [M+H2O+H]+ | C24H37N5 | Alkaloids |
| **589** | 382 | Unknown | 0.82 | 362.3262 | [M+H]+ | C20H43NO4 | Fatty acids |
| **590** | 365 | Unknown | 0.81 | 256.2269 | [M+H2O+H]+ | C15H27NO | Fatty acids |
| **591** | 364 | Unknown | 0.81 | 465.3475 | [M-H2O+H]+ | C30H46N2O3 | Terpenoids |
| **592** | 355 | Unknown | 0.79 | 356.2793 | [M-H2O+H]+ | C20H39NO5 | Fatty acids |
| **593** | 345 | Unknown | 0.78 | 328.248 | [M+NH4]+ | C18H30O4 | Fatty acids |
| **594** | 342 | Unknown | 0.78 | 293.2109 | [M-H2O+H]+ | C18H30O4 | Fatty acids |
| **595** | 328 | Unknown | 0.77 | 502.3162 | [M+H]+ | C29H43NO6 | Terpenoids |
| **596** | 326 | Unknown | 0.77 | 488.3003 | [M+H]+ | C28H41NO6 | Terpenoids |
| **597** | 324 | Unknown | 0.77 | 453.1673 | [M+K]+ | C25H26N4O2 | Alkaloids |
| **598** | 317 | Unknown | 0.76 | 226.1225 | [M+H]+ | C15H15NO | Alkaloids |
| **599** | 313 | Unknown | 0.76 | 271.0027 | [M+H]+ | C11H8Cl2N2O2 | Alkaloids |

**Table S5.** Annotated compounds by GNPS on the samples analyzed by LC-MS. (cont.)

| **#** | **Scan** | **Compound_name** | **RT_(min)** | **SpecMZ** | **Adduct** | **Molecular_formula** | **NPclassifier_pathway** |
| --- | --- | --- | --- | --- | --- | --- | --- |
| **600** | 312 | Unknown | 0.76 | 207.1416 | [M-H2O+H]+ | C10H24O3S | Fatty acids |
| **601** | 308 | Unknown | 0.75 | 422.2666 | [M+Na]+ | C25H37NO3 | Alkaloids |
| **602** | 304 | Unknown | 0.74 | 189.0525 | [M+Na]+ | C3H10BNO6 | Amino acids and Peptides |
| **603** | 302 | Unknown | 0.74 | 250.201 | [M+Na]+ | C11H25N5 | Alkaloids |
| **604** | 300 | Unknown | 0.76 | 205.1434 | [M+H]+ | C10H20O4 | Fatty acids |
| **605** | 299 | Unknown | 0.74 | 317.1148 | [M+K]+ | C16H22O4 | Terpenoids |
| **606** | 295 | Unknown | 0.73 | 178.1228 | [M+H]+ | C11H15NO | Alkaloids |
| **607** | 290 | Unknown | 0.74 | 240.1957 | [M-H2O+H]+ | C14H27NO3 | Fatty acids |
| **608** | 289 | Unknown | 0.74 | 300.2532 | [M+H2O+H]+ | C17H31NO2 | Alkaloids |
| **609** | 281 | Unknown | 0.73 | 293.1512 | [M+K]+ | C15H26O3 | Terpenoids |
| **610** | 280 | Unknown | 0.73 | 272.2219 | [M+NH4]+ | C15H26O3 | Terpenoids |
| **611** | 270 | Unknown | 0.73 | 237.1847 | [M-H2O+H]+ | C15H26O3 | Terpenoids |
| **612** | 255 | Unknown | 0.71 | 258.2064 | [M-H2O+H]+ | C14H29NO4 | Fatty acids |
| **613** | 251 | Unknown | 0.7 | 289.1795 | [M+H]+ | C18H24O3 | Terpenoids |
| **614** | 250 | Unknown | 0.7 | 207.1591 | [M-H2O+H]+ | C10H24O5 | Fatty acids |
| **615** | 249 | Unknown | 0.7 | 348.2743 | [M+NH4]+ | C18H34O5 | Fatty acids |
| **616** | 248 | Unknown | 0.7 | 291.1953 | [M-H2O+H]+ | C18H28O4 | Fatty acids |
| **617** | 242 | Unknown | 0.7 | 293.1744 | [M+Na]+ | C15H26O4 | Fatty acids |
| **618** | 241 | Unknown | 0.7 | 214.1801 | [M+H]+ | C12H23NO2 | Fatty acids |
| **619** | 240 | Unknown | 0.69 | 198.1855 | [M-H2O+H]+ | C12H25NO2 | Fatty acids |
| **620** | 239 | Unknown | 0.69 | 235.1692 | [M-H2O+H]+ | C15H24O3 | Terpenoids |
| **621** | 238 | Unknown | 0.71 | 271.144 | [M+H]+ | C16H18N2O2 | Alkaloids |
| **622** | 236 | Unknown | 0.67 | 346.2586 | [M-H2O+H]+ | C18H37NO6 | Fatty acids |
| **623** | 233 | Unknown | 0.67 | 439.23 | [M+Na]+ | C21H36O8 | Terpenoids |
| **624** | 232 | Unknown | 0.69 | 218.2113 | [M+H]+ | C12H27NO2 | Fatty acids |
| **625** | 230 | Unknown | 0.67 | 216.196 | [M+H]+ | C12H25NO2 | Fatty acids |
| **626** | 229 | Unknown | 0.67 | 198.1276 | [M-H2O+H]+ | C14H17NO | Alkaloids |
| **627** | 228 | Unknown | 0.67 | 130.0652 | [M-H2O+H]+ | C9H9NO | Alkaloids |
| **628** | 225 | Unknown | 0.67 | 132.0808 | [M-H2O+H]+ | C9H11NO | Alkaloids |
| **629** | 223 | Unknown | 0.67 | 161.1075 | [M-H2O+H]+ | C10H14N2O | Alkaloids |
| **630** | 222 | Unknown | 0.66 | 122.0965 | [M+H]+ | C8H11N | Alkaloids |

**Table S5.** Annotated compounds by GNPS on the samples analyzed by LC-MS. (cont.)

| **#** | **Scan** | **Compound_name** | **RT_(min)** | **SpecMZ** | **Adduct** | **Molecular_formula** | **NPclassifier_pathway** |
| --- | --- | --- | --- | --- | --- | --- | --- |
| **631** | 221 | Unknown | 0.69 | 244.1905 | [M-H2O+H]+ | C13H27NO4 | Fatty acids |
| **632** | 220 | Unknown | 0.66 | 191.1178 | [M-H2O+H]+ | C11H16N2O2 | Alkaloids |
| **633** | 213 | Unknown | 0.66 | 230.175 | [M-H2O+H]+ | C12H25NO4 | Fatty acids |
| **634** | 212 | Unknown | 0.65 | 212.1645 | [M-H2O+H]+ | C12H23NO3 | Fatty acids |
| **635** | 211 | Unknown | 0.67 | 114.0914 | [M-H2O+H]+ | C6H13NO2 | Amino acids and Peptides |
| **636** | 205 | Unknown | 0.65 | 144.0808 | [M-H2O+H]+ | C10H11NO | Alkaloids |
| **637** | 203 | Unknown | 0.65 | 118.0652 | [M-H2O+H]+ | C8H9NO | Alkaloids |
| **638** | 201 | Unknown | 0.65 | 143.0729 | [M-H2O+H]+ | C4H10BN2O4 | Amino acids and Peptides |
| **639** | 195 | Unknown | 0.65 | 190.1416 | [M+H]+ | C5H15N7O | Alkaloids |
| **640** | 190 | Unknown | 0.65 | 113.1074 | [M-H2O+H]+ | C6H14N2O | Alkaloids |
| **641** | 187 | Unknown | 0.64 | 203.1544 | [M+H]+ | C13H18N2 | Alkaloids |
| **642** | 186 | Unknown | 0.65 | 145.0841 | [M-H2O+H]+ | C3H10N6O2 | Alkaloids |
| **643** | 183 | Unknown | 0.62 | 416.247 | [M+H]+ | C22H33N5OS | Alkaloids |
| **644** | 181 | Unknown | 0.65 | 206.1369 | [M+Na]+ | C8H17N5 | Alkaloids |
| **645** | 179 | Unknown | 0.64 | 221.1283 | [M+H]+ | C12H16N2O2 | Alkaloids |
| **646** | 174 | Unknown | 0.63 | 477.2019 | [M+H2O+H]+ | C27H26N2O5 | Alkaloids |
| **647** | 172 | Unknown | 0.65 | 350.2172 | [M-H2O+H]+ | C16H33NO8 | Fatty acids |
| **648** | 165 | Unknown | 0.63 | 413.2464 | [M+H]+ | C25H28N6 | Alkaloids |
| **649** | 158 | Unknown | 0.62 | 134.1176 | [M-H2O+H]+ | C6H17NO3 | Alkaloids |
| **650** | 127 | Unknown | 0.61 | 136.0618 | [M+H]+ | C5H5N5 | Alkaloids |
| **651** | 122 | Unknown | 0.61 | 429.2171 | [M+H]+ | C27H28N2O3 | Alkaloids |
| **652** | 120 | Unknown | 0.61 | 461.2069 | [M-H2O+H]+ | C27H30N2O6 | Alkaloids |
| **653** | 118 | Unknown | 0.61 | 531.1649 | [M-H2O+H]+ | C30H28O10 | Shikimates and Phenylpropanoids |
| **654** | 115 | Unknown | 0.61 | 445.212 | [M+H2O+H]+ | C27H26N2O3 | Alkaloids |
| **655** | 103 | Unknown | 0.60 | 547.1598 | [M+H]+ | C30H26O10 | Shikimates and Phenylpropanoids |
| **656** | 99 | Unknown | 0.60 | 563.1546 | [M+H]+ | C30H26O11 | Shikimates and Phenylpropanoids |
| **657** | 83 | Unknown | 0.60 | 579.1495 | [M+H]+ | C30H26O12 | Shikimates and Phenylpropanoids |
| **658** | 73 | Unknown | 0.59 | 147.0845 | [M-H2O+H]+ | C7H16O2S | Fatty acids |
| **659** | 67 | Unknown | 0.59 | 146.0812 | [M-H2O+H]+ | C6H13NO4 | Amino acids and Peptides |
| **660** | 61 | Unknown | 0.59 | 210.0793 | [M+H]+ | C7H15NO4S | Amino acids and Peptides |
| **661** | 10 | Unknown | 0.57 | 365.1052 | [M+Na]+ | C12H22O11 | Carbohydrates |

**Table S6.** Annotated compounds by GNPS on the samples analyzed by GC-MS.

| **#** | **Scan** | **Compound_Name** | **RT (s)** | **Precursor *m/z*** | **Molecular formula** | **Cosine score** | **Shared Peaks** | **Ontology** |
| --- | --- | --- | --- | --- | --- | --- | --- | --- |
| **1** | 14 | Methyl Caprylate | 452.4 | 158.13 | C_9_H_18_O_2_ | 0.82 | 28 | Fatty acids |
| **2** | 19 | Dihydrouracil | 471.6 | 114.04 | C_4_H_6_N_2_O_2_ | 0.91 | 57 | Pyrimidines |
| **3** | 22 | Sarcosine | 489.0 | 89.05 | C_3_H_7_NO_2_ | 0.74 | 9 | Aminoacids |
| **4** | 42 | Glycerol | 589.8 | 92.05 | C_3_H_8_O_3_ | 0.88 | 74 | Carbohydrates |
| **5** | 44 | Maltitol | 598.2 | 344.31 | C_12_H_24_O_11_ | 0.76 | 6 | Carbohydrates |
| **6** | 50 | Succinic Acid | 618.6 | 118.03 | C_4_H_6_O_4_ | 0.88 | 29 | Carboxylic Acids |
| **7** | 51 | Methyl Decanoate | 624.0 | 186.16 | C_11_H_22_O_2_ | 0.85 | 18 | Fatty acids |
| **8** | 53 | Heptane, 2,2,4,6,6-Pentamethyl- | 629.4 | 170.00 | C_12_H_26_ | 0.74 | 6 | Hydrocarbons |
| **9** | 56 | 2,3-Bis(Trimethylsilyl)Oxy-Propionic Acid Trimethylsilyl Ester | 637.8 | 322.15 | C_12_H_30_O_4_Si_3_ | 0.78 | 23 | Glycerides |
| **10** | 65 | Serine | 661.8 | 105.04 | C_3_H_7_NO_3_ | 0.83 | 17 | Aminoacids |
| **11** | 66 | Methylmalonic Acid | 672.6 | 118.03 | C_4_H_6_O_4_ | 0.87 | 49 | Carboxylic Acids |
| **12** | 68 | Threonine | 684.0 | 119.06 | C_4_H_9_NO_3_ | 0.68 | 8 | Aminoacids |
| **13** | 73 | Pentane, 3-Ethyl-2,2-Dimethyl- | 709.8 | 128.00 | C_9_H_20_ | 0.72 | 6 | Fatty acyls |
| **14** | 74 | 8-Methoxycarbonyl Octanal | 712.2 | 186.13 | C_10_H_18_O_3_ | 0.76 | 13 | Fatty acids |
| **15** | 79 | Decanoic Acid Trimethylsilyl Ester | 729.6 | 244.19 | C_13_H_28_O_2_Si | 0.87 | 7 | Fatty acids |
| **16** | 81 | Xylulose | 750.0 | 150.05 | C_5_H_10_O_5_ | 0.83 | 38 | Carbohydrates |
| **17** | 82 | Ribulose | 757.2 | 150.05 | C_5_H_10_O_5_ | 0.63 | 25 | Carbohydrates |
| **18** | 85 | Malic Acid | 759.0 | 134.02 | C_4_H_6_O_5_ | 0.81 | 21 | Carboxylic Acids |
| **19** | 86 | Malic Acid | 760.8 | 134.02 | C_4_H_6_O_5_ | 0.90 | 30 | Carboxylic Acids |
| **20** | 89 | 1-Trimethylsiloxy-2-Butene | 766.8 | 144.10 | C_7_H_16_OSi | 0.77 | 10 | Alcohols |
| **21** | 90 | Threitol | 774.0 | 122.06 | C_4_H_10_O_4_ | 0.81 | 17 | Glycerides |
| **22** | 93 | 10-Methylundecanoic Acid Methyl Ester | 778.2 | 214.19 | C_13_H_26_O_2_ | 0.88 | 35 | Fatty acids |
| **23** | 94 | Meso-Erythritol | 780.0 | 122.06 | C_4_H_10_O_4_ | 0.89 | 23 | Carbohydrates |
| **24** | 96 | Pyroglutamic Acid-Di-Tms | 784.8 | 273.12 | C_11_H_23_NO_3_Si_2_ | 0.65 | 21 | Alkaloids |
| **25** | 127 | Gluconic Acid Lactone | 865.8 | 178.05 | C_6_H_10_O_6_ | 0.67 | 13 | Carbohydrates |
| **26** | 133 | Arabinose | 888.6 | 150.05 | C_5_H_10_O_5_ | 0.79 | 10 | Carbohydrates |
| **27** | 136 | Ribose | 903.6 | 150.05 | C_5_H_10_O_5_ | 0.93 | 56 | Carbohydrates |
| **28** | 143 | Methyl Tetradecanoate | 916.8 | 242.00 | C_15_H_30_O_2_ | 0.84 | 53 | Fatty acids |
| **29** | 151 | 1-Octene | 928.8 | 112.13 | C_8_H_16_ | 0.86 | 8 | Hydrocarbons |
| **30** | 152 | Adonitol | 931.8 | 152.07 | C_5_H_12_O_5_ | 0.92 | 46 | Carbohydrates |

**Table S6.** Annotated compounds by GNPS on the samples analyzed by GC-MS. (cont.)

| **#** | **Scan** | **Compound_Name** | **RT (s)** | **Precursor *m/z*** | **Molecular formula** | **Cosine score** | **Shared Peaks** | **Ontology*** |
| --- | --- | --- | --- | --- | --- | --- | --- | --- |
| **31** | 154 | Fructose 6-Phosphate | 942.6 | 260.03 | C_6_H_13_O_9_P | 0.67 | 32 | Carbohydrates |
| **32** | 156 | Beta-Glucose | 947.4 | 180.06 | C_6_H_12_O_6_ | 0.79 | 29 | Carbohydrates |
| **33** | 164 | Uridine 5'-Diphospho-N-Acetylglucosamine | 958.2 | 607.08 | C_17_H_27_N_3_O_17_P_2_ | 0.79 | 71 | Nucleoside |
| **34** | 170 | N,N-Dimethyltryptamine | 970.8 | 188.13 | C_12_H_16_N_2_ | 0.85 | 52 | Alkaloids |
| **35** | 172 | Galactosamine | 976.2 | 179.08 | C_6_H_13_NO_5_ | 0.66 | 38 | Aminosugars |
| **36** | 175 | Inosine | 984.6 | 268.08 | C_10_H_12_N_4_O_5_ | 0.71 | 44 | Nucleoside |
| **37** | 177 | Uridine | 987.0 | 244.07 | C_9_H_12_N_2_O_6_ | 0.79 | 53 | Nucleoside |
| **38** | 182 | Citric Acid | 1002.0 | 192.03 | C_6_H_8_O_7_ | 0.62 | 27 | Carboxylic Acids |
| **39** | 191 | Quinic Acid | 1023.0 | 192.06 | C_7_H_12_O_6_ | 0.88 | 115 | Carboxylic Acids |
| **40** | 192 | Isocitric Acid | 1023.0 | 192.03 | C_6_H_8_O_7_ | 0.76 | 70 | Carboxylic Acids |
| **41** | 196 | Dehydroascorbic Acid | 1026.6 | 174.02 | C_6_H_6_O_6_ | 0.63 | 34 | Carboxylic Acids |
| **42** | 197 | Methylcyclohexane | 1026.6 | 98.11 | C_7_H_14_ | 0.68 | 13 | Hydrocarbons |
| **43** | 198 | Quinic Acid | 1031.4 | 192.06 | C_7_H_12_O_6_ | 0.78 | 270 | Carboxylic Acids |
| **44** | 199 | Fructose | 1037.4 | 180.06 | C_6_H_12_O_6_ | 0.94 | 138 | Carbohydrates |
| **45** | 200 | 13-Methyltetradecanoic Acid Methyl Ester | 1042.8 | 256.24 | C_16_H_32_O_2_ | 0.85 | 33 | Fatty acids |
| **46** | 201 | Galactose | 1042.8 | 180.06 | C_6_H_12_O_6_ | 0.92 | 124 | Carbohydrates |
| **47** | 202 | 4a-Methyldecahydro-2-Naphthol-2 | 1044.6 | 168.15 | C_11_H_20_O | 0.72 | 21 | Alcohols |
| **48** | 203 | Mannose | 1047.6 | 180.06 | C_6_H_12_O_6_ | 0.94 | 148 | Carbohydrates |
| **49** | 204 | Ascorbic Acid | 1052.4 | 176.03 | C_6_H_8_O_6_ | 0.82 | 46 | Carboxylic Acids |
| **50** | 206 | Glucose | 1059.0 | 180.06 | C_6_H_12_O_6_ | 0.94 | 133 | Carbohydrates |
| **51** | 210 | Iditol | 1066.8 | 182.08 | C_6_H_14_O_6_ | 0.85 | 52 | Carbohydrates |
| **52** | 211 | 4-Methylheptane | 1068.6 | 114.14 | C_8_H_18_ | 0.66 | 8 | Fatty acyls |
| **53** | 214 | Gallic Acid | 1072.8 | 170.02 | C_7_H_6_O_5_ | 0.74 | 25 | Shikimates and Phenylpropanoids |
| **54** | 223 | Trans-2-Octene | 1083.0 | 112.13 | C_8_H_16_ | 0.76 | 8 | Hydrocarbons |
| **55** | 224 | Hexatrimethylsilylinositol | 1087.2 | 612.30 | C_24_H_60_O_6_Si_6_ | 0.83 | 96 | Carbohydrates |
| **56** | 225 | Melibiose | 1092.0 | 342.12 | C_12_H_22_O_11_ | 0.84 | 55 | Carbohydrates |
| **57** | 226 | 1-Nonene | 1095.6 | 126.14 | C_9_H_18_ | 0.87 | 6 | Hydrocarbons |
| **58** | 228 | Inositol | 1098.6 | 180.06 | C_6_H_12_O_6_ | 0.73 | 60 | Carbohydrates |
| **59** | 229 | Glucose-1-Phosphate | 1103.4 | 260.03 | C_6_H_13_O_9_P | 0.72 | 34 | Carbohydrates |
| **60** | 233 | Palmitic Acid | 1113.6 | 256.24 | C_16_H_32_O_2_ | 0.93 | 147 | Fatty acids |

**Table S6.** Annotated compounds by GNPS on the samples analyzed by GC-MS. (cont.)

| **#** | **Scan** | **Compound_Name** | **RT (s)** | **Precursor *m/z*** | **Molecular formula** | **Cosine score** | **Shared Peaks** | **Ontology*** |
| --- | --- | --- | --- | --- | --- | --- | --- | --- |
| **61** | 235 | Hexadecanoic Acid Trimethylsilyl Ester | 1118.4 | 328.28 | C_19_H_40_O_2_Si | 0.86 | 10 | Fatty acids |
| **62** | 236 | 2-Methylheptane | 1120.8 | 114.14 | C_8_H_18_ | 0.75 | 6 | Hydrocarbons |
| **63** | 238 | Octadecanedioic Acid Bis(Trimethylsilyl) Ester | 1125.6 | 458.33 | C_24_H_50_O_4_Si_2_ | 0.75 | 7 | Fatty acyls |
| **64** | 242 | Myo-Inositol | 1143.0 | 180.06 | C_6_H_12_O_6_ | 0.88 | 111 | Carbohydrates |
| **65** | 249 | Palmitoleic Acid Trimethylsilyl Ester | 1154.4 | 326.26 | C_19_H_38_O_2_Si | 0.65 | 11 | Fatty acids |
| **66** | 251 | 1,2,3,4,5,6-Hexa-O-Trimethelsilyl-Myo-Inositol | 1158.6 | 612.30 | C_24_H_60_O_6_Si_6_ | 0.95 | 78 | Carbohydrates |
| **67** | 252 | Methyl Stearate | 1158.6 | 298.29 | C_19_H_38_O_2_ | 0.83 | 44 | Fatty acyls |
| **68** | 254 | Gentistic Acid | 1161.0 | 154.03 | C_7_H_6_O_4_ | 0.61 | 31 | Shikimates and Phenylpropanoids |
| **69** | 256 | 14-Methylhexadecanoic Acid Trimethylsilylester | 1167.6 | 342.30 | C_20_H_42_O_2_Si | 0.82 | 17 | Fatty acids |
| **70** | 262 | Phytol | 1186.2 | 296.31 | C_20_H_40_O | 0.91 | 72 | Terpenes |
| **71** | 266 | Linalool | 1195.8 | 154.14 | C_10_H_18_O | 0.67 | 6 | Terpenes |
| **72** | 267 | 2,4-Dimethyl-1-Heptene | 1195.8 | 126.14 | C_9_H_18_ | 0.85 | 6 | Hydrocarbons |
| **73** | 268 | Nervonic Acid Trimethylsilyl Ester | 1200.0 | 438.39 | C_27_H_54_O_2_Si | 0.71 | 24 | Fatty acids |
| **74** | 271 | 6,9,15-Octadecatrienoic Acid Methyl Ester | 1204.8 | 292.24 | C_19_H_32_O_2_ | 0.71 | 33 | Fatty acyls |
| **75** | 272 | Linoleic Acid | 1209.0 | 280.24 | C_18_H_32_O_2_ | 0.81 | 166 | Fatty acids |
| **76** | 273 | Linolenic Acid | 1209.0 | 278.23 | C_18_H_30_O_2_ | 0.94 | 182 | Fatty acids |
| **77** | 274 | Sebacic Acid | 1214.4 | 202.12 | C_10_H_18_O_4_ | 0.77 | 47 | Fatty acids |
| **78** | 275 | Stearic Acid | 1219.8 | 284.27 | C_18_H_36_O_2_ | 0.92 | 119 | Fatty acids |
| **79** | 277 | 9-Octadecenamide | 1228.2 | 281.00 | C_18_H_35_NO | 0.70 | 72 | Fatty acyls |
| **80** | 278 | Shikimic Acid | 1231.8 | 174.05 | C_7_H_10_O_5_ | 0.79 | 28 | Shikimates and Phenylpropanoids |
| **81** | 289 | Eicosanoic Acid, Methyl Ester | 1263.0 | 326.00 | C_21_H_42_O_2_ | 0.84 | 80 | Fatty acids |
| **82** | 290 | Oleic Acid | 1266.0 | 282.26 | C_18_H_34_O_2_ | 0.87 | 114 | Fatty acids |
| **83** | 306 | Dihydrosphingosine | 1302.0 | 301.30 | C_18_H_39_NO_2_ | 0.62 | 6 | Sphingolipidis |
| **84** | 309 | Gamma-Linolenic Acid | 1313.4 | 278.23 | C_18_H_30_O_2_ | 0.63 | 55 | Fatty acids |
| **85** | 311 | 18-Methylnonadecanoic Acid Trimethylsilylester | 1318.8 | 384.34 | C_23_H_48_O_2_Si | 0.84 | 41 | Fatty acids |
| **86** | 322 | 9-Heptacosene | 1345.8 | 378.42 | C_27_H_54_ | 0.77 | 35 | Fatty acyls |
| **87** | 323 | Cis-2-Octene | 1345.8 | 112.13 | C_8_H_16_ | 0.87 | 12 | Hydrocarbons |
| **88** | 329 | Nonadecanoic Acid Methyl Ester | 1359.6 | 312.30 | C_20_H_40_O_2_ | 0.80 | 74 | Fatty acids |
| **89** | 335 | Phytosphingosine | 1368.0 | 317.29 | C_18_H_39_NO_3_ | 0.73 | 18 | Sphingolipidis |

**Table S6.** Annotated compounds by GNPS on the samples analyzed by GC-MS. (cont.)

| **#** | **Scan** | **Compound_Name** | **RT (s)** | **Precursor *m/z*** | **Molecular formula** | **Cosine score** | **Shared Peaks** | **Ontology*** |
| --- | --- | --- | --- | --- | --- | --- | --- | --- |
| **90** | 340 | Sucrose | 1381.8 | 342.12 | C_12_H_22_O_11_ | 0.92 | 159 | Carbohydrates |
| **91** | 344 | Alpha-Hydroxybutyric Acid | 1396.8 | 104.05 | C_4_H_8_O_3_ | 0.75 | 22 | Fatty acyls |
| **92** | 353 | 1-Kestose | 1411.8 | 518.19 | C_19_H_34_O_16_ | 0.85 | 232 | Carbohydrates |
| **93** | 354 | Alpha-Lactose | 1419.6 | 342.12 | C_12_H_22_O_11_ | 0.90 | 56 | Carbohydrates |
| **94** | 359 | Trehalose | 1432.2 | 342.12 | C_12_H_22_O_11_ | 0.91 | 127 | Carbohydrates |
| **95** | 360 | 1-Kestose | 1438.8 | 504.17 | C_18_H_32_O_16_ | 0.86 | 248 | Carbohydrates |
| **96** | 361 | Maltose | 1444.2 | 342.12 | C_12_H_22_O_11_ | 0.86 | 43 | Carbohydrates |
| **97** | 366 | Monoolein | 1455.0 | 356.29 | C_21_H_40_O_4_ | 0.67 | 21 | Glycerides |
| **98** | 374 | Cis-11-Eicosenamide | 1474.8 | 309.00 | C_20_H_39_NO | 0.89 | 169 | Fatty acids |
| **99** | 376 | Nonane, 2,2,4,4,6,8,8-Heptamethyl- | 1476.6 | 226.00 | C_16_H_34_ | 0.73 | 34 | Hydrocarbons |
| **100** | 384 | Trans-11-Icosenamide | 1490.4 | 309.00 | C_20_H_39_NO | 0.71 | 211 | Fatty acids |
| **101** | 385 | Supraene | 1495.2 | 410.00 | C_30_H_50_ | 0.94 | 159 | Terpenes |
| **102** | 407 | Tetratriacontane | 1520.4 | 478.00 | C_34_H_70_ | 0.89 | 114 | Fatty acyls |
| **103** | 408 | Cis-4-Octene | 1525.2 | 112.13 | C_8_H_16_ | 0.90 | 14 | Hydrocarbons |
| **104** | 418 | Maltotriose | 1540.8 | 504.17 | C_18_H_32_O_16_ | 0.86 | 62 | Carbohydrates |
| **105** | 424 | 9,12,15-Octadecatrienoic Acid Methyl Ester | 1551.6 | 292.24 | C_19_H_32_O_2_ | 0.76 | 30 | Fatty acids |
| **106** | 428 | 1-Undecene | 1566.0 | 154.17 | C_11_H_22_ | 0.86 | 26 | Fatty acyls |
| **107** | 431 | Tryptophane | 1567.8 | 204.09 | C_11_H_12_N_2_O_2_ | 0.66 | 8 | Aminoacids |
| **108** | 433 | Clionasteryl-Acetate | 1571.4 | 456.40 | C_31_H_52_O_2_ | 0.67 | 69 | Terpenes |
| **109** | 434 | 1-(2,4,6-Trimethylphenoxy)-2-Aminopropane | 1571.4 | 193.15 | C_12_H_19_NO | 0.64 | 8 | Alkaloids |
| **110** | 440 | 3,7,11,15-Tetramethyl-2,6,10,14-Hexadecatetraene-1-Ol Trimethylsilyl Ether | 1578.0 | 362.30 | C_23_H_42_OSi | 0.73 | 30 | Terpenes |
| **111** | 442 | Maltotriose | 1579.8 | 504.17 | C_18_H_32_O_16_ | 0.67 | 19 | Carbohydrates |
| **112** | 444 | Behenic Acid | 1585.8 | 340.33 | C_22_H_44_O_2_ | 0.68 | 6 | Fatty acids |
| **113** | 446 | Octadecanoic Acid Trimethylsilyl Ester | 1585.8 | 356.31 | C_21_H_44_O_2_Si | 0.69 | 6 | Fatty acids |
| **114** | 460 | 3,7,11,15-Tetramethyl-1-Hexadecen-3-Ol | 1597.8 | 296.31 | C_20_H_40_O | 0.85 | 13 | Terpenes |
| **115** | 467 | Trehalose | 1611.6 | 342.12 | C_12_H_22_O_11_ | 0.61 | 16 | Carbohydrates |
| **116** | 487 | Tocopherol Acetate | 1653.6 | 472.39 | C_31_H_52_O_3_ | 0.76 | 66 | Terpenes |
| **117** | 492 | Alpha-Tocopherol | 1660.2 | 430.38 | C_29_H_50_O_2_ | 0.88 | 175 | Terpenes |
| **118** | 493 | Chlorogenic Acid | 1662.6 | 354.10 | C_16_H_18_O_9_ | 0.85 | 146 | Shikimates and Phenylpropanoids |

**Table S6.** Annotated compounds by GNPS on the samples analyzed by GC-MS. (cont.)

| **#** | **Scan** | **Compound_Name** | **RT (s)** | **Precursor *m/z*** | **Molecular formula** | **Cosine score** | **Shared Peaks** | **Ontology*** |
| --- | --- | --- | --- | --- | --- | --- | --- | --- |
| **119** | 494 | Guanosine | 1662.6 | 283.09 | C_10_H_13_N_5_O_5_ | 0.74 | 102 | Nucleoside |
| **120** | 496 | Alpha-Tocopherol | 1665.6 | 430.38 | C_29_H_50_O_2_ | 0.71 | 8 | Terpenes |
| **121** | 533 | Cis-5-Caffeoylquinic Acid | 1714.2 | 354.10 | C_16_H_18_O_9_ | 0.60 | 34 | Shikimates and Phenylpropanoids |
| **122** | 546 | Methyl O-Galactopyranoside | 1728.6 | 194.08 | C_7_H_14_O_6_ | 0.70 | 27 | Carbohydrates |
| **123** | 568 | 24-Methyl-5-Cholesten-3beta-Yl Trimethylsilyl Ether | 1747.8 | 472.41 | C_31_H_56_OSi | 0.89 | 122 | Terpenes |
| **124** | 587 | Trimethylsilyl Stigmasterol | 1771.8 | 484.41 | C_32_H_56_OSi | 0.86 | 212 | Terpenes |
| **125** | 591 | Heptadecanoic Acid | 1779.6 | 270.26 | C_17_H_34_O_2_ | 0.75 | 10 | Fatty acids |
| **126** | 627 | Raffinose | 1811.4 | 504.17 | C_18_H_32_O_16_ | 0.78 | 56 | Carbohydrates |
| **127** | 628 | 24-Ethyl-5-Cholesten-3beta-Yl Trimethylsilyl Ether | 1819.2 | 486.43 | C_32_H_58_OSi | 0.91 | 161 | Terpenes |
| **128** | 712 | Sedoheptulose | 1917.0 | 210.07 | C_7_H_14_O_7_ | 0.60 | 13 | Carbohydrates |
| **129** | 717 | 3Beta-Trimethylsiloxy-5alpha,6alpha-Epoxycholestane | 1939.2 | 474.39 | C_30_H_54_O_2_Si | 0.86 | 168 | Terpenes |
| **130** | 850 | Alpha-Phenylpropionic Acid Trimethylsilyl Ester | 2045.4 | 222.11 | C_12_H_18_O_2_Si | 0.64 | 7 | Shikimates and Phenylpropanoids |
| **131** | 873 | Oleanolic Acid | 2064.6 | 456.36 | C_30_H_48_O_3_ | 0.67 | 34 | Terpenes |
| **132** | 899 | Shikimic Acid | 2097.6 | 174.05 | C_7_H_10_O_5_ | 0.63 | 58 | Shikimates and Phenylpropanoids |

**Table S7.** Number of compounds by class in each sample analyzed by GC-MS.

|  | **Aqueous phase** | | |  | **Organic phase** | | |
| --- | --- | --- | --- | --- | --- | --- | --- |
| **Season** | **Fall** | **Winter** | **Summer** |  | **Fall** | **Winter** | **Summer** |
| Alcohols | 1 | 0 | 1 |  | 1 | 1 | 1 |
| Alkaloids | 2 | 1 | 2 |  | 3 | 2 | 2 |
| Aminoacids | 3 | 1 | 1 |  | 2 | 4 | 4 |
| Aminosugars | 0 | 0 | 0 |  | 1 | 1 | 1 |
| Carbohydrates | 32 | 25 | 30 |  | 32 | 33 | 32 |
| Carboxylic Acids | 8 | 7 | 8 |  | 8 | 7 | 7 |
| Fatty acids | 10 | 10 | 15 |  | 20 | 20 | 19 |
| Fatty acyls | 6 | 6 | 6 |  | 7 | 6 | 8 |
| Glycerides | 2 | 1 | 2 |  | 3 | 3 | 2 |
| Hydrocarbons | 3 | 1 | 4 |  | 9 | 6 | 9 |
| Nucleoside | 2 | 3 | 3 |  | 3 | 1 | 3 |
| Pyrimidines | 1 | 1 | 0 |  | 1 | 1 | 1 |
| Shikimates and Phenylpropanoids | 5 | 6 | 5 |  | 3 | 6 | 7 |
| Sphingolipidis | 2 | 1 | 2 |  | 2 | 1 | 2 |
| Terpenes | 10 | 11 | 11 |  | 14 | 11 | 12 |

**Table S8.** Mummichog pathway analysis result table for seasons differentiation.

| **Pathway names** | **Pathway total** | **Hits. total** | **Hits. sig** | **P(Fisher)** | **P(EASE)** | **AdjP. Fisher** | **AdjP. EASE** | **cpd. hits** |
| --- | --- | --- | --- | --- | --- | --- | --- | --- |
| Ascorbate and aldarate metabolism | 20 | 12 | 8 | 1.23E-04 | 7.78E-04 | 8.26E-03 | 0.0521 | EC0004, EC0005, EC00064, EC00065, EC00066, EC00067, EC00017, EC00018, EC00019, EC00020, EC000111, EC00082 |
| alpha-Linolenic acid metabolism | 26 | 15 | 11 | 6.08E-04 | 2.13E-03 | 0.0401 | 0.1408 | EC000329, EC000330, EC000393, EC000394, EC000210, EC000211, EC000323, EC000324, EC000325, EC000326, EC000327, EC000328, EC000445, EC000446, EC000321, EC000322 |
| Pentose and glucuronate interconversions | 17 | 11 | 7 | 9.28E-04 | 4.94E-03 | 0.0603 | 0.3208 | EC00064, EC00065, EC00066, EC00067, EC00082, EC00035, EC000388, EC000389, EC000390, EC000391 |
| Citrate cycle (TCA cycle) | 20 | 9 | 8 | 9.87E-04 | 7.03E-03 | 0.0631 | 0.4498 | EC0003, EC0008, EC000343, EC00056, EC00047, EC000111, EC00036, EC00037 |
| C5-Branched dibasic acid metabolism | 6 | 5 | 5 | 6.42E-03 | 0.0709 | 0.4045 | 1 | EC000271, EC000258, EC000311, EC00032 |
| Inositol phosphate metabolism | 28 | 8 | 2 | 8.16E-03 | 0.0447 | 0.5060 | 1 | EC0004, EC0005, EC00064, EC00065, EC00066, EC00067 |
| Glyoxylate and dicarboxylate metabolism | 29 | 11 | 8 | 0.0152 | 0.0589 | 0.9319 | 1 | EC000111, EC00056, EC00060, EC00047, EC00084, EC0003, EC0008 |
| Glutathione metabolism | 26 | 6 | 3 | 0.0187 | 0.0779 | 1 | 1 | EC00041, EC00017, EC00018, EC00019, EC00020, EC000111 |
| Butanoate metabolism | 17 | 8 | 6 | 0.0223 | 0.1039 | 1 | 1 | EC00032, EC0003, EC0008, EC000311, EC000271 |
| Betalain biosynthesis | 3 | 2 | 2 | 0.0228 | 0.1947 | 1 | 1 | EC00093, EC00094, EC000304 |
| Linoleic acid metabolism | 4 | 3 | 3 | 0.0721 | 0.3186 | 1 | 1 | EC000231, EC000321, EC000322 |
| Amino sugar and nucleotide sugar metabolism | 52 | 25 | 8 | 0.0777 | 0.1768 | 1 | 1 | EC00064, EC00065, EC00066, EC00067, EC0004, EC0005, EC00035, EC00090 |
| Galactose metabolism | 27 | 21 | 15 | 0.1237 | 0.3052 | 1 | 1 | EC000128, EC00029, EC000346, EC0004, EC0005 |
| Alanine, aspartate and glutamate metabolism | 22 | 9 | 4 | 0.1237 | 0.3052 | 1 | 1 | EC00032, EC0003, EC00036, EC00037, EC0008 |
| Arachidonic acid metabolism | 9 | 4 | 3 | 0.1428 | 0.4372 | 1 | 1 | EC000187, EC000254, EC000153 |

**Table S8.** Mummichog pathway analysis result table for seasons differentiation. (cont.)

| **Pathway names** | **Pathway total** | **Hits. total** | **Hits. sig** | **P(Fisher)** | **P(EASE)** | **AdjP. Fisher** | **AdjP. EASE** | **cpd. hits** |
| --- | --- | --- | --- | --- | --- | --- | --- | --- |
| Porphyrin metabolism | 48 | 22 | 11 | 0.1679 | 0.2753 | 1 | 1 | EC000338, EC000132, EC000521, EC000523, EC000502, EC000559, EC000363, EC000376, EC000524, EC000440, EC000364, EC000365 |
| Cutin, suberine and wax biosynthesis | 18 | 9 | 5 | 0.1804 | 0.3603 | 1 | 1 | EC000538, EC000541, EC000400, EC000401, EC000526, EC000527 |
| Amino acid metabolism | 7 | 5 | 3 | 0.1968 | 0.6325 | 1 | 1 | EC000158, EC00010 |
| Propanoate metabolism | 19 | 4 | 3 | 0.1968 | 0.6325 | 1 | 1 | EC00032, EC0008 |
| Lysine biosynthesis | 9 | 7 | 4 | 0.2275 | 0.5437 | 1 | 1 | EC000158, EC000553, EC00010 |
| Starch and sucrose metabolism | 22 | 13 | 7 | 0.2353 | 0.4924 | 1 | 1 | EC00029, EC0004, EC0005, EC000128 |
| Riboflavin metabolism | 13 | 6 | 3 | 0.2353 | 0.4924 | 1 | 1 | EC00079, EC00080, EC000313, EC000217 |
| Sesquiterpenoid and triterpenoid biosynthesis | 24 | 15 | 14 | 0.2353 | 0.4924 | 1 | 1 | EC000200, EC000297, EC000548, EC000549 |
| Cyanoamino acid metabolism | 29 | 16 | 7 | 0.3192 | 0.4898 | 1 | 1 | EC000229, EC000144, EC000403, EC00026, EC00093, EC00094, EC000121, EC000268 |
| Glycolysis / Gluconeogenesis | 26 | 7 | 2 | 0.3216 | 0.7371 | 1 | 1 | EC0004, EC0005 |
| Fructose and mannose metabolism | 18 | 8 | 3 | 0.3216 | 0.7371 | 1 | 1 | EC0004, EC0005 |
| Arginine biosynthesis | 18 | 9 | 3 | 0.3860 | 0.6445 | 1 | 1 | EC000114, EC0003, EC00036, EC00037 |
| Biosynthesis of unsaturated fatty acids | 22 | 8 | 4 | 0.3860 | 0.6445 | 1 | 1 | EC000231, EC000393, EC000394, EC000398 |
| Tyrosine metabolism | 17 | 14 | 6 | 0.4211 | 0.5924 | 1 | 1 | EC000348, EC00093, EC00094, EC00026, EC000304, EC00036, EC00037, EC00032 |
| Histidine metabolism | 16 | 2 | 1 | 0.4891 | 1 | 1 | 1 | EC000320 |
| N-Glycan biosynthesis | 35 | 2 | 1 | 0.4891 | 1 | 1 | 1 | EC000214 |
| Glycerolipid metabolism | 21 | 2 | 1 | 0.4891 | 1 | 1 | 1 | EC00084 |
| Lipoic acid metabolism | 24 | 2 | 1 | 0.4891 | 1 | 1 | 1 | EC0003 |
| Glycine, serine and threonine metabolism | 33 | 11 | 3 | 0.4972 | 0.7746 | 1 | 1 | EC00084, EC00060, EC00032 |
| Pyruvate metabolism | 23 | 5 | 2 | 0.4972 | 0.7746 | 1 | 1 | EC00047, EC00036, EC00037 |
| Tryptophan metabolism | 29 | 14 | 6 | 0.5262 | 0.7133 | 1 | 1 | EC00089, EC000149, EC000151, EC000190, EC000191, EC000233 |

**Table S8.** Mummichog pathway analysis result table for seasons differentiation. (cont.)

| **Pathway names** | **Pathway total** | **Hits. total** | **Hits. sig** | **P(Fisher)** | **P(EASE)** | **AdjP. Fisher** | **AdjP. EASE** | **cpd. hits** |
| --- | --- | --- | --- | --- | --- | --- | --- | --- |
| Isoquinoline alkaloid biosynthesis | 6 | 6 | 3 | 0.5328 | 0.7616 | 1 | 1 | EC00026, EC00093, EC00094, EC000304 |
| Flavone and flavonol biosynthesis | 10 | 10 | 8 | 0.5438 | 0.6927 | 1 | 1 | EC000224, EC000102, EC000241, EC000506, EC000353, EC000355, EC000356, EC000357, EC000547 |
| Steroid biosynthesis | 44 | 27 | 14 | 0.5596 | 0.7571 | 1 | 1 | EC000200, EC000444, EC000245, EC000569, EC000570 |
| Flavonoid biosynthesis | 47 | 39 | 34 | 0.5795 | 0.6791 | 1 | 1 | EC000137, EC000290, EC000433, EC000181, EC000182, EC000224, EC000374, EC000370, EC000371, EC000299, EC000302, EC000195, EC000423, EC000235, EC000282, EC000102, EC000166, EC000169 |
| Valine, leucine and isoleucine biosynthesis | 22 | 15 | 6 | 0.7145 | 0.8770 | 1 | 1 | EC000271, EC000258, EC000311, EC00032 |
| Valine, leucine and isoleucine degradation | 37 | 10 | 4 | 0.7157 | 0.9317 | 1 | 1 | EC00032, EC0008 |
| Terpenoid backbone biosynthesis | 30 | 6 | 2 | 0.7157 | 0.9317 | 1 | 1 | EC000442, EC000297 |
| Monobactam biosynthesis | 8 | 4 | 1 | 0.7397 | 1 | 1 | 1 | EC000553 |
| Pentose phosphate pathway | 19 | 13 | 2 | 0.7781 | 0.9513 | 1 | 1 | EC00035, EC00082 |
| Fatty acid biosynthesis | 56 | 5 | 1 | 0.8144 | 1 | 1 | 1 | EC00060 |
| Purine metabolism | 73 | 19 | 4 | 0.8387 | 0.9401 | 1 | 1 | EC000320, EC000331, EC000315, EC000571 |
| Ubiquinone and other terpenoid-quinone biosynthesis | 47 | 22 | 5 | 0.8554 | 0.9300 | 1 | 1 | EC00051, EC00052, EC00055, EC00026, EC000261, EC000366, EC000294 |
| Carotenoid biosynthesis | 43 | 17 | 5 | 0.8617 | 0.9434 | 1 | 1 | EC000474, EC000385, EC000556, EC000478, EC000479 |
| Glycerophospholipid metabolism | 38 | 5 | 1 | 0.8677 | 1 | 1 | 1 | EC000159 |
| Folate biosynthesis | 31 | 7 | 1 | 0.8677 | 1 | 1 | 1 | EC000441 |
| Indole alkaloid biosynthesis | 4 | 3 | 1 | 0.8677 | 1 | 1 | 1 | EC000250 |
| Sulfur metabolism | 15 | 6 | 1 | 0.8677 | 1 | 1 | 1 | EC0008 |
| Pyrimidine metabolism | 41 | 14 | 2 | 0.8992 | 0.9824 | 1 | 1 | EC00086, EC000121 |
| Carbon fixation in photosynthetic organisms | 21 | 8 | 1 | 0.9058 | 1 | 1 | 1 | EC00047 |

**Table S8.** Mummichog pathway analysis result table for seasons differentiation. (cont.)

| **Pathway names** | **Pathway total** | **Hits. total** | **Hits. sig** | **P(Fisher)** | **P(EASE)** | **AdjP. Fisher** | **AdjP. EASE** | **cpd. hits** |
| --- | --- | --- | --- | --- | --- | --- | --- | --- |
| Pantothenate and CoA biosynthesis | 25 | 7 | 1 | 0.9058 | 1 | 1 | 1 | EC000311 |
| Biosynthesis of various plant secondary metabolites | 29 | 14 | 5 | 0.9238 | 0.9721 | 1 | 1 | EC000200, EC000367, EC000317, EC000318, EC000532 |
| Lysine degradation | 20 | 6 | 1 | 0.9330 | 1 | 1 | 1 | EC00010 |
| Diterpenoid biosynthesis | 29 | 9 | 1 | 0.9330 | 1 | 1 | 1 | EC000187 |
| Sphingolipid metabolism | 27 | 6 | 1 | 0.9662 | 1 | 1 | 1 | EC000292 |
| Cysteine and methionine metabolism | 47 | 12 | 1 | 0.9829 | 1 | 1 | 1 | EC00032 |
| Glucosinolate biosynthesis | 65 | 17 | 4 | 0.9909 | 0.9983 | 1 | 1 | EC000510, EC000564, EC000268 |
| Phenylalanine metabolism | 12 | 8 | 1 | 0.9957 | 1 | 1 | 1 | EC000268 |
| Brassinosteroid biosynthesis | 26 | 14 | 1 | 0.9957 | 1 | 1 | 1 | EC000486 |
| Phenylpropanoid biosynthesis | 43 | 21 | 4 | 0.9964 | 0.9989 | 1 | 1 | EC000290, EC000181, EC000182, EC000433, EC000279, EC000280 |
| Arginine and proline metabolism | 32 | 13 | 1 | 0.9969 | 1 | 1 | 1 | EC000380 |
| Phenylalanine, tyrosine and tryptophan biosynthesis | 22 | 12 | 1 | 0.9999 | 1 | 1 | 1 | EC00026 |

**Table S9.** Mummichog pathway analysis result table for cultivation differentiation.

| **Pathway names** | **Pathway total** | **Hits. total** | **Hits. sig** | **P(Fisher)** | **P(EASE)** | **AdjP. Fisher** | **AdjP. EASE** | **cpd.hits** |
| --- | --- | --- | --- | --- | --- | --- | --- | --- |
| Flavonoid biosynthesis | 47 | 39 | 39 | 2.87E-07 | 1.37E-06 | 1.95E-05 | 9.34E-05 | EC000109, EC000110, EC000111, EC000320, EC000321, EC000215, EC000296, EC000297, EC000143, EC000144, EC000173, EC000174, EC000175, EC000264, EC000266, EC000263, EC000221, EC000222, EC000223, EC000150, EC000151, EC000289, EC000290, EC000179, EC000180, EC000181, EC000182, EC000209, EC000210, EC000211, EC000212, EC000169, EC00087, EC00088, EC00089, EC00090, EC000133, EC000134, EC000135, EC000136, EC000294, EC000295, EC000334 |
| Flavone and flavonol biosynthesis | 10 | 10 | 10 | 1.86E-03 | 6.95E-03 | 0.1250 | 0.4659 | EC000173, EC000174, EC000175, EC000169, EC00087, EC00088, EC00089, EC00090, EC000184, EC000185, EC000340, EC000341, EC000342, EC000253, EC000254, EC000255, EC000362, EC000363, EC000373, EC000374 |
| Pentose phosphate pathway | 19 | 13 | 13 | 0.0065 | 0.0513 | 0.4289 | 1 | EC00020, EC000188, EC00025, EC00026, EC000246, EC00076, EC00068, EC000129 |
| C5-Branched dibasic acid metabolism | 6 | 5 | 5 | 0.0822 | 0.3652 | 1 | 1 | EC000201, EC000195, EC000230, EC00023 |
| Folate biosynthesis | 31 | 5 | 5 | 0.0822 | 0.3652 | 1 | 1 | EC00063, EC000300, EC00022, EC000142 |
| Terpenoid backbone biosynthesis | 30 | 5 | 4 | 0.0887 | 0.2851 | 1 | 1 | EC000129, EC000301, EC000218, EC000219, EC000220, EC000364 |
| Valine, leucine and isoleucine biosynthesis | 22 | 15 | 13 | 0.0970 | 0.2182 | 1 | 1 | EC000201, EC000195, EC00030, EC000196, EC000197, EC000188, EC000230, EC00023, EC000106, EC000107, EC00050 |

**Table S9.** Mummichog pathway analysis result table for cultivation differentiation. (cont.)

| **Pathway names** | **Pathway total** | **Hits. total** | **Hits. sig** | **P(Fisher)** | **P(EASE)** | **AdjP. Fisher** | **AdjP. EASE** | **cpd.hits** |
| --- | --- | --- | --- | --- | --- | --- | --- | --- |
| Phenylpropanoid biosynthesis | 43 | 18 | 15 | 0.1213 | 0.2015 | 1 | 1 | EC000250, EC000171, EC000320, EC000321, EC000215, EC000143, EC000144, EC000315, EC000317, EC000251, EC000252, EC000158, EC000160, EC000161, EC00047, EC00048, EC000318, EC000157, EC000296, EC000297, EC000207, EC000208 |
| Pyrimidine metabolism | 41 | 10 | 7 | 0.1292 | 0.3243 | 1 | 1 | EC00071, EC00039, EC00024, EC000101, EC00080, EC00079, EC00058 |
| Monobactam biosynthesis | 8 | 3 | 3 | 0.1539 | 0.5519 | 1 | 1 | EC0007, EC000365, EC000228 |
| beta-Alanine metabolism | 18 | 3 | 3 | 0.1539 | 0.5519 | 1 | 1 | EC00019, EC0007, EC00039 |
| Nicotinate and nicotinamide metabolism | 13 | 3 | 3 | 0.1539 | 0.5519 | 1 | 1 | EC0007, EC00064, EC00040 |
| Sesquiterpenoid and triterpenoid biosynthesis | 24 | 1 | 1 | 0.1539 | 0.5519 | 1 | 1 | EC000218, EC000219, EC000220 |
| Riboflavin metabolism | 13 | 5 | 4 | 0.1962 | 0.4403 | 1 | 1 | EC00025, EC000235, EC00065, EC00066, EC00067, EC000232 |
| Caffeine metabolism | 10 | 4 | 4 | 0.2879 | 0.7840 | 1 | 1 | EC000279, EC000333 |
| Glycerolipid metabolism | 21 | 2 | 2 | 0.2879 | 0.7840 | 1 | 1 | EC00069, EC00020 |
| Lysine degradation | 20 | 6 | 5 | 0.2901 | 0.5759 | 1 | 1 | EC00097, EC000213, EC00092, EC000149, EC00098 |
| Butanoate metabolism | 17 | 8 | 6 | 0.2901 | 0.5759 | 1 | 1 | EC0001, EC00023, EC0002, EC000230, EC000201 |
| Anthocyanin biosynthesis | 11 | 4 | 4 | 0.2901 | 0.5759 | 1 | 1 | EC000312, EC000285, EC000286, EC000366, EC000367 |
| Lysine biosynthesis | 9 | 7 | 5 | 0.4151 | 0.7202 | 1 | 1 | EC000127, EC0007, EC000365, EC000228 |
| Carbon fixation in photosynthetic organisms | 21 | 7 | 6 | 0.4151 | 0.7202 | 1 | 1 | EC0007, EC000246, EC00025, EC00020 |
| Pantothenate and CoA biosynthesis | 25 | 6 | 4 | 0.4151 | 0.7202 | 1 | 1 | EC00039, EC000188, EC000230, EC00050 |
| Glycine, serine and threonine metabolism | 33 | 11 | 5 | 0.4465 | 0.7114 | 1 | 1 | EC00069, EC0007, EC00050, EC00049, EC00023 |

**Table S9.** Mummichog pathway analysis result table for cultivation differentiation. (cont.)

| **Pathway names** | **Pathway total** | **Hits. total** | **Hits. sig** | **P(Fisher)** | **P(EASE)** | **AdjP. Fisher** | **AdjP. EASE** | **cpd.hits** |
| --- | --- | --- | --- | --- | --- | --- | --- | --- |
| Biosynthesis of various plant secondary metabolites | 29 | 10 | 8 | 0.4474 | 0.6230 | 1 | 1 | EC000202, EC000203, EC000261, EC000186, EC000292, EC000293, EC000244, EC00047, EC00048, EC000375, EC000354 |
| Taurine and hypotaurine metabolism | 5 | 1 | 1 | 0.5372 | 1 | 1 | 1 | EC000262 |
| N-Glycan biosynthesis | 35 | 1 | 1 | 0.5372 | 1 | 1 | 1 | EC00020 |
| Thiamine metabolism | 22 | 1 | 1 | 0.5372 | 1 | 1 | 1 | EC000129 |
| Lipoic acid metabolism | 24 | 1 | 1 | 0.5372 | 1 | 1 | 1 | EC0002 |
| Tyrosine metabolism | 17 | 14 | 10 | 0.5468 | 0.7091 | 1 | 1 | EC000249, EC000200, EC000112, EC000158, EC000160, EC000161, EC000225, EC00023, EC000226, EC000227, EC000229 |
| Fructose and mannose metabolism | 18 | 8 | 5 | 0.5559 | 0.9001 | 1 | 1 | EC000139, EC00020 |
| Fatty acid biosynthesis | 56 | 3 | 2 | 0.5559 | 0.9001 | 1 | 1 | EC00019, EC00049 |
| Propanoate metabolism | 19 | 4 | 3 | 0.5559 | 0.9001 | 1 | 1 | EC00023, EC00019 |
| Vitamin B6 metabolism | 12 | 3 | 2 | 0.5559 | 0.9001 | 1 | 1 | EC00025, EC00072 |
| Phenylalanine, tyrosine and tryptophan biosynthesis | 22 | 12 | 8 | 0.5576 | 0.7113 | 1 | 1 | EC00063, EC00047, EC00048, EC000106, EC000107, EC000147, EC00022, EC000158, EC000160, EC000161, EC000202, EC000203 |
| Arginine biosynthesis | 18 | 9 | 6 | 0.6012 | 0.8014 | 1 | 1 | EC0001, EC00096, EC000165, EC000121, EC0007, EC0002 |
| Cysteine and methionine metabolism | 47 | 11 | 6 | 0.6012 | 0.8014 | 1 | 1 | EC000156, EC0001, EC000149, EC0007, EC00023, EC000244 |
| Glyoxylate and dicarboxylate metabolism | 29 | 11 | 7 | 0.6012 | 0.8014 | 1 | 1 | EC00093, EC00045, EC00049, EC00069, EC0001, EC0002 |
| Ascorbate and aldarate metabolism | 20 | 12 | 10 | 0.7122 | 0.8606 | 1 | 1 | EC000154, EC00020, EC00056, EC00011, EC00093, EC00068, EC000140 |
| Amino sugar and nucleotide sugar metabolism | 52 | 25 | 15 | 0.7122 | 0.8606 | 1 | 1 | EC00056, EC00020, EC000153, EC00026, EC00025, EC00074, EC00034 |

**Table S9.** Mummichog pathway analysis result table for cultivation differentiation. (cont.)

| **Pathway names** | **Pathway total** | **Hits. total** | **Hits. sig** | **P(Fisher)** | **P(EASE)** | **AdjP. Fisher** | **AdjP. EASE** | **cpd.hits** |
| --- | --- | --- | --- | --- | --- | --- | --- | --- |
| Porphyrin metabolism | 48 | 13 | 6 | 0.7122 | 0.8606 | 1 | 1 | EC000308, EC000193, EC0001, EC000339, EC000132, EC000370, EC000371 |
| Alanine, aspartate and glutamate metabolism | 22 | 9 | 5 | 0.7131 | 0.8822 | 1 | 1 | EC0007, EC00039, EC00023, EC0002, EC0001 |
| Isoquinoline alkaloid biosynthesis | 6 | 6 | 3 | 0.7131 | 0.8822 | 1 | 1 | EC000225, EC000158, EC000160, EC000161, EC000229 |
| Glutathione metabolism | 26 | 5 | 4 | 0.7168 | 0.8987 | 1 | 1 | EC0001, EC000191, EC00011, EC00093 |
| Starch and sucrose metabolism | 22 | 13 | 6 | 0.7248 | 0.9215 | 1 | 1 | EC000130, EC000131, EC00020 |
| Glycerophospholipid metabolism | 38 | 5 | 3 | 0.7248 | 0.9215 | 1 | 1 | EC000118, EC000164, EC000162 |
| Diterpenoid biosynthesis | 29 | 7 | 3 | 0.7423 | 0.9539 | 1 | 1 | EC000311, EC000310 |
| Sulfur metabolism | 15 | 4 | 2 | 0.7423 | 0.9539 | 1 | 1 | EC0001, EC000156 |
| Glycolysis / Gluconeogenesis | 26 | 6 | 4 | 0.7865 | 1 | 1 | 1 | EC00020 |
| Fatty acid elongation | 23 | 2 | 1 | 0.7865 | 1 | 1 | 1 | EC00019 |
| Nitrogen metabolism | 12 | 2 | 1 | 0.7865 | 1 | 1 | 1 | EC0001 |
| Pentose and glucuronate interconversions | 17 | 11 | 10 | 0.7996 | 0.9174 | 1 | 1 | EC00056, EC00020, EC00068, EC00025, EC00026, EC00083 |
| Pyruvate metabolism | 23 | 4 | 2 | 0.8322 | 0.9587 | 1 | 1 | EC000196, EC000197, EC00019 |
| Ubiquinone and other terpenoid-quinone biosynthesis | 47 | 12 | 7 | 0.8495 | 0.9293 | 1 | 1 | EC000338, EC00063, EC00042, EC000158, EC000160, EC000161, EC00047, EC00048, EC000112 |
| Phenylalanine metabolism | 12 | 8 | 4 | 0.8739 | 0.9576 | 1 | 1 | EC00047, EC00048, EC000229, EC000226, EC000227 |
| Citrate cycle (TCA cycle) | 20 | 7 | 4 | 0.9014 | 0.9787 | 1 | 1 | EC0002, EC00045, EC00093 |
| Valine, leucine and isoleucine degradation | 37 | 10 | 6 | 0.9014 | 0.9787 | 1 | 1 | EC00050, EC00030, EC00023 |
| D-Amino acid metabolism | 7 | 5 | 2 | 0.9018 | 1 | 1 | 1 | EC000127 |
| Betalain biosynthesis | 3 | 2 | 1 | 0.9018 | 1 | 1 | 1 | EC000225 |
| Inositol phosphate metabolism | 28 | 6 | 5 | 0.9234 | 0.9903 | 1 | 1 | EC00020, EC00056 |

**Table S9.** Mummichog pathway analysis result table for cultivation differentiation. (cont.)

| **Pathway names** | **Pathway total** | **Hits. total** | **Hits. sig** | **P(Fisher)** | **P(EASE)** | **AdjP. Fisher** | **AdjP. EASE** | **cpd.hits** |
| --- | --- | --- | --- | --- | --- | --- | --- | --- |
| Tropane, piperidine and pyridine alkaloid biosynthesis | 9 | 3 | 1 | 0.9234 | 0.9903 | 1 | 1 | EC00047, EC00048 |
| Cyanoamino acid metabolism | 29 | 15 | 7 | 0.9326 | 0.9737 | 1 | 1 | EC00039, EC000176, EC000177, EC000117, EC000283, EC0001, EC000101, EC0007 |
| Glucosinolate biosynthesis | 65 | 15 | 6 | 0.9326 | 0.9737 | 1 | 1 | EC000345, EC000346, EC000347, EC000348, EC000349, EC000350. EC00050, EC00030 |
| Arginine and proline metabolism | 32 | 13 | 7 | 0.9439 | 0.9817 | 1 | 1 | EC00092, EC0001, EC000191, EC000213 , EC000298, EC000269 |
| Indole alkaloid biosynthesis | 4 | 3 | 1 | 0.9549 | 1 | 1 | 1 | EC000190 |
| Galactose metabolism | 27 | 21 | 5 | 0.9599 | 0.9956 | 1 | 1 | EC00020, EC000139 |
| Tryptophan metabolism | 29 | 13 | 4 | 0.9909 | 0.9987 | 1 | 1 | EC00073, EC000178, EC000257 |
| alpha-Linolenic acid metabolism | 26 | 13 | 8 | 0.9909 | 0.9987 | 1 | 1 | EC000241, EC000163, EC000284 |
| Purine metabolism | 73 | 18 | 3 | 0.9999 | 1 | 1 | 1 | EC00025, EC00084 |

**Table S10.** Environmental conditions across 2019 in Paulínia, SP, Brazil.

|  | **Temperature (°C)** | | **Air Humidity (%)** | | **Precipitation (mm)** |
| --- | --- | --- | --- | --- | --- |
|  | **Average Minimum** | **Average Maximum** | **Average Minimum** | **Average Maximum** |  |
| **Month-Year** | **T min (°C)** | **T max (°C)** | **H min (%)** | **H max (%)** |  |
| Jan-19 | 21.13 | 33.73 | 44.91 | 95.04 | 199.66 |
| Feb-19 | 20.23 | 30.89 | 54.62 | 96.06 | 155.17 |
| Mar-19 | 19.69 | 31.05 | 53.99 | 98.01 | 62.74 |
| Apr-19 | 18.87 | 30.63 | 49.72 | 97.37 | 4.57 |
| May-19 | 16.38 | 28.13 | 53.38 | 98.47 | 14.48 |
| Jun-19 | 13.95 | 27.17 | 44.99 | 96.39 | 0 |
| Jul-19 | 11.68 | 26.07 | 41.62 | 94.42 | 29.21 |
| Aug-19 | 13.39 | 27.36 | 42.17 | 92.88 | 2.03 |
| Sep-19 | 16.87 | 30.61 | 43.50 | 90.92 | 11.94 |
| Oct-19 | 18.91 | 33.40 | 36.20 | 90.89 | 3.30 |
| Nov-19 | 19.13 | 30.66 | 51.56 | 95.07 | 94.98 |
| Dec-19 | 19.80 | 30.87 | 52.69 | 97.08 | 176.03 |

Note: "Zero" lines indicate that there was a failure in the collection of the corresponding month.

**Table S11.** Injection order applied for the metabolomics and lipidomics assays in both ionization modes.

| **Injection Number** | **Sample Name** | **Injection Number** | **Sample Name** | **Injection Number** | **Sample Name** | **Injection Number** | **Sample Name** |
| --- | --- | --- | --- | --- | --- | --- | --- |
| 1 | BCor | 23 | SA1A_N | 45 | FC1A_N | 67 | SJ1A_N |
| 2 | BExt | 24 | SC6A_N | 46 | FA6A_N | 68 | FG6A_N |
| 3 | QCEq_1 | 25 | FL1A_N | 47 | FA1A_N | 69 | FJ1A_N |
| 4 | QCEq_2 | 26 | WH1A_N | 48 | FG1A_N | 70 | SB6A_N |
| 5 | QCEq_3 | 27 | SK1A_N | 49 | SF6A_N | 71 | FF6A_N |
| 6 | QCEq_4 | 28 | FC6A_N | 50 | FD6A_N | 72 | SB1A_N |
| 7 | QC1_N | 29 | QC3_N | 51 | FB6A_N | 73 | WA1A_N |
| 8 | SL1A_N | 30 | FI1A_N | 52 | WL1A_N | 74 | SC1A_N |
| 9 | SG1A_N | 31 | WJ6A_N | 53 | QC5_N | 75 | QC7_N |
| 10 | WE1A_N | 32 | FJ6A_N | 54 | SF1A_N | 76 | WF6A_N |
| 11 | FH1A_N | 33 | FE6A_N | 55 | WD1A_N | 77 | WI1A_N |
| 12 | WJ1A_N | 34 | WB6A_N | 56 | WH6A_N | 78 | FF1A_N |
| 13 | WE6A_N | 35 | SH1A_N | 57 | FI6A_N | 79 | WI6A_N |
| 14 | WL6A_N | 36 | WG6A_N | 58 | FD1A_N | 80 | WG1A_N |
| 15 | FH6A_N | 37 | SI6A_N | 59 | SE1A_N | 81 | FE1A_N |
| 16 | FL6A_N | 38 | SG6A_N | 60 | SJ6A_N | 82 | SA6A_N |
| 17 | SD6A_N | 39 | WF1A_N | 61 | WD6A_N | 83 | SI1A_N |
| 18 | QC2_N | 40 | FK6A_N | 62 | FB1A_N | 84 | WC6A_N |
| 19 | SK6A_N | 41 | QC4_N | 63 | SL6A_N | 85 | SH6A_N |
| 20 | FK1A_N | 42 | WK6A_N | 64 | QC6_N | 86 | QC8_N |
| 21 | SE6A_N | 43 | SD1A_N | 65 | WB1A_N | 87 | BExt |
| 22 | WC1A_N | 44 | WK1A_N | 66 | WA6A_N | 88 | BCor |
|  |  |  |  |  |  |  |  |
|  | Blank samples |  |  |  |  |  |  |
|  | System suitability samples | |  |  |  |  |  |
|  | Pooled QC samples | |  |  |  |  |  |
|  | Experimental samples | |  |  |  |  |  |

**Table S12.** Features of aqueous phase with their respective VIP (PLS-DA). VIP > 1.60 based on positive ionization mode.

| **VIP based on seasons** | | | | **VIP based on cultivation condition** | | | |
| --- | --- | --- | --- | --- | --- | --- | --- |
| **Negative ionization mode** | | **Positive ionization mode** | | **Negative ionization mode** | | **Positive ionization mode** | |
| **RT_*m/z*** | **VIP** | **RT_*m/z*** | **VIP** | **RT_*m/z*** | **VIP** | **RT_*m/z*** | **VIP** |
| 8.969_607.29932 | 2.23 | 0.628_235.02158 | 1.99 | 7.468_453.19827 | 1.89 | 8.95_325.22769 | 1.93 |
| 0.63_425.06979 | 2.06 | 0.615_272.97717 | 1.98 | 6.177_672.14148 | 1.88 | 0.743_202.14407 | 1.92 |
| 5.674_751.15314 | 2.05 | 5.767_365.20654 | 1.85 | 6.169_647.11963 | 1.88 | 7.9_287.05429 | 1.89 |
| 6.843_661.15765 | 2.05 | 7.429_273.15973 | 1.83 | 6.168_677.133 | 1.87 | 6.809_625.17609 | 1.88 |
| 6.256_269.13934 | 2.03 | 8.909_369.25369 | 1.79 | 6.173_708.06287 | 1.87 | 7.896_433.1123 | 1.86 |
| 6.994_305.16135 | 2.02 | 0.727_474.21985 | 1.77 | 6.182_152.16356 | 1.87 | 6.162_333.04291 | 1.86 |
| 6.553_215.12909 | 2.01 | 4.824_276.17041 | 1.77 | 6.902_665.13745 | 1.86 | 5.804_781.26013 | 1.86 |
| 0.637_195.05103 | 1.99 | 6.437_228.10167 | 1.77 | 6.169_645.12341 | 1.86 | 5.114_212.1283 | 1.85 |
| 9.484_445.24451 | 1.98 | 0.629_293.06296 | 1.76 | 6.174_373.1514 | 1.85 | 6.162_333.54471 | 1.85 |
| 7.99_625.30792 | 1.96 | 5.073_234.13492 | 1.76 | 7.185_579.1344 | 1.85 | 8.992_287.05542 | 1.84 |
| 0.612_268.98364 | 1.96 | 6.432_273.15973 | 1.75 | 6.167_727.08295 | 1.85 | 4.265_355.10229 | 1.84 |
| 5.084_639.17804 | 1.93 | 5.73_291.1705 | 1.75 | 6.164_608.73602 | 1.84 | 7.179_581.15118 | 1.83 |
| 8.892_519.24518 | 1.90 | 6.433_245.12846 | 1.73 | 6.166_609.14581 | 1.84 | 6.144_781.26111 | 1.83 |
| 0.611_270.98145 | 1.88 | 10.166_439.26947 | 1.72 | 7.187_615.11145 | 1.84 | 6.165_633.14362 | 1.83 |
| 8.823_533.22479 | 1.86 | 6.67_475.18604 | 1.72 | 6.479_656.14661 | 1.83 | 6.17_345.57056 | 1.83 |
| 9.021_445.24432 | 1.84 | 0.741_198.07602 | 1.70 | 0.736_187.0247 | 1.83 | 6.169_334.06293 | 1.83 |
| 8.6_607.29919 | 1.83 | 0.657_400.1813 | 1.70 | 7.9_599.11743 | 1.83 | 6.163_649.11646 | 1.82 |
| 5.558_723.15637 | 1.83 | 6.725_259.14444 | 1.69 | 7.077_648.04504 | 1.83 | 6.16_152.78871 | 1.82 |
| 6.594_475.18668 | 1.83 | 0.655_704.21198 | 1.69 | 5.651_789.18878 | 1.83 | 6.162_611.15985 | 1.82 |
| 8.654_489.27164 | 1.82 | 6.541_478.20627 | 1.69 | 7.078_585.10297 | 1.83 | 4.267_356.10623 | 1.82 |
| 8.871_547.31335 | 1.81 | 7.187_305.18604 | 1.69 | 6.163_609.55206 | 1.82 | 5.726_464.21283 | 1.82 |
| 8.329_341.19644 | 1.80 | 6.546_477.20203 | 1.69 | 5.873_740.65057 | 1.82 | 6.691_595.16577 | 1.82 |
| 9.412_260.12964 | 1.79 | 9.591_275.2005 | 1.69 | 5.871_741.11945 | 1.82 | 0.84_426.00934 | 1.82 |
| 10.098_543.28113 | 1.79 | 0.658_867.28119 | 1.68 | 6.136_584.12433 | 1.82 | 5.902_1321.3429 | 1.82 |
| 8.525_535.27533 | 1.79 | 6.431_216.1022 | 1.68 | 6.482_631.12451 | 1.82 | 7.897_565.15509 | 1.81 |
| 8.522_401.25525 | 1.77 | 9.183_369.25378 | 1.67 | 7.544_533.09357 | 1.82 | 7.449_581.151 | 1.81 |
| 0.682_163.02501 | 1.77 | 0.658_696.22528 | 1.67 | 7.186_642.13037 | 1.82 | 7.066_419.0979 | 1.81 |
| 0.735_331.06641 | 1.76 | 6.726_216.10204 | 1.67 | 7.46_615.11133 | 1.81 | 0.732_423.19717 | 1.81 |
| 0.658_369.1026 | 1.76 | 10.119_328.24738 | 1.67 | 0.692_149.04536 | 1.81 | 6.412_289.07068 | 1.80 |

**Table S12.** Features of aqueous phase with their respective VIP (PLS-DA). VIP > 1.60 based on positive ionization mode. (cont.)

| **VIP based on seasons** | | | | | | | | **VIP based on cultivation condition** | | | | | | |
| --- | --- | --- | --- | --- | --- | --- | --- | --- | --- | --- | --- | --- | --- | --- |
| **Negative ionization mode** | | | | **Positive ionization mode** | | | | **Negative ionization mode** | | | | **Positive ionization mode** | | |
| **RT_*m/z*** | **VIP** | | **RT_*m/z*** | | **VIP** | | **RT_*m/z*** | | **VIP** | | **RT_*m/z*** | | **VIP** | |
| 6.153_271.15576 | | 1.76 | | 0.621_481.08084 | | 1.65 | | 6.153_609.21338 | | 1.81 | | 5.874_871.23022 | | 1.80 |
| 0.939_300.03595 | | 1.76 | | 9.588_293.21054 | | 1.65 | | 6.124_725.1944 | | 1.81 | | 6.477_595.16602 | | 1.80 |
| 8.38_463.25635 | | 1.76 | | 9.094_291.19501 | | 1.65 | | 6.171_723.13947 | | 1.81 | | 6.088_271.07919 | | 1.80 |
| 4.53_153.01952 | | 1.76 | | 0.648_432.17032 | | 1.65 | | 0.922_128.03542 | | 1.81 | | 6.155_317.0683 | | 1.80 |
| 0.69_515.12555 | | 1.76 | | 0.626_575.1662 | | 1.65 | | 6.484_661.1369 | | 1.81 | | 5.311_923.24487 | | 1.80 |
| 6.488_243.11403 | | 1.76 | | 0.653_533.65643 | | 1.65 | | 7.546_489.10309 | | 1.81 | | 6.458_287.05444 | | 1.80 |
| 0.741_300.03622 | | 1.75 | | 0.63_288.58789 | | 1.64 | | 6.483_593.14966 | | 1.81 | | 0.629_363.1134 | | 1.80 |
| 0.698_239.077 | | 1.75 | | 0.654_525.16724 | | 1.64 | | 7.883_447.09259 | | 1.81 | | 6.163_630.13605 | | 1.79 |
| 0.672_239.07771 | | 1.75 | | 6.404_611.22491 | | 1.64 | | 6.7_593.15295 | | 1.81 | | 0.731_304.17526 | | 1.79 |
| 9.09_385.25876 | | 1.75 | | 0.657_488.19641 | | 1.64 | | 7.078_549.12646 | | 1.80 | | 5.754_1155.27734 | | 1.79 |
| 0.648_324.13077 | | 1.75 | | 0.652_1065.3114 | | 1.63 | | 0.742_274.05695 | | 1.80 | | 6.014_749.27075 | | 1.79 |
| 7.417_317.16074 | | 1.75 | | 0.63_288.08749 | | 1.63 | | 6.323_373.14987 | | 1.80 | | 7.572_447.19049 | | 1.79 |
| 7.424_231.12358 | | 1.75 | | 0.653_533.15601 | | 1.63 | | 5.873_741.27716 | | 1.80 | | 6.123_727.2063 | | 1.79 |
| 6.46_243.11389 | | 1.74 | | 10.728_432.23831 | | 1.62 | | 5.871_804.18347 | | 1.80 | | 6.46_449.1076 | | 1.79 |
| 8.734_289.16599 | | 1.74 | | 0.644_287.07031 | | 1.62 | | 5.869_767.6427 | | 1.80 | | 7.54_535.1087 | | 1.79 |
| 5.99_167.03488 | | 1.73 | | 0.655_525.66693 | | 1.62 | | 7.901_563.14014 | | 1.80 | | 5.933_291.08591 | | 1.78 |
| 6.348_443.15546 | | 1.73 | | 0.928_307.08383 | | 1.61 | | 7.079_612.11908 | | 1.80 | | 6.418_884.24097 | | 1.78 |
| 10.379_593.2735 | | 1.72 | | 9.892_431.2189 | | 1.61 | | 5.875_741.18646 | | 1.80 | | 7.069_551.13818 | | 1.78 |
| 0.699_142.99843 | | 1.72 | | 10.093_309.20587 | | 1.61 | | 7.077_587.09863 | | 1.80 | | 6.415_867.2121 | | 1.78 |
| 0.678_445.12006 | | 1.72 | | 9.074_346.25784 | | 1.61 | | 6.197_607.12872 | | 1.80 | | 0.74_158.11749 | | 1.77 |
| 0.725_545.13629 | | 1.71 | | 9.62_499.35229 | | 1.61 | | 6.139_1335.34375 | | 1.79 | | 7.88_449.1084 | | 1.77 |
| 0.638_147.02982 | | 1.71 | | 9.076_351.21356 | | 1.61 | | 7.459_579.1344 | | 1.79 | | 7.45_303.04996 | | 1.77 |
| 0.978_219.0144 | | 1.71 | | 0.657_777.75281 | | 1.60 | | 6.157_1219.29822 | | 1.79 | | 6.112_765.26556 | | 1.77 |
| 0.61_217.02989 | | 1.71 | | 0.641_381.07883 | | 1.60 | | 6.458_617.1084 | | 1.79 | | 5.932_289.0708 | | 1.77 |
| 8.626_547.31268 | | 1.71 | | 5.884_477.2009 | | 1.60 | | 7.077_667.06262 | | 1.79 | | 6.977_767.28308 | | 1.77 |
| 0.618_277.03314 | | 1.71 | | 0.626_591.14374 | | 1.60 | | 5.868_825.15125 | | 1.79 | | 5.958_449.1077 | | 1.76 |

**Table S13.** Features of organic phase with their respective VIP (PLS-DA) based on seasons. VIP > 1.60 based on negative ionization mode.

| **Negative ionization mode** | | **Positive ionization mode** | |
| --- | --- | --- | --- |
| **RT_*m/z*** | **VIP** | **RT_*m/z*** | **VIP** |
| 10.353_843.61145 | 2.39 | 1.712_365.26883 | 4.26 |
| 10.352_844.61444 | 2.19 | 0.963_442.31616 | 4.09 |
| 10.363_517.4624 | 2.18 | 8.981_670.5791 | 3.98 |
| 6.551_727.49249 | 2.13 | 8.966_642.50897 | 3.97 |
| 6.868_727.4917 | 2.12 | 10.315_824.69019 | 3.97 |
| 4.302_701.43909 | 2.05 | 8.484_742.5979 | 3.91 |
| 0.622_219.01443 | 2.01 | 0.633_284.15005 | 3.85 |
| 0.755_499.25479 | 2.01 | 1.798_365.26913 | 3.85 |
| 0.87_451.22827 | 2.01 | 8.305_291.19559 | 3.78 |
| 0.62_153.01915 | 2.01 | 8.974_712.62366 | 3.77 |
| 0.62_287.0567 | 2.00 | 10.108_295.22595 | 3.74 |
| 0.867_449.23026 | 1.99 | 0.802_329.18768 | 3.73 |
| 10.214_721.57733 | 1.99 | 8.973_698.60968 | 3.67 |
| 1.522_605.23987 | 1.99 | 1.673_625.26471 | 3.67 |
| 9.098_895.62775 | 1.97 | 0.899_294.24319 | 3.64 |
| 0.613_269.04343 | 1.95 | 0.958_419.24106 | 3.63 |
| 0.916_405.24017 | 1.94 | 0.966_414.284 | 3.62 |
| 8.966_609.48853 | 1.93 | 6.128_617.5033 | 3.62 |
| 0.618_142.99844 | 1.92 | 0.926_655.39075 | 3.60 |
| 1.496_374.3269 | 1.91 | 8.989_633.48645 | 3.59 |
| 2.114_699.42554 | 1.91 | 9.119_588.4986 | 3.59 |
| 2.269_563.22882 | 1.89 | 1.353_829.50598 | 3.56 |
| 0.593_591.11383 | 1.87 | 1.093_333.18259 | 3.55 |
| 10.716_862.62036 | 1.86 | 0.74_268.22733 | 3.53 |
| 8.965_655.49414 | 1.86 | 6.092_617.50336 | 3.52 |
| 2.298_701.44006 | 1.86 | 8.071_609.48584 | 3.52 |
| 0.922_415.26974 | 1.86 | 1.329_368.31555 | 3.49 |
| 8.257_651.52075 | 1.86 | 10.859_715.60107 | 3.48 |
| 1.668_623.25165 | 1.85 | 8.488_770.63074 | 3.47 |
| 8.475_723.5578 | 1.84 | 9.727_618.58173 | 3.46 |
| 0.694_307.19162 | 1.83 | 8.937_277.21655 | 3.45 |
| 0.6_226.99632 | 1.83 | 6.087_618.50531 | 3.45 |
| 5.592_814.46558 | 1.82 | 9.092_634.57733 | 3.43 |
| 10.318_763.60358 | 1.82 | 0.706_481.34171 | 3.41 |
| 1.331_307.22739 | 1.82 | 8.979_656.56158 | 3.40 |
| 1.692_629.23846 | 1.82 | 8.065_649.47894 | 3.39 |
| 0.617_173.00926 | 1.81 | 0.908_467.36203 | 3.39 |
| 0.877_359.1839 | 1.79 | 8.971_611.50262 | 3.38 |
| 8.506_803.55084 | 1.78 | 0.695_224.18527 | 3.36 |
| 1.691_697.22479 | 1.77 | 8.968_628.52881 | 3.36 |
| 10.328_764.61041 | 1.77 | 10.319_803.57117 | 3.35 |
| 0.764_343.16785 | 1.77 | 0.928_392.24954 | 3.35 |
| 0.923_609.4137 | 1.77 | 0.904_313.17725 | 3.34 |
| 1.229_741.43365 | 1.76 | 5.979_617.50354 | 3.33 |
| 8.911_531.4425 | 1.76 | 2.916_303.28922 | 3.33 |
| 8.366_669.45111 | 1.76 | 0.804_364.22476 | 3.32 |
| 0.844_503.28485 | 1.76 | 10.946_1202.33215 | 3.30 |
| 0.922_587.43292 | 1.75 | 8.064_672.55548 | 3.29 |
| 8.507_800.54541 | 1.75 | 1.699_631.25061 | 3.28 |
| 8.367_737.43945 | 1.74 | 10.788_714.58533 | 3.28 |
| 10.174_769.59894 | 1.74 | 9.776_293.21048 | 3.26 |
| 10.614_545.49261 | 1.73 | 10.629_685.59247 | 3.26 |

**Table S13.** Features of organic phase with their respective VIP (PLS-DA) based on seasons. VIP > 1.60 based on negative ionization mode. (cont.)

| **Negative ionization mode** | | **Positive ionization mode** | |
| --- | --- | --- | --- |
| **RT_*m/z*** | **VIP** | **RT_*m/z*** | **VIP** |
| 1.575_653.26288 | 1.73 | 5.04_589.48163 | 3.26 |
| 0.599_129.01921 | 1.73 | 0.705_245.11446 | 3.25 |
| 7.238_734.47571 | 1.73 | 10.786_1058.28625 | 3.25 |
| 8.658_712.49329 | 1.72 | 10.293_903.56403 | 3.25 |
| 0.994_329.18915 | 1.72 | 1.581_610.27521 | 3.24 |
| 8.152_736.49072 | 1.72 | 1.306_447.2518 | 3.24 |
| 0.921_363.15079 | 1.72 | 1.099_351.25336 | 3.23 |
| 6.444_691.43677 | 1.72 | 10.874_913.67053 | 3.23 |
| 6.346_730.50421 | 1.72 | 10.72_659.57678 | 3.23 |
| 2.863_893.51184 | 1.71 | 9_649.45905 | 3.23 |
| 0.781_323.22308 | 1.71 | 8.068_728.61914 | 3.22 |
| 1.575_675.24286 | 1.71 | 9.726_555.51276 | 3.22 |
| 0.758_501.27136 | 1.71 | 10.254_911.55688 | 3.22 |
| 6.475_1003.62054 | 1.71 | 0.801_465.34839 | 3.21 |
| 0.993_397.17572 | 1.71 | 6.248_462.4296 | 3.21 |
| 0.888_289.18121 | 1.71 | 2.284_565.2453 | 3.21 |
| 9.567_899.66034 | 1.71 | 3.406_806.53912 | 3.21 |
| 8.508_802.55139 | 1.70 | 3.356_811.49414 | 3.18 |
| 1.44_267.19659 | 1.70 | 10.704_108.91933 | 3.18 |
| 1.226_481.25735 | 1.70 | 5.161_405.12933 | 3.18 |
| 1.67_645.2326 | 1.70 | 5.01_772.54767 | 3.18 |
| 1.67_564.23889 | 1.70 | 2.918_348.34723 | 3.17 |
| 4.165_508.33975 | 1.70 | 8.07_714.60449 | 3.17 |
| 0.875_273.186 | 1.69 | 0.759_439.2659 | 3.17 |
| 9.703_899.6604 | 1.69 | 9.255_875.63696 | 3.17 |
| 0.874_247.20653 | 1.69 | 0.809_311.17703 | 3.16 |
| 0.58_189.00423 | 1.69 | 8.394_761.62421 | 3.15 |
| 9.333_533.45551 | 1.69 | 10.588_100.69492 | 3.14 |
| 0.998_465.16248 | 1.68 | 10.067_557.52997 | 3.14 |
| 1.692_607.25677 | 1.68 | 1.789_341.26624 | 3.14 |
| 0.585_228.9931 | 1.68 | 11.004_1276.35095 | 3.12 |
| 8.005_831.50049 | 1.68 | 9.727_595.50433 | 3.12 |
| 10.848_1419.9342 | 1.68 | 0.918_409.23514 | 3.11 |
| 5.167_637.43323 | 1.67 | 4.216_360.32526 | 3.09 |
| 0.641_273.09799 | 1.67 | 8.49_725.57428 | 3.09 |
| 1.575_697.22479 | 1.67 | 8.372_612.53479 | 3.08 |
| 4.762_561.37939 | 1.67 | 0.924_317.20828 | 3.08 |
| 1.115_623.25159 | 1.67 | 2.621_535.27081 | 3.06 |
| 8.133_687.46112 | 1.67 | 10.059_597.52167 | 3.04 |
| 6.047_730.50476 | 1.67 | 10.321_788.60321 | 3.04 |
| 8.37_805.42365 | 1.66 | 0.668_198.12758 | 3.04 |
| 0.923_351.17001 | 1.66 | 1.314_479.23978 | 3.03 |
| 0.994_331.18546 | 1.66 | 0.694_289.17941 | 3.03 |
| 10.284_969.53601 | 1.66 | 10.292_100.39534 | 3.02 |
| 1.321_524.28497 | 1.65 | 10.339_747.60498 | 3.02 |
| 6.012_819.56036 | 1.65 | 1.349_277.21625 | 3.01 |
| 0.598_575.12 | 1.65 | 10.768_960.80475 | 2.99 |
| 0.613_289.07184 | 1.64 | 0.9_336.25284 | 2.99 |
| 0.809_345.18341 | 1.64 | 2.941_794.53027 | 2.99 |
| 7.86_553.42737 | 1.64 | 0.786_403.18799 | 2.99 |
| 5.684_725.47772 | 1.64 | 1.698_609.27155 | 2.98 |
| 8.649_860.53864 | 1.63 | 10.049_763.63635 | 2.98 |

**Table S13.** Features of organic phase with their respective VIP (PLS-DA) based on seasons. VIP > 1.60 based on negative ionization mode. (cont.)

| **Negative ionization mode** | | **Positive ionization mode** | |
| --- | --- | --- | --- |
| **RT_*m/z*** | **VIP** | **RT_*m/z*** | **VIP** |
| 1.377_589.24677 | 1.62 | 9.727_611.47748 | 2.98 |
| 7.767_693.44977 | 1.62 | 10.801_1059.28857 | 2.97 |
| 0.759_455.26474 | 1.62 | 0.994_623.24878 | 2.96 |
| 1.304_499.29166 | 1.62 | 10.783_713.58405 | 2.94 |
| 7.013_483.38464 | 1.62 | 2.914_320.31525 | 2.93 |
| 7.944_564.40399 | 1.62 | 10.874_1128.31531 | 2.92 |
| 4.749_753.43262 | 1.62 | 9.732_590.54901 | 2.92 |
| 0.925_293.34482 | 1.62 | 1.218_609.27081 | 2.91 |
| 8.155_872.46655 | 1.62 | 11.054_1350.37012 | 2.90 |
| 0.612_383.08383 | 1.62 | 0.602_435.09229 | 2.89 |
| 8.987_697.48145 | 1.61 | 0.652_365.10519 | 2.89 |
| 0.819_413.17224 | 1.61 | 6.245_467.38553 | 2.88 |
| 8.622_738.50922 | 1.61 | 1.107_339.18991 | 2.87 |
| 0.815_413.25415 | 1.61 | 8.53_427.39252 | 2.86 |
| 3.563_655.44287 | 1.60 | 4.072_310.31049 | 2.86 |
| 8.508_868.53217 | 1.60 | 10.731_814.57141 | 2.86 |
